# Cancer testis antigens: Emerging therapeutic targets leveraging genomic instability in cancer

**DOI:** 10.1016/j.omton.2024.200768

**Published:** 2024-01-26

**Authors:** Adviti Naik, Boucif Lattab, Hanan Qasem, Julie Decock

**Affiliations:** 1 Cancer Research Center, Qatar Biomedical Research Institute (QBRI), Hamad Bin Khalifa University (HBKU), Qatar Foundation, Doha, Qatar; 2 College of Health and Life Sciences (CHLS), Qatar Biomedical Research Institute (QBRI), Hamad Bin Khalifa University (HBKU), Doha, Qatar

**Keywords:** MT: Regular Issue, cancer testis antigen, genomic integrity, DNA repair, tumor cellular fitness, therapeutic intervention

## Abstract

Cancer care has witnessed remarkable progress in recent decades, with a wide array of targeted therapies and immune-based interventions being added to the traditional treatment options such as surgery, chemotherapy, and radiotherapy. However, despite these advancements, the challenge of achieving high tumor specificity while minimizing adverse side effects continues to dictate the benefit-risk balance of cancer therapy, guiding clinical decision making. As such, the targeting of cancer testis antigens (CTAs) offers exciting new opportunities for therapeutic intervention of cancer since they display highly tumor specific expression patterns, natural immunogenicity and play pivotal roles in various biological processes that are critical for tumor cellular fitness. In this review, we delve deeper into how CTAs contribute to the regulation and maintenance of genomic integrity in cancer, and how these mechanisms can be exploited to specifically target and eradicate tumor cells. We review the current clinical trials targeting aforementioned CTAs, highlight promising pre-clinical data and discuss current challenges and future perspectives for future development of CTA-based strategies that exploit tumor genomic instability.

## Teaser

Decock and colleagues discuss the diverse roles of cancer testis antigens in the regulation of genomic stability and their therapeutic potential as modulators of tumor cellular fitness. Their pro-tumorigenic functions along with highly tumor-specific expression make CTAs great candidate targets for specific eradication of tumor cells with minimal adverse effects.

## Graphical abstract

**Figure:**
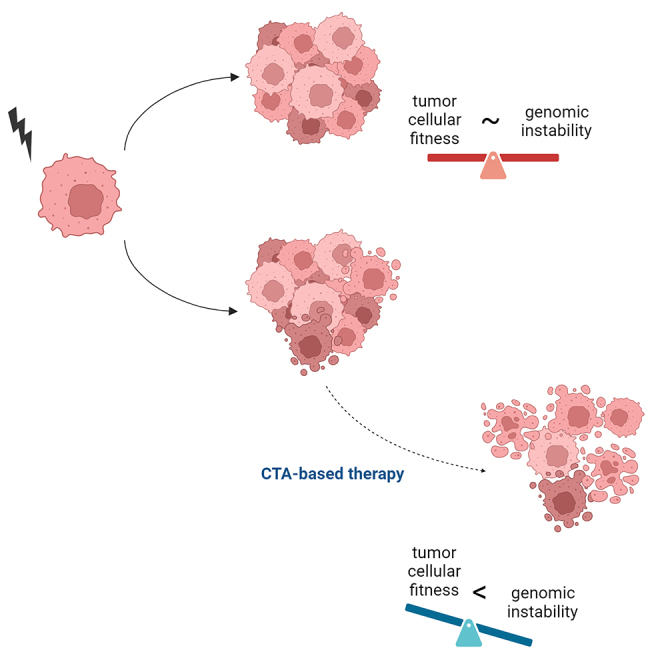
The various roles of CTAs in regulating and maintaining genomic integrity provide novel opportunities for therapeutic intervention by tipping the balance of tumor cellular fitness towards unsustainable genomic instability.

## Introduction

Cancer-testis antigens (CTA) are a large family of tumor-associated proteins that under physiological conditions are predominantly expressed in the testes, specifically in the proliferating germ cells, spermatogonia and spermatocytes.^1^^,^^2^^,^^3^^,^^4^^,^^5^^,^^6^^,^^7^ Their expression is tightly controlled by DNA methylation and histone modifications involving epigenetic modulatory proteins such as the germ-cell specific CCCTC-binding factor (CTCF) and Brother of Regulator of Imprinted Sites (BORIS).^4^^,^^8^^,^^9^^,^^10^^,^^11^ Furthermore, aberrant expression of CTAs in tumors may depend on chromosomal location since chromosome X-encoded CTAs, encompassing the majority of multigene CTA families (MAGE/GAGE/PAGE/XAGE, NY-ESO-1 and SSX genes), are more frequently expressed in tumors compared to autosomal single-copy CTA genes (non-CT-X).^12^ While CTA expression is a recurrent observation in tumors, the extent of their expression differs between cancers as well as between tumors of the same cancer type.^13^^,^^14^^,^^15^ Based on the frequency of CTA expression, cancers can be grouped into CTA-rich and -poor subgroups.^16^ CTA-rich cancers include melanoma, lung cancer, hepatocellular carcinoma, germ cell cancer, gastric cancer, and chondrosarcoma with a CTA expression frequency of at least 50%. On the other hand, leukemia, lymphoma, renal carcinoma, glioblastoma, and colon carcinoma constitute CTA-poor cancer types with expression frequencies of less than 20%. Interestingly, da Silva et al. reported that 17% of all CTAs show exclusive expression in a single tumor type.^14^ For instance, they identified 32 CTAs that were expressed solely in leukemia, eleven in melanoma, and fourteen in ovarian cancer; thus, suggesting tumor-type specific roles for CTAs. In addition, CTA expression shows heterogeneous patterns within a single cancer type. For example, in breast cancer, the CTAs *CXorf61*, *HORMAD1*, *ACTL8* and *PRAME* are specifically enriched in basal subtypes, while the expression of *PLAC1* and *POTEC* is more frequently observed in non-basal subtypes.^15^

Overall, CTA expression has been associated with worse clinical outcome.^5^^,^^8^^,^^12^ For example, LDHC expression has been correlated with poor prognosis in breast cancer,^17^ lung cancer,^18^ renal cell carcinoma^19^ and hepatocellular carcinoma.^20^ In contrast, few CTAs have been correlated with a better prognosis such as ACTL8, OIP5, XAGE3 and CTCFL in glioblastoma.^21^ Finally, a select few show a differential prognostic value based on the tumor type; for instance, NY-ESO-1 is associated with favorable prognosis in melanoma but poor outcome in other tumor types.^5^ These divergent findings suggest that CTAs exhibit pro- and anti-tumorigenic functions in a context-dependent manner. To date, sparse information is available on anti-tumorigenic functions of CTAs with few studies reporting that TSAG10, RGS22, MAGE-A4 and SPANXA restrict tumorigenesis through the inhibition of tumor metabolic activity, proliferation and metastasis.^22^^,^^23^^,^^24^^,^^25^^,^^26^ Traditionally, CTAs are thought to predominantly support cancer hallmarks such as sustaining proliferative signaling, resisting cell death, deregulating cellular energetics, activating invasion and metastasis, inducing angiogenesis, and genome instability and mutation.^5^^,^^6^^,^^7^^,^^8^^,^^12^^,^^27^^,^^28^ For instance, members of the MAGE family have been shown to, in part through binding of the master tumor suppressor p53, promote tumor cell proliferation and cell cycle progression while inhibiting tumor cell survival.^29^^,^^30^ SSX and CAGE family members increase tumor cell growth and survival through the activation of the MAPK and Wnt signaling pathways and upregulation of cell cycle proteins.^31^^,^^32^^,^^33^ CTAs have also been implicated in blocking cellular senescence programs, allowing cancer cells to bypass several checkpoints that are crucial to suppress tumorigenesis, and promoting epithelial-mesenchymal transition, leading to an increased migratory and invasive potential of cancer cells.^34^^,^^35^^,^^36^ A growing body of evidence further indicates that expression of CTAs in tumors affects tumor cellular fitness by enhancing genomic instability. Indeed, genomic integrity is a critical characteristic of both tumor cells and germ cells which has a profound impact on the transmission of error-free hereditary information and defines cellular fitness.^37^^,^^38^

Given their highly tumor specific expression and pro-tumorigenic functions, CTA-based therapy provides novel opportunities for potent treatment responses with minimal adverse effects. This review specifically highlights the current knowledge on CTAs that play a role in regulating genomic integrity and discusses current and emerging therapeutic approaches targeting these specific CTAs to improve the clinical outcome of cancer patients.

## Genomic integrity-regulatory CTAS

**Figure 1:**
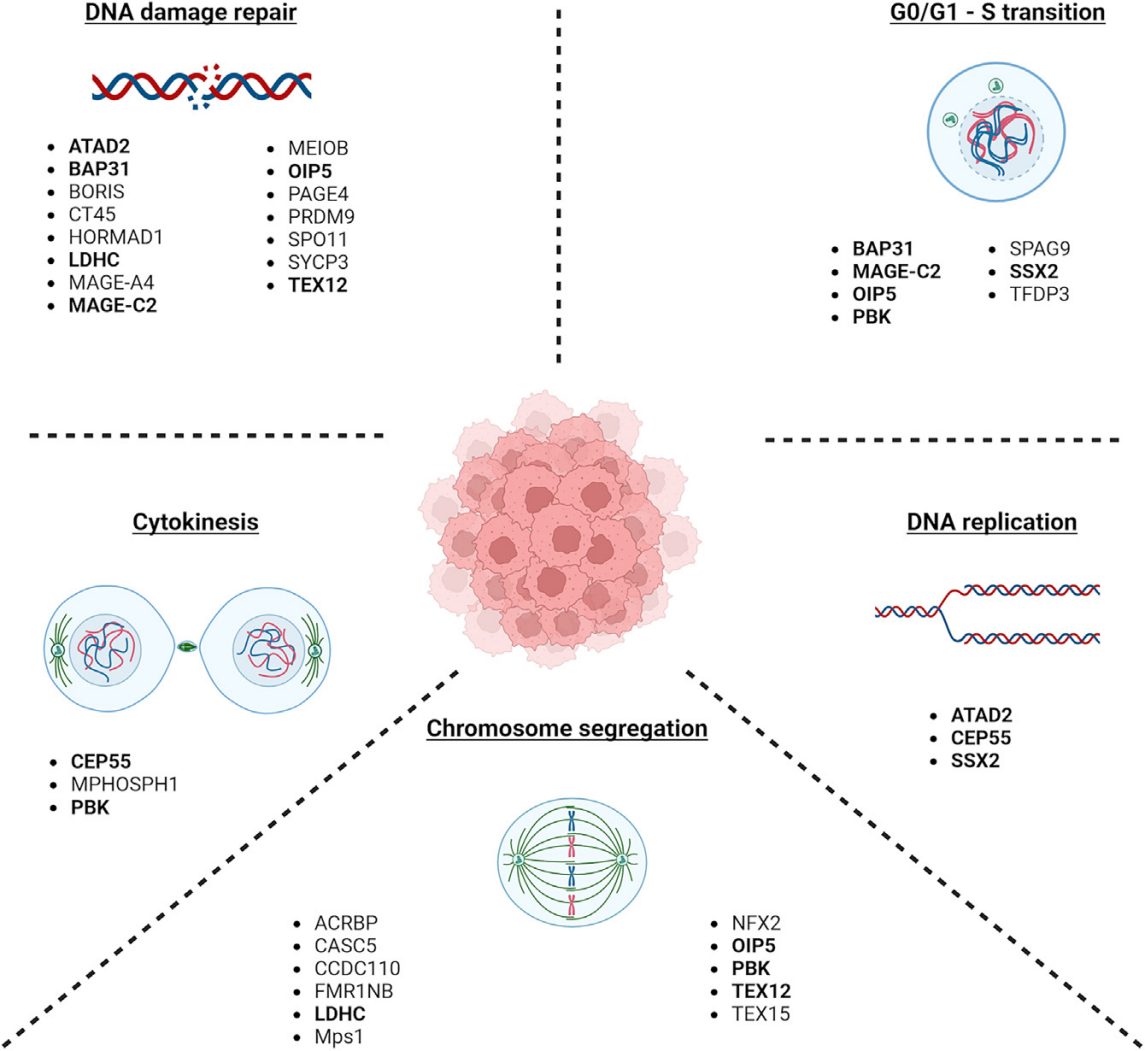
Diverse roles of cancer testis antigens in biological processes that regulate genomic integrity in cancer Tumoral expression of distinct CTAs impacts tumor cellular fitness through their role in DNA damage repair, G0/G1-S cell cycle transition, DNA replication, chromosome segregation and cytokinesis. CTAs highlihgted in bold exert multiple regulatory functions.

Genomic instability is a major driving force of tumorigenesis and tumor progression as it leads to the further accumulation of genomic and chromosomal alterations that promote the acquisition of tumor features and cancer hallmarks. This increased propensity for genomic alterations often results from DNA damage introduced by extrinsic, environmental (mutagenic chemical agents, ultraviolet radiation) or intrinsic factors (replication errors, oxidative stress, spontaneous hydrolysis). As such, normal cells utilize various mechanisms to minimize transmission of genetic errors to daughter cells.^39^^,^^40^ Central to these is the tight regulation of cell division whereby cell cycle checkpoints either induce cell-cycle arrest to promote repair, or trigger apoptosis or senescence in case of excessive damage. Aberrant expression of CTAs has been shown to dysregulate cell cycle surveillance, leading to enhanced genomic instability. This review provides a comprehensive overview of the expression, clinical relevance and molecular function of CTAs that have been implicated in regulating genomic integrity through dysregulation of DNA damage repair, G0/G1-S phase transition, DNA replication, chromosome segregation and cytokinesis (Figure 1; Table S1). A few CTAs, highlighted in bold, play a role in multiple regulatory mechanisms and are discussed separately at the end of this section.

### DNA damage repair

DNA damage repair is critical to cell cycle progression of normal and malignant cells and can be triggered at several points during the cell cycle by DNA damage checkpoints. The G1 checkpoint ensures that cells do not undergo replication until DNA damage is repaired, while the S and G2 checkpoints prevent cells with damaged DNA from undergoing mitosis. Of note, there is a fine balance between the tolerable levels of DNA damage that drive oncogenic transformation and excessive levels that ultimately induce cancer cell senescence or cell death. Aberrant tumor expression of CTAs has been found to differentially regulate genomic integrity in a context-dependent manner and is thus associated with either a better or worse clinical outcome in different cancers. Mechanistically, CTAs have been shown on one hand to impair DNA damage checkpoints, resulting in the propagation of cells with unresolved DNA damage and enhancing genomic instability, and on the other hand to promote the DNA damage response in order to enhance tumor cellular fitness. Therefore, caution is warranted when targeting these particular CTAs for which the tumor context should be taken into consideration. When favorable, targeting of CTAs could be combined with DNA damage response-related drugs to improve treatment response and clinical outcome. We recently provided experimental evidence to support this approach, whereby silencing of LDHC greatly sensitized breast cancer cells to treatment with cisplatin and olaparib.^17^

#### BORIS/CTCFL/CT27

Brother of the Regulator of Imprinted Sites (BORIS) is abnormally expressed in lung, breast, hepatocellular and cervical carcinoma and is associated with worse prognosis. The aberrant expression of BORIS in cancer cells supports tumor progression through enhanced cell proliferation, cell survival and DNA damage repair. Its expression has been shown to promote colorectal cancer cell proliferation, reduce apoptosis and induce fluorouracil (5-FU) resistance.^41^ In accordance, depletion of BORIS in a colorectal cancer mouse model suppressed DNA damage repair and promoted apoptosis.^42^ Furthermore, overexpression of BORIS in non-small cell lung cancer cell lines was shown to suppress cisplatin-induced DNA damage, likely through upregulation of the mismatch repair factor mutS homolog 6 (MSH6).^43^ Conversely, silencing of BORIS increased DNA damage, inhibited cell proliferation and enhanced treatment response.

#### CT45/CT45A1

Cancer testis antigen 45 (CT45) is highly expressed in ovarian cancer, lung cancer, endometrial cancer and to a lesser extent in breast cancer and is correlated with poor prognosis. In high-grade serous ovarian cancer, CT45 has been implicated in the DNA damage response through its inhibition of the PP4 phosphatase complex, leading to increased DNA damage and worse clinical outcome.^44^ On the other hand, this rise in genomic instability can be exploited for therapeutic purposes in combination with platinum-based chemotherapy, exacerbating DNA damage and inducing cell death followed by antigen release and T cell activation. In contrast, CT45 expression in endometrial cancer has been linked to cancer cell stemness and paclitaxel resistance through its co-expression and upregulation by Y-Box binding proteins (YBX2).^45^

#### HORMAD1/NOHMA/CT46

HORMA domain-containing protein 1 (HORMAD1) was identified as the first meiotic checkpoint gene that is essential for double strand break-dependent homologous recombination and accurate chromosome segregation,.^46^ Overall, HORMAD1 expression is upregulated in cancer, including gastric cancer, lung cancer, breast cancer and ovarian cancer, and has been associated with increased genomic instability and worse overall survival. Notably, HORMAD1 can regulate DNA double-strand break repair in cancer cells through either homologous recombination or non-homologous end-joining. In lung cancer, HORMAD1 promotes homologous recombination to mitigate DNA damage and protect stalled replication forks from excessive MRE1-mediated nucleolytic degradation, while loss of HORMAD1 significantly reduces tumor growth *in vivo* and enhances sensitivity to irradiation and PARP inhibition.^47^^,^^48^ In contrast, in triple negative breast cancer, HORMAD1 suppresses homologous recombination and induces non-homologues end joining repair, hence sensitizing cancer cells to the use of homologous recombination-targeting therapy such as platinum-based chemotherapy.^49^^,^^50^ In addition, HORMAD1 compromises DNA mismatch repair of single strand breaks through cytosolic retention of the MCM8–MCM9 complex and reduction of MLH1 chromatin binding.^51^ HORMAD1 also enhances DNA damage tolerance resulting in increased dependency on replication stress tolerance pathways, such as *trans*-lesion synthesis, and thus induces resistance to chemotherapeutic drugs such as docetaxel.^52^^,^^53^ Furthermore, silencing of HORMAD improves sensitivity to cisplatin treatment.^54^

#### MAGE-A4/CT1.4

Melanoma-Associated Antigen 4 (MAGE-A4) is a member of the melanoma-associated antigen (MAGE) family of proteins and is expressed in lung cancer, breast cancer, bladder cancer, hepatocellular carcinoma, esophageal cancer, oral squamous cell carcinoma, gastrointestinal stromal tumors and gastric cancer, urothelial carcinoma, colorectal cancer, desmoid tumors, osteosarcoma, soft tissue sarcoma, synovial sarcoma, T cell leukemia/lymphoma, endometrial cancer, cervical cancer, ovarian cancer, vulvar cancer, uterine carcinosarcoma, salivary gland tumors, melanoma, and head and neck cancer. Overall, MAGE-A4 expression is associated with a poor prognosis, except for breast cancer and salivary gland carcinoma where it is linked to a favorable prognosis. In analogy with HORMAD1, MAGE-A4 has been shown to enhance DNA damage tolerance, in particular through binding and stabilization of the E3 ubiquitin ligase RAD18, resulting in enhanced PCNA mono-ubiquitination and subsequent activation of *trans*-lesion synthesis, one of the main effector pathways of DNA damage response.^55^ Oral squamous cell carcinoma cell lines expressing MAGE-A4 demonstrated lower treatment responses to docetaxel and paclitaxel.^56^

#### MEIOB/SPGF22

Meiosis Specific With OB-Fold (MEIOB) is a single strand DNA-binding protein that is vital for homologous recombination and faithful chromosome segregation during meiosis.^57^ MEIOB expression is positively correlated with the copy number aberrations in lung adenocarcinoma, bladder urothelial carcinoma, thyroid carcinoma, and uterine corpus endometrial carcinoma and its expression positively correlates with poor survival in triple negative breast cancer.^13^ In lung adenocarcinoma, MEIOB overexpression increases cell viability, proliferation, and the proportion of cells in the G2 phase.^13^ In contrast to its role in meiosis, MEIOB was shown to drive the error-prone non-homologous end-joining DNA repair mechanism while inducing homologous recombination deficiency, rendering cancer cells and xenografts more sensitive to treatment with PARP inhibitors.^58^

#### PAGE4/GAGEC1/CT16.7

Prostate Associated Gene 4 protein (PAGE4) is a transcription regulator of the c-Jun/AP-1/Fos signaling pathway that potentiates the transcription of prostate gland development genes.^59^ Expression of PAGE4 is upregulated in primary prostate tumors and was found to be associated with a reduced risk of prostate cancer recurrence and a favorable prognosis. Further, PAGE expression has been shown to attenuate androgen receptor signaling which likely impedes tumor progression to advanced stages.^60^ In contrast, in colorectal cancer, PAGE expression was shown to be significantly higher in primary tumors with liver metastasis.^61^ Under oxidative stress conditions, PAGE4 has been shown to protect prostate cancer cells from reactive oxygen species-induced DNA damage and apoptosis.^62^^,^^63^

#### PRDM9/PFM6

PR/SET Domain 9 (PRDM9) is a zinc finger histone methyltransferase that marks the localization of meiotic recombination hotspots.^64^ Expression of PRDM9 in tumors has been suggested to increase DNA double-strand breaks and genomic instability due to the enrichment of structural variant breakpoints and intersecting chromosome loop anchor points at sites of PRDM9 activity.^65^^,^^66^ PRDM9 is overexpressed in several cancers, including head and neck squamous cell carcinoma, bladder urothelial carcinoma, liver cancer and ovarian cancer. Moreover, rare allelic forms of PRDM9 have been reported in aneuploid and childhood B-cell precursor acute lymphoblastic leukemia.^67^

#### SPO11/CT35

SPO11 initiator of meiotic double-stranded breaks (SPO11) is a meiosis-specific endonuclease that catalyzes the formation of DNA double-strand breaks to initiate meiotic recombination.^68^ Disruption of SPO11 in murine spermatocytes results in defective meiosis, apoptosis and hence, severe gonadal abnormalities.^69^ SPO11 expression has been reported in cutaneous T cell lymphoma, colorectal cancer, melanoma, and cervical cancer. High expression of SPO11 in conjunction with EME2, MSH2 and MLH3 has been associated with worse prognosis in metastatic colorectal cancer. Although the molecular function of SPO11 in tumors has not yet been reported, it is likely that its role in double-strand break formation enhances genomic instability in cancer cells.

#### SYCP3/SCP3

Synaptonemal Complex Protein 3 (SYCP3) is a vital structural component of the synaptonemal complex that is formed between homologous chromosomes during meiosis.^70^ Expression of SYCP3 has been reported in various cancers including cervical cancer, acute lymphoblastic leukemia, non-small cell lung cancer, ovarian cancer and astrocytoma and is associated with shorter overall survival. SYCP3 expression has been linked to immune resistance and enhanced cancer stemness through Akt-mediated upregulation of anti-apoptotic molecules and Nanog.^71^^,^^72^ Sparse information is available on the role of SYCP3 in tumor genomic integrity except that is binds to BRCA2, which subsequently inhibits RAD51-dependent homologous recombination and confers hypersensitivity of cancer cells to PARP inhibition.^73^

### G0/G1-S cell cycle transition

As previously mentioned, transition from the G0/G1 to the S phase is tightly controlled by the G1 checkpoint to enable DNA damage repair prior to DNA replication. Inhibitors against the G1-S cyclin-dependent kinases CDK4/6 have shown potent anti-tumor activity in metastatic hormone receptor positive breast cancer, non-small-cell lung cancer, prostate cancer and acute myeloid leukemia while modest efficacy was observed in triple negative cancer, colorectal cancer and melanoma.^74^ Drug resistance poses a major challenge for CDK4/6 inhibitors and is often the result of compensatory mechanisms. Hence, it is imperative to advance our understanding of the molecular networks associated with the expression of CTAs that play a role in G1-S checkpoint regulation in order to inform the development of combination therapeutic approaches.

#### SPAG9/JIP-4/CT89

Sperm-associated antigen 9 (SPAG9) is aberrantly expressed in multiple cancers such as hepatocellular carcinoma, chronic myeloid leukemia, thyroid cancer, bladder cancer, endometrial cancer, gastric cancer, prostate cancer, non-melanoma skin cancer, osteosarcoma, salivary gland tumors and astrocytoma. Its expression has been correlated with poor prognosis in breast cancer, non-small cell lung cancer, gastric cancer, prostate cancer, and hepatocellular carcinoma. In contrast, SPAG9 expression in clear-cell renal cell carcinoma predicted a better overall survival where it was shown to promote autophagy and inhibit inflammatory responses.^75^ Furthermore, SPAG9 deficient mouse models demonstrated that SPAG9 plays a key role in regulating CD4+ T cells response to TCR stimulation.^76^ Silencing of SPAG9 has been shown to arrest cancer cells in G0/G1 or S phase and decrease expression of cyclins and cyclin-dependent kinases.^77^^,^^78^^,^^79^^,^^80^^,^^81^^,^^82^^,^^83^ SPAG9 silencing in combination with paclitaxel treatment synergistically inhibited ovarian cancer cell viability, and SPAG9 depletion in ovarian cancer xenograft mouse models significantly reduced tumor growth.^81^

#### TFDP3/DP4/HCA661/CT30

Transcription Factor Dimerization Partner 3 (TFDP3) is a E2F partner protein that, in contrast to other DP family members, downregulates E2F activity in response to DNA damage, inhibiting E2F-transcriptional activity, and E2F1-induced induced apoptosis and G1-S cell cycle progression.^84^^,^^85^^,^^86^^,^^87^^,^^88^ TFDP3 expression has been reported in prostate cancer, pancreatic cancer, breast cancer, hepatocellular carcinoma and gastric adenocarcinoma, the latter where it is associated with worse survival.

### DNA replication

Defects in replication due to replication stress such as altered replication fork progression, reduced replication fidelity and DNA breaks, can lead to genomic and chromosomal instability. Currently, three CTAs have been implicated in the regulation of DNA replication; ATAD2, CEP55 and SSX2. Each of these plays added roles in DNA damage repair, G0/G1-S cell cycle transition or cytokinesis and will be discussed in the section on multifunctional genomic integrity-regulatory CTAs.

### Chromosome segregation

Inaccurate chromosome segregation poses a great threat to genomic integrity as it can result in chromosome copy number alterations (aneuploidy, polyploidy), the formation of micronuclei and chromosomal structural aberrations. Anti-mitotic drugs have been used to treat multiple cancer types by interfering with microtubule stabilization, spindle formation, chromosome segregation and mitotic exit, driving cells toward mitotic arrest.^89^ While a proportion of arrested cells will undergo cell death, others may survive through mitotic slippage, eventually becoming genomically unstable or senescent, negatively affecting treatment response. Combination treatment of anti-mitotic drugs with distinct CTAs may help to improve treatment efficacy through further dysregulation of chromosome segregation and the spindle assembly checkpoint. For instance, a phase I study on combination treatment of taxanes with inhibitors targeting Mps1 demonstrated an anti-tumor activity in 32% of patients with solid tumors, establishing a precedent for further clinical trials.^90^

#### ACRBP/OY-TES-1/CT23

Acrosin binding protein (ACRBP) is a CTA that is abnormally expressed in tumors of many different tissue origins including the breast, bladder, colon, liver, lung, and ovaries and can induce specific cytotoxic cellular and humoral immune responses. High ACRBP expression in ovarian cancer correlates with reduced survival, earlier relapse and paclitaxel resistance.^91^ Molecularly, ACRBP tumor expression was found to be required for robust mitotic spindle assembly and function through its antagonistic interaction with the spindle-associated protein NuMa. As such, depletion of ACRBP in ovarian cancer cells treated with paclitaxel resulted in elevated NuMa levels, activating the spindle assembly checkpoint response, and leading to mitotic delay, mitotic catastrophe and subsequently enhanced treatment response.

#### CASC5/KNL1/KIAA1570/CT29

Cancer Susceptibility Candidate gene 5 protein (CASC5) is a scaffolding protein that is involved in kinetochore assembly, kinetochore-microtubule attachment, and chromosome segregation during mitosis.^92^^,^^93^ Pan-cancer analysis revealed aberrant CASC5 expression in papillary renal cell carcinoma, lung adenocarcinoma, pancreatic adenocarcinoma, thymoma and urinary bladder cancer. Further, high CASC5 expression is associated with advanced disease and poor overall survival in lung adenocarcinoma, with cell line models demonstrating reduced cancer cell proliferation upon silencing of CASC5.^94^ On the other hand, CASC5 expression is reduced in leukemia patients and is associated with overexpression of genes related to chemoresistance and disease progression.^95^

#### CCDC110/KM-HN-1/CT52

Coiled-Coil Domain Containing 110 (CCDC110) is expressed in numerous cancers including esophageal cancer, breast cancer, colon cancer, melanoma, hepatocellular carcinoma, gastric cancer, and pancreatic cancer. CCDC110 expression is localized at centrosomes during mitosis, supporting a role for CCDC110 in G2/M regulation and chromosome segregation.^96^ Further, CCDC110 overexpression has been shown to delay G2/M phase transition in osteosarcoma cells.^97^

#### FMR1NB/NY-SAR-35/CT37

Fragile X Mental Retardation 1 Neighbor (FMR1NB) expression has been observed in sarcoma, melanoma, esophageal cancer, lung cancer and breast cancer and correlates with advanced grade and poor prognosis in glioma. It plays an important role in chromosome segregation and mitotic fidelity,^98^ and its expression is regulated by promoter methylation.^99^ Silencing of FMR1NB reduces cancer cell proliferation and motility, induces cell death, and improves sensitivity to paclitaxel treatment.^98^^,^^100^^,^^101^^,^^102^

#### Mps1/TTK/CT96

Monopolar spindle 1 kinase (Mps1) is a spindle assembly checkpoint kinase that delays mitotic exit from the anaphase and corrects erroneous attachments to prevent chromosome missegregation by facilitating the recruitment of key checkpoint proteins to kinetochores and regulating kinetochore-microtubule attachments.^103^^,^^104^^,^^105^ As such, Mps1 inhibition selectively reduces cancer cell proliferation and increases cancer cell aneuploidy and cell death as a result of mitotic checkpoint override, chromosomal misalignment, destabilization of kinetochores and mitotic checkpoint complexes, accumulation of irreparable DNA damage and polyploidy.^104^^,^^105^^,^^106^^,^^107^^,^^108^^,^^109^^,^^110^^,^^111^^,^^112^^,^^113^^,^^114^ Mps1 expression has been observed in multiple myeloma, osteosarcoma, hepatocellular carcinoma, neuroblastoma, glioma and glioblastoma, prostate cancer, colon cancer, lung cancer, mesothelioma, pancreatic cancer, and has been associated with poor prognosis in multiple cancers. In contrast, high expression of Mps1 in triple negative breast tumors was found to correlate with a better prognosis, however, the mechanisms driving this phenotype remain unexplored. The small molecule Mps1 inhibitors, commonly referred to as TTK inhibitors, CFI-402257, MPI-0479605 and NMS-P715 have been found to reduce tumor growth in hepatocellular carcinoma, lung cancer, ovarian cancer and melanoma xenograft models.^106^^,^^107^^,^^108^^,^^115^ Further, a phase I study of the Mps1 inhibitor S81694 in 35 patients with advanced, metastatic solid tumors reported one patient with a complete response and 13 who had stable disease.^116^ Given the role of Mps1 in regulating the mitotic checkpoint complex, inhibition of Mps1 potentiates the activity of taxanes in preclinical setting.^117^^,^^118^^,^^119^^,^^120^^,^^121^^,^^122^ For instance, in prostate cancer cells Mps1 inhibition induced mitotic slippage and mitotic catastrophe of cancer cells that are undergoing prolonged mitotic block as a result of taxane-induced spindle assembly checkpoint activation.^119^ Furthermore, inhibition of Mps1 in p53-mutant breast cancer cells enhanced taxane treatment response through activation of the p53-dependent postmitotic spindle checkpoint as a result of loss of Mps1-mediated phosphorylation of p53 and MDM2-mediated p53 ubiquitination.^123^^,^^124^^,^^125^ Mps1 inhibition has also been shown to enhance radiotherapy response of glioblastoma through modulation of the activity of DNA damage repair pathways.^126^

#### NXF2/TAPL2/CT39

Nuclear RNA Export Factor 2 (NXF2) is well known as a key regulator of spermatogonial proliferation, stem cell population maintenance and meiotic progression.^127^ NXF2 expression has been found in esophageal squamous cell carcinoma, head and neck squamous cell carcinoma, seminomas, and estrogen receptor negative breast cancer. Although little is known about the role of NFX2 in cancer, a paclitaxel synthetic lethality screen identified NFX2 to be critical for accurate chromosome segregation in tumor cells.^98^

#### TEX15/SPGF25/CT42

Testis-expressed protein 15 (TEX15) plays a key role in the formation of the chromosomal synapsis during male meiosis where it regulates the localization of SYCP1, RAD51 and DMC1 to the synaptonemal complex synapse.^128^ In line with this, a nonsense mutation in TEX15, resulting in a truncated form of the protein, has been reported as causal in a familial infertility phenotype.^129^ In addition, two deleterious TEX15 genetic variants, Q1631H and c.7253dupT, have been associated with a higher risk of developing prostate and breast cancer respectively, further supporting a role for TEX15 in tumorigenesis.^130^^,^^131^

### Cytokinesis

Faithful transmission of chromosomes to daughter cells is dependent on the accurate segregation of replicated chromosomes to opposite spindle poles followed by the formation of two daughter cells during cytokinesis. Dysregulation of cytokinesis by CTAs may therefore increase genomic instability and could be used in combination with anti-mitotic drugs to compromise tumor cell survival.

#### MPHOSPH1/KIF20B/KRMP1/CT90

High expression of M-phase Phosphoprotein 1 (MPHOSPH1) has been observed in testicular germ cell cancer, bladder cancer, pancreatic cancer, hepatocellular carcinoma, colorectal cancer, oral cancer, renal cell carcinoma and breast cancer, where its expression directly correlates with poor prognosis. Silencing of MPHOSPH1 reduces proliferation and tumor growth, attenuates distant lung metastasis and improves sensitivity to taxol treatment.^132^^,^^133^^,^^134^^,^^135^ Mechanistically, MPHOSPH1 directly interacts with the protein-regulating cytokinesis 1 (PRC1) protein, translocating it along the mitotic spindles during mitosis and promoting cytokinesis.^136^ In line with this, knockdown of MPHOSPH1 induces aberrant cytokinesis, resulting in the formation of multinucleated cells and increased cell death.^136^^,^^137^^,^^138^

### Multifunctional genomic integrity-regulatory CTAS

Several CTAs, discussed below in alphabetical order, are implicated in the regulation of multiple genomic integrity surveillance mechanisms, thus, augmenting the implications of CTA therapeutic intervention.

#### ATAD2/ANCCA/CT137

ATPase Family AAA Domain Containing 2 (ATAD2) is frequently expressed in breast, lung, colorectal, liver, gastric, oral, ovarian, cervical, and endometrial tumors, and correlates with disease severity and poor prognosis. ATAD2 expression can be regulated by the oncogenic E2F transcription factor and in turn binds and activates the MYC transcription factor, thus driving cancer progression.^139^ In colorectal cancer, the oncogenic ubiquitin E3 ligase TRIM25 binds and stabilizes ATAD2 expression following genotoxic stress, which induces a positive ATAD2-E2F-TRIM25 feedback loop whereby ATAD2 acts as a transcriptional co-activator of E2Fs to promote TRIM25 expression.^140^ ATAD2 has been shown to act as a chromatin regulator and to play a role in DNA replication during the S phase by enabling the assembly of histone-modifying protein complexes at target gene chromatin loci.^141^^,^^142^ In addition, Duan et al. reported that ATAD2 is involved in the regulation of the expression and activation of BRCA1, Chk1 and Chk2 in response to DNA damaging anti-cancer drugs, suggesting that ATAD2 is a key mediator of the DNA damage response and repair mechanism.^143^ In accordance, knockdown of ATAD2 sensitizes triple negative breast cancer cells to DNA-damaging agents such as carboplatin^143^ and pancreatic cancer cells to gemcitabine-radiation combination treatment.^144^

#### BAP31/BCAP31

Aberrant expression of B cell receptor Associated Protein 31 (BAP31) can be observed in cervical cancer, colorectal cancer, hepatocellular carcinoma, ovarian cancer, and gastric cancer. In cervical cancer, breast cancer and non-small lung cancer, its expression is positively correlated with poor prognosis, whereas the opposite is true in colorectal cancer and hepatocellular carcinoma. These divergent associations of BAP1 expression with prognosis are likely the result of dual functionality. BAP31 is an endoplasmic reticulum chaperone and regulates apoptosis through its interaction with Bcl and caspase proteins.^145^^,^^146^ It can be cleaved by caspase-8 into a p20 fragment, p20BAP31, which transmits proapoptotic signals between the endoplasmic reticulum and mitochondria,^147^ and increases ROS production,^148^ promoting the induction of DNA damage and cell-cycle arrest. On the other hand, BAP31 was found to regulate the proteasomal degradation of the Cdk inhibitor p27kip1, thereby directly promoting G1/S cell cycle progression.^149^ In line with this finding, depletion of BAP31 has been shown to result in G0/G1 cell-cycle arrest, aberrant cytoskeletal assembly, reduced cancer cell motility, increased apoptosis and reduced tumor progression in xenografts models.^150^^,^^151^^,^^152^^,^^153^^,^^154^ Conversely, BAP31 overexpression is associated with increased tumor cell proliferation, colony formation and tumor growth in *in vitro* and xenograft models respectively.^155^ In cervical cancer, BAP31 expression is regulated by microRNA-362 (miR-362), inhibiting BAP31-mediated activation of the TGFβ/Smad pathway,^156^ which may in part explain the miR-362 associated reduction in cancer cell proliferation and induction of apoptosis.

#### CEP55/URCC6/CT111

Centrosomal Protein 55 (CEP55) is a key regulator of cytokinesis and has been included in a 70-gene chromosomal instability signature associated with aneuploidy in cancer.^157^^,^^158^^,^^159^ Increased CEP55 expression can be found in several cancers including endometrial cancer, bladder cancer, colorectal cancer, liver cancer, non-small cell lung cancer, renal cell carcinoma, cervical cancer, and esophageal squamous cell carcinoma and is generally associated with poor prognosis. Furthermore, overexpression of CEP55 in cervical cancer is associated with increased risk of lymph node metastasis and tumor progression. In breast cancer, CEP55 expression has been linked to docetaxel resistance.^160^ Conversely, knockdown of CEP55 expression reduces breast cancer cell proliferation, and following mitotic arrest by anti-mitotic drugs CEP55 increases cell death while reducing mitotic slippage.^160^^,^^161^ In addition, CEP55 has been shown to promote aberrant mitosis in mouse embryonic fibroblasts through hyperactivation of the PI3K/Akt pathway and a defective S-phase checkpoint, resulting in increased DNA replication, excess DNA damage and microtubule stabilization.^158^

#### LDHC/LDHX/LDH3/CT32

Lactate dehydrogenase C (LDHC) is a metabolic enzyme that plays a critical role in sperm motility, capacitation, and fertilization.^162^^,^^163^^,^^164^ Its expression has been associated with poor prognosis when detected in tumor tissues, cancer patients’ serum and serum-derived exosomes. In few cancers such as head and neck squamous cell carcinoma, and cervical squamous cell carcinoma and endocervical adenocarcinoma LDHC expression is associated with favorable prognosis.^165^ In addition to the full length protein, four splice variants with structural alterations of the catalytic domain have been identified in tumor cells, however, their biological significance has not yet been determined.^166^ LDHC has been shown to promote tumor growth and metastasis of lung adenocarcinomas via activation of the PI3K/Akt/GSK-3β oncogenic-signaling pathway.^18^^,^^167^ We previously demonstrated that LDHC is an immunogenic antigen and plays a role in maintaining genomic stability and mitotic fidelity in breast cancer cell lines, safekeeping tumor cellular fitness.^17^^,^^168^ More specifically, we showed that silencing LDHC results in DNA damage accumulation, microtubule destabilization, mitotic slippage, increased polyploidy and aberrant mitosis, ultimately reducing long-term tumor cell survival. Furthermore, we demonstrated that silencing of LDHC improves sensitivity to treatment with DNA damage response-related drugs.

#### MAGE-C2/HCA587/CT10

Melanoma-Associated Antigen C2 (MAGE-C2) is often expressed in tumors, including seminomas, melanoma, breast tumors, colorectal carcinomas, prostate cancers, non-small cell lung carcinomas, bladder tumors, hepatocellular carcinomas, head and neck squamous carcinomas, squamous cell carcinomas of the larynx, medulloblastomas, gliomas, multiple myelomas, Hodgkin lymphomas, gastrointestinal stromal tumors, salivary gland carcinomas, esophageal squamous cell carcinomas, in particular in those with features of more advanced disease and poor prognosis. Its expression has been associated with chemoresistance in melanoma, as a likely result of enhanced DNA double-strand break repair following heterochromatin relaxation and increased MAGE-C2 mediated phosphorylation of KAP1-Ser824.^169^ Further, MAGE-C2 has been shown to regulate cancer cell proliferation through p53 ubiquitination whereby the E3 ubiquitin ligases TRIM28 and MDM2 compete for binding with MAGE-C2, releasing and activating MDM2 which subsequently ubiquitinates p53.^170^^,^^171^ MAGE-C2 was also found to directly bind the RING domain protein Rbx1 of the E3 ligase Skp1-Cullin1-F box complex, inhibiting cyclin E ubiquitin-dependent degradation and promoting G1-S cell cycle progression.^172^ In multiple myeloma, MAGE-C2 is associated with chemoresistance, promotes double-strand break repair and inhibits p53-dependent apoptosis.^30^^,^^173^

#### OIP5/MIS18B/CT86

Opa interacting protein 5 (OIP5) is a centromere-associated protein that is essential for the recruitment of CENP-A to the site of centromere formation and kinetochore assembly, and as such affects chromosome segregation and maintenance of genomic integrity.^174^ It is expressed across a wide range of solid tumors including ovarian, breast, liver, lung and bladder tumors whereby high OIP5 expression correlates with poor overall and disease-specific survival except for glioblastoma for which conflicting findings have been reported. OIP5 copy number has been shown to correlate with immune cell infiltration in clear-cell renal cell carcinoma.^175^ Furthermore, gene set enrichment analysis of OIP5 low and high expressing tumors identified differential enrichment of genes involved in base excision repair, homologous recombination, DNA replication, cell cycle progression, and the p53 and mismatch repair pathways. OIP5 expression also positively correlates with tumor mutational burden and microsatellite instability in several cancers.^176^ Moreover, OIP5 likely regulates cell cycle progression in a tumor-type specific manner, affecting either G0/G1, G2/M or G1/S transition, leading to apoptosis and senescence.^177^^,^^178^^,^^179^

#### PBK/TOPK/CT84

PDZ-binding kinase (PBK) is a serine threonine kinase capable of phosphorylating and activating several oncogenic signaling pathways.^180^ It is expressed in numerous tumor types including hematological malignancies, bladder cancer, breast cancer, glioma, kidney cancer, prostate cancer, lung adenocarcinoma, hepatocellular carcinoma, cervical cancer, esophageal squamous cell carcinoma, osteosarcoma, medulloblastoma, glioblastoma, adrenocortical carcinoma, colon cancer, ovarian cancer, colorectal carcinoma, and gastric cancer. Overall, high PBK expression is associated with poor prognosis, except in patients with colon cancer, cholangiocarcinoma, and oral squamous cell carcinoma. Similarly to OIP5, PBK expression correlates with features of genomic instability such as high tumor mutational burden and microsatellite instability.^181^ PBK has been shown to regulate genomic instability through its involvement in multiple cell cycle checkpoints. It is best known as a mitotic kinase that mediates G2/M progression, dissociation of proteins from condensed chromatin, chromosome segregation, spindle formation and cytokinesis.^182^^,^^183^ In addition, PBK downregulates p53 signaling through either direct interaction, inhibiting transactivation of p53 target genes, or through phosphorylation of histone H3 and subsequent reduction in p53 expression.^182^^,^^184^ A recent study demonstrated that PBK also plays a role in G1/S transition through phospho-activation of Chk1 and Cdc25C in response to replication stress, and that deletion of PBK renders cancer cells vulnerable to radiation-induced DNA damage.^185^^,^^186^ Furthermore, inhibition of PBK enhanced treatment response to olaparib and cisplatin in ovarian cancer,^187^^,^^188^ cisplatin in cervical cancer^189^ and paclitaxel in non-small cell lung cancer.^190^ Mechanistically, inhibition of PBK was found to induce G1 cell-cycle arrest through activation of the PI3K-Akt-IKK signaling pathway, suppress cell survival signals while promoting pro-apoptosis signaling, and inhibit cancer cell migration and invasion.^191^^,^^192^

#### SSX2/HOM-MEL-40/CT5.2a

Synovial Sarcoma, X-breakpoint 2 (SSX2) belongs to a family of ten homologous members that function as transcription repressors and are widely expressed in tumors. Expression analysis showed aberrant expression of SSX2 in various cancers including melanoma, colon cancer, hepatocellular carcinoma, and breast cancer. SSX2 expression has been associated with advanced disease and worse prognosis and can induce humoral and cellular immune responses in cancer patients. Gene rearrangements can lead to SSX2 fusion genes such as SS18-SSX2, a main oncogenic driver and diagnostic marker in synovial sarcoma, and the more recently identified EWSR1-SSX2 fusion gene in a patient with undifferentiated sarcoma of the bone.^193^^,^^194^^,^^195^ The tumorigenic role and prognostic value of SSX2 remain elusive. In melanoma, ectopic expression of SSX2 promotes genomic instability through the induction of replication defects and p53-mediated G1 cell-cycle arrest.^31^^,^^196^ Furthermore, SSX2 reduces the stability of Polycomb-group (PcG) repressive complexes at the chromosome 1q12 pericentromeric heterochromatin structure, a site of frequent genetic aberrations in cancer, promoting the de-repression of 1q12 heterochromatin and resulting in increased genomic instability due to segregation abnormalities and generation of micronuclei.^197^ In contrast, in MCF7 breast cancer cells, the cell line’s molecular landscape likely supports the maintenance of genomic integrity upon activation of the p53-p21 cell cycle checkpoint and drives SSX2-expressing cancer cells toward senescence through the Mediator complex.^198^ These findings highlight the need for further investigation to discern the tissue type-dependent factors that determine the SSX2-associated cell fates in different cancers.

#### TEX12

Testis-expressed protein 12 (TEX12), like SYCP3, is a component of the synaptonemal complex which localizes to the centrosomes during meiosis and mitosis.^199^ TEX12 expression in cancer cells has been associated with centrosome amplification, a process that is closely linked to oncogenesis and poor prognosis.^199^ Analysis of a large-scale transcriptomic dataset revealed aberrant expression of TEX12 in breast, ovarian, stomach, liver, glioblastoma and myeloid leukemia cancer cells where it was associated with more aggressive tumors. Furthermore, TEX12 has been identified as one of five DNA damage repair genes that form a prognostic signature that can predict the overall survival and response to immunotherapy of cervical squamous cell carcinoma patients.^200^

## Clinical trials targeting genomic integrity-regulatory CTAS

**Figure 2:**
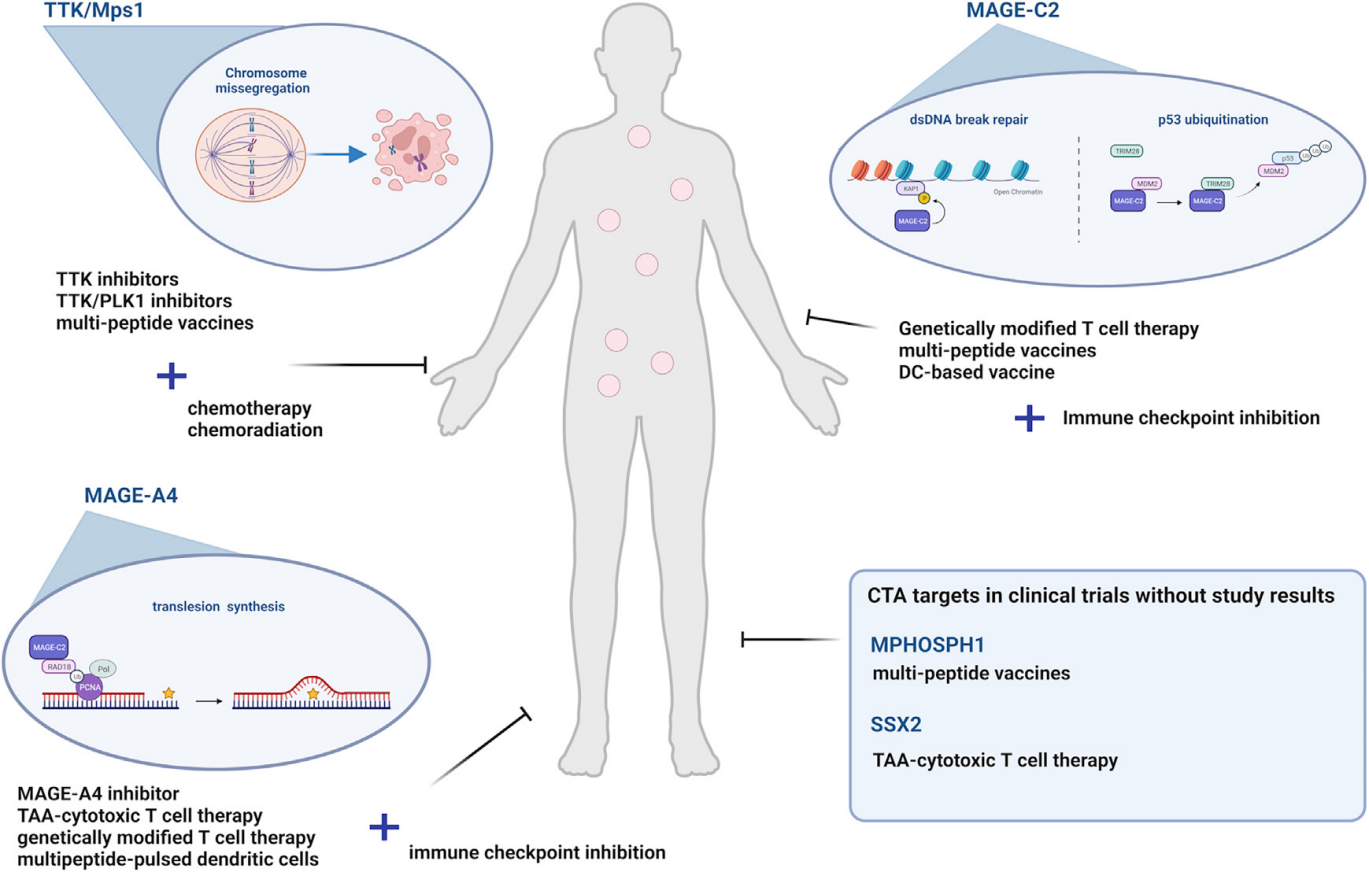
Therapeutic potential of targeting CTAs involved in regulating genomic integrity in cancer The clinical value of targeting specific CTAs that play key roles in regulating tumor genomic integrity is currently studied in phase I and phase II clinical trials. To date, the majority of clinical studies focus on targeting TTK/Mps1, MAGE-A4 and MAGE-C2 with few trials investigating the safety and anti-tumor activity of targeting MPHOSPH1 and SSX2. DC, dendritic cell; TAA, tumor associated antigen.

The discovery of cancer testis antigens as highly tumor-specific antigens with important roles in cancer hallmarks opens up new avenues for cancer cell-specific targeting. Historically, CTAs have been identified through autologous typing of T cell clones and serological analysis of cDNA libraries derived from cancer patients, and since several CTAs have been reported to exhibit immunogenic properties which could be exploited in immune-based interventions.^201^^,^^202^ Hence, various studies and clinical trials have focused on the potential use of CTA-targeted antibodies, vaccines, and adoptive cell therapy approaches to elicit potent, durable anti-tumor responses. Most notably, NY-ESO-1, MAGE-A3 and PRAME have been extensively studied as prime targets for immunotherapy as reviewed in detail elsewhere.^4^^,^^203^^,^^204^ Likewise, efforts to target genomic integrity-regulatory CTAs have been directed toward the development of immunotherapeutic strategies in addition to small molecule inhibitors (Figure 2).

### Immunotherapy

As immunotherapy has emerged at the forefront in cancer treatment, several immunotherapeutic approaches targeting a select few genomic integrity-regulatory CTAs are currently in clinical trial. In particular, Mps1/TTK has been investigated as candidate target using multi-peptide cancer vaccines (NCT00681330, NCT00676949, NCT00674258). Notably, treatment of patients with advanced esophageal cancer with a multi-CTA vaccine against TTK, LY6K and IMP3 (NCT00682227) induced specific T cell immunity and resulted in clinical responses in 50% of patients.^205^ Another trial in patients with metastatic esophageal squamous cell carcinoma (NCT00669292) revealed peptide-specific cytotoxic T cell responses and stable disease response rates of 67% after treatment with a TTK and LY6K peptide vaccine in combination with the immunostimulatory TLR9 agonist CpG-7907.^206^ Vaccination of patients with advanced or recurrent non-small cell lung cancer with a multi-peptide vaccine targeting TTK, Ly6K, VEGFR1, and VEGFR2 (NCT00633724) induced strong specific T cell responses and stable disease in 47% of patients.^207^ To date, one phase I clinical trial has been conducted to study the effect of multi-peptide vaccination (TTK, URLC10, KOC1, VEGFR1, VEGFR2) in combination with chemoradiation therapy in esophageal cancer (NCT00632333), demonstrating peptide-specific cytotoxic T cell immune responses and durable complete responses in 54.5% of patients.^208^ In analogy with TTK, the cancer testis antigen MPHOSPH1 is being targeted as part of multi-peptide vaccinations to treat metastatic breast cancer (NCT01259505), bladder cancer (NCT00633204, NCT00635336), unresectable recurrent and/or metastatic solid tumors (NCT04316689), and cervical, gastro-intestinal and lung tumors (NCT00676949); however, no data has been published as yet. In turn, targeting of SSX2 is being explored using multi tumor-associated antigen (TAA)-specific cytotoxic T lymphocytes in patients with breast cancer (TACTIC study, NCT03093350), pancreatic cancer (TACTOPS study, NCT03192462) and lymphoma (NCT01333046).^209^ Likewise, various immune-based strategies are currently explored to target MAGE-C2 in solid cancer, including autologous MAGE-C2 engineered T cells (NCT04729543) and multi-peptide vaccines in combination with PD-L1 immune checkpoint inhibition (NCT03164772). Results from a phase 2 study in advanced melanoma demonstrated that dendritic cell-based mRNA and tumor antigen vaccination combined with CTLA-4 blockade (NCT01302496) induced multi-antigen, polyfunctional CD8+ T cell responses in 80% of patients.^210^ Another MAGE family member, MAGE-A4, is extensively evaluated as a novel immunotherapy approach to treat patients with a variety of cancers with TAA-specific cytotoxic T lymphocytes (NCT01333046)^209^ or genetically modified cytotoxic T cells (NCT01694472, NCT03132922, NCT04044859, NCT05601752, NCT03973333, NCT02096614, NCT04752358, NCT04044768, NCT04408898, NCT03247309, NCT03356808, NCT03132922 and NCT04044768).^211^^,^^212^^,^^213^ Specifically, treatment with autologous T cells with an affinity-optimized T cell receptor against MAGE-A4 (afamitresgene autoleucel or afami-cel) resulted in an overall response rate of 24% in patients with 9 different relapsed/refractory metastatic solid tumor types, with the highest clinical activity in synovial sarcoma (overall response rate of 44%).^212^^,^^214^ Preliminary peripheral and tumor analyses revealed the presence of MAGE-A4 engineered T cell immune cells in the circulation up to 18 months after treatment, elevated levels of IFN-γ up to 12 days after T cell infusion, and intra-tumoral T cell infiltration. In addition, the combination of autologous dendritic cells pulsed with multi-TAA peptides, including MAGE-A4, and the PD-1 immune checkpoint inhibitor nivolumab is in phase II trial in advanced non-small cell lung cancer (NCT04199559). Preclinical research further suggests that the addition of the CD8α receptor to an affinity-enhanced HLA class I-restricted TCR against MAGE-A4 can enhance CD4+ T helper and effector functions which could improve the depth and durability of anti-tumor immune responses.^215^

### Small molecule inhibitors

In contrast to the number of available immunotherapy clinical trials, sparse information is available on clinical trials of targeted therapy against the twenty-eight aforementioned CTAs. More specifically, a few studies have investigated the safety and efficacy of small molecule inhibitors against MAGE-A4 and Mps1/TTK. The MAGE-A4 inhibitor RO7444973 (NCT05129280) is currently under safety, pharmacokinetics, pharmacodynamics, and preliminary efficacy assessment in patients with unresectable and/or metastatic solid tumors.^216^ To date, four small molecule inhibitors targeting Mps1/TTK (BAY-1217389, CFI-402257, BOS-172722, and S-81694) are being investigated as single agents or in combination with chemotherapeutic drugs. Treatment with BAY-1217389 in combination with paclitaxel (NCT02366949) demonstrated partial responses in 31.6% of the patients; however, these were associated with considerable toxicity, limiting the therapeutic window.^90^ The safety profile and pharmacokinetics of CFI-402257 are under evaluation in solid tumors as either monotherapy, or in combination with hormonal therapy or paclitaxel (NCT02792465, NCT05251714, NCT03568422). The safety, maximum tolerated dose, and anti-tumor activity of BOS-172722 (NCT03328494) and S-81694 (NCT03411161) alone or in combination with paclitaxel are under study in patients with advanced non-hematologic malignancies and metastatic breast cancer. Furthermore, a phase I clinical trial is currently recruiting patients with advanced solid tumors to assess the safety and tolerability of dual inhibition of TTK and PLK1 by BAL0891 with or without carboplatin or paclitaxel (NCT05768932). In addition, preclinical studies have reported that TTK inhibition in combination with radiotherapy enhances mitotic catastrophe and impaired DNA damage repair, suggesting that the combination treatment may result in synergistic effects.^217^

## Challenges of CTA-targeted therapy

Traditionally, cancer care comprises of surgery and systemic treatment with chemotherapy and/or radiotherapy. A deeper understanding of molecular differences between breast tumors in the last decades has led to the development of targeted approaches, tailored to interact with specific molecules expressed by the cancer cells. More recently, there has been a surge in immunotherapy clinical trials, demonstrating promising anti-tumor activities in a range of cancers. As such, the development of CTA-based treatment has been focused on cancer vaccines and adoptive cell therapy. However, the benefits in solid cancers have been limited so far, which may be attributed to tumor-intrinsic and extrinsic factors that also play a role in the efficacy of other treatment modalities. Firstly, tumor heterogeneity greatly impacts cancer treatment outcomes. Tumor cells can dysregulate the expression and function of the antigen processing and presentation machinery, thereby impairing the cell surface expression of intracellular CTAs and resulting in tumor cell subpopulations with reduced target expression within a single tumor. In addition, patients may develop acquired resistance through antigen escape whereby CTA-based treatment result in a positive selective pressure toward CTA-negative tumor cell subclones. Aberrant expression of genomic integrity-regulatory CTAs could also directly promote tumor heterogeneity as increased genomic instability enables the acquisition of other cancer hallmarks, giving rise to genetically distinct subpopulations which may be more resistant to combination therapy. Secondly, the tumor microenvironment has a profound effect on the efficacy of cancer treatment. Tumor-derived and microenvironmental cues together shape the tumor immune microenvironment as either immune favorable or unfavorable. Increased genomic instability, conferred by the aberrant tumor expression of genomic integrity-regulatory CTAs, can impede anti-tumor immune responses as the higher tumor cell diversity may facilitate the adoption of immune escape mechanisms. In addition, tumor cells exhibit high rates of aerobic glycolysis and extracellular lactic acidosis, dampening the anti-tumor activity of cytotoxic T cells. Furthermore, we demonstrated that expression of PRAME is associated with increased immune checkpoint expression and reduced T cell functionality.^218^ Likewise, expression of CEP55 has been positively correlated with the expression of more than 30 immune checkpoint genes in a wide range of solid tumors, as well as enhanced infiltration of immunosuppressive myeloid-derived suppressor cells and Th2 cells, expression of immunomodulators and response to immune checkpoint blockade.^219^^,^^220^^,^^221^ Furthermore, high expression of PBK has been associated with immune escape due to upregulation of PD-L1 expression, dysregulation of antigen presentation and immune cell infiltration.^181^^,^^222^^,^^223^^,^^224^^,^^225^^,^^226^ Of note, a subcluster of MAGE-A antigens has been reported to predict treatment response of melanoma tumors to immune checkpoint blockade.^227^^,^^228^

We would like to highlight some precautions that should be considered when developing novel CTA-based treatments. We believe that future development of CTA-based cancer care should include a careful comprehensive mapping of CTA expression in normal and tumor tissues along with an accurate annotation of CTAs as testis-restricted or testis-selective antigens in order to advance personalized cancer treatment with favorable safety profiles. The significance of such efforts becomes apparent when considering PBK inhibition, which exhibits remarkable anti-tumor activity in pre-clinical models, but at the same time targets a protein that is crucial for neuronal self-renewal and protection against ischemic postconditioning in the heart and brain.^182^ Lastly, it is important to consider the potential adverse effects of CTA-based therapy on male fertility due to the diverse roles of CTAs in spermatogenesis which may be compromised by the uptake of CTA-targeting drugs across the blood-testis-barrier by drug transporters in Sertoli cells of the testis.

## Future therapeutic perspectives for genomic integrity-regulatory CTAS

With only a handful of genomic integrity-regulatory CTAs currently being evaluated in clinical trials, there is ample room for the development of novel therapeutic opportunities targeting other members of this subset of CTAs. Based on the growing evidence from pre-clinical reports, there are additional less-studied CTAs that could be targeted to leverage genomic instability and improve treatment response to existing treatment regimens including chemotherapy and radiotherapy. Several naturally occurring compounds have been identified as inhibitors of CEP55, MPHOSPH1, and PRDM9 and are undergoing pre-clinical testing,^229^^,^^230^^,^^231^ Inhibitors targeting PBK/TOPK have been shown to suppress tumor growth and metastasis in cancer xenograft models,^192^^,^^232^^,^^233^ and to sensitize tumor cells to anti-cancer drugs such as olaparib^187^ and lenalidomide.^234^^,^^235^ Further, depletion of BORIS,^236^^,^^237^ HORMAD1,^54^ OIP5^238^^,^^239^ and CT45^240^ results in increased sensitivity of tumor cells to cisplatin and docetaxel treatment in different cancer cell line models. In contrast, the presence of MEIOB was shown to sensitize triple negative breast cancer cells to PARP inhibitors *in vitro* and in patient-derived xenograft models, suggesting that MEIOB expression could be used as a predictive biomarker for treatment response to PARP inhibition.^58^ Furthermore, targeting genomic integrity-regulatory CTAs could also be used to promote radiosensitivity. For instance, PBK inhibition by OTS964 has been shown to sensitize ovarian cancer cells to radiotherapy due to increased fork stalling and collapse in response to radiation-induced replication stress and DNA damage, highlighting the potential of combination treatment.^185^ In line with this finding, upregulation of miR-372 enhanced the radiosensitivity of nasopharyngeal carcinoma cells by downregulating PKB and subsequently activating the p53 signaling pathway, leading to increased cell death and cell-cycle arrest in response to radiation-induced DNA damage.^241^

Furthermore, preclinical studies are exploring alternative therapeutic approaches including the use of miRNA-based therapy,^241^ CTA-specific antibodies^155^ and CRISPR/Cas9 gene editing,^240^ which will require further validation in clinical studies. Finally, the aforementioned challenges that impact the therapeutic potential of targeting CTAs could be exploited to improve treatment responses. For instance, CTAs could be targeted in combination with metabolic therapy or drugs to upregulate the antigen presentation machinery.

## Conclusions

In normal physiological conditions, CTAs play critical roles during meiosis by regulating DNA damage repair and maintaining genomic integrity, processes which are also intricately linked to tumor cellular fitness. The highly tumor specific expression of CTAs in conjunction with these pro-tumorigenic functions make them lucrative anti-cancer targets to directly impair tumor cell survival or to enhance treatment responses to drugs that negatively impact genomic stability. As such, CTAs are increasingly being studied in phase I and II clinical trials using specific inhibitors or CTA-specific immunotherapy approaches, either as monotherapy or combination therapy. Initial study results reveal promising anti-tumor activities with durable cellular immune responses in multiple solid tumors, underscoring the need for systematic analysis of CTA expression and functionality in cancer.

## Supplemental information

Document S1. Supplemental references, related to Table S1

Table S1. Expression and prognostic connotation of genomic integrity-regulatory CTAs, related to Figure 1

Document S2. Article plus supplemental information
