# Supplementary material for: Cancer testis antigens: Emerging therapeutic targets leveraging genomic instability in cancer
Source: Mol Ther Oncol. 2024 Jan 26;32(1):200768. doi: 10.1016/j.omton.2024.200768 (PMC10876628; doi:10.1016/j.omton.2024.200768)
Supplement: Document S1. Supplemental references, related to Table S1 [file mmc1.pdf]

OMTON, Volume *32*

Supplemental information

Cancer testis antigens: Emerging therapeutic targets leveraging genomic instability in cancer

Adviti Naik, Boucif Lattab, Hanan Qasem, and Julie Decock

Supplemental material

Supplemental References, related to Table S1.

[S1] Fan, R., Huang, W., Luo, B., Zhang, Q.M., Xiao, S.W., and Xie, X.X. (2015). Cancer testis antigen OY-TES-1: analysis of protein expression in ovarian cancer with tissue microarrays. Eur J Gynaecol Oncol 36, 298–303.

[S2] Fu, J., Luo, B., Guo, W.W., Zhang, Q.M., Shi, L., Hu, Q.P., Chen, F., Xiao, S.W., and Xie, X.X. (2015). Down-regulation of cancer/testis antigen OY-TES-1 attenuates malignant behaviors of hepatocellular carcinoma cells in vitro. Int J Clin Exp Pathol 8, 7786–7797.

[S3] Li, X., Yan, J., Fan, R., Luo, B., Zhang, Q., Lin, Y., Zhou, S., Luo, G., Xie, X., and Xiao, S. (2017). Serum immunoreactivity of cancer/testis antigen OY-TES-1 and its tissues expression in glioma. Oncol Lett 13, 3080–3086. 10.3892/ol.2017.5799.

[S4] Luo, B., Yun, X., Fan, R., Lin, Y.-D., He, S.-J., Zhang, Q.-M., Mo, F.-R., Chen, F., Xiao, S.-W., and Xie, X.-X. (2013). Cancer testis antigen OY-TES-1 expression and serum immunogenicity in colorectal cancer: its relationship to clinicopathological parameters. Int J Clin Exp Pathol 6, 2835–2845.

[S5] Luo, B., Yun, X., Li, J., Fan, R., Guo, W.-W., Liu, C., Lin, Y., Ge, Y.-Y., Zeng, X., Bi, S.-Q., et al. (2020). Cancer-testis Antigen OY-TES-1 Expression and Immunogenicity in Hepatocellular Carcinoma. Curr Med Sci 40, 719–728. 10.1007/s11596-020-2241-x.

[S6] Ono, T., Kurashige, T., Harada, N., Noguchi, Y., Saika, T., Niikawa, N., Aoe, M., Nakamura, S., Higashi, T., Hiraki, A., et al. (2001). Identification of proacrosin binding protein sp32 precursor as a human cancer/testis antigen. Proc Natl Acad Sci U S A 98, 3282–3287. 10.1073/pnas.041625098.

[S7] Whitehurst, A.W., Xie, Y., Purinton, S.C., Cappell, K.M., Swanik, J.T., Larson, B., Girard, L., Schorge, J.O., and White, M.A. (2010). Tumor antigen acrosin binding protein normalizes mitotic spindle function to promote cancer cell proliferation. Cancer Res 70, 7652–7661. 10.1158/0008-5472.CAN-10-0840.

[S8] Ciro, M., Prosperini, E., Quarto, M., Grazini, U., Walfridsson, J., McBlane, F., Nucifero, P., Pacchiana, G., Capra, M., Christensen, J., et al. (2009). ATAD2 is a novel cofactor for MYC, overexpressed and amplified in aggressive tumors. Cancer Res 69, 8491–8498. 10.1158/0008-5472.CAN-09-2131.

[S9] Duan, Z., Andrews, N.P., Chen, C.Z., Fan, M., Wang, J., Shen, J., Li, J.J., and Chen, H.W. (2020). Targeting bromodomain protein ANCCA/ATAD2 enhances the efficacy of DNA-damaging chemotherapy agents and radiation. Oncol Rep 43, 318–327. 10.3892/or.2019.7418.

[S10] Dutta, M., Mohapatra, D., Mohapatra, A.P., Senapati, S., and Roychowdhury, A. (2022). ATAD2 suppression enhances the combinatorial effect of gemcitabine and radiation in pancreatic cancer cells. Biochem Biophys Res Commun 635, 179–186. 10.1016/j.bbrc.2022.10.021.

[S11] Hou, M., Huang, R., Song, Y., Feng, D., Jiang, Y., and Liu, M. (2016). ATAD2 overexpression is associated with progression and prognosis in colorectal cancer. Jpn J Clin Oncol 46, 222–227. 10.1093/jjco/hyv195.

[S12] Hwang, H.W., Ha, S.Y., Bang, H., and Park, C.-K. (2015). ATAD2 as a Poor Prognostic Marker for Hepatocellular Carcinoma after Curative Resection. Cancer Res Treat 47, 853–861. 10.4143/crt.2014.177.

[S13] Kalashnikova, E.V., Revenko, A.S., Gemo, A.T., Andrews, N.P., Tepper, C.G., Zou, J.X., Cardiff, R.D., Borowsky, A.D., and Chen, H.-W. (2010). ANCCA/ATAD2 overexpression identifies breast cancer patients with poor prognosis, acting to drive proliferation and survival of triple-negative cells through control of B-Myb and EZH2. Cancer Res 70, 9402–9412. 10.1158/0008-5472.CAN-10-1199.

[S14] Koo, S.J., Fernandez-Montalvan, A.E., Badock, V., Ott, C.J., Holton, S.J., von Ahsen, O., Toedling, J., Vittori, S., Bradner, J.E., and Gorjanacz, M. (2016). ATAD2 is an epigenetic reader of newly synthesized histone marks during DNA replication. Oncotarget 7, 70323–70335. 10.18632/oncotarget.11855.

[S15] Liu, Q., Liu, H., Li, L., Dong, X., Ru, X., Fan, X., Wen, T., and Liu, J. (2020). ATAD2 predicts poor outcomes in patients with ovarian cancer and is a marker of proliferation. Int J Oncol 56, 219–231. 10.3892/ijo.2019.4913.

[S16] Liu, X., Zhou, Y., Liu, X., Peng, A., Gong, H., Huang, L., Ji, K., Petersen, R.B., Zheng, L., and Huang, K. (2014). MPHOSPH1: a potential therapeutic target for hepatocellular carcinoma. Cancer Res 74, 6623–6634. 10.1158/0008-5472.CAN-14-1279.

[S17] Revenko, A.S., Kalashnikova, E.V., Gemo, A.T., Zou, J.X., and Chen, H.-W. (2010). Chromatin loading of E2F-MLL complex by cancer-associated coregulator ANCCA via reading a specific histone mark. Mol Cell Biol 30, 5260–5272. 10.1128/MCB.00484-10.

[S18] Shang, P., Meng, F., Liu, Y., and Chen, X. (2015). Overexpression of ANCCA/ATAD2 in endometrial carcinoma and its correlation with tumor progression and poor prognosis. Tumour Biol 36, 4479–4485. 10.1007/s13277-015-3089-8.

[S19] Tong, Y., Li, J., Peng, M., Qian, Q., Shi, W., Chen, Z., and Liu, B. (2022). ATAD2 drives colorectal cancer progression by regulating TRIM25 expression via a positive feedback loop with E2F transcriptional factors. Biochem Biophys Res Commun 594, 146–152. 10.1016/j.bbrc.2022.01.036.

[S20] Wang, D., Pan, Y., Hao, T., Chen, Y., Qiu, S., Chen, L., and Zhao, J. (2016). Clinical and Prognostic Significance of ANCCA in Squamous Cell Lung Carcinoma Patients. Arch Med Res 47, 89–95. 10.1016/j.arcmed.2016.04.001.

[S21] Zhang, M.-J., Zhang, C.-Z., Du, W.-J., Yang, X.-Z., and Chen, Z.-P. (2016). ATAD2 is overexpressed in gastric cancer and serves as an independent poor prognostic biomarker. Clin Transl Oncol 18, 776–781. 10.1007/s12094-015-1430-8.

[S22] Zhang, Y., Sun, Y., Li, Y., Fang, Z., Wang, R., Pan, Y., Hu, H., Luo, X., Ye, T., Li, H., et al. (2013). ANCCA protein expression is a novel independent poor prognostic marker in surgically resected lung adenocarcinoma. Ann Surg Oncol 20 Suppl 3, S577-582. 10.1245/s10434-013-3027-1.

[S23] Zheng, L., Li, T., Zhang, Y., Guo, Y., Yao, J., Dou, L., and Guo, K. (2015). Oncogene ATAD2 promotes cell proliferation, invasion and migration in cervical cancer. Oncol Rep 33, 2337–2344. 10.3892/or.2015.3867.

[S24] Chandra, D., Choy, G., Deng, X., Bhatia, B., Daniel, P., and Tang, D.G. (2004). Association of active caspase 8 with the mitochondrial membrane during apoptosis: potential roles in cleaving BAP31 and caspase 3 and mediating mitochondrion-endoplasmic reticulum cross talk in etoposide-induced cell death. Mol Cell Biol 24, 6592–6607. 10.1128/MCB.24.15.6592-6607.2004.

[S25] Chen, J., Guo, H., Jiang, H., Namusamba, M., Wang, C., Lan, T., Wang, T., and Wang, B. (2019). A BAP31 intrabody induces gastric cancer cell death by inhibiting p27(kip1) proteasome degradation. Int J Cancer 144, 2051–2062. 10.1002/ijc.31930.

[S26] Grimm, S. (2012). The ER-mitochondria interface: the social network of cell death. Biochim Biophys Acta 1823, 327–334. 10.1016/j.bbamcr.2011.11.018.

[S27] Jiang, X., Li, G., Zhu, B., Zang, J., Lan, T., Jiang, R., and Wang, B. (2023). p20BAP31 induces cell apoptosis via both AIF caspase-independent and the ROS/JNK mitochondrial pathway in colorectal cancer. Cell Mol Biol Lett 28, 25. 10.1186/s11658-023-00434-z.

[S28] Li, T., Hao, Z., Tang, Z., Li, C., Cheng, L., Wang, T., Zhu, X., He, Y., Huang, Y., and Wang, B. (2022). BAP31 Regulates Wnt Signaling to Modulate Cell Migration in Lung Cancer. Front Oncol 12, 859195. 10.3389/fonc.2022.859195.

[S29] Liang, H., Dong, J., Cheng, Z., Li, Q., Feng, D., and Ling, B. (2021). B-cell receptor-associated protein 31 promotes migration and invasion in ovarian cancer cells. Exp Ther Med 22, 858. 10.3892/etm.2021.10290.

[S30] Liu, T., Yu, J., Ge, C., Zhao, F., Miao, C., Jin, W., Su, Y., Geng, Q., Chen, T., Xie, H., et al. (2021). B-Cell Receptor-Associated Protein 31 Promotes Metastasis via AKT/beta-Catenin/Snail Pathway in Hepatocellular Carcinoma. Front Mol Biosci 8, 656151. 10.3389/fmolb.2021.656151.

[S31] Ma, C., Jin, R.M., Chen, K.J., Hao, T., Li, B.S., Zhao, D.H., and Jiang, H. (2018). Low expression of B-Cell-Associated protein 31 is associated with unfavorable prognosis in human colorectal cancer. Pathol Res Pract 214, 661–666. 10.1016/j.prp.2018.03.023.

[S32] Quistgaard, E.M. (2021). BAP31: Physiological functions and roles in disease. Biochimie 186, 105–129. 10.1016/j.biochi.2021.04.008.

[S33] Sun, M., Liu, X., Wei, W., Ge, N., Luo, S., Shen, S., and Ge, R. (2022). BAP31 Promotes Proliferation, Invasion, and Metastasis of Liver Cancer Cells via Activating PI3K/AKT Pathway. J Healthc Eng 2022, 7686728. 10.1155/2022/7686728.

[S34] Tan, N., Liu, Q., Liu, X., Gong, Z., Zeng, Y., Pan, G., Xu, Q., and He, S. (2016). Low expression of B-cell-associated protein 31 in human primary hepatocellular carcinoma correlates with poor prognosis. Histopathology 68, 221–229. 10.1111/his.12738.

[S35] Wang, A., Zhang, Y., and Cao, P. (2019). Inhibition of BAP31 expression inhibits cervical cancer progression by suppressing metastasis and inducing intrinsic and extrinsic apoptosis. Biochem Biophys Res Commun 508, 499–506. 10.1016/j.bbrc.2018.11.017.

[S36] Wang, B., Pelletier, J., Massaad, M.J., Herscovics, A., and Shore, G.C. (2004). The yeast split-ubiquitin membrane protein two-hybrid screen identifies BAP31 as a regulator of the turnover of endoplasmic reticulum-associated protein tyrosine phosphatase-like B. Mol Cell Biol 24, 2767–2778. 10.1128/MCB.24.7.2767-2778.2004.

[S37] Xu, K., Han, B., Bai, Y., Ma, X.Y., Ji, Z.N., Xiong, Y., Miao, S.K., Zhang, Y.Y., and Zhou, L.M. (2019). MiR-451a suppressing BAP31 can inhibit proliferation and increase apoptosis through inducing ER stress in colorectal cancer. Cell Death Dis 10, 152. 10.1038/s41419-019-1403-x.

[S38] Yang, S., Sun, Y., Jiang, D., Wang, J., Dang, E., Li, Z., Zhou, J., Lu, Y., Shi, J., Tao, L., et al. (2021). MiR-362 suppresses cervical cancer progression via directly targeting BAP31 and activating TGFbeta/Smad pathway. Cancer Med 10, 305–316. 10.1002/cam4.3601.

[S39] Zhang, X., Jiang, D., Yang, S., Sun, Y., Liu, Y., Shi, J., Hu, C., Pan, J., Liu, T., Jin, B., et al. (2020). BAP31 Promotes Tumor Cell Proliferation by Stabilizing SERPINE2 in Hepatocellular Carcinoma. Front Cell Dev Biol 8, 607906. 10.3389/fcell.2020.607906.

[S40] Dougherty, C.J., Ichim, T.E., Liu, L., Reznik, G., Min, W.-P., Ghochikyan, A., Agadjanyan, M.G., and Reznik, B.N. (2008). Selective apoptosis of breast cancer cells by siRNA targeting of BORIS. Biochem Biophys Res Commun 370, 109–112. 10.1016/j.bbrc.2008.03.040.

[S41] He, J., Huang, Y., Liu, Z., Zhao, R., Liu, Q., Wei, L., Yu, X., Li, B., and Qin, Y. (2017). Hypomethylation of BORIS is a promising prognostic biomarker in hepatocellular carcinoma. Gene 629, 29–34. 10.1016/j.gene.2017.07.077.

[S42] Martin-Kleiner, I. (2012). BORIS in human cancers -- a review. Eur J Cancer 48, 929–935. 10.1016/j.ejca.2011.09.009.

[S43] Okabayashi, K., Fujita, T., Miyazaki, J., Okada, T., Iwata, T., Hirao, N., Noji, S., Tsukamoto, N., Goshima, N., Hasegawa, H., et al. (2012). Cancer-testis antigen BORIS is a novel prognostic marker for patients with esophageal cancer. Cancer Sci 103, 1617–1624. 10.1111/j.1349-7006.2012.02355.x.

[S44] Zhang, Y., Fang, M., Song, Y., Ren, J., Fang, J., and Wang, X. (2017). Brother of Regulator of Imprinted Sites (BORIS) suppresses apoptosis in colorectal cancer. Sci Rep 7, 40786. 10.1038/srep40786.

[S45] Zhang, Y., Song, Y., Li, C., Ren, J., Fang, M., Fang, J., and Wang, X. (2020). Brother of regulator of imprinted sites inhibits cisplatin-induced DNA damage in non-small cell lung cancer. Oncol Lett 20, 251. 10.3892/ol.2020.12114.

[S46] Zuo, B., Yao, W., Fang, M., Ren, J., Tu, L., Fan, R., and Zhang, Y. (2023). Boris knockout eliminates AOM/DSS-induced in situ colorectal cancer by suppressing DNA damage repair and inflammation. Cancer Sci 114, 1972–1985. 10.1111/cas.15732.

[S47] Bogdanov, K.V., Merzlikina, O.V., Mirolyubova, Y.V., Girshova, L.L., Lomaia, E.G., and Zaritskey, A.Y. (2021). CASC5 Gene Expression Changes Correlate with Targeted Mutations in Leukemia. Molecular Biology 55, 121–132. 10.1134/S0026893321010027.

[S48] Cheeseman, I.M., Hori, T., Fukagawa, T., and Desai, A. (2008). KNL1 and the CENP-H/I/K complex coordinately direct kinetochore assembly in vertebrates. Mol Biol Cell 19, 587–594. 10.1091/mbc.e07-10-1051.

[S49] Cui, Y., Zhang, C., Ma, S., Guo, W., Cao, W., and Guan, F. (2020). CASC5 is a potential tumour driving gene in lung adenocarcinoma. Cell Biochem Funct 38, 733–742. 10.1002/cbf.3540.

[S50] Rosenberg, J.S., Cross, F.R., and Funabiki, H. (2011). KNL1/Spc105 recruits PP1 to silence the spindle assembly checkpoint. Curr Biol 21, 942–947. 10.1016/j.cub.2011.04.011.

[S51] Singh, P.K., Bhatt, M.L.B., Singh, P., Rath, S.K., Dalela, D., and Goel, M.M. (2021). CASC5 is a potential cancer-testis gene in human urinary bladder transitional cell carcinoma. Drug Discov Ther 15, 331–336. 10.5582/ddt.2021.01108.

[S52] Lee, S.N., Hong, K.M., Seong, Y.S., and Kwak, S.J. (2020). Ectopic Overexpression of Coiled-Coil Domain Containing 110 Delays G2/M Entry in U2-OS Cells. Dev Reprod 24, 101–111. 10.12717/DR.2020.24.2.101.

[S53] Monji, M., Nakatsura, T., Senju, S., Yoshitake, Y., Sawatsubashi, M., Shinohara, M., Kageshita, T., Ono, T., Inokuchi, A., and Nishimura, Y. (2004). Identification of a novel human cancer/testis

antigen, KM-HN-1, recognized by cellular and humoral immune responses. Clin Cancer Res 10, 6047–6057. 10.1158/1078-0432.CCR-04-0475.
[S54] Park, H.J., Seo, H.J., Kim, H.W., Kim, J.S., Hwang, S.Y., and Seong, Y.S. (2007). The centrosomal localization of KM-HN-1 (MGC33607) depends on the leucine zipper motif and the C-terminal coiled-coil domain. Exp Mol Med 39, 828–838. 10.1038/emm.2007.90.
[S55] Du, T., Yi, S., Wang, Y., Zhao, Q., Ma, P., and Jiang, W. (2022). Circular RNA_0120376 regulates microRNA-148b-3 and centrosomal protein 55 to promote non-small cell lung cancer development. Bioengineered 13, 11844–11855. 10.1080/21655979.2022.2052647.
[S56] Jeffery, J., Sinha, D., Srihari, S., Kalimutho, M., and Khanna, K.K. (2016). Beyond cytokinesis: the emerging roles of CEP55 in tumorigenesis. Oncogene 35, 683–690. 10.1038/onc.2015.128.
[S57] Jiang, W., Wang, Z., and Jia, Y. (2017). CEP55 overexpression predicts poor prognosis in patients with locally advanced esophageal squamous cell carcinoma. Oncol Lett 13, 236–242. 10.3892/ol.2016.5414.
[S58] Kalimutho, M., Sinha, D., Jeffery, J., Nones, K., Srihari, S., Fernando, W.C., Duijf, P.H., Vennin, C., Raninga, P., Nanayakkara, D., et al. (2018). CEP55 is a determinant of cell fate during perturbed mitosis in breast cancer. EMBO Mol Med 10. 10.15252/emmm.201708566.
[S59] Li, G.-S., Zhang, W., Huang, W.-Y., He, R.-Q., Huang, Z.-G., Gan, X.-Y., Yang, Z., Dang, Y.-W., Kong, J.-L., Zhou, H.-F., et al. (2023). CEP55: an immune-related predictive and prognostic molecular biomarker for multiple cancers. BMC Pulm Med 23, 166. 10.1186/s12890-023-02452-1.
[S60] Lin, K., Zhu, X., Luo, C., Bu, F., Zhu, J., and Zhu, Z. (2021). Data mining combined with experiments to validate CEP55 as a prognostic biomarker in colorectal cancer. Immun Inflamm Dis 9, 167–182. 10.1002/iid3.375.
[S61] Pei, L., Dong, C., Wang, Y., Lv, X., Jia, G., and Zhang, A. (2022). Circular RNA circSDHC (hsa_circ_0015004) regulates tumor growth and angiogenesis via regulating centrosomal protein 55 expression in renal cell carcinoma. Histol Histopathol 37, 971–983. 10.14670/HH-18-467.
[S62] Qi, J., Liu, G., and Wang, F. (2018). High levels of centrosomal protein 55 expression is associated with poor clinical prognosis in patients with cervical cancer. Oncol Lett 15, 9347–9352. 10.3892/ol.2018.8448.
[S63] Sinha, D., Nag, P., Nanayakkara, D., Duijf, P.H.G., Burgess, A., Raninga, P., Smits, V.A.J., Bain, A.L., Subramanian, G., Wall, M., et al. (2020). Cep55 overexpression promotes genomic instability and tumorigenesis in mice. Commun Biol 3, 593. 10.1038/s42003-020-01304-6.
[S64] van der Horst, A., Simmons, J., and Khanna, K.K. (2009). Cep55 stabilization is required for normal execution of cytokinesis. Cell Cycle 8, 3742–3749. 10.4161/cc.8.22.10047.
[S65] Wang, G., Chen, B., Su, Y., Qu, N., Zhou, D., and Zhou, W. (2023). CEP55 as a Promising Immune Intervention Marker to Regulate Tumor Progression: A Pan-Cancer Analysis with Experimental Verification. Cells 12, 2457. 10.3390/cells12202457.
[S66] Yan, S.-M., Liu, L., Gu, W.-Y., Huang, L.-Y., Yang, Y., Huang, Y.-H., and Luo, R.-Z. (2021). CEP55 Positively Affects Tumorigenesis of Esophageal Squamous Cell Carcinoma and Is Correlated with Poor Prognosis. J Oncol 2021, 8890715. 10.1155/2021/8890715.
[S67] Yang, C., Yang, Y., Wang, W., Zhou, W., Zhang, X., Xiao, Y., and Zhang, H. (2022). CEP55 3'-UTR promotes epithelial-mesenchymal transition and enhances tumorigenicity of bladder cancer cells by acting as a ceRNA regulating miR-497-5p. Cell Oncol (Dordr) 45, 1217–1236. 10.1007/s13402-022-00712-6.
[S68] Yang, L., He, Y., Zhang, Z., and Wang, W. (2020). Upregulation of CEP55 Predicts Dismal Prognosis in Patients with Liver Cancer. Biomed Res Int 2020, 4139320. 10.1155/2020/4139320.
[S69] Zhang, X., Xu, Q., Li, E., Shi, T., and Chen, H. (2022). CEP55 predicts the poor prognosis and promotes tumorigenesis in endometrial cancer by regulating the Foxo1 signaling. Mol Cell Biochem. 10.1007/s11010-022-04607-w.
[S70] Chen, Y.-T., Hsu, M., Lee, P., Shin, S.J., Mhawech-Fauceglia, P., Odunsi, K., Altorki, N.K., Song, C.-J., Jin, B.-Q., Simpson, A.J., et al. (2009). Cancer/testis antigen CT45: analysis of mRNA and protein expression in human cancer. Int J Cancer 124, 2893–2898. 10.1002/ijc.24296.
[S71] Chen, Y.T., Ross, D.S., Chiu, R., Zhou, X.K., Chen, Y.Y., Lee, P., Hoda, S.A., Simpson, A.J., Old, L.J., Caballero, O., et al. (2011). Multiple cancer/testis antigens are preferentially expressed in hormone-receptor negative and high-grade breast cancers. PLoS One 6, e17876. 10.1371/journal.pone.0017876.

[S72] Suzuki, I., Yoshida, S., Tabu, K., Kusunoki, S., Matsumura, Y., Izumi, H., Asanoma, K., Yagi, H., Onoyama, I., Sonoda, K., et al. (2021). YBX2 and cancer testis antigen 45 contribute to stemness, chemoresistance and a high degree of malignancy in human endometrial cancer. Sci Rep 11, 4220. 10.1038/s41598-021-83200-5.
[S73] Vlasenkova, R., Konysheva, D., Nurgalieva, A., and Kiyamova, R. (2023). Characterization of Cancer/Testis Antigens as Prognostic Markers of Ovarian Cancer. Diagnostics (Basel) 13, 3092. 10.3390/diagnostics13193092.
[S74] Bi, S.Q., Peng, Y., Wei, Z.D., Yao, S.Z., Luo, B., Ge, Y.Y., Xie, X.X., Nong, W.X., Liu, C., Xiao, S.W., et al. (2022). FMR1NB Involved in Glioma Tumorigenesis Is a Promising Target for Prognosis and Therapy. Curr Med Sci 42, 803–816. 10.1007/s11596-022-2586-4.
[S75] Cappell, K.M., Sinnott, R., Taus, P., Maxfield, K., Scarbrough, M., and Whitehurst, A.W. (2012). Multiple cancer testis antigens function to support tumor cell mitotic fidelity. Mol Cell Biol 32, 4131–4140. 10.1128/MCB.00686-12.
[S76] Kim, Y.D., Park, H.R., Song, M.H., Shin, D.H., Lee, C.H., Lee, M.K., and Lee, S.Y. (2012). Pattern of cancer/testis antigen expression in lung cancer patients. Int J Mol Med 29, 656–662. 10.3892/ijmm.2012.896.
[S77] Lee, S.Y., Obata, Y., Yoshida, M., Stockert, E., Williamson, B., Jungbluth, A.A., Chen, Y.T., Old, L.J., and Scanlan, M.J. (2003). Immunomic analysis of human sarcoma. Proc Natl Acad Sci U S A 100, 2651–2656. 10.1073/pnas.0437972100.
[S78] Park, J.H., Song, M.H., Lee, C.H., Lee, M.K., Park, Y.M., Old, L., and Lee, S.Y. (2011). Expression of the human cancer/testis antigen NY-SAR-35 is activated by CpG island hypomethylation. Biotechnol Lett 33, 1085–1091. 10.1007/s10529-011-0559-y.
[S79] Song, M.H., Kim, Y.R., Bae, J.H., Shin, D.H., and Lee, S.Y. (2017). A cancer/testis antigen, NY-SAR-35, induces EpCAM, CD44, and CD133, and activates ERK in HEK293 cells. Biochem Biophys Res Commun 484, 298–303. 10.1016/j.bbrc.2017.01.105.
[S80] Song, M.H., Kim, Y.R., Lee, J.W., Lee, C.H., and Lee, S.Y. (2016). Cancer/testis antigen NY-SAR-35 enhances cell proliferation, migration, and invasion. Int J Oncol 48, 569–576. 10.3892/ijo.2015.3264.
[S81] Bissanum, R., Kamolphiwong, R., Navakanitworakul, R., and Kanokwiroon, K. (2022). Integrated bioinformatic analysis of potential biomarkers of poor prognosis in triple-negative breast cancer. Transl Cancer Res 11, 3039–3049. 10.21037/tcr-22-662.
[S82] Chen, B., Tang, H., Chen, X., Zhang, G., Wang, Y., Xie, X., and Liao, N. (2019). Transcriptomic analyses identify key differentially expressed genes and clinical outcomes between triple-negative and non-triple-negative breast cancer. Cancer Manag Res 11, 179–190. 10.2147/CMAR.S187151.
[S83] El-Botty, R., Vacher, S., Mainguené, J., Briaux, A., Ibadioune, S., Dahmani, A., Montaudon, E., Nemati, F., Huguet, L., Sourd, L., et al. (2023). HORMAD1 overexpression predicts response to anthracycline-cyclophosphamide and survival in triple-negative breast cancers. Mol Oncol 17, 2017–2028. 10.1002/1878-0261.13412.
[S84] Gantchev, J., Messina-Pacheco, J., Martínez Villarreal, A., Ramchatesingh, B., Lefrançois, P., Xie, P., Amar, L., Xu, H.H., Raveendra, K., Sikorski, D., et al. (2023). Ectopically Expressed Meiosis-Specific Cancer Testis Antigen HORMAD1 Promotes Genomic Instability in Squamous Cell Carcinomas. Cells 12, 1627. 10.3390/cells12121627.
[S85] Gao, Y., Kardos, J., Yang, Y., Tamir, T.Y., Mutter-Rottmayer, E., Weissman, B., Major, M.B., Kim, W.Y., and Vaziri, C. (2018). The Cancer/Testes (CT) Antigen HORMAD1 promotes Homologous Recombinational DNA Repair and Radioresistance in Lung adenocarcinoma cells. Sci Rep 8, 15304. 10.1038/s41598-018-33601-w.
[S86] Herrera, L.R., Johnson, R.A., McGlynn, K., Gibbs, Z.A., Davis, A.J., and Whitehurst, A.W. (2023). The cancer testes antigen, HORMAD1, limits genomic instability in cancer cells by protecting stalled replication forks. J Biol Chem 299, 105348. 10.1016/j.jbc.2023.105348.
[S87] Kogo, H., Tsutsumi, M., Ohye, T., Inagaki, H., Abe, T., and Kurahashi, H. (2012). HORMAD1-dependent checkpoint/surveillance mechanism eliminates asynaptic oocytes. Genes to Cells 17, 439–454. 10.1111/j.1365-2443.2012.01600.x.
[S88] Liu, K., Wang, Y., Zhu, Q., Li, P., Chen, J., Tang, Z., Shen, Y., Cheng, X., Lu, L.Y., and Liu, Y. (2020). Aberrantly expressed HORMAD1 disrupts nuclear localization of MCM8-MCM9 complex and compromises DNA mismatch repair in cancer cells. Cell Death Dis 11, 519. 10.1038/s41419-020-2736-1.

[S89] Nichols, B.A., Oswald, N.W., McMillan, E.A., McGlynn, K., Yan, J., Kim, M.S., Saha, J., Mallipeddi, P.L., LaDuke, S.A., Villalobos, P.A., et al. (2018). HORMAD1 Is a Negative Prognostic Indicator in Lung Adenocarcinoma and Specifies Resistance to Oxidative and Genotoxic Stress. Cancer Res 78, 6196–6208. 10.1158/0008-5472.CAN-18-1377.

[S90] Shahzad, M.M., Shin, Y.H., Matsuo, K., Lu, C., Nishimura, M., Shen, D.Y., Kang, Y., Hu, W., Mora, E.M., Rodriguez-Aguayo, C., et al. (2013). Biological significance of HORMA domain containing protein 1 (HORMAD1) in epithelial ovarian carcinoma. Cancer Lett 330, 123–129. 10.1016/j.canlet.2012.07.001.

[S91] Tarantino, D., Walker, C., Weekes, D., Pemberton, H., Davidson, K., Torga, G., Frankum, J., Mendes-Pereira, A.M., Prince, C., Ferro, R., et al. (2022). Functional screening reveals HORMAD1-driven gene dependencies associated with translesion synthesis and replication stress tolerance. Oncogene 41, 3969–3977. 10.1038/s41388-022-02369-9.

[S92] Watkins, J., Weekes, D., Shah, V., Gazinska, P., Joshi, S., Sidhu, B., Gillett, C., Pinder, S., Vanoli, F., Jasin, M., et al. (2015). Genomic Complexity Profiling Reveals That HORMAD1 Overexpression Contributes to Homologous Recombination Deficiency in Triple-Negative Breast Cancers. Cancer Discov 5, 488–505. 10.1158/2159-8290.CD-14-1092.

[S93] Zong, B., Sun, L., Peng, Y., Wang, Y., Yu, Y., Lei, J., Zhang, Y., Guo, S., Li, K., and Liu, S. (2021). HORMAD1 promotes docetaxel resistance in triple negative breast cancer by enhancing DNA damage tolerance Corrigendum in /10.3892/or.2021.8146. Oncology Reports 46, 1–15. 10.3892/or.2021.8089.

[S94] Chen, L., Wu, Q., Xu, X., Yang, C., You, J., Chen, F., and Zeng, Y. (2021). Cancer/testis antigen LDHC promotes proliferation and metastasis by activating the PI3K/Akt/GSK-3beta-signaling pathway and the in lung adenocarcinoma. Exp Cell Res 398, 112414. 10.1016/j.yexcr.2020.112414.

[S95] Cui, Z., Chen, Y., Hu, M., Lin, Y., Zhang, S., Kong, L., and Chen, Y. (2020). Diagnostic and prognostic value of the cancer-testis antigen lactate dehydrogenase C4 in breast cancer. Clin Chim Acta 503, 203–209. 10.1016/j.cca.2019.11.032.

[S96] Cui, Z., Li, Y., Gao, Y., Kong, L., Lin, Y., and Chen, Y. (2020). Cancer-testis antigen lactate dehydrogenase C4 in hepatocellular carcinoma: a promising biomarker for early diagnosis, efficacy evaluation and prognosis prediction. Aging (Albany NY) 12, 19455–19467. 10.18632/aging.103879.

[S97] Gupta, G.S. (2012). LDH-C4: a target with therapeutic potential for cancer and contraception. Mol. Cell. Biochem. 371, 115–127. 10.1007/s11010-012-1428-2.

[S98] Koslowski, M., Tureci, O., Bell, C., Krause, P., Lehr, H.A., Brunner, J., Seitz, G., Nestle, F.O., Huber, C., and Sahin, U. (2002). Multiple splice variants of lactate dehydrogenase C selectively expressed in human cancer. Cancer Res 62, 6750–6755.

[S99] Naik, A., and Decock, J. (2022). Targeting of lactate dehydrogenase C dysregulates the cell cycle and sensitizes breast cancer cells to DNA damage response targeted therapy. Mol Oncol 16, 885–903. 10.1002/1878-0261.13024.

[S100] Naik, A., and Decock, J. (2023). Commentary: Cancer-testis antigen lactate dehydrogenase C4 as a novel biomarker of male infertility and cancer. Front Oncol 13, 1115620. 10.3389/fonc.2023.1115620.

[S101] Odet, F., Gabel, S., London, R.E., Goldberg, E., and Eddy, E.M. (2013). Glycolysis and mitochondrial respiration in mouse LDHC-null sperm. Biol Reprod 88, 95. 10.1095/biolreprod.113.108530.

[S102] Odet, F., Gabel, S.A., Williams, J., London, R.E., Goldberg, E., and Eddy, E.M. (2011). Lactate dehydrogenase C and energy metabolism in mouse sperm. Biol Reprod 85, 556–564. 10.1095/biolreprod.111.091546.

[S103] Peng, W., Chen, J., Xiao, Y., Su, G., Chen, Y., and Cui, Z. (2022). Cancer-Testis Antigen LDH-C4 in Tissue, Serum, and Serum-Derived Exosomes Serves as a Promising Biomarker in Lung Adenocarcinoma. Front Oncol 12, 912624. 10.3389/fonc.2022.912624.

[S104] Tan, H., Wang, H., Ma, J., Deng, H., He, Q., Chen, Q., and Zhang, Q. (2022). Identification of human LDHC4 as a potential target for anticancer drug discovery. Acta Pharm Sin B 12, 2348–2357. 10.1016/j.apsb.2021.12.002.

[S105] Thomas, R., Shaath, H., Naik, A., Toor, S.M., Elkord, E., and Decock, J. (2020). Identification of two HLA-A*0201 immunogenic epitopes of lactate dehydrogenase C (LDHC): potential novel targets

for cancer immunotherapy. Cancer Immunol Immunother 69, 449–463. 10.1007/s00262-020-02480-4.

[S106] Wu, J., Chen, Y., Lin, Y., Lan, F., and Cui, Z. (2022). Cancer-testis antigen lactate dehydrogenase C4 as a novel biomarker of male infertility and cancer. Front Oncol 12, 936767. 10.3389/fonc.2022.936767.

[S107] Alves, P.M.S., Lévy, N., Bouzourene, H., Viatte, S., Bricard, G., Ayyoub, M., Vuilleumier, H., Givel, J.-C.R., Halkic, N., Speiser, D.E., et al. (2007). Molecular and immunological evaluation of the expression of cancer/testis gene products in human colorectal cancer. Cancer Immunol Immunother 56, 839–847. 10.1007/s00262-006-0228-5.

[S108] Baba, T., Shiota, H., Kuroda, K., Shigematsu, Y., Ichiki, Y., Uramoto, H., Hanagiri, T., and Tanaka, F. (2013). Cancer/testis antigen expression as a predictor for epidermal growth factor receptor mutation and prognosis in lung adenocarcinoma. Eur J Cardiothorac Surg 43, 759–764. 10.1093/ejcts/ezs426.

[S109] Bandić, D., Juretić, A., Sarcević, B., Separović, V., Kujundzić-Tiljak, M., Hudolin, T., Spagnoli, G.C., Ković, D., and Samija, M. (2006). Expression and possible prognostic role of MAGE-A4, NY-ESO-1, and HER-2 antigens in women with relapsing invasive ductal breast cancer: retrospective immunohistochemical study. Croat Med J 47, 32–41.

[S110] Barrow, C., Browning, J., MacGregor, D., Davis, I.D., Sturrock, S., Jungbluth, A.A., and Cebon, J. (2006). Tumor antigen expression in melanoma varies according to antigen and stage. Clin. Cancer Res. 12, 764–771. 10.1158/1078-0432.CCR-05-1544.

[S111] Bellati, F., Napoletano, C., Tarquini, E., Palaia, I., Landi, R., Manci, N., Spagnoli, G., Rughetti, A., Panici, P.B., and Nuti, M. (2007). Cancer testis antigen expression in primary and recurrent vulvar cancer: association with prognostic factors. Eur J Cancer 43, 2621–2627. 10.1016/j.ejca.2007.08.031.

[S112] Bergeron, A., Picard, V., LaRue, H., Harel, F., Hovington, H., Lacombe, L., and Fradet, Y. (2009). High frequency of MAGE-A4 and MAGE-A9 expression in high-risk bladder cancer. Int J Cancer 125, 1365–1371. 10.1002/ijc.24503.

[S113] Cabezón, T., Gromova, I., Gromov, P., Serizawa, R., Timmermans Wielenga, V., Kroman, N., Celis, J.E., and Moreira, J.M.A. (2013). Proteomic profiling of triple-negative breast carcinomas in combination with a three-tier orthogonal technology approach identifies Mage-A4 as potential therapeutic target in estrogen receptor negative breast cancer. Mol Cell Proteomics 12, 381–394. 10.1074/mcp.M112.019786.

[S114] Chen, X., Cai, S., Wang, L., Zhang, X., Li, W., and Cao, X. (2019). Analysis of the function of MAGE-A in esophageal carcinoma by bioinformatics. Medicine (Baltimore) 98, e15774. 10.1097/MD.0000000000015774.

[S115] Chen, X., Wang, L., Yue, D., Liu, J., Huang, L., Yang, L., Cao, L., Qin, G., Li, A., Wang, D., et al. (2017). Correlation between the high expression levels of cancer-germline genes with clinical characteristics in esophageal squamous cell carcinoma. Histol Histopathol 32, 793–803. 10.14670/HH-11-847.

[S116] Chitale, D.A., Jungbluth, A.A., Marshall, D.S., Leitao, M.M., Hedvat, C.V., Kolb, D., Spagnoli, G.C., Iversen, K., and Soslow, R.A. (2005). Expression of cancer-testis antigens in endometrial carcinomas using a tissue microarray. Mod Pathol 18, 119–126. 10.1038/modpathol.3800232.

[S117] Daudi, S., Eng, K.H., Mhawech-Fauceglia, P., Morrison, C., Miliotto, A., Beck, A., Matsuzaki, J., Tsuji, T., Groman, A., Gnjatic, S., et al. (2014). Expression and immune responses to MAGE antigens predict survival in epithelial ovarian cancer. PLoS One 9, e104099. 10.1371/journal.pone.0104099.

[S118] Errington, J.A., Conway, R.M., Walsh-Conway, N., Browning, J., Freyer, C., Cebon, J., and Madigan, M.C. (2012). Expression of cancer-testis antigens (MAGE-A1, MAGE-A3/6, MAGE-A4, MAGE-C1 and NY-ESO-1) in primary human uveal and conjunctival melanoma. Br J Ophthalmol 96, 451–458. 10.1136/bjophthalmol-2011-300432.

[S119] Forghanifard, M.M., Gholamin, M., Farshchian, M., Moaven, O., Memar, B., Forghani, M.N., Dadkhah, E., Naseh, H., Moghbeli, M., Raeisossadati, R., et al. (2011). Cancer-testis gene expression profiling in esophageal squamous cell carcinoma: identification of specific tumor marker and potential targets for immunotherapy. Cancer Biol Ther 12, 191–197. 10.4161/cbt.12.3.15949.

[S120] Gao, Y., Mutter-Rottmayer, E., Greenwalt, A.M., Goldfarb, D., Yan, F., Yang, Y., Martinez-Chacin, R.C., Pearce, K.H., Tateishi, S., Major, M.B., et al. (2016). A neomorphic cancer cell-specific role of MAGE-A4 in trans-lesion synthesis. Nat Commun 7, 12105. 10.1038/ncomms12105.

[S121] Garcia-Soto, A.E., Schreiber, T., Strbo, N., Ganjei-Azar, P., Miao, F., Koru-Sengul, T., Simpkins, F., Nieves-Neira, W., Lucci, J., and Podack, E.R. (2017). Cancer-testis antigen expression is shared between epithelial ovarian cancer tumors. Gynecol Oncol 145, 413–419. 10.1016/j.ygyno.2017.03.512.

[S122] Gure, A.O., Chua, R., Williamson, B., Gonen, M., Ferrera, C.A., Gnjatic, S., Ritter, G., Simpson, A.J.G., Chen, Y.-T., Old, L.J., et al. (2005). Cancer-testis genes are coordinately expressed and are markers of poor outcome in non-small cell lung cancer. Clin. Cancer Res. 11, 8055–8062. 10.1158/1078-0432.CCR-05-1203.

[S123] Hanagiri, T., Shigematsu, Y., Shinohara, S., Takenaka, M., Oka, S., Chikaishi, Y., Nagata, Y., Baba, T., Uramoto, H., So, T., et al. (2013). Clinical significance of expression of cancer/testis antigen and down-regulation of HLA class-I in patients with stage I non-small cell lung cancer. Anticancer Res 33, 2123–2128.

[S124] Hashimoto, K., Nishimura, S., Ito, T., and Akagi, M. (2022). Clinicopathological Assessment of Cancer/Testis Antigens NY-ESO-1 and MAGE-A4 in Highly Aggressive Soft Tissue Sarcomas. Diagnostics (Basel) 12, 733. 10.3390/diagnostics12030733.

[S125] Hashimoto, K., Nishimura, S., Ito, T., Kakinoki, R., and Akagi, M. (2022). Immunohistochemical expression and clinicopathological assessment of PD-1, PD-L1, NY-ESO-1, and MAGE-A4 expression in highly aggressive soft tissue sarcomas. Eur J Histochem 66, 3393. 10.4081/ejh.2022.3393.

[S126] Hashimoto, K., Nishimura, S., Ito, T., Oka, N., Kakinoki, R., and Akagi, M. (2022). Clinicopathological assessment of cancer/testis antigens NY-ESO-1 and MAGE-A4 in osteosarcoma. Eur J Histochem 66. 10.4081/ejh.2022.3377.

[S127] Hashimoto, K., Nishimura, S., Shinyashiki, Y., Ito, T., Kakinoki, R., and Akagi, M. (2023). Involvement of NY-ESO-1 and MAGE-A4 in the pathogenesis of desmoid tumors. Medicine (Baltimore) 102, e33908. 10.1097/MD.0000000000033908.

[S128] Hong, D.S., Van Tine, B.A., Biswas, S., McAlpine, C., Johnson, M.L., Olszanski, A.J., Clarke, J.M., Araujo, D., Blumenschein, G.R., Kebriaei, P., et al. (2023). Autologous T cell therapy for MAGE-A4(+) solid cancers in HLA-A*02(+) patients: a phase 1 trial. Nat Med 29, 104–114. 10.1038/s41591-022-02128-z.

[S129] Hou, Z., Liang, X., Wang, X., Zhou, Z., and Shi, G. (2020). Myeloid-derived suppressor cells infiltration in non-small-cell lung cancer tumor and MAGE-A4 and NY-ESO-1 expression. Oncol Lett 19, 3982–3992. 10.3892/ol.2020.11497.

[S130] Hussein, Y.M., Gharib, A.F., Etewa, R.L., El-Shal, A.S., Abdel-Ghany, M.E., and Elsawy, W.H. (2011). The melanoma-associated antigen-A3, -A4 genes: relation to the risk and clinicopathological parameters in breast cancer patients. Mol Cell Biochem 351, 261–268. 10.1007/s11010-011-0734-4.

[S131] Ishihara, M., Kageyama, S., Miyahara, Y., Ishikawa, T., Ueda, S., Soga, N., Naota, H., Mukai, K., Harada, N., Ikeda, H., et al. (2020). MAGE-A4, NY-ESO-1 and SAGE mRNA expression rates and co-expression relationships in solid tumours. BMC Cancer 20, 606. 10.1186/s12885-020-07098-4.

[S132] Kakimoto, T., Matsumine, A., Kageyama, S., Asanuma, K., Matsubara, T., Nakamura, T., Iino, T., Ikeda, H., Shiku, H., and Sudo, A. (2019). Immunohistochemical expression and clinicopathological assessment of the cancer testis antigens NY-ESO-1 and MAGE-A4 in high-grade soft-tissue sarcoma. Oncol Lett 17, 3937–3943. 10.3892/ol.2019.10044.

[S133] Karimi, S., Mohammadi, F., Porabdollah, M., Mohajerani, S.A., Khodadad, K., and Nadji, S.A. (2012). Characterization of melanoma-associated antigen-a genes family differential expression in non-small-cell lung cancers. Clin Lung Cancer 13, 214–219. 10.1016/j.cllc.2011.09.007.

[S134] Kocher, T., Zheng, M., Bolli, M., Simon, R., Forster, T., Schultz-Thater, E., Remmel, E., Noppen, C., Schmid, U., Ackermann, D., et al. (2002). Prognostic relevance of MAGE-A4 tumor antigen expression in transitional cell carcinoma of the urinary bladder: a tissue microarray study. Int J Cancer 100, 702–705. 10.1002/ijc.10540.

[S135] Lüftl, M., Schuler, G., and Jungbluth, A.A. (2004). Melanoma or not? Cancer testis antigens may help. Br J Dermatol 151, 1213–1218. 10.1111/j.1365-2133.2004.06260.x.

[S136] Montoro, J.R. de M.C., Mamede, R.C.M., Neder Serafini, L., Saggioro, F.P., Figueiredo, D.L.A., Silva, W.A. da, Jungbluth, A.A., Spagnoli, G.C., and Zago, M.A. (2012). Expression of cancer-testis antigens MAGE-A4 and MAGE-C1 in oral squamous cell carcinoma. Head Neck 34, 1123–1128. 10.1002/hed.21880.

[S137] Müller-Richter, U.D.A., Dowejko, A., Driemel, O., Reuther, T., Reichert, T.E., and Kübler, A.C. (2010). Impact of MAGE-A antigens on taxane response in oral squamous cell carcinoma. Oncol Lett 1, 181–185. 10.3892/ol_00000033.

[S138] Nishikawa, H., Maeda, Y., Ishida, T., Gnjatic, S., Sato, E., Mori, F., Sugiyama, D., Ito, A., Fukumori, Y., Utsunomiya, A., et al. (2012). Cancer/testis antigens are novel targets of immunotherapy for adult T-cell leukemia/lymphoma. Blood 119, 3097–3104. 10.1182/blood-2011-09-379982.

[S139] Otte, M., Zafrakas, M., Riethdorf, L., Pichlmeier, U., Löning, T., Jänicke, F., and Pantel, K. (2001). MAGE-A gene expression pattern in primary breast cancer. Cancer Res 61, 6682–6687.

[S140] Peikert, T., Specks, U., Farver, C., Erzurum, S.C., and Comhair, S.A. (2006). Melanoma antigen A4 is expressed in non-small cell lung cancers and promotes apoptosis. Cancer Res 66, 4693–4700. 10.1158/0008-5472.CAN-05-3327.

[S141] Peng, J., Chen, H., Mou, D., Cao, J., Cong, X., Qin, L., Wei, L., Leng, X., Wang, Y., and Chen, W. (2005). Expression of cancer/testis (CT) antigens in Chinese hepatocellular carcinoma and its correlation with clinical parameters. Cancer Lett. 219, 223–232. 10.1016/j.canlet.2004.07.028.

[S142] Perez, D., Herrmann, T., Jungbluth, A.A., Samartzis, P., Spagnoli, G., Demartines, N., Clavien, P.-A., Marino, S., Seifert, B., and Jaeger, D. (2008). Cancer testis antigen expression in gastrointestinal stromal tumors: new markers for early recurrence. Int J Cancer 123, 1551–1555. 10.1002/ijc.23698.

[S143] Prasad, M.L., Jungbluth, A.A., Patel, S.G., Iversen, K., Hoshaw-Woodard, S., and Busam, K.J. (2004). Expression and significance of cancer testis antigens in primary mucosal melanoma of the head and neck. Head Neck 26, 1053–1057. 10.1002/hed.20112.

[S144] Resnick, M.B., Sabo, E., Kondratev, S., Kerner, H., Spagnoli, G.C., and Yakirevich, E. (2002). Cancer-testis antigen expression in uterine malignancies with an emphasis on carcinosarcomas and papillary serous carcinomas. Int J Cancer 101, 190–195. 10.1002/ijc.10585.

[S145] Sarcevic, B., Spagnoli, G.C., Terracciano, L., Schultz-Thater, E., Heberer, M., Gamulin, M., Krajina, Z., Oresic, T., Separovic, R., and Juretic, A. (2003). Expression of cancer/testis tumor associated antigens in cervical squamous cell carcinoma. Oncology 64, 443–449. 10.1159/000070305.

[S146] Sharma, P., Shen, Y., Wen, S., Bajorin, D.F., Reuter, V.E., Old, L.J., and Jungbluth, A.A. (2006). Cancer-testis antigens: expression and correlation with survival in human urothelial carcinoma. Clin Cancer Res 12, 5442–5447. 10.1158/1078-0432.CCR-06-0527.

[S147] Shigematsu, Y., Hanagiri, T., Shiota, H., Kuroda, K., Baba, T., Mizukami, M., So, T., Ichiki, Y., Yasuda, M., So, T., et al. (2010). Clinical significance of cancer/testis antigens expression in patients with non-small cell lung cancer. Lung Cancer 68, 105–110. 10.1016/j.lungcan.2009.05.010.

[S148] Soga, N., Hori, Y., Yamakado, K., Ikeda, H., Imai, N., Kageyama, S., Nakase, K., Yuta, A., Hayashi, N., Shiku, H., et al. (2013). Limited expression of cancer-testis antigens in renal cell carcinoma patients. Mol Clin Oncol 1, 326–330. 10.3892/mco.2012.40.

[S149] Srdelić, S., Kuzmić-Prusac, I., Spagnoli, G.C., Juretić, A., and Čapkun, V. (2019). MAGE-A4 and MAGE-A1 Immunohistochemical Expression in High-grade Endometrial Cancer. Int J Gynecol Pathol 38, 59–65. 10.1097/PGP.0000000000000470.

[S150] Trippel, A., Halling, F., Heymann, P., Ayna, M., Al-Nawas, B., and Ziebart, T. (2019). The expression of melanoma-associated antigen A (MAGE-A) in oral squamous cell carcinoma: an evaluation of the significance for tumor prognosis. Oral Maxillofac Surg 23, 343–352. 10.1007/s10006-019-00778-x.

[S151] Vital, D., Ikenberg, K., Moch, H., Roessle, M., and Huber, G.F. (2018). The expression of the cancer testis antigen MAGE A4: A favorable prognostic biomarker in salivary gland carcinomas related to low tumor grading. Laryngoscope Investig Otolaryngol 3, 182–190. 10.1002/lio2.160.

[S152] Wang, M., Li, J., Wang, L., Chen, X., Zhang, Z., Yue, D., Ping, Y., Shi, X., Huang, L., Zhang, T., et al. (2015). Combined cancer testis antigens enhanced prediction accuracy for prognosis of patients with hepatocellular carcinoma. Int J Clin Exp Pathol 8, 3513–3528.

[S153] Xiao, J., Huang, F., Li, L., Zhang, L., Xie, L., and Liu, B. (2023). Expression of four cancer-testis antigens in TNBC indicating potential universal immunotherapeutic targets. J Cancer Res Clin Oncol 149, 15003–15011. 10.1007/s00432-023-05274-0.

[S154] Yakirevich, E., Sabo, E., Lavie, O., Mazareb, S., Spagnoli, G.C., and Resnick, M.B. (2003). Expression of the MAGE-A4 and NY-ESO-1 cancer-testis antigens in serous ovarian neoplasms. Clin Cancer Res 9, 6453–6460.

[S155] Yoshida, N., Abe, H., Ohkuri, T., Wakita, D., Sato, M., Noguchi, D., Miyamoto, M., Morikawa, T., Kondo, S., Ikeda, H., et al. (2006). Expression of the MAGE-A4 and NY-ESO-1 cancer-testis antigens and T cell infiltration in non-small cell lung carcinoma and their prognostic significance. Int J Oncol 28, 1089–1098.

[S156] Zimmermann, A.-K., Imig, J., Klar, A., Renner, C., Korol, D., Fink, D., Stadlmann, S., Singer, G., Knuth, A., Moch, H., et al. (2013). Expression of MAGE-C1/CT7 and selected cancer/testis antigens in ovarian borderline tumours and primary and recurrent ovarian carcinomas. Virchows Arch 462, 565–574. 10.1007/s00428-013-1395-3.

[S157] Bhatia, N., Xiao, T.Z., Rosenthal, K.A., Siddiqui, I.A., Thiyagarajan, S., Smart, B., Meng, Q., Zuleger, C.L., Mukhtar, H., Kenney, S.C., et al. (2013). MAGE-C2 promotes growth and tumorigenicity of melanoma cells, phosphorylation of KAP1, and DNA damage repair. J Invest Dermatol 133, 759–767. 10.1038/jid.2012.355.

[S158] Bode, P.K., Barghorn, A., Fritzsche, F.R., Riener, M.-O., Kristiansen, G., Knuth, A., and Moch, H. (2011). MAGEC2 is a sensitive and novel marker for seminoma: a tissue microarray analysis of 325 testicular germ cell tumors. Mod Pathol 24, 829–835. 10.1038/modpathol.2011.6.

[S159] Chen, X., Wang, L., Liu, J., Huang, L., Yang, L., Gao, Q., Shi, X., Li, J., Li, F., Zhang, Z., et al. (2017). Expression and prognostic relevance of MAGE-A3 and MAGE-C2 in non-small cell lung cancer. Oncol Lett 13, 1609–1618. 10.3892/ol.2017.5665.

[S160] Chen, Y.-T., Cao, D., Chiu, R., and Lee, P. (2013). Chromosome X-encoded Cancer/Testis antigens are less frequently expressed in non-seminomatous germ cell tumors than in seminomas. Cancer Immun 13, 10.

[S161] Chen, Y.T., Chiu, R., Lee, P., Beneck, D., Jin, B., and Old, L.J. (2011). Chromosome X-encoded cancer/testis antigens show distinctive expression patterns in developing gonads and in testicular seminoma. Hum Reprod 26, 3232–3243. 10.1093/humrep/der330.

[S162] Condomines, M., Hose, D., Raynaud, P., Hundemer, M., De Vos, J., Baudard, M., Moehler, T., Pantesco, V., Moos, M., Schved, J.-F., et al. (2007). Cancer/testis genes in multiple myeloma: expression patterns and prognosis value determined by microarray analysis. J Immunol 178, 3307–3315. 10.4049/jimmunol.178.5.3307.

[S163] Cuffel, C., Rivals, J.-P., Zaugg, Y., Salvi, S., Seelentag, W., Speiser, D.E., Liénard, D., Monnier, P., Romero, P., Bron, L., et al. (2011). Pattern and clinical significance of cancer-testis gene expression in head and neck squamous cell carcinoma. Int. J. Cancer 128, 2625–2634. 10.1002/ijc.25607.

[S164] Curioni-Fontecedro, A., Nuber, N., Mihic-Probst, D., Seifert, B., Soldini, D., Dummer, R., Knuth, A., van den Broek, M., and Moch, H. (2011). Expression of MAGE-C1/CT7 and MAGE-C2/CT10 predicts lymph node metastasis in melanoma patients. PLoS One 6, e21418. 10.1371/journal.pone.0021418.

[S165] Curioni-Fontecedro, A., Pitocco, R., Schoenewolf, N.L., Holzmann, D., Soldini, D., Dummer, R., Calvieri, S., Moch, H., Mihic-Probst, D., and Fitsche, A. (2015). Intratumoral Heterogeneity of MAGE-C1/CT7 and MAGE-C2/CT10 Expression in Mucosal Melanoma. Biomed Res Int 2015, 432479. 10.1155/2015/432479.

[S166] de Carvalho, F., Alves, V.L.F., Braga, W.M.T., Xavier, C.V., and Colleoni, G.W.B. (2013). MAGE-C1/CT7 and MAGE-C2/CT10 are frequently expressed in multiple myeloma and can be explored in combined immunotherapy for this malignancy. Cancer Immunol Immunother 62, 191–195. 10.1007/s00262-012-1376-4.

[S167] Espantman, K.C., and O'Shea, C.C. (2010). aMAGEing new players enter the RING to promote ubiquitylation. Mol Cell 39, 835–837. 10.1016/j.molcel.2010.09.006.

[S168] Figueiredo, D.L.A., Mamede, R.C.M., Spagnoli, G.C., Silva, W.A., Zago, M., Neder, L., Jungbluth, A.A., and Saggioro, F.P. (2011). High expression of cancer testis antigens MAGE-A, MAGE-C1/CT7, MAGE-C2/CT10, NY-ESO-1, and gage in advanced squamous cell carcinoma of the larynx. Head Neck 33, 702–707. 10.1002/hed.21522.

[S169] Ghadban, T., Perez, D.R., Vashist, Y.K., Bockhorn, M., Koenig, A.M., El Gammal, A.T., Izbicki, J.R., Metzger, U., Hauswirth, F., Frosina, D., et al. (2014). Expression of cancer testis antigens CT10 (MAGE-C2) and GAGE in gastrointestinal stromal tumors. Eur J Surg Oncol 40, 1307–1312. 10.1016/j.ejso.2014.03.011.

[S170] Hao, J., Song, X., Wang, J., Guo, C., Li, Y., Li, B., Zhang, Y., and Yin, Y. (2015). Cancer-testis antigen MAGE-C2 binds Rbx1 and inhibits ubiquitin ligase-mediated turnover of cyclin E. Oncotarget 6, 42028–42039. 10.18632/oncotarget.5973.

[S171] Hodgson, A., Jungbluth, A.A., Katabi, N., Xu, B., and Downes, M.R. (2020). Evaluation of cancer testis antigen (CT10, PRAME) and MHC I expression in high-grade urothelial carcinoma of the bladder. Virchows Arch 476, 535–542. 10.1007/s00428-019-02661-2.

[S172] Hou, S., Sang, M., Zhao, L., Hou, R., and Shan, B. (2016). The expression of MAGE-C1 and MAGE-C2 in breast cancer and their clinical significance. Am J Surg 211, 142–151. 10.1016/j.amjsurg.2015.05.028.

[S173] Inaoka, R.J., Jungbluth, A.A., Baiocchi, O.C., Assis, M.C., Hanson, N.C., Frosina, D., Tassello, J., Bortoluzzo, A.B., Alves, A.C., and Colleoni, G.W. (2011). An overview of cancer/testis antigens expression in classical Hodgkin's lymphoma (cHL) identifies MAGE-A family and MAGE-C1 as the most frequently expressed antigens in a set of Brazilian cHL patients. BMC Cancer 11, 416. 10.1186/1471-2407-11-416.

[S174] Lajmi, N., Luetkens, T., Yousef, S., Templin, J., Cao, Y., Hildebrandt, Y., Bartels, K., Kroger, N., and Atanackovic, D. (2015). Cancer-testis antigen MAGEC2 promotes proliferation and resistance to apoptosis in Multiple Myeloma. Br J Haematol 171, 752–762. 10.1111/bjh.13762.

[S175] Li, B., Qian, X.-P., Pang, X.-W., Zou, W.-Z., Wang, Y.-P., Wu, H.-Y., and Chen, W.-F. (2003). HCA587 antigen expression in normal tissues and cancers: correlation with tumor differentiation in hepatocellular carcinoma. Lab Invest 83, 1185–1192. 10.1097/01.lab.0000080605.73839.96.

[S176] Li, M., Yuan, Y.-H., Han, Y., Liu, Y.-X., Yan, L., Wang, Y., and Gu, J. (2005). Expression profile of cancer-testis genes in 121 human colorectal cancer tissue and adjacent normal tissue. Clin Cancer Res 11, 1809–1814. 10.1158/1078-0432.CCR-04-1365.

[S177] Liu, Y., Cao, B., Hu, L., Ye, J., Tian, W., and He, X. (2022). The Dual Roles of MAGE-C2 in p53 Ubiquitination and Cell Proliferation Through E3 Ligases MDM2 and TRIM28. Front Cell Dev Biol 10, 922675. 10.3389/fcell.2022.922675.

[S178] Mischo, A., Kubuschok, B., Ertan, K., Preuss, K.-D., Romeike, B., Regitz, E., Schormann, C., de Bruijn, D., Wadle, A., Neumann, F., et al. (2006). Prospective study on the expression of cancer testis genes and antibody responses in 100 consecutive patients with primary breast cancer. Int. J. Cancer 118, 696–703. 10.1002/ijc.21352.

[S179] Nardiello, T., Jungbluth, A.A., Mei, A., Diliberto, M., Huang, X., Dabrowski, A., Andrade, V.C.C., Wasserstrum, R., Ely, S., Niesvizky, R., et al. (2011). MAGE-A inhibits apoptosis in proliferating myeloma cells through repression of Bax and maintenance of survivin. Clin Cancer Res 17, 4309–4319. 10.1158/1078-0432.CCR-10-1820.

[S180] Oba-Shinjo, S.M., Caballero, O.L., Jungbluth, A.A., Rosemberg, S., Old, L.J., Simpson, A.J.G., and Marie, S.K.N. (2008). Cancer-testis (CT) antigen expression in medulloblastoma. Cancer Immun 8, 7.

[S181] Riener, M.-O., Wild, P.J., Soll, C., Knuth, A., Jin, B., Jungbluth, A., Hellerbrand, C., Clavien, P.-A., Moch, H., and Jochum, W. (2009). Frequent expression of the novel cancer testis antigen MAGE-C2/CT-10 in hepatocellular carcinoma. Int J Cancer 124, 352–357. 10.1002/ijc.23966.

[S182] Scanlan, M.J., Altorki, N.K., Gure, A.O., Williamson, B., Jungbluth, A., Chen, Y.T., and Old, L.J. (2000). Expression of cancer-testis antigens in lung cancer: definition of bromodomain testis-specific gene (BRDT) as a new CT gene, CT9. Cancer Lett 150, 155–164. 10.1016/s0304-3835(99)00385-7.

[S183] Sideras, K., Bots, S.J., Biermann, K., Sprengers, D., Polak, W.G., IJzermans, J.N.M., de Man, R.A., Pan, Q., Sleijfer, S., Bruno, M.J., et al. (2015). Tumour antigen expression in hepatocellular carcinoma in a low-endemic western area. Br J Cancer 112, 1911–1920. 10.1038/bjc.2015.92.

[S184] Syed, O.N., Mandigo, C.E., Killory, B.D., Canoll, P., and Bruce, J.N. (2012). Cancer-testis and melanocyte-differentiation antigen expression in malignant glioma and meningioma. J Clin Neurosci 19, 1016–1021. 10.1016/j.jocn.2011.10.008.

[S185] von Boehmer, L., Keller, L., Mortezavi, A., Provenzano, M., Sais, G., Hermanns, T., Sulser, T., Jungbluth, A.A., Old, L.J., Kristiansen, G., et al. (2011). MAGE-C2/CT10 protein expression is an

independent predictor of recurrence in prostate cancer. PLoS One 6, e21366. 10.1371/journal.pone.0021366.
[S186] Zhao, L., Mou, D.-C., Leng, X.-S., Peng, J.-R., Wang, W.-X., Huang, L., Li, S., and Zhu, J.-Y. (2004). Expression of cancer-testis antigens in hepatocellular carcinoma. World J Gastroenterol 10, 2034–2038. 10.3748/wjg.v10.i14.2034.
[S187] Zhao, Q., Xu, W.-T., and Shalieer, T. (2016). Pilot Study on MAGE-C2 as a Potential Biomarker for Triple-Negative Breast Cancer. Dis Markers 2016, 2325987. 10.1155/2016/2325987.
[S188] Zhou, X., Yang, F., Zhang, T., Zhuang, R., Sun, Y., Fang, L., Zhang, C., Ma, Y., Huang, G., Ma, F., et al. (2013). Heterogeneous expression of CT10, CT45 and GAGE7 antigens and their prognostic significance in human breast carcinoma. Jpn J Clin Oncol 43, 243–250. 10.1093/jjco/hys236.
[S189] Gu, Y., Wang, C., Zhu, R., Yang, J., Yuan, W., Zhu, Y., Zhou, Y., Qin, N., Shen, H., Ma, H., et al. (2021). The cancer-testis gene, MEIOB, sensitizes triple-negative breast cancer to PARP1 inhibitors by inducing homologous recombination deficiency. Cancer Biol Med 18, 74–87. 10.20892/j.issn.2095-3941.2020.0071.
[S190] Luo, M., Yang, F., Leu, N.A., Landaiche, J., Handel, M.A., Benavente, R., La Salle, S., and Wang, P.J. (2013). MEIOB exhibits single-stranded DNA-binding and exonuclease activities and is essential for meiotic recombination. Nat Commun 4, 2788. 10.1038/ncomms3788.
[S191] Wang, C., Gu, Y., Zhang, K., Xie, K., Zhu, M., Dai, N., Jiang, Y., Guo, X., Liu, M., Dai, J., et al. (2016). Systematic identification of genes with a cancer-testis expression pattern in 19 cancer types. Nat Commun 7, 10499. 10.1038/ncomms10499.
[S192] Abe, T., Kohashi, K., Takemoto, J., Kinoshita, F., Eto, M., and Oda, Y. (2018). Clinicopathological Significance and Antitumor Effect of MPHOSPH1 in Testicular Germ Cell Tumor. J Cancer 9, 4440–4448. 10.7150/jca.25279.
[S193] Ansari, D., Andersson, R., Bauden, M.P., Andersson, B., Connolly, J.B., Welinder, C., Sasor, A., and Marko-Varga, G. (2015). Protein deep sequencing applied to biobank samples from patients with pancreatic cancer. J Cancer Res Clin Oncol 141, 369–380. 10.1007/s00432-014-1817-x.
[S194] Chen, J., Zhao, C.C., Chen, F.R., Feng, G.W., Luo, F., and Jiang, T. (2021). KIF20B Promotes Cell Proliferation and May Be a Potential Therapeutic Target in Pancreatic Cancer. J Oncol 2021, 5572402. 10.1155/2021/5572402.
[S195] Kanehira, M., Katagiri, T., Shimo, A., Takata, R., Shuin, T., Miki, T., Fujioka, T., and Nakamura, Y. (2007). Oncogenic role of MPHOSPH1, a cancer-testis antigen specific to human bladder cancer. Cancer Res 67, 3276–3285. 10.1158/0008-5472.CAN-06-3748.
[S196] Li, G., Xie, Z.K., Zhu, D.S., Guo, T., Cai, Q.L., and Wang, Y. (2019). KIF20B promotes the progression of clear cell renal cell carcinoma by stimulating cell proliferation. J Cell Physiol 234, 16517–16525. 10.1002/jcp.28322.
[S197] Li, T.F., Zeng, H.J., Shan, Z., Ye, R.Y., Cheang, T.Y., Zhang, Y.J., Lu, S.H., Zhang, Q., Shao, N., and Lin, Y. (2020). Overexpression of kinesin superfamily members as prognostic biomarkers of breast cancer. Cancer Cell Int 20, 123. 10.1186/s12935-020-01191-1.
[S198] Li, Z.Y., Wang, Z.X., and Li, C.C. (2019). Kinesin family member 20B regulates tongue cancer progression by promoting cell proliferation. Mol Med Rep 19, 2202–2210. 10.3892/mmr.2019.9851.
[S199] Lin, W.F., Lin, X.L., Fu, S.W., Yang, L., Tang, C.T., Gao, Y.J., Chen, H.Y., and Ge, Z.Z. (2018). Pseudopod-associated protein KIF20B promotes Gli1-induced epithelial-mesenchymal transition modulated by pseudopodial actin dynamic in human colorectal cancer. Mol Carcinog 57, 911–925. 10.1002/mc.22812.
[S200] Liu, X., Li, Y., Zhang, X., Liu, X.Y., Peng, A., Chen, Y., Meng, L., Chen, H., Zhang, Y., Miao, X., et al. (2018). Inhibition of kinesin family member 20B sensitizes hepatocellular carcinoma cell to microtubule-targeting agents by blocking cytokinesis. Cancer Sci 109, 3450–3460. 10.1111/cas.13794.
[S201] Abrieu, A., Magnaghi-Jaulin, L., Kahana, J.A., Peter, M., Castro, A., Vigneron, S., Lorca, T., Cleveland, D.W., and Labbe, J.C. (2001). Mps1 is a kinetochore-associated kinase essential for the vertebrate mitotic checkpoint. Cell 106, 83–93. 10.1016/s0092-8674(01)00410-x.
[S202] Anderhub, S.J., Mak, G.W.-Y., Gurden, M.D., Faisal, A., Drosopoulos, K., Walsh, K., Woodward, H.L., Innocenti, P., Westwood, I.M., Naud, S., et al. (2019). High Proliferation Rate and a Compromised Spindle Assembly Checkpoint Confers Sensitivity to the MPS1 Inhibitor BOS172722 in Triple-Negative Breast Cancers. Mol Cancer Ther 18, 1696–1707. 10.1158/1535-7163.MCT-18-1203.

[S203] Chan, C.Y., Chiu, D.K., Yuen, V.W., Law, C.T., Wong, B.P., Thu, K.L., Cescon, D.W., Soria-Bretones, I., Cheu, J.W., Lee, D., et al. (2022). CFI-402257, a TTK inhibitor, effectively suppresses hepatocellular carcinoma. Proc Natl Acad Sci U S A 119, e2119514119. 10.1073/pnas.2119514119.

[S204] Chen, S., Wang, J., Wang, L., Peng, H., Xiao, L., Li, C., Lin, D., and Yang, K. (2019). Silencing TTK expression inhibits the proliferation and progression of prostate cancer. Exp Cell Res 385, 111669. 10.1016/j.yexcr.2019.111669.

[S205] Choi, M., Min, Y.H., Pyo, J., Lee, C.-W., Jang, C.-Y., and Kim, J.-E. (2017). TC Mps1 12, a novel Mps1 inhibitor, suppresses the growth of hepatocellular carcinoma cells via the accumulation of chromosomal instability. Br J Pharmacol 174, 1810–1825. 10.1111/bph.13782.

[S206] Colombo, R., Caldarelli, M., Mennecozzi, M., Giorgini, M.L., Sola, F., Cappella, P., Perrera, C., Depaolini, S.R., Rusconi, L., Cucchi, U., et al. (2010). Targeting the mitotic checkpoint for cancer therapy with NMS-P715, an inhibitor of MPS1 kinase. Cancer Res 70, 10255–10264. 10.1158/0008-5472.CAN-10-2101.

[S207] Gyorffy, B., Bottai, G., Lehmann-Che, J., Keri, G., Orfi, L., Iwamoto, T., Desmedt, C., Bianchini, G., Turner, N.C., de The, H., et al. (2014). TP53 mutation-correlated genes predict the risk of tumor relapse and identify MPS1 as a potential therapeutic kinase in TP53-mutated breast cancers. Mol Oncol 8, 508–519. 10.1016/j.molonc.2013.12.018.

[S208] Huang, Y.-F., Chang, M.D.-T., and Shieh, S.-Y. (2009). TTK/hMps1 mediates the p53-dependent postmitotic checkpoint by phosphorylating p53 at Thr18. Mol Cell Biol 29, 2935–2944. 10.1128/MCB.01837-08.

[S209] Jelluma, N., Brenkman, A.B., van den Broek, N.J.F., Cruijsen, C.W.A., van Osch, M.H.J., Lens, S.M.A., Medema, R.H., and Kops, G.J.P.L. (2008). Mps1 phosphorylates Borealin to control Aurora B activity and chromosome alignment. Cell 132, 233–246. 10.1016/j.cell.2007.11.046.

[S210] Jemaà, M., Galluzzi, L., Kepp, O., Senovilla, L., Brands, M., Boemer, U., Koppitz, M., Lienau, P., Prechtl, S., Schulze, V., et al. (2013). Characterization of novel MPS1 inhibitors with preclinical anticancer activity. Cell Death Differ 20, 1532–1545. 10.1038/cdd.2013.105.

[S211] Jiang, H., Yuan, F., Zhao, Z., Xue, T., Ge, N., Ren, Z., and Zhang, L. (2021). Expression and Clinical Significance of MPS-1 in Hepatocellular Carcinoma. Int J Gen Med 14, 9145–9152. 10.2147/IJGM.S334378.

[S212] Kagami, Y., Nihira, K., Wada, S., Ono, M., Honda, M., and Yoshida, K. (2014). Mps1 phosphorylation of condensin II controls chromosome condensation at the onset of mitosis. J Cell Biol 205, 781–790. 10.1083/jcb.201308172.

[S213] Kaistha, B.P., Honstein, T., Muller, V., Bielak, S., Sauer, M., Kreider, R., Fassan, M., Scarpa, A., Schmees, C., Volkmer, H., et al. (2014). Key role of dual specificity kinase TTK in proliferation and survival of pancreatic cancer cells. Br J Cancer 111, 1780–1787. 10.1038/bjc.2014.460.

[S214] Kessler, A.F., Feldheim, J., Schmitt, D., Feldheim, J.J., Monoranu, C.M., Ernestus, R.-I., Löhr, M., and Hagemann, C. (2020). Monopolar Spindle 1 Kinase (MPS1/TTK) mRNA Expression is Associated with Earlier Development of Clinical Symptoms, Tumor Aggressiveness and Survival of Glioma Patients. Biomedicines 8, 192. 10.3390/biomedicines8070192.

[S215] Liang, X.D., Dai, Y.C., Li, Z.Y., Gan, M.F., Zhang, S.R., Yin, P., Lu, H.S., Cao, X.Q., Zheng, B.J., Bao, L.F., et al. (2014). Expression and function analysis of mitotic checkpoint genes identifies TTK as a potential therapeutic target for human hepatocellular carcinoma. PLoS One 9, e97739. 10.1371/journal.pone.0097739.

[S216] Ling, Y., Zhang, X., Bai, Y., Li, P., Wei, C., Song, T., Zheng, Z., Guan, K., Zhang, Y., Zhang, B., et al. (2014). Overexpression of Mps1 in colon cancer cells attenuates the spindle assembly checkpoint and increases aneuploidy. Biochem Biophys Res Commun 450, 1690–1695. 10.1016/j.bbrc.2014.07.071.

[S217] Longo, L.V.G., Hughes, T., McNeil-Laidley, B., Cottini, F., Hilinski, G., Merritt, E., and Benson, D.M. (2023). TTK/MPS1 inhibitor OSU-13 targets the mitotic checkpoint and is a potential therapeutic strategy for myeloma. Haematologica. 10.3324/haematol.2023.282838.

[S218] Lu, L., Wang, Y., Chen, J., Li, Y., Liang, Q., Li, F., Zhen, C., and Xie, K. (2021). Targeting Mps1 in combination with paclitaxel inhibits osteosarcoma progression by modulating spindle assembly checkpoint and Akt/mTOR signaling. Oncol Lett 22, 797. 10.3892/ol.2021.13058.

[S219] Maachani, U.B., Kramp, T., Hanson, R., Zhao, S., Celiku, O., Shankavaram, U., Colombo, R., Caplen, N.J., Camphausen, K., and Tandle, A. (2015). Targeting MPS1 Enhances

Radiosensitization of Human Glioblastoma by Modulating DNA Repair Proteins. Mol Cancer Res 13, 852–862. 10.1158/1541-7786.MCR-14-0462-T.
[S220] Maia, A.R.R., de Man, J., Boon, U., Janssen, A., Song, J.-Y., Omerzu, M., Sterrenburg, J.G., Prinsen, M.B.W., Willemsen-Seegers, N., de Roos, J. a. D.M., et al. (2015). Inhibition of the spindle assembly checkpoint kinase TTK enhances the efficacy of docetaxel in a triple-negative breast cancer model. Ann Oncol 26, 2180–2192. 10.1093/annonc/mdv293.
[S221] Maia, A.R.R., Linder, S., Song, J.-Y., Vaarting, C., Boon, U., Pritchard, C.E.J., Velds, A., Huijbers, I.J., van Tellingen, O., Jonkers, J., et al. (2018). Mps1 inhibitors synergise with low doses of taxanes in promoting tumour cell death by enhancement of errors in cell division. Br J Cancer 118, 1586–1595. 10.1038/s41416-018-0081-2.
[S222] Pachis, S.T., and Kops, G.J.P.L. (2018). Leader of the SAC: molecular mechanisms of Mps1/TTK regulation in mitosis. Open Biol 8, 180109. 10.1098/rsob.180109.
[S223] Sarangapani, K.K., Koch, L.B., Nelson, C.R., Asbury, C.L., and Biggins, S. (2021). Kinetochore-bound Mps1 regulates kinetochore-microtubule attachments via Ndc80 phosphorylation. J Cell Biol 220. 10.1083/jcb.202106130.
[S224] Sarwar, S., Morozov, V.M., Purayil, H., Daaka, Y., and Ishov, A.M. (2022). Inhibition of Mps1 kinase enhances taxanes efficacy in castration resistant prostate cancer. Cell Death Dis 13, 868. 10.1038/s41419-022-05312-8.
[S225] Schöffski, P., Awada, A., de la Bigne, A.-M., Felloussi, Z., Burbridge, M., Cantero, F., Colombo, R., Maruzzelli, S., Ammattatelli, K., de Jonge, M., et al. (2022). First-in-man, first-in-class phase I study with the monopolar spindle 1 kinase inhibitor S81694 administered intravenously in adult patients with advanced, metastatic solid tumours. Eur J Cancer 169, 135–145. 10.1016/j.ejca.2022.04.001.
[S226] Shiraishi, T., Terada, N., Zeng, Y., Suyama, T., Luo, J., Trock, B., Kulkarni, P., and Getzenberg, R.H. (2011). Cancer/Testis Antigens as potential predictors of biochemical recurrence of prostate cancer following radical prostatectomy. J Transl Med 9, 153. 10.1186/1479-5876-9-153.
[S227] Simon Serrano, S., Sime, W., Abassi, Y., Daams, R., Massoumi, R., and Jemaa, M. (2020). Inhibition of mitotic kinase Mps1 promotes cell death in neuroblastoma. Sci Rep 10, 11997. 10.1038/s41598-020-68829-y.
[S228] Stucke, V.M., Silljé, H.H.W., Arnaud, L., and Nigg, E.A. (2002). Human Mps1 kinase is required for the spindle assembly checkpoint but not for centrosome duplication. EMBO J 21, 1723–1732. 10.1093/emboj/21.7.1723.
[S229] Szymiczek, A., Carbone, M., Pastorino, S., Napolitano, A., Tanji, M., Minaai, M., Pagano, I., Mason, J.M., Pass, H.I., Bray, M.R., et al. (2017). Inhibition of the spindle assembly checkpoint kinase Mps-1 as a novel therapeutic strategy in malignant mesothelioma. Oncogene 36, 6501–6507. 10.1038/onc.2017.266.
[S230] Tannous, B.A., Kerami, M., Van der Stoop, P.M., Kwiatkowski, N., Wang, J., Zhou, W., Kessler, A.F., Lewandrowski, G., Hiddingh, L., Sol, N., et al. (2013). Effects of the selective MPS1 inhibitor MPS1-IN-3 on glioblastoma sensitivity to antimitotic drugs. J Natl Cancer Inst 105, 1322–1331. 10.1093/jnci/djt168.
[S231] Tardif, K.D., Rogers, A., Cassiano, J., Roth, B.L., Cimbora, D.M., McKinnon, R., Peterson, A., Douce, T.B., Robinson, R., Dorweiler, I., et al. (2011). Characterization of the cellular and antitumor effects of MPI-0479605, a small-molecule inhibitor of the mitotic kinase Mps1. Mol Cancer Ther 10, 2267–2275. 10.1158/1535-7163.MCT-11-0453.
[S232] Waenphimai, O., Mahalapbutr, P., Vaeteewoottacharn, K., Wongkham, S., and Sawanyawisuth, K. (2022). Multiple actions of NMS-P715, the monopolar spindle 1 (MPS1) mitotic checkpoint inhibitor in liver fluke-associated cholangiocarcinoma cells. Eur J Pharmacol 922, 174899. 10.1016/j.ejphar.2022.174899.
[S233] Wengner, A.M., Siemeister, G., Koppitz, M., Schulze, V., Kosemund, D., Klar, U., Stoeckigt, D., Neuhaus, R., Lienau, P., Bader, B., et al. (2016). Novel Mps1 Kinase Inhibitors with Potent Antitumor Activity. Mol Cancer Ther 15, 583–592. 10.1158/1535-7163.MCT-15-0500.
[S234] Xu, Q., Xu, Y., Pan, B., Wu, L., Ren, X., Zhou, Y., Mao, F., Lin, Y., Guan, J., Shen, S., et al. (2016). TTK is a favorable prognostic biomarker for triple-negative breast cancer survival. Oncotarget 7, 81815–81829. 10.18632/oncotarget.13245.
[S235] Yu, L., Lang, Y., Hsu, C.C., Chen, W.M., Chiang, J.C., Hsieh, J.T., Story, M.D., Shang, Z.F., Chen, B.P.C., and Saha, D. (2022). Mitotic phosphorylation of tumor suppressor DAB2IP maintains spindle assembly checkpoint and chromosomal stability through activating PLK1-Mps1 signal

pathway and stabilizing mitotic checkpoint complex. Oncogene 41, 489–501. 10.1038/s41388-021-02106-8.
[S236] Yu, Z.-C., Huang, Y.-F., and Shieh, S.-Y. (2016). Requirement for human Mps1/TTK in oxidative DNA damage repair and cell survival through MDM2 phosphorylation. Nucleic Acids Res 44, 1133–1150. 10.1093/nar/gkv1173.
[S237] Zhang, L., Jiang, B., Zhu, N., Tao, M., Jun, Y., Chen, X., Wang, Q., and Luo, C. (2019). Mitotic checkpoint kinase Mps1/TTK predicts prognosis of colon cancer patients and regulates tumor proliferation and differentiation via PKCalpha/ERK1/2 and PI3K/Akt pathway. Med Oncol 37, 5. 10.1007/s12032-019-1320-y.
[S238] Zheng, L., Chen, Z., Kawakami, M., Chen, Y., Roszik, J., Mustachio, L.M., Kurie, J.M., Villalobos, P., Lu, W., Behrens, C., et al. (2019). Tyrosine Threonine Kinase Inhibition Eliminates Lung Cancers by Augmenting Apoptosis and Polyploidy. Mol Cancer Ther 18, 1775–1786. 10.1158/1535-7163.MCT-18-0864.
[S239] Chen, Y.T., Panarelli, N.C., Piotti, K.C., and Yantiss, R.K. (2014). Cancer-testis antigen expression in digestive tract carcinomas: frequent expression in esophageal squamous cell carcinoma and its precursor lesions. Cancer Immunol Res 2, 480–486. 10.1158/2326-6066.CIR-13-0124.
[S240] Pan, J., Eckardt, S., Leu, N.A., Buffone, M.G., Zhou, J., Gerton, G.L., McLaughlin, K.J., and Wang, P.J. (2009). Inactivation of Nxf2 causes defects in male meiosis and age-dependent depletion of spermatogonia. Dev Biol 330, 167–174. 10.1016/j.ydbio.2009.03.022.
[S241] Piotti, K.C., Scognamiglio, T., Chiu, R., and Chen, Y.T. (2013). Expression of cancer/testis (CT) antigens in squamous cell carcinoma of the head and neck: evaluation as markers of squamous dysplasia. Pathol Res Pract 209, 721–726. 10.1016/j.prp.2013.08.004.
[S242] Fujita, Y., Hayashi, T., Kiyomitsu, T., Toyoda, Y., Kokubu, A., Obuse, C., and Yanagida, M. (2007). Priming of centromere for CENP-A recruitment by human hMis18alpha, hMis18beta, and M18BP1. Dev Cell 12, 17–30. 10.1016/j.devcel.2006.11.002.
[S243] Gong, M., Li, Y., Song, E., Li, M., Qiu, S., Dong, W., and Yuan, R. (2022). OIP5 Is a Novel Prognostic Biomarker in Clear Cell Renal Cell Cancer Correlating With Immune Infiltrates. Front Immunol 13, 805552. 10.3389/fimmu.2022.805552.
[S244] He, J., Zhao, Y., Zhao, E., Wang, X., Dong, Z., Chen, Y., Yang, L., and Cui, H. (2018). Cancer-testis specific gene OIP5: a downstream gene of E2F1 that promotes tumorigenesis and metastasis in glioblastoma by stabilizing E2F1 signaling. Neuro Oncol 20, 1173–1184. 10.1093/neuonc/noy037.
[S245] He, X., Hou, J., Ping, J., Wen, D., and He, J. (2017). Opa interacting protein 5 acts as an oncogene in bladder cancer. J Cancer Res Clin Oncol 143, 2221–2233. 10.1007/s00432-017-2485-4.
[S246] Li, Y., Xiao, F., Li, W., Hu, P., Xu, R., Li, J., Li, G., and Zhu, C. (2019). Overexpression of Opa interacting protein 5 increases the progression of liver cancer via BMPR2/JUN/CHEK1/RAC1 dysregulation. Oncol Rep 41, 2075–2088. 10.3892/or.2019.7006.
[S247] Pan, M., Wang, Y., Wang, Z., Duan, H., Shao, C., Ding, P., Lei, J., Zhao, J., Ma, Z., Zhang, F., et al. (2023). The mitosis-related gene OIP5 is a potential biomarker in pan-cancer. Ann Transl Med 11, 117. 10.21037/atm-22-6640.
[S248] Rodrigues-Junior, D.M., Biassi, T.P., Carlin, V., Buri, M.V., Torrecilhas, A.C., Bortoluci, K.R., and Vettore, A.L. (2018). OIP5 Expression Sensitize Glioblastoma Cells to Lomustine Treatment. J Mol Neurosci 66, 383–389. 10.1007/s12031-018-1184-1.
[S249] Zhang, X., Gu, W., Lin, A., Duan, R., Lian, L., Huang, Y., Li, T., and Sun, Q. (2023). The role of OIP5 in the carcinogenesis and progression of ovarian cancer. J Ovarian Res 16, 185. 10.1186/s13048-023-01265-4.
[S250] Zhu, M., Takano, A., Tsevegjav, B., Yoshitake, Y., Shinohara, M., and Daigo, Y. (2022). Characterization of Opa interacting protein 5 as a new biomarker and therapeutic target for oral cancer. Int J Oncol 60. 10.3892/ijo.2022.5317.
[S251] Kulkarni, P., Dunker, A.K., Weninger, K., and Orban, J. (2016). Prostate-associated gene 4 (PAGE4), an intrinsically disordered cancer/testis antigen, is a novel therapeutic target for prostate cancer. Asian J Androl 18, 695–703. 10.4103/1008-682X.181818.
[S252] Lv, C., Fu, S., Dong, Q., Yu, Z., Zhang, G., Kong, C., Fu, C., and Zeng, Y. (2019). PAGE4 promotes prostate cancer cells survive under oxidative stress through modulating MAPK/JNK/ERK pathway. J Exp Clin Cancer Res 38, 24. 10.1186/s13046-019-1032-3.

[S253] Molania, R., Mahjoubi, F., Mirzaei, R., Khatami, S.-R., and Mahjoubi, B. (2014). A Panel of Cancer Testis Antigens and Clinical Risk Factors to Predict Metastasis in Colorectal Cancer. J Biomark 2014, 272683. 10.1155/2014/272683.

[S254] Sampson, N., Ruiz, C., Zenzmaier, C., Bubendorf, L., and Berger, P. (2012). PAGE4 positivity is associated with attenuated AR signaling and predicts patient survival in hormone-naive prostate cancer. Am J Pathol 181, 1443–1454. 10.1016/j.ajpath.2012.06.040.

[S255] Suyama, T., Shiraishi, T., Zeng, Y., Yu, W., Parekh, N., Vessella, R.L., Luo, J., Getzenberg, R.H., and Kulkarni, P. (2010). Expression of cancer/testis antigens in prostate cancer is associated with disease progression. Prostate 70, 1778–1787. 10.1002/pros.21214.

[S256] Zeng, Y., Gao, D., Kim, J.J., Shiraishi, T., Terada, N., Kakehi, Y., Kong, C., Getzenberg, R.H., and Kulkarni, P. (2013). Prostate-associated gene 4 (PAGE4) protects cells against stress by elevating p21 and suppressing reactive oxygen species production. Am J Clin Exp Urol 1, 39–52.

[S257] Chang, C.-F., Chen, S.-L., Sung, W.-W., Hsieh, M.-J., Hsu, H.-T., Chen, L.-H., Chen, M.-K., Ko, J.-L., Chen, C.-J., and Chou, M.-C. (2016). PBK/TOPK Expression Predicts Prognosis in Oral Cancer. Int J Mol Sci 17, 1007. 10.3390/ijms17071007.

[S258] Chen, J.H., Liang, Y.X., He, H.C., Chen, J.Y., Lu, J.M., Chen, G., Lin, Z.Y., Fu, X., Ling, X.H., Han, Z.D., et al. (2015). Overexpression of PDZ-binding kinase confers malignant phenotype in prostate cancer via the regulation of E2F1. Int J Biol Macromol 81, 615–623. 10.1016/j.ijbiomac.2015.08.048.

[S259] Deng, Y., Wen, H., Yang, H., Zhu, Z., Huang, Q., Bi, Y., Wang, P., Zhou, M., Guan, J., Zhang, W., et al. (2022). Identification of PBK as a hub gene and potential therapeutic target for medulloblastoma. Oncol Rep 48, 125. 10.3892/or.2022.8336.

[S260] Dong, C., Fan, W., and Fang, S. (2020). PBK as a Potential Biomarker Associated with Prognosis of Glioblastoma. J Mol Neurosci 70, 56–64. 10.1007/s12031-019-01400-1.

[S261] Feng, T., Zhang, Y., Ling, S., Xu, C., Lyu, Y., Lu, T., Liu, X., Ying, L., Wan, Y., Zhong, H., et al. (2021). PDZ Binding Kinase/T-LAK Cell-Derived Protein Kinase Plays an Oncogenic Role and Promotes Immune Escape in Human Tumors. J Oncol 2021, 8892479. 10.1155/2021/8892479.

[S262] Gao, T., Hu, Q., Hu, X., Lei, Q., Feng, Z., Yu, X., Peng, C., Song, X., He, H., Xu, Y., et al. (2019). Novel selective TOPK inhibitor SKLB-C05 inhibits colorectal carcinoma growth and metastasis. Cancer Lett 445, 11–23. 10.1016/j.canlet.2018.12.016.

[S263] Han, Z., Li, L., Huang, Y., Zhao, H., and Luo, Y. (2021). PBK/TOPK: A Therapeutic Target Worthy of Attention. Cells 10, 371. 10.3390/cells10020371.

[S264] Hayashi, T., Hayakawa, Y., Koh, M., Tomita, T., Nagai, S., Kashiwazaki, D., Sugimori, M., Origasa, H., and Kuroda, S. (2018). Impact of a novel biomarker, T-LAK cell-originating protein kinase (TOPK) expression on outcome in malignant glioma. Neuropathology 38, 144–153. 10.1111/neup.12446.

[S265] He, F., Yan, Q., Fan, L., Liu, Y., Cui, J., Wang, J., Wang, L., Wang, Y., Wang, Z., Guo, Y., et al. (2010). PBK/TOPK in the differential diagnosis of cholangiocarcinoma from hepatocellular carcinoma and its involvement in prognosis of human cholangiocarcinoma. Hum Pathol 41, 415–424. 10.1016/j.humpath.2009.05.016.

[S266] Herbert, K.J., Puliyadi, R., Prevo, R., Rodriguez-Berriguete, G., Ryan, A., Ramadan, K., and Higgins, G.S. (2021). Targeting TOPK sensitises tumour cells to radiation-induced damage by enhancing replication stress. Cell Death Differ 28, 1333–1346. 10.1038/s41418-020-00655-1.

[S267] Hu, F., Gartenhaus, R.B., Eichberg, D., Liu, Z., Fang, H.B., and Rapoport, A.P. (2010). PBK/TOPK interacts with the DBD domain of tumor suppressor p53 and modulates expression of transcriptional targets including p21. Oncogene 29, 5464–5474. 10.1038/onc.2010.275.

[S268] Hu, F., Gartenhaus, R.B., Zhao, X.F., Fang, H.-B., Minkove, S., Poss, D.E., and Rapoport, A.P. (2013). c-Myc and E2F1 drive PBK/TOPK expression in high-grade malignant lymphomas. Leuk Res 37, 447–454. 10.1016/j.leukres.2012.11.010.

[S269] Huang, H., Lee, M.H., Liu, K., Dong, Z., Ryoo, Z., and Kim, M.O. (2021). PBK/TOPK: An Effective Drug Target with Diverse Therapeutic Potential. Cancers (Basel) 13. 10.3390/cancers13092232.

[S270] Ikeda, Y., Park, J.-H., Miyamoto, T., Takamatsu, N., Kato, T., Iwasa, A., Okabe, S., Imai, Y., Fujiwara, K., Nakamura, Y., et al. (2016). T-LAK Cell-Originated Protein Kinase (TOPK) as a Prognostic Factor and a Potential Therapeutic Target in Ovarian Cancer. Clin Cancer Res 22, 6110–6117. 10.1158/1078-0432.CCR-16-0207.

[S271] Ishikawa, C., Senba, M., and Mori, N. (2018). Mitotic kinase PBK/TOPK as a therapeutic target for adult T-cell leukemia/lymphoma. Int J Oncol 53, 801–814. 10.3892/ijo.2018.4427.

[S272] Kar, A., Zhang, Y., Yacob, B.W., Saeed, J., Tompkins, K.D., Bagby, S.M., Pitts, T.M., Somerset, H., Leong, S., Wierman, M.E., et al. (2019). Targeting PDZ-binding kinase is anti-tumorigenic in novel preclinical models of ACC. Endocr Relat Cancer 26, 765–778. 10.1530/ERC-19-0262.

[S273] Kwon, C.H., Park, H.J., Choi, Y.R., Kim, A., Kim, H.W., Choi, J.H., Hwang, C.S., Lee, S.J., Choi, C.I., Jeon, T.Y., et al. (2016). PSMB8 and PBK as potential gastric cancer subtype-specific biomarkers associated with prognosis. Oncotarget 7, 21454–21468. 10.18632/oncotarget.7411.

[S274] Lee, D.H., Jeong, Y.J., Won, J.Y., Sim, H.I., Park, Y., and Jin, H.S. (2022). PBK/TOPK Is a Favorable Prognostic Biomarker Correlated with Antitumor Immunity in Colon Cancers. Biomedicines 10. 10.3390/biomedicines10020299.

[S275] Lei, B., Liu, S., Qi, W., Zhao, Y., Li, Y., Lin, N., Xu, X., Zhi, C., Mei, J., Yan, Z., et al. (2013). PBK/TOPK expression in non-small-cell lung cancer: its correlation and prognostic significance with Ki67 and p53 expression. Histopathology 63, 696–703. 10.1111/his.12215.

[S276] Lei, B., Qi, W., Zhao, Y., Li, Y., Liu, S., Xu, X., Zhi, C., Wan, L., and Shen, H. (2015). PBK/TOPK expression correlates with mutant p53 and affects patients' prognosis and cell proliferation and viability in lung adenocarcinoma. Hum Pathol 46, 217–224. 10.1016/j.humpath.2014.07.026.

[S277] Li, F., Liu, C., Nong, W., Lin, L., Ge, Y., Luo, B., Xiao, S., Zhang, Q., and Xie, X. (2023). Identification of potential biomarkers in cancer testis antigens for glioblastoma. Am J Transl Res 15, 799–816.

[S278] Li, J., and Hou, W. (2021). Expression patterns and clinical significances of PBK in lung cancer: an analysis based on Oncomine database. Transl Cancer Res 10, 2036–2043. 10.21037/tcr-20-3435.

[S279] Liu, K., Chen, Y., Feng, P., Wang, Y., Sun, M., Song, T., Tan, J., Li, C., Liu, S., Kong, Q., et al. (2022). Identification of Pathologic and Prognostic Genes in Prostate Cancer Based on Database Mining. Front Genet 13, 854531. 10.3389/fgene.2022.854531.

[S280] Liu, Y., Liu, H., Cao, H., Song, B., Zhang, W., and Zhang, W. (2015). PBK/TOPK mediates promyelocyte proliferation via Nrf2-regulated cell cycle progression and apoptosis. Oncol Rep 34, 3288–3296. 10.3892/or.2015.4308.

[S281] Liu, Y., Xiang, J., Peng, G., and Shen, C. (2021). Omics- and Pharmacogenomic Evidence for the Prognostic, Regulatory, and Immune-Related Roles of PBK in a Pan-Cancer Cohort. Front Mol Biosci 8, 785370. 10.3389/fmolb.2021.785370.

[S282] Luo, Q., Lei, B., Liu, S., Chen, Y., Sheng, W., Lin, P., Li, W., Zhu, H., and Shen, H. (2014). Expression of PBK/TOPK in cervical cancer and cervical intraepithelial neoplasia. Int J Clin Exp Pathol 7, 8059–8064.

[S283] Ma, H., Han, F., Yan, X., Qi, G., Li, Y., Li, R., Yan, S., Yuan, C., Song, K., and Kong, B. (2021). PBK promotes aggressive phenotypes of cervical cancer through ERK/c-Myc signaling pathway. J Cell Physiol 236, 2767–2781. 10.1002/jcp.30134.

[S284] Ma, H., Li, Y., Wang, X., Wu, H., Qi, G., Li, R., Yang, N., Gao, M., Yan, S., Yuan, C., et al. (2019). PBK, targeted by EVI1, promotes metastasis and confers cisplatin resistance through inducing autophagy in high-grade serous ovarian carcinoma. Cell Death Dis 10, 166. 10.1038/s41419-019-1415-6.

[S285] Ma, H., Qi, G., Han, F., Peng, J., Yuan, C., and Kong, B. (2022). PBK drives PARP inhibitor resistance through the TRIM37/NFκB axis in ovarian cancer. Exp Mol Med 54, 999–1010. 10.1038/s12276-022-00809-w.

[S286] Ma, H., Zhang, J., Shi, Y., Wang, Z., Nie, W., Cai, J., Huang, Y., Liu, B., Wang, X., and Lian, C. (2023). PBK correlates with prognosis, immune escape and drug response in LUAD. Sci Rep 13, 20452. 10.1038/s41598-023-47781-7.

[S287] Mao, P., Bao, G., Wang, Y.-C., Du, C.-W., Yu, X., Guo, X.-Y., Li, R.-C., and Wang, M.-D. (2020). PDZ-Binding Kinase-Dependent Transcriptional Regulation of CCNB2 Promotes Tumorigenesis and Radio-Resistance in Glioblastoma. Transl Oncol 13, 287–294. 10.1016/j.tranon.2019.09.011.

[S288] Mu, W., Xie, Y., Li, J., Yan, R., Zhang, J., Liu, Y., and Fan, Y. (2022). High expression of PDZ-binding kinase is correlated with poor prognosis and immune infiltrates in hepatocellular carcinoma. World J Surg Oncol 20, 22. 10.1186/s12957-021-02479-w.

[S289] Nagano-Matsuo, A., Inoue, S., Koshino, A., Ota, A., Nakao, K., Komura, M., Kato, H., Naiki-Ito, A., Watanabe, K., Nagayasu, Y., et al. (2021). PBK expression predicts favorable survival in colorectal cancer patients. Virchows Arch 479, 277–284. 10.1007/s00428-021-03062-0.

[S290] Nandi, A., Tidwell, M., Karp, J., and Rapoport, A.P. (2004). Protein expression of PDZ-binding kinase is up-regulated in hematologic malignancies and strongly down-regulated during terminal differentiation of HL-60 leukemic cells. Blood Cells Mol Dis 32, 240–245. 10.1016/j.bcmd.2003.10.004.
[S291] Ohashi, T., Komatsu, S., Ichikawa, D., Miyamae, M., Okajima, W., Imamura, T., Kiuchi, J., Kosuga, T., Konishi, H., Shiozaki, A., et al. (2017). Overexpression of PBK/TOPK relates to tumour malignant potential and poor outcome of gastric carcinoma. Br J Cancer 116, 218–226. 10.1038/bjc.2016.394.
[S292] Ohashi, T., Komatsu, S., Ichikawa, D., Miyamae, M., Okajima, W., Imamura, T., Kiuchi, J., Nishibeppu, K., Kosuga, T., Konishi, H., et al. (2016). Overexpression of PBK/TOPK Contributes to Tumor Development and Poor Outcome of Esophageal Squamous Cell Carcinoma. Anticancer Res 36, 6457–6466. 10.21873/anticanres.11244.
[S293] Park, J.H., Lin, M.L., Nishidate, T., Nakamura, Y., and Katagiri, T. (2006). PDZ-binding kinase/T-LAK cell-originated protein kinase, a putative cancer/testis antigen with an oncogenic activity in breast cancer. Cancer Res 66, 9186–9195. 10.1158/0008-5472.CAN-06-1601.
[S294] Park, J.-H., Park, S.-A., Lee, Y.-J., Park, H.-W., and Oh, S.-M. (2020). PBK attenuates paclitaxel-induced autophagic cell death by suppressing p53 in H460 non-small-cell lung cancer cells. FEBS Open Bio 10, 937–950. 10.1002/2211-5463.12855.
[S295] Qiao, L., Ba, J., Xie, J., Zhu, R., Wan, Y., Zhang, M., Jin, Z., Guo, Z., Yu, J., Chen, S., et al. (2022). Overexpression of PBK/TOPK relates to poor prognosis of patients with breast cancer: a retrospective analysis. World J Surg Oncol 20, 316. 10.1186/s12957-022-02769-x.
[S296] Shih, M.-C., Chen, J.-Y., Wu, Y.-C., Jan, Y.-H., Yang, B.-M., Lu, P.-J., Cheng, H.-C., Huang, M.-S., Yang, C.-J., Hsiao, M., et al. (2012). TOPK/PBK promotes cell migration via modulation of the PI3K/PTEN/AKT pathway and is associated with poor prognosis in lung cancer. Oncogene 31, 2389–2400. 10.1038/onc.2011.419.
[S297] Singh, P.K., Srivastava, A.K., Dalela, D., Rath, S.K., Goel, M.M., and Bhatt, M.L.B. (2014). Expression of PDZ-binding kinase/T-LAK cell-originated protein kinase (PBK/TOPK) in human urinary bladder transitional cell carcinoma. Immunobiology 219, 469–474. 10.1016/j.imbio.2014.02.003.
[S298] Su, T.C., Chen, C.Y., Tsai, W.C., Hsu, H.T., Yen, H.H., Sung, W.W., and Chen, C.J. (2018). Cytoplasmic, nuclear, and total PBK/TOPK expression is associated with prognosis in colorectal cancer patients: A retrospective analysis based on immunohistochemistry stain of tissue microarrays. PLoS One 13, e0204866. 10.1371/journal.pone.0204866.
[S299] Thanindratarn, P., Wei, R., Dean, D.C., Singh, A., Federman, N., Nelson, S.D., Hornicek, F.J., and Duan, Z. (2021). T-LAK cell-originated protein kinase (TOPK): an emerging prognostic biomarker and therapeutic target in osteosarcoma. Mol Oncol 15, 3721–3737. 10.1002/1878-0261.13039.
[S300] Wang, K., Chai, J., Xu, J., Wei, J., Li, P., Liu, Y., Ma, J., Xu, T., Zhao, D., Yu, K., et al. (2021). TOPK: A new predictor of the therapeutic response to neoadjuvant chemotherapy and prognosis in triple-negative breast cancer. Pathol Res Pract 226, 153603. 10.1016/j.prp.2021.153603.
[S301] Wen, H., Chen, Z., Li, M., Huang, Q., Deng, Y., Zheng, J., Xiong, M., Wang, P., and Zhang, W. (2021). An Integrative Pan-Cancer Analysis of PBK in Human Tumors. Front Mol Biosci 8, 755911. 10.3389/fmolb.2021.755911.
[S302] Wu, W., Xu, J., Gao, D., Xie, Z., Chen, W., Li, W., Yuan, Q., Duan, L., Zhang, Y., Yang, X., et al. (2023). TOPK promotes the growth of esophageal cancer in vitro and in vivo by enhancing YB1/eEF1A1 signal pathway. Cell Death Dis 14, 364. 10.1038/s41419-023-05883-0.
[S303] Yu, W.-N., Lin, H.-F., Lee, Y.I., Shia, W.-C., Sung, W.-W., Yeh, C.-M., and Lin, Y.-M. (2021). PBK Expression Is Associated With Prognosis of Patients With Oral Squamous Cell Carcinoma Treated With Radiotherapy: A Retrospective Study. Anticancer Res 41, 2177–2182. 10.21873/anticanres.14991.
[S304] Zhang, Y., Yang, X., Wang, R., and Zhang, X. (2019). Prognostic Value of PDZ-Binding Kinase/T-LAK Cell-Originated Protein Kinase (PBK/TOPK) in Patients with Cancer. J Cancer 10, 131–137. 10.7150/jca.28216.
[S305] Zheng, L., Li, L., Xie, J., Jin, H., and Zhu, N. (2021). Six Novel Biomarkers for Diagnosis and Prognosis of Esophageal squamous cell carcinoma: validated by scRNA-seq and qPCR. J Cancer 12, 899–911. 10.7150/jca.50443.

[S306] Zhou, L., Zhang, Y., Wei, M., Du, K., Lin, J., and Wei, L. (2023). Comprehensive analysis of CXCL14 uncovers its role during liver metastasis in colon cancer. BMC Gastroenterol 23, 273. 10.1186/s12876-023-02896-z.

[S307] Gantchev, J., Martinez Villarreal, A., Gunn, S., Zetka, M., Odum, N., and Litvinov, I.V. (2020). The ectopic expression of meiCT genes promotes meiomitosis and may facilitate carcinogenesis. Cell Cycle 19, 837–854. 10.1080/15384101.2020.1743902.

[S308] Houle, A.A., Gibling, H., Lamaze, F.C., Edgington, H.A., Soave, D., Fave, M.J., Agbessi, M., Bruat, V., Stein, L.D., and Awadalla, P. (2018). Aberrant PRDM9 expression impacts the pan-cancer genomic landscape. Genome Res 28, 1611–1620. 10.1101/gr.231696.117.

[S309] Hussin, J., Sinnett, D., Casals, F., Idaghdour, Y., Bruat, V., Saillour, V., Healy, J., Grenier, J.C., de Malliard, T., Busche, S., et al. (2013). Rare allelic forms of PRDM9 associated with childhood leukemogenesis. Genome Res 23, 419–430. 10.1101/gr.144188.112.

[S310] Kaiser, V.B., and Semple, C.A. (2018). Chromatin loop anchors are associated with genome instability in cancer and recombination hotspots in the germline. Genome Biol 19, 101. 10.1186/s13059-018-1483-4.

[S311] Agarwal, S., Parashar, D., Gupta, N., Jagadish, N., Thakar, A., Suri, V., Kumar, R., Gupta, A., Ansari, A.S., Lohiya, N.K., et al. (2015). Sperm associated antigen 9 (SPAG9) expression and humoral response in benign and malignant salivary gland tumors. Oncoimmunology 3, e974382. 10.4161/2162402X.2014.974382.

[S312] Baser, E., Togrul, C., Ozgu, E., Ayhan, S., Caglar, M., Erkaya, S., and Gungor, T. (2013). Sperm-associated antigen 9 is a promising marker for early diagnosis of endometrial cancer. Asian Pac J Cancer Prev 14, 7635–7638. 10.7314/apjcp.2013.14.12.7635.

[S313] Bi, B.-A., C, R.C., N, A., Am, K., N, T., D, M., M, S., M, K.S., A, P., D, M., et al. (2023). An HLA-G/SPAG9/STAT3 axis promotes brain metastases. Proceedings of the National Academy of Sciences of the United States of America 120. 10.1073/pnas.2205247120.

[S314] Garg, M., Kanojia, D., Suri, S., Gupta, S., Gupta, A., and Suri, A. (2009). Sperm-associated antigen 9: a novel diagnostic marker for thyroid cancer. J Clin Endocrinol Metab 94, 4613–4618. 10.1210/jc.2009-0703.

[S315] Jagadish, N., Fatima, R., Sharma, A., Devi, S., Suri, V., Kumar, V., and Suri, A. (2018). Sperm associated antigen 9 (SPAG9) a promising therapeutic target of ovarian carcinoma. Tumour Biol 40, 1010428318773652. 10.1177/1010428318773652.

[S316] Kanojia, D., Garg, M., Gupta, S., Gupta, A., and Suri, A. (2011). Sperm-associated antigen 9 is a novel biomarker for colorectal cancer and is involved in tumor growth and tumorigenicity. Am J Pathol 178, 1009–1020. 10.1016/j.ajpath.2010.11.047.

[S317] Kanojia, D., Garg, M., Saini, S., Agarwal, S., Kumar, R., and Suri, A. (2010). Sperm associated antigen 9 expression and humoral response in chronic myeloid leukemia. Leuk Res 34, 858–863. 10.1016/j.leukres.2010.01.017.

[S318] Kanojia, D., Garg, M., Saini, S., Agarwal, S., Parashar, D., Jagadish, N., Seth, A., Bhatnagar, A., Gupta, A., Kumar, R., et al. (2013). Sperm associated antigen 9 plays an important role in bladder transitional cell carcinoma. PLoS One 8, e81348. 10.1371/journal.pone.0081348.

[S319] Li, H., Peng, Y., Niu, H., Wu, B., Zhang, Y., Zhang, Y., Bai, X., and He, P. (2014). SPAG9 is overexpressed in human prostate cancer and promotes cancer cell proliferation. Tumour Biol 35, 6949–6954. 10.1007/s13277-014-1947-4.

[S320] Luo, S., Ren, B., Zou, G., Liu, J., Chen, W., Huang, Y., Chen, X., and Fu, Y. (2019). SPAG9/MKK3/p38 axis is a novel therapeutic target for liver cancer. Oncol Rep 41, 2329–2336. 10.3892/or.2019.6987.

[S321] Miao, Z.F., Wang, Z.N., Zhao, T.T., Xu, Y.Y., Wu, J.H., Liu, X.Y., Xu, H., You, Y., and Xu, H.M. (2015). Overexpression of SPAG9 in human gastric cancer is correlated with poor prognosis. Virchows Arch 467, 525–533. 10.1007/s00428-015-1826-4.

[S322] Pan, J., Yu, H., Guo, Z., Liu, Q., Ding, M., Xu, K., and Mao, L. (2018). Emerging role of sperm-associated antigen 9 in tumorigenesis. Biomed Pharmacother 103, 1212–1216. 10.1016/j.biopha.2018.04.168.

[S323] Qiao, L., Zhang, L., and Wang, H. (2023). SPAG9 Expression Predicts Good Prognosis in Patients with Clear-Cell Renal Cell Carcinoma: A Bioinformatics Analysis with Experimental Validation. Genes (Basel) 14, 944. 10.3390/genes14040944.

[S324] Ren, B., Wei, X., Zou, G., He, J., Xu, G., Xu, F., Huang, Y., Zhu, H., Li, Y., Ma, G., et al. (2016). Cancer testis antigen SPAG9 is a promising marker for the diagnosis and treatment of lung cancer. Oncol Rep 35, 2599–2605. 10.3892/or.2016.4645.

[S325] Seleit, I., Bakry, O.A., Samaka, R.M., and Malak, M.A. (2015). Immunohistochemical expression of sperm-associated antigen 9 in nonmelanoma skin cancer. Am J Dermatopathol 37, 38–45. 10.1097/DAD.0000000000000126.

[S326] Sun, H.-F., Wang, W.-D., and Feng, L. (2017). Effect of SPAG9 on migration, invasion and prognosis of prostate cancer. Int J Clin Exp Pathol 10, 9468–9474.

[S327] Wang, Y., Dong, Q., Miao, Y., Fu, L., Lin, X., and Wang, E. (2013). Clinical significance and biological roles of SPAG9 overexpression in non-small cell lung cancer. Lung Cancer 81, 266–272. 10.1016/j.lungcan.2013.04.021.

[S328] Xiao, C., Fu, L., Yan, C., Shou, F., Liu, Q., Li, L., Cui, S., Duan, J., Jin, G., Chen, J., et al. (2016). SPAG9 is overexpressed in osteosarcoma, and regulates cell proliferation and invasion through regulation of JunD. Oncol Lett 12, 2674–2679. 10.3892/ol.2016.4920.

[S329] Xie, C., Fu, L., Liu, N., and Li, Q. (2014). Overexpression of SPAG9 correlates with poor prognosis and tumor progression in hepatocellular carcinoma. Tumour Biol 35, 7685–7691. 10.1007/s13277-014-2030-x.

[S330] Yan, Q., Yang, C., Fu, Q., Chen, Z., Liu, S., Fu, D., Rahman, R.N., Nakazato, R., Yoshioka, K., Kung, S.K.P., et al. (2017). Scaffold protein JLP mediates TCR-initiated CD4+T cell activation and CD154 expression. Mol Immunol 87, 258–266. 10.1016/j.molimm.2017.05.006.

[S331] Yang, C., Shen, B., Zhang, J., and Zhang, Q. (2016). Sperm-associated antigen 9 overexpression correlates with poor prognosis and insensitive to Taxol treatment in breast cancer. Biomarkers 21, 62–67. 10.3109/1354750X.2015.1118534.

[S332] Yi, F., Ni, W., Liu, W., Pan, X., Han, X., Yang, L., Kong, X., Ma, R., and Chang, R. (2013). SPAG9 is overexpressed in human astrocytoma and promotes cell proliferation and invasion. Tumour Biol 34, 2849–2855. 10.1007/s13277-013-0845-5.

[S333] Baudat, F., Manova, K., Yuen, J.P., Jasin, M., and Keeney, S. (2000). Chromosome synapsis defects and sexually dimorphic meiotic progression in mice lacking Spo11. Mol Cell 6, 989–998. 10.1016/s1097-2765(00)00098-8.

[S334] Eldai, H., Periyasamy, S., Al Qarni, S., Al Rodayyan, M., Muhammed Mustafa, S., Deeb, A., Al Sheikh, E., Afzal, M., Johani, M., Yousef, Z., et al. (2013). Novel genes associated with colorectal cancer are revealed by high resolution cytogenetic analysis in a patient specific manner. PLoS One 8, e76251. 10.1371/journal.pone.0076251.

[S335] Keeney, S. (2008). Spo11 and the Formation of DNA Double-Strand Breaks in Meiosis. Genome Dyn Stab 2, 81–123. 10.1007/7050_2007_026.

[S336] Lindsey, S.F., Byrnes, D.M., Eller, M.S., Rosa, A.M., Dabas, N., Escandon, J., and Grichnik, J.M. (2013). Potential role of meiosis proteins in melanoma chromosomal instability. J Skin Cancer 2013, 190109. 10.1155/2013/190109.

[S337] Litvinov, I.V., Cordeiro, B., Huang, Y., Zargham, H., Pehr, K., Dore, M.A., Gilbert, M., Zhou, Y., Kupper, T.S., and Sasseville, D. (2014). Ectopic expression of cancer-testis antigens in cutaneous T-cell lymphoma patients. Clin Cancer Res 20, 3799–3808. 10.1158/1078-0432.CCR-14-0307.

[S338] Nielsen, A.Y., and Gjerstorff, M.F. (2016). Ectopic Expression of Testis Germ Cell Proteins in Cancer and Its Potential Role in Genomic Instability. Int J Mol Sci 17. 10.3390/ijms17060890.

[S339] Wei, M., Su, J., Zhang, J., Liu, S., Ma, J., and Meng, X.P. (2023). Construction of a DDR-related signature for predicting of prognosis in metastatic colorectal carcinoma. Front Oncol 13, 1043160. 10.3389/fonc.2023.1043160.

[S340] Brückmann, N.H., Bennedsen, S.N., Duijf, P.H.G., Terp, M.G., Thomassen, M., Larsen, M., Pedersen, C.B., Kruse, T., Alcaraz, N., Ditzel, H.J., et al. (2019). A functional genetic screen identifies the Mediator complex as essential for SSX2-induced senescence. Cell Death Dis 10, 841. 10.1038/s41419-019-2068-1.

[S341] Cordier, F., Van der Meulen, J., Van Gaever, B., Lapeire, L., Sys, G., Van Dorpe, J., and Creytens, D. (2022). Undifferentiated sarcoma of bone with a round to epithelioid cell phenotype harboring a novel EWSR1-SSX2 fusion identified by RNA-based next-generation sequencing. Genes, Chromosomes and Cancer 61, 44–49. 10.1002/gcc.22999.

[S342] Eisenhardt, A.E., Brugger, Z., Lausch, U., Kiefer, J., Zeller, J., Runkel, A., Schmid, A., Bronsert, P., Wehrle, J., Leithner, A., et al. (2022). Genotyping of Circulating Free DNA Enables Monitoring of Tumor Dynamics in Synovial Sarcomas. Cancers (Basel) 14, 2078. 10.3390/cancers14092078.

[S343] Gjerstorff, M.F., Relster, M.M., Greve, K.B.V., Moeller, J.B., Elias, D., Lindgreen, J.N., Schmidt, S., Mollenhauer, J., Voldborg, B., Pedersen, C.B., et al. (2014). SSX2 is a novel DNA-binding protein that antagonizes polycomb group body formation and gene repression. Nucleic Acids Res 42, 11433–11446. 10.1093/nar/gku852.

[S344] Greve, K.B., Lindgreen, J.N., Terp, M.G., Pedersen, C.B., Schmidt, S., Mollenhauer, J., Kristensen, S.B., Andersen, R.S., Relster, M.M., Ditzel, H.J., et al. (2015). Ectopic expression of cancer/testis antigen SSX2 induces DNA damage and promotes genomic instability. Mol Oncol 9, 437–449. 10.1016/j.molonc.2014.09.001.

[S345] Traynor, S., Mollegaard, N.E., Jorgensen, M.G., Bruckmann, N.H., Pedersen, C.B., Terp, M.G., Johansen, S., Dejardin, J., Ditzel, H.J., and Gjerstorff, M.F. (2019). Remodeling and destabilization of chromosome 1 pericentromeric heterochromatin by SSX proteins. Nucleic Acids Res 47, 6668–6684. 10.1093/nar/gkz396.

[S346] Türeci, O., Sahin, U., Schobert, I., Koslowski, M., Scmitt, H., Schild, H.J., Stenner, F., Seitz, G., Rammensee, H.G., and Pfreundschuh, M. (1996). The SSX-2 gene, which is involved in the t(X;18) translocation of synovial sarcomas, codes for the human tumor antigen HOM-MEL-40. Cancer Res 56, 4766–4772.

[S347] Zaborowski, M., Vargas, A.C., Pulvers, J., Clarkson, A., de Guzman, D., Sioson, L., Maclean, F., Chou, A., and Gill, A.J. (2020). When used together SS18-SSX fusion-specific and SSX C-terminus immunohistochemistry are highly specific and sensitive for the diagnosis of synovial sarcoma and can replace FISH or molecular testing in most cases. Histopathology 77, 588–600. 10.1111/his.14190.

[S348] Zhang, Y., Bao, L., Lu, J., Liu, K.-Y., Li, J.-L., Qin, Y.-Z., Chen, H., Li, L.-D., Kong, Y., Shi, H.-X., et al. (2014). The clinical value of the quantitative detection of four cancer-testis antigen genes in multiple myeloma. Mol Cancer 13, 25. 10.1186/1476-4598-13-25.

[S349] Cho, H., Noh, K.H., Chung, J.-Y., Takikita, M., Chung, E.J., Kim, B.W., Hewitt, S.M., Kim, T.W., and Kim, J.-H. (2014). Synaptonemal complex protein 3 is a prognostic marker in cervical cancer. PLoS One 9, e98712. 10.1371/journal.pone.0098712.

[S350] Chung, J.Y., Kitano, H., Takikita, M., Cho, H., Noh, K.H., Kim, T.W., Ylaya, K., Hanaoka, J., Fukuoka, J., and Hewitt, S.M. (2013). Synaptonemal complex protein 3 as a novel prognostic marker in early stage non-small cell lung cancer. Hum Pathol 44, 472–479. 10.1016/j.humpath.2012.06.018.

[S351] Hosoya, N., and Miyagawa, K. (2021). Synaptonemal complex proteins modulate the level of genome integrity in cancers. Cancer Sci 112, 989–996. 10.1111/cas.14791.

[S352] Hosoya, N., Okajima, M., Kinomura, A., Fujii, Y., Hiyama, T., Sun, J., Tashiro, S., and Miyagawa, K. (2011). Synaptonemal complex protein SYCP3 impairs mitotic recombination by interfering with BRCA2. EMBO Rep 13, 44–51. 10.1038/embor.2011.221.

[S353] Kang, T.H., Noh, K.H., Kim, J.H., Bae, H.C., Lin, K.Y., Monie, A., Pai, S.I., Hung, C.F., Wu, T.C., and Kim, T.W. (2010). Ectopic expression of X-linked lymphocyte-regulated protein pM1 renders tumor cells resistant to antitumor immunity. Cancer Res 70, 3062–3070. 10.1158/0008-5472.CAN-09-3856.

[S354] Kitano, H., Chung, J.Y., Noh, K.H., Lee, Y.H., Kim, T.W., Lee, S.H., Eo, S.H., Cho, H.J., Choi, C.H., Inoue, S., et al. (2017). Synaptonemal complex protein 3 is associated with lymphangiogenesis in non-small cell lung cancer patients with lymph node metastasis. J Transl Med 15, 138. 10.1186/s12967-017-1241-5.

[S355] Mobasheri, M.B., Jahanzad, I., Mohagheghi, M.A., Aarabi, M., Farzan, S., and Modarressi, M.H. (2007). Expression of two testis-specific genes, TSGA10 and SYCP3, in different cancers regarding to their pathological features. Cancer Detect Prev 31, 296–302. 10.1016/j.cdp.2007.05.002.

[S356] Niemeyer, P., Tureci, O., Eberle, T., Graf, N., Pfreundschuh, M., and Sahin, U. (2003). Expression of serologically identified tumor antigens in acute leukemias. Leuk Res 27, 655–660. 10.1016/s0145-2126(02)00230-8.

[S357] Oh, S.J., Cho, H., Kim, S., Noh, K.H., Song, K.-H., Lee, H.-J., Woo, S.R., Kim, S., Choi, C.H., Chung, J.-Y., et al. (2018). Targeting Cyclin D-CDK4/6 Sensitizes Immune-Refractory Cancer by Blocking the SCP3–NANOG Axis. Cancer Res 78, 2638–2653. 10.1158/0008-5472.CAN-17-2325.

[S358] Oh, S.J., Noh, K.H., Song, K.-H., and Kim, T.W. (2021). Interaction between SCP3 and JAB1 Confers Cancer Therapeutic Resistance and Stem-like Properties through EGF Expression. Int J Mol Sci 22, 8839. 10.3390/ijms22168839.
[S359] Yuan, L., Liu, J.G., Zhao, J., Brundell, E., Daneholt, B., and Höög, C. (2000). The murine SCP3 gene is required for synaptonemal complex assembly, chromosome synapsis, and male fertility. Mol Cell 5, 73–83. 10.1016/s1097-2765(00)80404-9.
[S360] Sandhu, S., Sou, I.F., Hunter, J.E., Salmon, L., Wilson, C.L., Perkins, N.D., Hunter, N., Davies, O.R., and McClurg, U.L. (2021). Centrosome dysfunction associated with somatic expression of the synaptonemal complex protein TEX12. Commun Biol 4, 1371. 10.1038/s42003-021-02887-4.
[S361] Zhou, H., Wu, L., Yu, L., Yang, Y., Kong, L., Liu, S., Chen, W., and Li, R. (2022). Identify a DNA Damage Repair Gene Signature for Predicting Prognosis and Immunotherapy Response in Cervical Squamous Cell Carcinoma. J Oncol 2022, 8736575. 10.1155/2022/8736575.
[S362] Lin, X., Chen, Z., Gao, P., Gao, Z., Chen, H., Qi, J., Liu, F., Ye, D., Jiang, H., Na, R., et al. (2017). TEX15: A DNA repair gene associated with prostate cancer risk in Han Chinese. Prostate 77, 1271–1278. 10.1002/pros.23387.
[S363] Mantere, T., Tervasmaki, A., Nurmi, A., Rapakko, K., Kauppila, S., Tang, J., Schleutker, J., Kallioniemi, A., Hartikainen, J.M., Mannermaa, A., et al. (2017). Case-control analysis of truncating mutations in DNA damage response genes connects TEX15 and FANCD2 with hereditary breast cancer susceptibility. Sci Rep 7, 681. 10.1038/s41598-017-00766-9.
[S364] Okutman, O., Muller, J., Baert, Y., Serdarogullari, M., Gultomruk, M., Piton, A., Rombaut, C., Benkhalifa, M., Teletin, M., Skory, V., et al. (2015). Exome sequencing reveals a nonsense mutation in TEX15 causing spermatogenic failure in a Turkish family. Hum Mol Genet 24, 5581–5588. 10.1093/hmg/ddv290.
[S365] Yang, F., Eckardt, S., Leu, N.A., McLaughlin, K.J., and Wang, P.J. (2008). Mouse TEX15 is essential for DNA double-strand break repair and chromosomal synapsis during male meiosis. J Cell Biol 180, 673–679. 10.1083/jcb.200709057.
[S366] Huang, J., Wang, Y., Liu, J., Chu, M., and Wang, Y. (2021). TFDP3 as E2F Unique Partner, Has Crucial Roles in Cancer Cells and Testis. Front Oncol 11, 742462. 10.3389/fonc.2021.742462.
[S367] Ingram, L., Munro, S., Coutts, A.S., and La Thangue, N.B. (2011). E2F-1 regulation by an unusual DNA damage-responsive DP partner subunit. Cell Death Differ 18, 122–132. 10.1038/cdd.2010.70.
[S368] Jiao, Y., Ding, L., Chu, M., Wang, T., Kang, J., Zhao, X., Li, H., Chen, X., Gao, Z., Gao, L., et al. (2017). Effects of cancer-testis antigen, TFDP3, on cell cycle regulation and its mechanism in L-02 and HepG2 cell lines in vitro. PLoS One 12, e0182781. 10.1371/journal.pone.0182781.
[S369] Ma, Y., Xin, Y., Li, R., Wang, Z., Yue, Q., Xiao, F., and Hao, X. (2014). TFDP3 was expressed in coordination with E2F1 to inhibit E2F1-mediated apoptosis in prostate cancer. Gene 537, 253–259. 10.1016/j.gene.2013.12.051.
[S370] Qiao, H., Di Stefano, L., Tian, C., Li, Y.Y., Yin, Y.H., Qian, X.P., Pang, X.W., Li, Y., McNutt, M.A., Helin, K., et al. (2007). Human TFDP3, a novel DP protein, inhibits DNA binding and transactivation by E2F. J Biol Chem 282, 454–466. 10.1074/jbc.M606169200.
[S371] Tian, C., Lv, D., Qiao, H., Zhang, J., Yin, Y.H., Qian, X.P., Wang, Y.P., Zhang, Y., and Chen, W.F. (2007). TFDP3 inhibits E2F1-induced, p53-mediated apoptosis. Biochem Biophys Res Commun 361, 20–25. 10.1016/j.bbrc.2007.06.128.
[S372] Wang, X., Xing, Z., Xu, H., Yang, H., and Xing, T. (2021). Development and validation of epithelial mesenchymal transition-related prognostic model for hepatocellular carcinoma. Aging (Albany NY) 13, 13822–13845. 10.18632/aging.202976.
[S373] Zhang, D., Zhou, S., and Liu, B. (2020). Identification and Validation of an Individualized EMT-Related Prognostic Risk Score Formula in Gastric Adenocarcinoma Patients. Biomed Res Int 2020, 7082408. 10.1155/2020/7082408.
