# Supplementary material for: Cancer testis antigens: Emerging therapeutic targets leveraging genomic instability in cancer
Source: Mol Ther Oncol. 2024 Jan 26;32(1):200768. doi: 10.1016/j.omton.2024.200768 (PMC10876628; doi:10.1016/j.omton.2024.200768)
Supplement: Document S2. Article plus supplemental information [file mmc3.pdf]

Molecular Therapy
Oncology
Review

Cancer testis antigens: Emerging therapeutic targets leveraging genomic instability in cancer

Adviti Naik,[1,3] Boucif Lattab,[1] Hanan Qasem,[1,2] and Julie Decock[1,2]

[1]Cancer Research Center, Qatar Biomedical Research Institute (QBRI), Hamad Bin Khalifa University (HBKU), Qatar Foundation, Doha, Qatar; [2]College of Health and Life Sciences (CHLS), Qatar Biomedical Research Institute (QBRI), Hamad Bin Khalifa University (HBKU), Doha, Qatar

Cancer care has witnessed remarkable progress in recent decades, with a wide array of targeted therapies and immune-based interventions being added to the traditional treatment options such as surgery, chemotherapy, and radiotherapy. However, despite these advancements, the challenge of achieving high tumor specificity while minimizing adverse side effects continues to dictate the benefit-risk balance of cancer therapy, guiding clinical decision making. As such, the targeting of cancer testis antigens (CTAs) offers exciting new opportunities for therapeutic intervention of cancer since they display highly tumor specific expression patterns, natural immunogenicity and play pivotal roles in various biological processes that are critical for tumor cellular fitness. In this review, we delve deeper into how CTAs contribute to the regulation and maintenance of genomic integrity in cancer, and how these mechanisms can be exploited to specifically target and eradicate tumor cells. We review the current clinical trials targeting aforementioned CTAs, highlight promising pre-clinical data and discuss current challenges and future perspectives for future development of CTA-based strategies that exploit tumor genomic instability.

INTRODUCTION

Cancer-testis antigens (CTA) are a large family of tumor-associated proteins that under physiological conditions are predominantly expressed in the testes, specifically in the proliferating germ cells, spermatogonia and spermatocytes.[1–7] Their expression is tightly controlled by DNA methylation and histone modifications involving epigenetic modulatory proteins such as the germ-cell specific CCCTC-binding factor (CTCF) and Brother of Regulator of Imprinted Sites (BORIS).[4,8–11] Furthermore, aberrant expression of CTAs in tumors may depend on chromosomal location since chromosome X-encoded CTAs, encompassing the majority of multigene CTA families (MAGE/GAGE/PAGE/XAGE, NY-ESO-1 and SSX genes), are more frequently expressed in tumors compared to autosomal single-copy CTA genes (non-CT-X).[12] While CTA expression is a recurrent observation in tumors, the extent of their expression differs between cancers as well as between tumors of the same cancer type.[13–15] Based on the frequency of CTA expression, cancers can be grouped into CTA-rich and -poor subgroups.[16] CTA-rich cancers include melanoma, lung cancer, hepatocellular carcinoma, germ cell cancer, gastric cancer, and chondrosarcoma with a CTA expression frequency of at least 50%. On the other hand, leukemia, lymphoma, renal carcinoma, glioblastoma, and colon carcinoma constitute CTA-poor cancer types with expression frequencies of less than 20%. Interestingly, da Silva et al. reported that 17% of all CTAs show exclusive expression in a single tumor type.[14] For instance, they identified 32 CTAs that were expressed solely in leukemia, eleven in melanoma, and fourteen in ovarian cancer; thus, suggesting tumor-type specific roles for CTAs. In addition, CTA expression shows heterogeneous patterns within a single cancer type. For example, in breast cancer, the CTAs *CXorf61*, *HORMAD1*, *ACTL8* and *PRAME* are specifically enriched in basal subtypes, while the expression of *PLAC1* and *POTEC* is more frequently observed in non-basal subtypes.[15]

Overall, CTA expression has been associated with worse clinical outcome.[5,8,12] For example, LDHC expression has been correlated with poor prognosis in breast cancer,[17] lung cancer,[18] renal cell carcinoma[19] and hepatocellular carcinoma.[20] In contrast, few CTAs have been correlated with a better prognosis such as ACTL8, OIP5, XAGE3 and CTCFL in glioblastoma.[21] Finally, a select few show a differential prognostic value based on the tumor type; for instance, NY-ESO-1 is associated with favorable prognosis in melanoma but poor outcome in other tumor types.[5] These divergent findings suggest that CTAs exhibit pro- and anti-tumorigenic functions in a context-dependent manner. To date, sparse information is available on anti-tumorigenic functions of CTAs with few studies reporting that TSAG10, RGS22, MAGE-A4 and SPANXA restrict tumorigenesis through the inhibition of tumor metabolic activity, proliferation and metastasis.[22–26] Traditionally, CTAs are thought to predominantly support cancer hallmarks such as sustaining proliferative signaling, resisting cell death, deregulating cellular energetics, activating invasion and metastasis, inducing angiogenesis, and genome instability and mutation.[5–8,12,27,28] For instance, members of the MAGE family have been shown to, in part through binding of the master tumor suppressor p53, promote tumor cell proliferation and

https://doi.org/10.1016/j.omton.2024.200768.

[3]Present address: Biological Sciences, Carnegie Mellon University- Qatar, Doha, Qatar

Correspondence: Julie Decock, PhD, Cancer Research Center, Qatar Biomedical Research Institute (QBRI), Hamad Bin Khalifa University (HBKU), Qatar Foundation, Doha, Qatar.
E-mail: jdecock@hbku.edu.qa

This is an open access article under the CC BY license (http://creativecommons.org/licenses/by/4.0/).

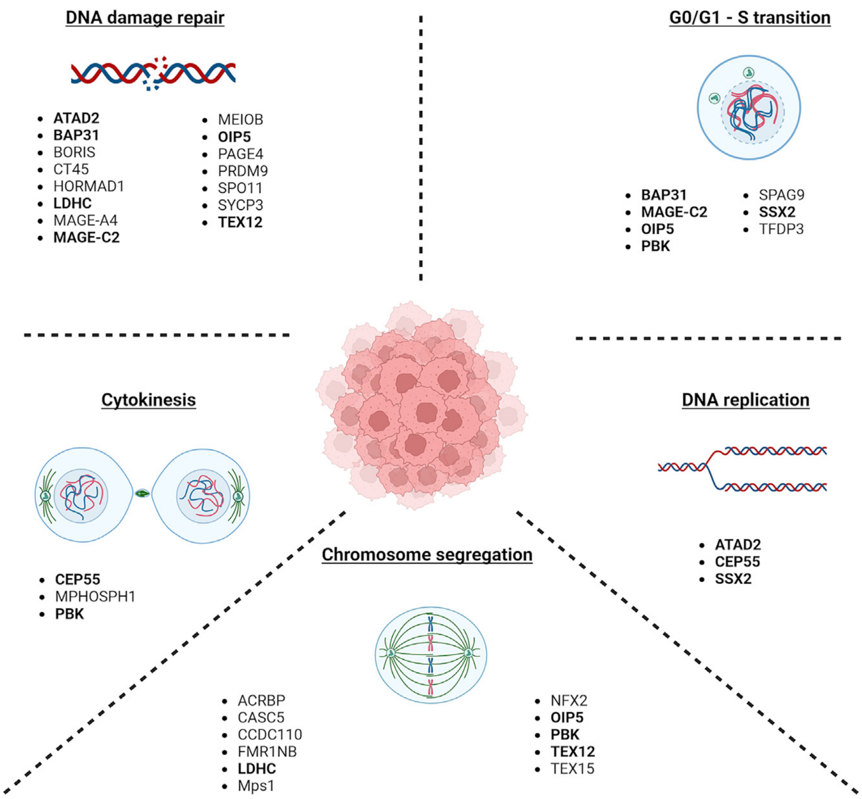

Figure 1. Diverse roles of cancer testis antigens in biological processes that regulate genomic integrity in cancer

Tumoral expression of distinct CTAs impacts tumor cellular fitness through their role in DNA damage repair, G0/G1-S cell cycle transition, DNA replication, chromosome segregation and cytokinesis. CTAs highlihgted in bold exert multiple regulatory functions.

cell cycle progression while inhibiting tumor cell survival.[29,30] SSX and CAGE family members increase tumor cell growth and survival through the activation of the MAPK and Wnt signaling pathways and upregulation of cell cycle proteins.[31–33] CTAs have also been implicated in blocking cellular senescence programs, allowing cancer cells to bypass several checkpoints that are crucial to suppress tumorigenesis, and promoting epithelial-mesenchymal transition, leading to an increased migratory and invasive potential of cancer cells.[34–36] A growing body of evidence further indicates that expression of CTAs in tumors affects tumor cellular fitness by enhancing genomic instability. Indeed, genomic integrity is a critical characteristic of both tumor cells and germ cells which has a profound impact on the transmission of error-free hereditary information and defines cellular fitness.[37,38]

Given their highly tumor specific expression and pro-tumorigenic functions, CTA-based therapy provides novel opportunities for potent treatment responses with minimal adverse effects. This review specifically highlights the current knowledge on CTAs that play a role in regulating genomic integrity and discusses current and emerging therapeutic approaches targeting these specific CTAs to improve the clinical outcome of cancer patients.

GENOMIC INTEGRITY-REGULATORY CTAS

Genomic instability is a major driving force of tumorigenesis and tumor progression as it leads to the further accumulation of genomic and chromosomal alterations that promote the acquisition of tumor features and cancer hallmarks. This increased propensity for genomic alterations often results from DNA damage introduced by extrinsic, environmental (mutagenic chemical agents, ultraviolet radiation) or intrinsic factors (replication errors, oxidative stress, spontaneous hydrolysis). As such, normal cells utilize various mechanisms to minimize transmission of genetic errors to daughter cells.[39,40] Central to these is the tight regulation of cell division whereby cell cycle checkpoints either induce cell-cycle arrest to promote repair, or trigger apoptosis or senescence in case of excessive damage. Aberrant expression of CTAs has been shown to dysregulate cell cycle surveillance, leading to enhanced genomic instability. This review provides a comprehensive overview of the expression, clinical relevance and molecular function of CTAs that have been implicated in regulating genomic integrity through dysregulation of DNA damage repair, G0/G1-S phase transition, DNA replication, chromosome segregation and cytokinesis (Figure 1; Table S1). A few CTAs, highlighted in bold, play a role in multiple regulatory mechanisms and are discussed separately at the end of this section.

DNA damage repair

DNA damage repair is critical to cell cycle progression of normal and malignant cells and can be triggered at several points during the cell cycle by DNA damage checkpoints. The G1 checkpoint ensures that cells do not undergo replication until DNA damage is repaired, while the S and G2 checkpoints prevent cells with damaged DNA from undergoing mitosis. Of note, there is a fine balance between the tolerable levels of DNA damage that drive oncogenic transformation and excessive levels that ultimately induce cancer cell senescence or cell death. Aberrant tumor expression of CTAs has been found to differentially regulate genomic integrity in a context-dependent manner and is thus associated with either a better or worse clinical outcome in different cancers. Mechanistically, CTAs have been shown on one hand to impair DNA damage checkpoints, resulting in the propagation of cells with unresolved DNA damage and enhancing genomic instability, and on the other hand to promote the DNA damage response in order to enhance tumor cellular fitness. Therefore, caution is warranted when targeting these particular CTAs for which the tumor context should be taken into consideration. When

favorable, targeting of CTAs could be combined with DNA damage response-related drugs to improve treatment response and clinical outcome. We recently provided experimental evidence to support this approach, whereby silencing of LDHC greatly sensitized breast cancer cells to treatment with cisplatin and olaparib.[17]

BORIS/CTCFL/CT27

Brother of the Regulator of Imprinted Sites (BORIS) is abnormally expressed in lung, breast, hepatocellular and cervical carcinoma and is associated with worse prognosis. The aberrant expression of BORIS in cancer cells supports tumor progression through enhanced cell proliferation, cell survival and DNA damage repair. Its expression has been shown to promote colorectal cancer cell proliferation, reduce apoptosis and induce fluorouracil (5-FU) resistance.[41] In accordance, depletion of BORIS in a colorectal cancer mouse model suppressed DNA damage repair and promoted apoptosis.[42] Furthermore, overexpression of BORIS in non-small cell lung cancer cell lines was shown to suppress cisplatin-induced DNA damage, likely through upregulation of the mismatch repair factor mutS homolog 6 (MSH6).[43] Conversely, silencing of BORIS increased DNA damage, inhibited cell proliferation and enhanced treatment response.

CT45/CT45A1

Cancer testis antigen 45 (CT45) is highly expressed in ovarian cancer, lung cancer, endometrial cancer and to a lesser extent in breast cancer and is correlated with poor prognosis. In high-grade serous ovarian cancer, CT45 has been implicated in the DNA damage response through its inhibition of the PP4 phosphatase complex, leading to increased DNA damage and worse clinical outcome.[44] On the other hand, this rise in genomic instability can be exploited for therapeutic purposes in combination with platinum-based chemotherapy, exacerbating DNA damage and inducing cell death followed by antigen release and T cell activation. In contrast, CT45 expression in endometrial cancer has been linked to cancer cell stemness and paclitaxel resistance through its co-expression and upregulation by Y-Box binding proteins (YBX2).[45]

HORMAD1/NOHMA/CT46

HORMA domain-containing protein 1 (HORMAD1) was identified as the first meiotic checkpoint gene that is essential for double strand break-dependent homologous recombination and accurate chromosome segregation,.[46] Overall, HORMAD1 expression is upregulated in cancer, including gastric cancer, lung cancer, breast cancer and ovarian cancer, and has been associated with increased genomic instability and worse overall survival. Notably, HORMAD1 can regulate DNA double-strand break repair in cancer cells through either homologous recombination or non-homologous end-joining. In lung cancer, HORMAD1 promotes homologous recombination to mitigate DNA damage and protect stalled replication forks from excessive MRE1-mediated nucleolytic degradation, while loss of HORMAD1 significantly reduces tumor growth *in vivo* and enhances sensitivity to irradiation and PARP inhibition.[47,48] In contrast, in triple negative breast cancer, HORMAD1 suppresses homologous recombination and induces non-homologues end joining repair, hence sensitizing cancer cells to the use of homologous recombination-targeting therapy such as platinum-based chemotherapy.[49,50] In addition, HORMAD1 compromises DNA mismatch repair of single strand breaks through cytosolic retention of the MCM8–MCM9 complex and reduction of MLH1 chromatin binding.[51] HORMAD1 also enhances DNA damage tolerance resulting in increased dependency on replication stress tolerance pathways, such as *trans*-lesion synthesis, and thus induces resistance to chemotherapeutic drugs such as docetaxel.[52,53] Furthermore, silencing of HORMAD improves sensitivity to cisplatin treatment.[54]

MAGE-A4/CT1.4

Melanoma-Associated Antigen 4 (MAGE-A4) is a member of the melanoma-associated antigen (MAGE) family of proteins and is expressed in lung cancer, breast cancer, bladder cancer, hepatocellular carcinoma, esophageal cancer, oral squamous cell carcinoma, gastrointestinal stromal tumors and gastric cancer, urothelial carcinoma, colorectal cancer, desmoid tumors, osteosarcoma, soft tissue sarcoma, synovial sarcoma, T cell leukemia/lymphoma, endometrial cancer, cervical cancer, ovarian cancer, vulvar cancer, uterine carcinosarcoma, salivary gland tumors, melanoma, and head and neck cancer. Overall, MAGE-A4 expression is associated with a poor prognosis, except for breast cancer and salivary gland carcinoma where it is linked to a favorable prognosis. In analogy with HORMAD1, MAGE-A4 has been shown to enhance DNA damage tolerance, in particular through binding and stabilization of the E3 ubiquitin ligase RAD18, resulting in enhanced PCNA mono-ubiquitination and subsequent activation of *trans*-lesion synthesis, one of the main effector pathways of DNA damage response.[55] Oral squamous cell carcinoma cell lines expressing MAGE-A4 demonstrated lower treatment responses to docetaxel and paclitaxel.[56]

MEIOB/SPGF22

Meiosis Specific With OB-Fold (MEIOB) is a single strand DNA-binding protein that is vital for homologous recombination and faithful chromosome segregation during meiosis.[57] MEIOB expression is positively correlated with the copy number aberrations in lung adenocarcinoma, bladder urothelial carcinoma, thyroid carcinoma, and uterine corpus endometrial carcinoma and its expression positively correlates with poor survival in triple negative breast cancer.[13] In lung adenocarcinoma, MEIOB overexpression increases cell viability, proliferation, and the proportion of cells in the G2 phase.[13] In contrast to its role in meiosis, MEIOB was shown to drive the error-prone non-homologous end-joining DNA repair mechanism while inducing homologous recombination deficiency, rendering cancer cells and xenografts more sensitive to treatment with PARP inhibitors.[58]

PAGE4/GAGEC1/CT16.7

Prostate Associated Gene 4 protein (PAGE4) is a transcription regulator of the c-Jun/AP-1/Fos signaling pathway that potentiates the transcription of prostate gland development genes.[59] Expression of PAGE4 is upregulated in primary prostate tumors and was found to be associated with a reduced risk of prostate cancer recurrence

and a favorable prognosis. Further, PAGE expression has been shown to attenuate androgen receptor signaling which likely impedes tumor progression to advanced stages.[60] In contrast, in colorectal cancer, PAGE expression was shown to be significantly higher in primary tumors with liver metastasis.[61] Under oxidative stress conditions, PAGE4 has been shown to protect prostate cancer cells from reactive oxygen species-induced DNA damage and apoptosis.[62,63]

PRDM9/PFM6

PR/SET Domain 9 (PRDM9) is a zinc finger histone methyltransferase that marks the localization of meiotic recombination hotspots.[64] Expression of PRDM9 in tumors has been suggested to increase DNA double-strand breaks and genomic instability due to the enrichment of structural variant breakpoints and intersecting chromosome loop anchor points at sites of PRDM9 activity.[65,66] PRDM9 is overexpressed in several cancers, including head and neck squamous cell carcinoma, bladder urothelial carcinoma, liver cancer and ovarian cancer. Moreover, rare allelic forms of PRDM9 have been reported in aneuploid and childhood B-cell precursor acute lymphoblastic leukemia.[67]

SPO11/CT35

SPO11 initiator of meiotic double-stranded breaks (SPO11) is a meiosis-specific endonuclease that catalyzes the formation of DNA double-strand breaks to initiate meiotic recombination.[68] Disruption of SPO11 in murine spermatocytes results in defective meiosis, apoptosis and hence, severe gonadal abnormalities.[69] SPO11 expression has been reported in cutaneous T cell lymphoma, colorectal cancer, melanoma, and cervical cancer. High expression of SPO11 in conjunction with EME2, MSH2 and MLH3 has been associated with worse prognosis in metastatic colorectal cancer. Although the molecular function of SPO11 in tumors has not yet been reported, it is likely that its role in double-strand break formation enhances genomic instability in cancer cells.

SYCP3/SCP3

Synaptonemal Complex Protein 3 (SYCP3) is a vital structural component of the synaptonemal complex that is formed between homologous chromosomes during meiosis.[70] Expression of SYCP3 has been reported in various cancers including cervical cancer, acute lymphoblastic leukemia, non-small cell lung cancer, ovarian cancer and astrocytoma and is associated with shorter overall survival. SYCP3 expression has been linked to immune resistance and enhanced cancer stemness through Akt-mediated upregulation of anti-apoptotic molecules and Nanog.[71,72] Sparse information is available on the role of SYCP3 in tumor genomic integrity except that is binds to BRCA2, which subsequently inhibits RAD51-dependent homologous recombination and confers hypersensitivity of cancer cells to PARP inhibition.[73]

G0/G1-S cell cycle transition

As previously mentioned, transition from the G0/G1 to the S phase is tightly controlled by the G1 checkpoint to enable DNA damage repair prior to DNA replication. Inhibitors against the G1-S cyclin-dependent kinases CDK4/6 have shown potent anti-tumor activity in metastatic hormone receptor positive breast cancer, non-small-cell lung cancer, prostate cancer and acute myeloid leukemia while modest efficacy was observed in triple negative cancer, colorectal cancer and melanoma.[74] Drug resistance poses a major challenge for CDK4/6 inhibitors and is often the result of compensatory mechanisms. Hence, it is imperative to advance our understanding of the molecular networks associated with the expression of CTAs that play a role in G1-S checkpoint regulation in order to inform the development of combination therapeutic approaches.

SPAG9/JIP-4/CT89

Sperm-associated antigen 9 (SPAG9) is aberrantly expressed in multiple cancers such as hepatocellular carcinoma, chronic myeloid leukemia, thyroid cancer, bladder cancer, endometrial cancer, gastric cancer, prostate cancer, non-melanoma skin cancer, osteosarcoma, salivary gland tumors and astrocytoma. Its expression has been correlated with poor prognosis in breast cancer, non-small cell lung cancer, gastric cancer, prostate cancer, and hepatocellular carcinoma. In contrast, SPAG9 expression in clear-cell renal cell carcinoma predicted a better overall survival where it was shown to promote autophagy and inhibit inflammatory responses.[75] Furthermore, SPAG9 deficient mouse models demonstrated that SPAG9 plays a key role in regulating CD4+ T cells response to TCR stimulation.[76] Silencing of SPAG9 has been shown to arrest cancer cells in G0/G1 or S phase and decrease expression of cyclins and cyclin-dependent kinases.[77–83] SPAG9 silencing in combination with paclitaxel treatment synergistically inhibited ovarian cancer cell viability, and SPAG9 depletion in ovarian cancer xenograft mouse models significantly reduced tumor growth.[81]

TFDP3/DP4/HCA661/CT30

Transcription Factor Dimerization Partner 3 (TFDP3) is a E2F partner protein that, in contrast to other DP family members, downregulates E2F activity in response to DNA damage, inhibiting E2F-transcriptional activity, and E2F1-induced induced apoptosis and G1-S cell cycle progression.[84–88] TFDP3 expression has been reported in prostate cancer, pancreatic cancer, breast cancer, hepatocellular carcinoma and gastric adenocarcinoma, the latter where it is associated with worse survival.

DNA replication

Defects in replication due to replication stress such as altered replication fork progression, reduced replication fidelity and DNA breaks, can lead to genomic and chromosomal instability. Currently, three CTAs have been implicated in the regulation of DNA replication; ATAD2, CEP55 and SSX2. Each of these plays added roles in DNA damage repair, G0/G1-S cell cycle transition or cytokinesis and will be discussed in the section on multifunctional genomic integrity-regulatory CTAs.

Chromosome segregation

Inaccurate chromosome segregation poses a great threat to genomic integrity as it can result in chromosome copy number alterations

(aneuploidy, polyploidy), the formation of micronuclei and chromosomal structural aberrations. Anti-mitotic drugs have been used to treat multiple cancer types by interfering with microtubule stabilization, spindle formation, chromosome segregation and mitotic exit, driving cells toward mitotic arrest.[89] While a proportion of arrested cells will undergo cell death, others may survive through mitotic slippage, eventually becoming genomically unstable or senescent, negatively affecting treatment response. Combination treatment of anti-mitotic drugs with distinct CTAs may help to improve treatment efficacy through further dysregulation of chromosome segregation and the spindle assembly checkpoint. For instance, a phase I study on combination treatment of taxanes with inhibitors targeting Mps1 demonstrated an anti-tumor activity in 32% of patients with solid tumors, establishing a precedent for further clinical trials.[90]

ACRBP/OY-TES-1/CT23

Acrosin binding protein (ACRBP) is a CTA that is abnormally expressed in tumors of many different tissue origins including the breast, bladder, colon, liver, lung, and ovaries and can induce specific cytotoxic cellular and humoral immune responses. High ACRBP expression in ovarian cancer correlates with reduced survival, earlier relapse and paclitaxel resistance.[91] Molecularly, ACRBP tumor expression was found to be required for robust mitotic spindle assembly and function through its antagonistic interaction with the spindle-associated protein NuMa. As such, depletion of ACRBP in ovarian cancer cells treated with paclitaxel resulted in elevated NuMa levels, activating the spindle assembly checkpoint response, and leading to mitotic delay, mitotic catastrophe and subsequently enhanced treatment response.

CASC5/KNL1/KIAA1570/CT29

Cancer Susceptibility Candidate gene 5 protein (CASC5) is a scaffolding protein that is involved in kinetochore assembly, kinetochore-microtubule attachment, and chromosome segregation during mitosis.[92,93] Pan-cancer analysis revealed aberrant CASC5 expression in papillary renal cell carcinoma, lung adenocarcinoma, pancreatic adenocarcinoma, thymoma and urinary bladder cancer. Further, high CASC5 expression is associated with advanced disease and poor overall survival in lung adenocarcinoma, with cell line models demonstrating reduced cancer cell proliferation upon silencing of CASC5.[94] On the other hand, CASC5 expression is reduced in leukemia patients and is associated with overexpression of genes related to chemoresistance and disease progression.[95]

CCDC110/KM-HN-1/CT52

Coiled-Coil Domain Containing 110 (CCDC110) is expressed in numerous cancers including esophageal cancer, breast cancer, colon cancer, melanoma, hepatocellular carcinoma, gastric cancer, and pancreatic cancer. CCDC110 expression is localized at centrosomes during mitosis, supporting a role for CCDC110 in G2/M regulation and chromosome segregation.[96] Further, CCDC110 overexpression has been shown to delay G2/M phase transition in osteosarcoma cells.[97]

FMR1NB/NY-SAR-35/CT37

Fragile X Mental Retardation 1 Neighbor (FMR1NB) expression has been observed in sarcoma, melanoma, esophageal cancer, lung cancer and breast cancer and correlates with advanced grade and poor prognosis in glioma. It plays an important role in chromosome segregation and mitotic fidelity,[98] and its expression is regulated by promoter methylation.[99] Silencing of FMR1NB reduces cancer cell proliferation and motility, induces cell death, and improves sensitivity to paclitaxel treatment.[98,100–102]

Mps1/TTK/CT96

Monopolar spindle 1 kinase (Mps1) is a spindle assembly checkpoint kinase that delays mitotic exit from the anaphase and corrects erroneous attachments to prevent chromosome missegregation by facilitating the recruitment of key checkpoint proteins to kinetochores and regulating kinetochore-microtubule attachments.[103–105] As such, Mps1 inhibition selectively reduces cancer cell proliferation and increases cancer cell aneuploidy and cell death as a result of mitotic checkpoint override, chromosomal misalignment, destabilization of kinetochores and mitotic checkpoint complexes, accumulation of irreparable DNA damage and polyploidy.[104–114] Mps1 expression has been observed in multiple myeloma, osteosarcoma, hepatocellular carcinoma, neuroblastoma, glioma and glioblastoma, prostate cancer, colon cancer, lung cancer, mesothelioma, pancreatic cancer, and has been associated with poor prognosis in multiple cancers. In contrast, high expression of Mps1 in triple negative breast tumors was found to correlate with a better prognosis, however, the mechanisms driving this phenotype remain unexplored. The small molecule Mps1 inhibitors, commonly referred to as TTK inhibitors, CFI-402257, MPI-0479605 and NMS-P715 have been found to reduce tumor growth in hepatocellular carcinoma, lung cancer, ovarian cancer and melanoma xenograft models.[106–108,115] Further, a phase I study of the Mps1 inhibitor S81694 in 35 patients with advanced, metastatic solid tumors reported one patient with a complete response and 13 who had stable disease.[116] Given the role of Mps1 in regulating the mitotic checkpoint complex, inhibition of Mps1 potentiates the activity of taxanes in preclinical setting.[117–122] For instance, in prostate cancer cells Mps1 inhibition induced mitotic slippage and mitotic catastrophe of cancer cells that are undergoing prolonged mitotic block as a result of taxane-induced spindle assembly checkpoint activation.[119] Furthermore, inhibition of Mps1 in p53-mutant breast cancer cells enhanced taxane treatment response through activation of the p53-dependent postmitotic spindle checkpoint as a result of loss of Mps1-mediated phosphorylation of p53 and MDM2-mediated p53 ubiquitination.[123–125] Mps1 inhibition has also been shown to enhance radiotherapy response of glioblastoma through modulation of the activity of DNA damage repair pathways.[126]

NXF2/TAPL2/CT39

Nuclear RNA Export Factor 2 (NXF2) is well known as a key regulator of spermatogonial proliferation, stem cell population maintenance and meiotic progression.[127] NXF2 expression has been found in esophageal squamous cell carcinoma, head and neck squamous cell carcinoma, seminomas, and estrogen receptor negative breast

cancer. Although little is known about the role of NFX2 in cancer, a paclitaxel synthetic lethality screen identified NFX2 to be critical for accurate chromosome segregation in tumor cells.[98]

TEX15/SPGF25/CT42

Testis-expressed protein 15 (TEX15) plays a key role in the formation of the chromosomal synapsis during male meiosis where it regulates the localization of SYCP1, RAD51 and DMC1 to the synaptonemal complex synapse.[128] In line with this, a nonsense mutation in TEX15, resulting in a truncated form of the protein, has been reported as causal in a familial infertility phenotype.[129] In addition, two deleterious TEX15 genetic variants, Q1631H and c.7253dupT, have been associated with a higher risk of developing prostate and breast cancer respectively, further supporting a role for TEX15 in tumorigenesis.[130,131]

Cytokinesis

Faithful transmission of chromosomes to daughter cells is dependent on the accurate segregation of replicated chromosomes to opposite spindle poles followed by the formation of two daughter cells during cytokinesis. Dysregulation of cytokinesis by CTAs may therefore increase genomic instability and could be used in combination with anti-mitotic drugs to compromise tumor cell survival.

MPHOSPH1/KIF20B/KRMP1/CT90

High expression of M-phase Phosphoprotein 1 (MPHOSPH1) has been observed in testicular germ cell cancer, bladder cancer, pancreatic cancer, hepatocellular carcinoma, colorectal cancer, oral cancer, renal cell carcinoma and breast cancer, where its expression directly correlates with poor prognosis. Silencing of MPHOSPH1 reduces proliferation and tumor growth, attenuates distant lung metastasis and improves sensitivity to taxol treatment.[132–135] Mechanistically, MPHOSPH1 directly interacts with the protein-regulating cytokinesis 1 (PRC1) protein, translocating it along the mitotic spindles during mitosis and promoting cytokinesis.[136] In line with this, knockdown of MPHOSPH1 induces aberrant cytokinesis, resulting in the formation of multinucleated cells and increased cell death.[136–138]

Multifunctional genomic integrity-regulatory CTAS

Several CTAs, discussed below in alphabetical order, are implicated in the regulation of multiple genomic integrity surveillance mechanisms, thus, augmenting the implications of CTA therapeutic intervention.

ATAD2/ANCCA/CT137

ATPase Family AAA Domain Containing 2 (ATAD2) is frequently expressed in breast, lung, colorectal, liver, gastric, oral, ovarian, cervical, and endometrial tumors, and correlates with disease severity and poor prognosis. ATAD2 expression can be regulated by the oncogenic E2F transcription factor and in turn binds and activates the MYC transcription factor, thus driving cancer progression.[139] In colorectal cancer, the oncogenic ubiquitin E3 ligase TRIM25 binds and stabilizes ATAD2 expression following genotoxic stress, which induces a positive ATAD2-E2F-TRIM25 feedback loop whereby ATAD2 acts as a transcriptional co-activator of E2Fs to promote TRIM25 expression.[140] ATAD2 has been shown to act as a chromatin regulator and to play a role in DNA replication during the S phase by enabling the assembly of histone-modifying protein complexes at target gene chromatin loci.[141,142] In addition, Duan et al. reported that ATAD2 is involved in the regulation of the expression and activation of BRCA1, Chk1 and Chk2 in response to DNA damaging anti-cancer drugs, suggesting that ATAD2 is a key mediator of the DNA damage response and repair mechanism.[143] In accordance, knockdown of ATAD2 sensitizes triple negative breast cancer cells to DNA-damaging agents such as carboplatin[143] and pancreatic cancer cells to gemcitabine-radiation combination treatment.[144]

BAP31/BCAP31

Aberrant expression of B cell receptor Associated Protein 31 (BAP31) can be observed in cervical cancer, colorectal cancer, hepatocellular carcinoma, ovarian cancer, and gastric cancer. In cervical cancer, breast cancer and non-small lung cancer, its expression is positively correlated with poor prognosis, whereas the opposite is true in colorectal cancer and hepatocellular carcinoma. These divergent associations of BAP1 expression with prognosis are likely the result of dual functionality. BAP31 is an endoplasmic reticulum chaperone and regulates apoptosis through its interaction with Bcl and caspase proteins.[145,146] It can be cleaved by caspase-8 into a p20 fragment, p20BAP31, which transmits proapoptotic signals between the endoplasmic reticulum and mitochondria,[147] and increases ROS production,[148] promoting the induction of DNA damage and cell-cycle arrest. On the other hand, BAP31 was found to regulate the proteasomal degradation of the Cdk inhibitor p27kip1, thereby directly promoting G1/S cell cycle progression.[149] In line with this finding, depletion of BAP31 has been shown to result in G0/G1 cell-cycle arrest, aberrant cytoskeletal assembly, reduced cancer cell motility, increased apoptosis and reduced tumor progression in xenografts models.[150–154] Conversely, BAP31 overexpression is associated with increased tumor cell proliferation, colony formation and tumor growth in *in vitro* and xenograft models respectively.[155] In cervical cancer, BAP31 expression is regulated by microRNA-362 (miR-362), inhibiting BAP31-mediated activation of the TGFβ/Smad pathway,[156] which may in part explain the miR-362 associated reduction in cancer cell proliferation and induction of apoptosis.

CEP55/URCC6/CT111

Centrosomal Protein 55 (CEP55) is a key regulator of cytokinesis and has been included in a 70-gene chromosomal instability signature associated with aneuploidy in cancer.[157–159] Increased CEP55 expression can be found in several cancers including endometrial cancer, bladder cancer, colorectal cancer, liver cancer, non-small cell lung cancer, renal cell carcinoma, cervical cancer, and esophageal squamous cell carcinoma and is generally associated with poor prognosis. Furthermore, overexpression of CEP55 in cervical cancer is associated with increased risk of lymph node metastasis and tumor progression. In breast cancer, CEP55 expression has been linked to docetaxel resistance.[160] Conversely, knockdown of CEP55 expression reduces breast cancer cell proliferation, and following mitotic arrest by anti-mitotic drugs CEP55 increases cell death while reducing mitotic

slippage.[160,161] In addition, CEP55 has been shown to promote aberrant mitosis in mouse embryonic fibroblasts through hyperactivation of the PI3K/Akt pathway and a defective S-phase checkpoint, resulting in increased DNA replication, excess DNA damage and microtubule stabilization.[158]

LDHC/LDHX/LDH3/CT32

Lactate dehydrogenase C (LDHC) is a metabolic enzyme that plays a critical role in sperm motility, capacitation, and fertilization.[162–164] Its expression has been associated with poor prognosis when detected in tumor tissues, cancer patients' serum and serum-derived exosomes. In few cancers such as head and neck squamous cell carcinoma, and cervical squamous cell carcinoma and endocervical adenocarcinoma LDHC expression is associated with favorable prognosis.[165] In addition to the full length protein, four splice variants with structural alterations of the catalytic domain have been identified in tumor cells, however, their biological significance has not yet been determined.[166] LDHC has been shown to promote tumor growth and metastasis of lung adenocarcinomas via activation of the PI3K/Akt/GSK-3β oncogenic-signaling pathway.[18,167] We previously demonstrated that LDHC is an immunogenic antigen and plays a role in maintaining genomic stability and mitotic fidelity in breast cancer cell lines, safekeeping tumor cellular fitness.[17,168] More specifically, we showed that silencing LDHC results in DNA damage accumulation, microtubule destabilization, mitotic slippage, increased polyploidy and aberrant mitosis, ultimately reducing long-term tumor cell survival. Furthermore, we demonstrated that silencing of LDHC improves sensitivity to treatment with DNA damage response-related drugs.

MAGE-C2/HCA587/CT10

Melanoma-Associated Antigen C2 (MAGE-C2) is often expressed in tumors, including seminomas, melanoma, breast tumors, colorectal carcinomas, prostate cancers, non-small cell lung carcinomas, bladder tumors, hepatocellular carcinomas, head and neck squamous carcinomas, squamous cell carcinomas of the larynx, medulloblastomas, gliomas, multiple myelomas, Hodgkin lymphomas, gastrointestinal stromal tumors, salivary gland carcinomas, esophageal squamous cell carcinomas, in particular in those with features of more advanced disease and poor prognosis. Its expression has been associated with chemoresistance in melanoma, as a likely result of enhanced DNA double-strand break repair following heterochromatin relaxation and increased MAGE-C2 mediated phosphorylation of KAP1-Ser824.[169] Further, MAGE-C2 has been shown to regulate cancer cell proliferation through p53 ubiquitination whereby the E3 ubiquitin ligases TRIM28 and MDM2 compete for binding with MAGE-C2, releasing and activating MDM2 which subsequently ubiquitinates p53.[170,171] MAGE-C2 was also found to directly bind the RING domain protein Rbx1 of the E3 ligase Skp1-Cullin1-F box complex, inhibiting cyclin E ubiquitin-dependent degradation and promoting G1-S cell cycle progression.[172] In multiple myeloma, MAGE-C2 is associated with chemoresistance, promotes double-strand break repair and inhibits p53-dependent apoptosis.[30,173]

OIP5/MIS18B/CT86

Opa interacting protein 5 (OIP5) is a centromere-associated protein that is essential for the recruitment of CENP-A to the site of centromere formation and kinetochore assembly, and as such affects chromosome segregation and maintenance of genomic integrity.[174] It is expressed across a wide range of solid tumors including ovarian, breast, liver, lung and bladder tumors whereby high OIP5 expression correlates with poor overall and disease-specific survival except for glioblastoma for which conflicting findings have been reported. OIP5 copy number has been shown to correlate with immune cell infiltration in clear-cell renal cell carcinoma.[175] Furthermore, gene set enrichment analysis of OIP5 low and high expressing tumors identified differential enrichment of genes involved in base excision repair, homologous recombination, DNA replication, cell cycle progression, and the p53 and mismatch repair pathways. OIP5 expression also positively correlates with tumor mutational burden and microsatellite instability in several cancers.[176] Moreover, OIP5 likely regulates cell cycle progression in a tumor-type specific manner, affecting either G0/G1, G2/M or G1/S transition, leading to apoptosis and senescence.[177–179]

PBK/TOPK/CT84

PDZ-binding kinase (PBK) is a serine threonine kinase capable of phosphorylating and activating several oncogenic signaling pathways.[180] It is expressed in numerous tumor types including hematological malignancies, bladder cancer, breast cancer, glioma, kidney cancer, prostate cancer, lung adenocarcinoma, hepatocellular carcinoma, cervical cancer, esophageal squamous cell carcinoma, osteosarcoma, medulloblastoma, glioblastoma, adrenocortical carcinoma, colon cancer, ovarian cancer, colorectal carcinoma, and gastric cancer. Overall, high PBK expression is associated with poor prognosis, except in patients with colon cancer, cholangiocarcinoma, and oral squamous cell carcinoma. Similarly to OIP5, PBK expression correlates with features of genomic instability such as high tumor mutational burden and microsatellite instability.[181] PBK has been shown to regulate genomic instability through its involvement in multiple cell cycle checkpoints. It is best known as a mitotic kinase that mediates G2/M progression, dissociation of proteins from condensed chromatin, chromosome segregation, spindle formation and cytokinesis.[182,183] In addition, PBK downregulates p53 signaling through either direct interaction, inhibiting transactivation of p53 target genes, or through phosphorylation of histone H3 and subsequent reduction in p53 expression.[182,184] A recent study demonstrated that PBK also plays a role in G1/S transition through phospho-activation of Chk1 and Cdc25C in response to replication stress, and that deletion of PBK renders cancer cells vulnerable to radiation-induced DNA damage.[185,186] Furthermore, inhibition of PBK enhanced treatment response to olaparib and cisplatin in ovarian cancer,[187,188] cisplatin in cervical cancer[189] and paclitaxel in non-small cell lung cancer.[190] Mechanistically, inhibition of PBK was found to induce G1 cell-cycle arrest through activation of the PI3K-Akt-IKK signaling pathway, suppress cell survival signals while promoting pro-apoptosis signaling, and inhibit cancer cell migration and invasion.[191,192]

SSX2/HOM-MEL-40/CT5.2a

Synovial Sarcoma, X-breakpoint 2 (SSX2) belongs to a family of ten homologous members that function as transcription repressors and are widely expressed in tumors. Expression analysis showed aberrant expression of SSX2 in various cancers including melanoma, colon cancer, hepatocellular carcinoma, and breast cancer. SSX2 expression has been associated with advanced disease and worse prognosis and can induce humoral and cellular immune responses in cancer patients. Gene rearrangements can lead to SSX2 fusion genes such as SS18-SSX2, a main oncogenic driver and diagnostic marker in synovial sarcoma, and the more recently identified EWSR1-SSX2 fusion gene in a patient with undifferentiated sarcoma of the bone.[193–195] The tumorigenic role and prognostic value of SSX2 remain elusive. In melanoma, ectopic expression of SSX2 promotes genomic instability through the induction of replication defects and p53-mediated G1 cell-cycle arrest.[31,196] Furthermore, SSX2 reduces the stability of Polycomb-group (PcG) repressive complexes at the chromosome 1q12 pericentromeric heterochromatin structure, a site of frequent genetic aberrations in cancer, promoting the de-repression of 1q12 heterochromatin and resulting in increased genomic instability due to segregation abnormalities and generation of micronuclei.[197] In contrast, in MCF7 breast cancer cells, the cell line's molecular landscape likely supports the maintenance of genomic integrity upon activation of the p53-p21 cell cycle checkpoint and drives SSX2-expressing cancer cells toward senescence through the Mediator complex.[198] These findings highlight the need for further investigation to discern the tissue type-dependent factors that determine the SSX2-associated cell fates in different cancers.

TEX12

Testis-expressed protein 12 (TEX12), like SYCP3, is a component of the synaptonemal complex which localizes to the centrosomes during meiosis and mitosis.[199] TEX12 expression in cancer cells has been associated with centrosome amplification, a process that is closely linked to oncogenesis and poor prognosis.[199] Analysis of a large-scale transcriptomic dataset revealed aberrant expression of TEX12 in breast, ovarian, stomach, liver, glioblastoma and myeloid leukemia cancer cells where it was associated with more aggressive tumors. Furthermore, TEX12 has been identified as one of five DNA damage repair genes that form a prognostic signature that can predict the overall survival and response to immunotherapy of cervical squamous cell carcinoma patients.[200]

CLINICAL TRIALS TARGETING GENOMIC INTEGRITY-REGULATORY CTAS

The discovery of cancer testis antigens as highly tumor-specific antigens with important roles in cancer hallmarks opens up new avenues for cancer cell-specific targeting. Historically, CTAs have been identified through autologous typing of T cell clones and serological analysis of cDNA libraries derived from cancer patients, and since several CTAs have been reported to exhibit immunogenic properties which could be exploited in immune-based interventions.[201,202] Hence, various studies and clinical trials have focused on the potential use of CTA-targeted antibodies, vaccines, and adoptive cell therapy approaches to elicit potent, durable anti-tumor responses. Most notably, NY-ESO-1, MAGE-A3 and PRAME have been extensively studied as prime targets for immunotherapy as reviewed in detail elsewhere.[4,203,204] Likewise, efforts to target genomic integrity-regulatory CTAs have been directed toward the development of immunotherapeutic strategies in addition to small molecule inhibitors (Figure 2).

Immunotherapy

As immunotherapy has emerged at the forefront in cancer treatment, several immunotherapeutic approaches targeting a select few genomic integrity-regulatory CTAs are currently in clinical trial. In particular, Mps1/TTK has been investigated as candidate target using multi-peptide cancer vaccines (NCT00681330, NCT00676949, NCT00674258). Notably, treatment of patients with advanced esophageal cancer with a multi-CTA vaccine against TTK, LY6K and IMP3 (NCT00682227) induced specific T cell immunity and resulted in clinical responses in 50% of patients.[205] Another trial in patients with metastatic esophageal squamous cell carcinoma (NCT00669292) revealed peptide-specific cytotoxic T cell responses and stable disease response rates of 67% after treatment with a TTK and LY6K peptide vaccine in combination with the immunostimulatory TLR9 agonist CpG-7907.[206] Vaccination of patients with advanced or recurrent non-small cell lung cancer with a multi-peptide vaccine targeting TTK, Ly6K, VEGFR1, and VEGFR2 (NCT00633724) induced strong specific T cell responses and stable disease in 47% of patients.[207] To date, one phase I clinical trial has been conducted to study the effect of multi-peptide vaccination (TTK, URLC10, KOC1, VEGFR1, VEGFR2) in combination with chemoradiation therapy in esophageal cancer (NCT00632333), demonstrating peptide-specific cytotoxic T cell immune responses and durable complete responses in 54.5% of patients.[208] In analogy with TTK, the cancer testis antigen MPHOSPH1 is being targeted as part of multi-peptide vaccinations to treat metastatic breast cancer (NCT01259505), bladder cancer (NCT00633204, NCT00635336), unresectable recurrent and/or metastatic solid tumors (NCT04316689), and cervical, gastro-intestinal and lung tumors (NCT00676949); however, no data has been published as yet. In turn, targeting of SSX2 is being explored using multi tumor-associated antigen (TAA)-specific cytotoxic T lymphocytes in patients with breast cancer (TACTIC study, NCT03093350), pancreatic cancer (TACTOPS study, NCT03192462) and lymphoma (NCT01333046).[209] Likewise, various immune-based strategies are currently explored to target MAGE-C2 in solid cancer, including autologous MAGE-C2 engineered T cells (NCT04729543) and multi-peptide vaccines in combination with PD-L1 immune checkpoint inhibition (NCT03164772). Results from a phase 2 study in advanced melanoma demonstrated that dendritic cell-based mRNA and tumor antigen vaccination combined with CTLA-4 blockade (NCT01302496) induced multi-antigen, polyfunctional CD8+ T cell responses in 80% of patients.[210] Another MAGE family member, MAGE-A4, is extensively evaluated as a novel immunotherapy approach to treat patients with a variety of cancers with TAA-specific cytotoxic T lymphocytes (NCT01333046)[209] or genetically modified cytotoxic T cells (NCT01694472, NCT03132922, NCT04044859, NCT05601752, NCT03973333, NCT02096614, NCT04752358,

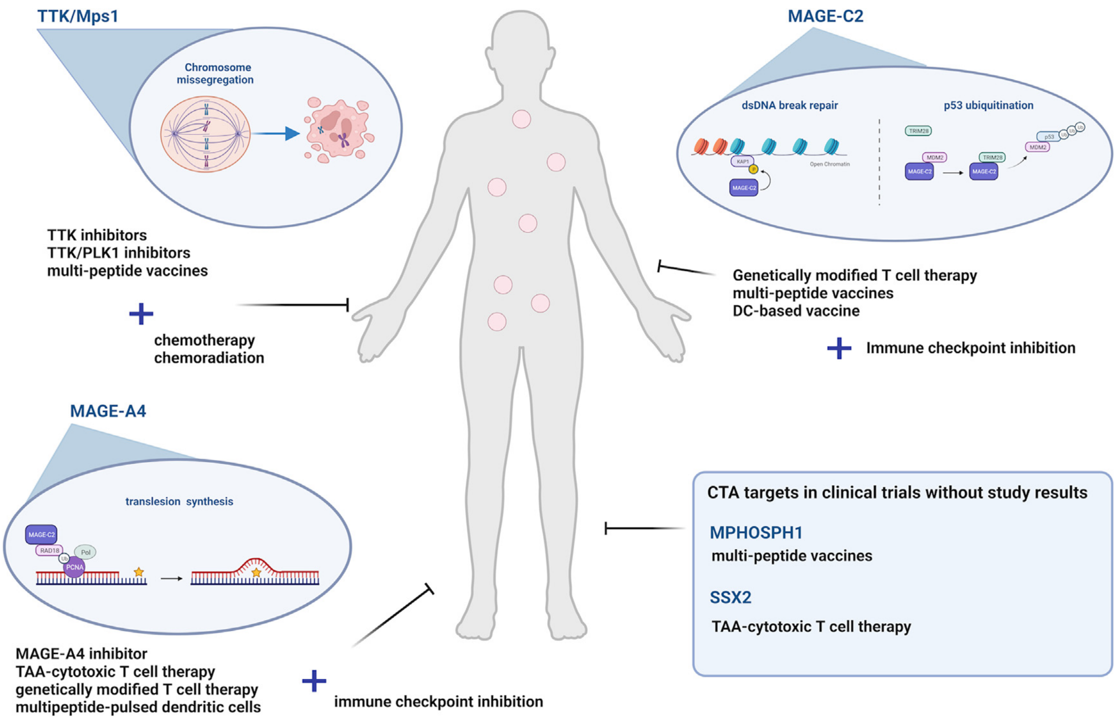

Figure 2. Therapeutic potential of targeting CTAs involved in regulating genomic integrity in cancer
The clinical value of targeting specific CTAs that play key roles in regulating tumor genomic integrity is currently studied in phase I and phase II clinical trials. To date, the majority of clinical studies focus on targeting TTK/Mps1, MAGE-A4 and MAGE-C2 with few trials investigating the safety and anti-tumor activity of targeting MPHOSPH1 and SSX2. DC, dendritic cell; TAA, tumor associated antigen.

NCT04044768, NCT04408898, NCT03247309, NCT03356808, NCT03132922 and NCT04044768).[211–213] Specifically, treatment with autologous T cells with an affinity-optimized T cell receptor against MAGE-A4 (afamitresgene autoleucel or afami-cel) resulted in an overall response rate of 24% in patients with 9 different relapsed/refractory metastatic solid tumor types, with the highest clinical activity in synovial sarcoma (overall response rate of 44%).[212,214] Preliminary peripheral and tumor analyses revealed the presence of MAGE-A4 engineered T cell immune cells in the circulation up to 18 months after treatment, elevated levels of IFN-γ up to 12 days after T cell infusion, and intra-tumoral T cell infiltration. In addition, the combination of autologous dendritic cells pulsed with multi-TAA peptides, including MAGE-A4, and the PD-1 immune checkpoint inhibitor nivolumab is in phase II trial in advanced non-small cell lung cancer (NCT04199559). Preclinical research further suggests that the addition of the CD8α receptor to an affinity-enhanced HLA class I-restricted TCR against MAGE-A4 can enhance CD4+ T helper and effector functions which could improve the depth and durability of anti-tumor immune responses.[215]

Small molecule inhibitors

In contrast to the number of available immunotherapy clinical trials, sparse information is available on clinical trials of targeted therapy against the twenty-eight aforementioned CTAs. More specifically, a few studies have investigated the safety and efficacy of small molecule inhibitors against MAGE-A4 and Mps1/TTK. The MAGE-A4 inhibitor RO7444973 (NCT05129280) is currently under safety, pharmacokinetics, pharmacodynamics, and preliminary efficacy assessment in patients with unresectable and/or metastatic solid tumors.[216] To date, four small molecule inhibitors targeting Mps1/TTK (BAY-1217389, CFI-402257, BOS-172722, and S-81694) are being investigated as single agents or in combination with chemotherapeutic drugs. Treatment with BAY-1217389 in combination with paclitaxel (NCT02366949) demonstrated partial responses in 31.6% of the patients; however, these were associated with considerable toxicity, limiting the therapeutic window.[90] The safety profile and pharmacokinetics of CFI-402257 are under evaluation in solid tumors as either monotherapy, or in combination with hormonal therapy or paclitaxel (NCT02792465, NCT05251714, NCT03568422). The safety, maximum tolerated dose, and anti-tumor activity of BOS-172722 (NCT03328494) and S-81694 (NCT03411161) alone or in combination with paclitaxel are under study in patients with advanced non-hematologic malignancies and metastatic breast cancer. Furthermore, a phase I clinical trial is currently recruiting patients with advanced solid tumors to assess the safety and tolerability of dual inhibition of TTK and PLK1 by BAL0891 with or without carboplatin or paclitaxel (NCT05768932). In addition, preclinical studies have reported that TTK inhibition in combination with radiotherapy enhances mitotic catastrophe and impaired DNA damage repair,

suggesting that the combination treatment may result in synergistic effects.[217]

CHALLENGES OF CTA-TARGETED THERAPY

Traditionally, cancer care comprises of surgery and systemic treatment with chemotherapy and/or radiotherapy. A deeper understanding of molecular differences between breast tumors in the last decades has led to the development of targeted approaches, tailored to interact with specific molecules expressed by the cancer cells. More recently, there has been a surge in immunotherapy clinical trials, demonstrating promising anti-tumor activities in a range of cancers. As such, the development of CTA-based treatment has been focused on cancer vaccines and adoptive cell therapy. However, the benefits in solid cancers have been limited so far, which may be attributed to tumor-intrinsic and extrinsic factors that also play a role in the efficacy of other treatment modalities. Firstly, tumor heterogeneity greatly impacts cancer treatment outcomes. Tumor cells can dysregulate the expression and function of the antigen processing and presentation machinery, thereby impairing the cell surface expression of intracellular CTAs and resulting in tumor cell subpopulations with reduced target expression within a single tumor. In addition, patients may develop acquired resistance through antigen escape whereby CTA-based treatment result in a positive selective pressure toward CTA-negative tumor cell subclones. Aberrant expression of genomic integrity-regulatory CTAs could also directly promote tumor heterogeneity as increased genomic instability enables the acquisition of other cancer hallmarks, giving rise to genetically distinct subpopulations which may be more resistant to combination therapy. Secondly, the tumor microenvironment has a profound effect on the efficacy of cancer treatment. Tumor-derived and microenvironmental cues together shape the tumor immune microenvironment as either immune favorable or unfavorable. Increased genomic instability, conferred by the aberrant tumor expression of genomic integrity-regulatory CTAs, can impede anti-tumor immune responses as the higher tumor cell diversity may facilitate the adoption of immune escape mechanisms. In addition, tumor cells exhibit high rates of aerobic glycolysis and extracellular lactic acidosis, dampening the anti-tumor activity of cytotoxic T cells. Furthermore, we demonstrated that expression of PRAME is associated with increased immune checkpoint expression and reduced T cell functionality.[218] Likewise, expression of CEP55 has been positively correlated with the expression of more than 30 immune checkpoint genes in a wide range of solid tumors, as well as enhanced infiltration of immunosuppressive myeloid-derived suppressor cells and Th2 cells, expression of immunomodulators and response to immune checkpoint blockade.[219–221] Furthermore, high expression of PBK has been associated with immune escape due to upregulation of PD-L1 expression, dysregulation of antigen presentation and immune cell infiltration.[181,222–226] Of note, a subcluster of MAGE-A antigens has been reported to predict treatment response of melanoma tumors to immune checkpoint blockade.[227,228]

We would like to highlight some precautions that should be considered when developing novel CTA-based treatments. We believe that future development of CTA-based cancer care should include a careful comprehensive mapping of CTA expression in normal and tumor tissues along with an accurate annotation of CTAs as testis-restricted or testis-selective antigens in order to advance personalized cancer treatment with favorable safety profiles. The significance of such efforts becomes apparent when considering PBK inhibition, which exhibits remarkable anti-tumor activity in pre-clinical models, but at the same time targets a protein that is crucial for neuronal self-renewal and protection against ischemic postconditioning in the heart and brain.[182] Lastly, it is important to consider the potential adverse effects of CTA-based therapy on male fertility due to the diverse roles of CTAs in spermatogenesis which may be compromised by the uptake of CTA-targeting drugs across the blood-testis-barrier by drug transporters in Sertoli cells of the testis.

FUTURE THERAPEUTIC PERSPECTIVES FOR GENOMIC INTEGRITY-REGULATORY CTAS

With only a handful of genomic integrity-regulatory CTAs currently being evaluated in clinical trials, there is ample room for the development of novel therapeutic opportunities targeting other members of this subset of CTAs. Based on the growing evidence from pre-clinical reports, there are additional less-studied CTAs that could be targeted to leverage genomic instability and improve treatment response to existing treatment regimens including chemotherapy and radiotherapy. Several naturally occurring compounds have been identified as inhibitors of CEP55, MPHOSPH1, and PRDM9 and are undergoing pre-clinical testing,[229–231] Inhibitors targeting PBK/TOPK have been shown to suppress tumor growth and metastasis in cancer xenograft models,[192,232,233] and to sensitize tumor cells to anti-cancer drugs such as olaparib[187] and lenalidomide.[234,235] Further, depletion of BORIS,[236,237] HORMAD1,[54] OIP5[238,239] and CT45[240] results in increased sensitivity of tumor cells to cisplatin and docetaxel treatment in different cancer cell line models. In contrast, the presence of MEIOB was shown to sensitize triple negative breast cancer cells to PARP inhibitors *in vitro* and in patient-derived xenograft models, suggesting that MEIOB expression could be used as a predictive biomarker for treatment response to PARP inhibition.[58] Furthermore, targeting genomic integrity-regulatory CTAs could also be used to promote radiosensitivity. For instance, PBK inhibition by OTS964 has been shown to sensitize ovarian cancer cells to radiotherapy due to increased fork stalling and collapse in response to radiation-induced replication stress and DNA damage, highlighting the potential of combination treatment.[185] In line with this finding, upregulation of miR-372 enhanced the radiosensitivity of nasopharyngeal carcinoma cells by downregulating PKB and subsequently activating the p53 signaling pathway, leading to increased cell death and cell-cycle arrest in response to radiation-induced DNA damage.[241]

Furthermore, preclinical studies are exploring alternative therapeutic approaches including the use of miRNA-based therapy,[241] CTA-specific antibodies[155] and CRISPR/Cas9 gene editing,[240] which will require further validation in clinical studies. Finally, the aforementioned challenges that impact the therapeutic potential of targeting CTAs could be exploited to improve treatment responses. For

instance, CTAs could be targeted in combination with metabolic therapy or drugs to upregulate the antigen presentation machinery.

CONCLUSIONS

In normal physiological conditions, CTAs play critical roles during meiosis by regulating DNA damage repair and maintaining genomic integrity, processes which are also intricately linked to tumor cellular fitness. The highly tumor specific expression of CTAs in conjunction with these pro-tumorigenic functions make them lucrative anti-cancer targets to directly impair tumor cell survival or to enhance treatment responses to drugs that negatively impact genomic stability. As such, CTAs are increasingly being studied in phase I and II clinical trials using specific inhibitors or CTA-specific immunotherapy approaches, either as monotherapy or combination therapy. Initial study results reveal promising anti-tumor activities with durable cellular immune responses in multiple solid tumors, underscoring the need for systematic analysis of CTA expression and functionality in cancer.

SUPPLEMENTAL INFORMATION

Supplemental information can be found online at https://doi.org/10.1016/j.omton.2024.200768.

ACKNOWLEDGMENTS

This work was supported by a grant from the Qatar Biomedical Research Institute (VR94-IGP3-2020), Hamad Bin Khalifa University, Qatar awarded to JD. HQ is supported by a scholarship from the College of Health and Life Sciences, Hamad Bin Khalifa University.

AUTHOR CONTRIBUTIONS

AN: Conceptualization, Writing - Original Draft, Visualization; BL: Writing - Original Draft; HQ: Writing - Original Draft, Visualization; JD: Conceptualization, Visualization, Writing - Review & Editing, Supervision, Project administration, Funding acquisition. All authors reviewed the manuscript.

DECLARATION OF INTERESTS

The authors declare that they have no competing interests.

REFERENCES

1. Chen, Y.T., Ross, D.S., Chiu, R., Zhou, X.K., Chen, Y.Y., Lee, P., Hoda, S.A., Simpson, A.J., Old, L.J., Caballero, O., and Neville, A.M. (2011). Multiple cancer/testis antigens are preferentially expressed in hormone-receptor negative and high-grade breast cancers. PLoS One *6*, e17876. https://doi.org/10.1371/journal.pone.0017876.
2. Caballero, O.L., and Chen, Y.-T. (2009). Cancer/testis (CT) antigens: potential targets for immunotherapy. Cancer Sci. *100*, 2014–2021. https://doi.org/10.1111/j.1349-7006.2009.01303.x.
3. Fratta, E., Coral, S., Covre, A., Parisi, G., Colizzi, F., Danielli, R., Nicolay, H.J.M., Sigalotti, L., and Maio, M. (2011). The biology of cancer testis antigens: putative function, regulation and therapeutic potential. Mol. Oncol. *5*, 164–182. https://doi.org/10.1016/j.molonc.2011.02.001.
4. Li, X.F., Ren, P., Shen, W.Z., Jin, X., and Zhang, J. (2020). The expression, modulation and use of cancer-testis antigens as potential biomarkers for cancer immunotherapy. Am. J. Transl. Res. *12*, 7002–7019.
5. Yang, P., Meng, M., and Zhou, Q. (2021). Oncogenic cancer/testis antigens are a hallmarker of cancer and a sensible target for cancer immunotherapy. Biochim. Biophys. Acta Rev. Canc *1876*, 188558. https://doi.org/10.1016/j.bbcan.2021.188558.
6. Shim, K., Jo, H., and Jeoung, D. (2023). Cancer/Testis Antigens as Targets for RNA-Based Anticancer Therapy. Int. J. Mol. Sci. *24*, 14679. https://doi.org/10.3390/ijms241914679.
7. Ren, S., Zhang, Z., Li, M., Wang, D., Guo, R., Fang, X., and Chen, F. (2023). Cancer testis antigen subfamilies: Attractive targets for therapeutic vaccine (Review). Int. J. Oncol. *62*, 71. https://doi.org/10.3892/ijo.2023.5519.
8. Gibbs, Z.A., and Whitehurst, A.W. (2018). Emerging Contributions of Cancer/Testis Antigens to Neoplastic Behaviors. Trends Cancer *4*, 701–712. https://doi.org/10.1016/j.trecan.2018.08.005.
9. Kim, R., Kulkarni, P., and Hannenhalli, S. (2013). Derepression of Cancer/testis antigens in cancer is associated with distinct patterns of DNA hypomethylation. BMC Cancer *13*, 144. https://doi.org/10.1186/1471-2407-13-144.
10. Van Tongelen, A., Loriot, A., and De Smet, C. (2017). Oncogenic roles of DNA hypomethylation through the activation of cancer-germline genes. Cancer Lett. *396*, 130–137. https://doi.org/10.1016/j.canlet.2017.03.029.
11. Karpf, A.R. (2006). A potential role for epigenetic modulatory drugs in the enhancement of cancer/germ-line antigen vaccine efficacy. Epigenetics *1*, 116–120. https://doi.org/10.4161/epi.1.3.2988.
12. Gjerstorff, M.F., Andersen, M.H., and Ditzel, H.J. (2015). Oncogenic cancer/testis antigens: prime candidates for immunotherapy. Oncotarget *6*, 15772–15787. https://doi.org/10.18632/oncotarget.4694.
13. Wang, C., Gu, Y., Zhang, K., Xie, K., Zhu, M., Dai, N., Jiang, Y., Guo, X., Liu, M., Dai, J., et al. (2016). Systematic identification of genes with a cancer-testis expression pattern in 19 cancer types. Nat. Commun. *7*, 10499. https://doi.org/10.1038/ncomms10499.
14. da Silva, V.L., Fonseca, A.F., Fonseca, M., da Silva, T.E., Coelho, A.C., Kroll, J.E., de Souza, J.E.S., Stransky, B., de Souza, G.A., and de Souza, S.J. (2017). Genome-wide identification of cancer/testis genes and their association with prognosis in a pan-cancer analysis. Oncotarget *8*, 92966–92977. https://doi.org/10.18632/oncotarget.21715.
15. Yao, J., Caballero, O.L., Yung, W.K.A., Weinstein, J.N., Riggins, G.J., Strausberg, R.L., and Zhao, Q. (2014). Tumor subtype-specific cancer-testis antigens as potential biomarkers and immunotherapeutic targets for cancers. Cancer Immunol. Res. *2*, 371–379. https://doi.org/10.1158/2326-6066.CIR-13-0088.
16. Hofmann, O., Caballero, O.L., Stevenson, B.J., Chen, Y.T., Cohen, T., Chua, R., Maher, C.A., Panji, S., Schaefer, U., Kruger, A., et al. (2008). Genome-wide analysis of cancer/testis gene expression. Proc. Natl. Acad. Sci. USA *105*, 20422–20427. https://doi.org/10.1073/pnas.0810777105.
17. Naik, A., and Decock, J. (2022). Targeting of lactate dehydrogenase C dysregulates the cell cycle and sensitizes breast cancer cells to DNA damage response targeted therapy. Mol. Oncol. *16*, 885–903. https://doi.org/10.1002/1878-0261.13024.
18. Chen, L., Wu, Q., Xu, X., Yang, C., You, J., Chen, F., and Zeng, Y. (2021). Cancer/testis antigen LDHC promotes proliferation and metastasis by activating the PI3K/Akt/GSK-3beta-signaling pathway and the in lung adenocarcinoma. Exp. Cell Res. *398*, 112414. https://doi.org/10.1016/j.yexcr.2020.112414.
19. Hua, Y., Liang, C., Zhu, J., Miao, C., Yu, Y., Xu, A., Zhang, J., Li, P., Li, S., Bao, M., et al. (2017). Expression of lactate dehydrogenase C correlates with poor prognosis in renal cell carcinoma. Tumour Biol. *39*, 1010428317695968. https://doi.org/10.1177/1010428317695968.
20. Cui, Z., Li, Y., Gao, Y., Kong, L., Lin, Y., and Chen, Y. (2020). Cancer-testis antigen lactate dehydrogenase C4 in hepatocellular carcinoma: a promising biomarker for early diagnosis, efficacy evaluation and prognosis prediction. Aging (Albany NY) *12*, 19455–19467. https://doi.org/10.18632/aging.103879.
21. Freitas, M., Malheiros, S., Stávale, J.N., Biassi, T.P., Zamunér, F.T., de Souza Begnami, M., Soares, F.A., and Vettore, A.L. (2013). Expression of cancer/testis antigens is correlated with improved survival in glioblastoma. Oncotarget *4*, 636–646. https://doi.org/10.18632/oncotarget.950.

22. Yang, P., Qiao, Y., Meng, M., and Zhou, Q. (2022). Cancer/Testis Antigens as Biomarker and Target for the Diagnosis, Prognosis, and Therapy of Lung Cancer. Front. Oncol. *12*, 864159. https://doi.org/10.3389/fonc.2022.864159.

23. Jahani, M., Shahlaei, M., Norooznezhad, F., Miraghaee, S.S., Hosseinzadeh, L., Moasefi, N., Khodarahmi, R., Farokhi, A., Mahnam, A., and Mansouri, K. (2020). TSGA10 Over Expression Decreases Metastasic and Metabolic Activity by Inhibiting HIF-1 in Breast Cancer Cells. Arch. Med. Res. *51*, 41–53. https://doi.org/10.1016/j.arcmed.2019.12.002.

24. Hu, Y., Xing, J., Wang, L., Huang, M., Guo, X., Chen, L., Lin, M., Zhou, Y., Liu, Z., Zhou, Z., and Sha, J. (2011). RGS22, a novel cancer/testis antigen, inhibits epithelial cell invasion and metastasis. Clin. Exp. Metastasis *28*, 541–549. https://doi.org/10.1007/s10585-011-9390-z.

25. Sakurai, T., Itoh, K., Higashitsuji, H., Nagao, T., Nonoguchi, K., Chiba, T., and Fujita, J. (2004). A cleaved form of MAGE-A4 binds to Miz-1 and induces apoptosis in human cells. J. Biol. Chem. *279*, 15505–15514. https://doi.org/10.1074/jbc.M310437200.

26. Hsiao, Y.J., Su, K.Y., Hsu, Y.C., Chang, G.C., Chen, J.S., Chen, H.Y., Hong, Q.S., Hsu, S.C., Kang, P.H., Hsu, C.Y., et al. (2016). SPANXA suppresses EMT by inhibiting c-JUN/SNAI2 signaling in lung adenocarcinoma. Oncotarget *7*, 44417–44429. https://doi.org/10.18632/oncotarget.10088.

27. Maxfield, K.E., Taus, P.J., Corcoran, K., Wooten, J., Macion, J., Zhou, Y., Borromeo, M., Kollipara, R.K., Yan, J., Xie, Y., et al. (2015). Comprehensive functional characterization of cancer-testis antigens defines obligate participation in multiple hallmarks of cancer. Nat. Commun. *6*, 8840. https://doi.org/10.1038/ncomms9840.

28. Mahmoud, A.M. (2018). Cancer testis antigens as immunogenic and oncogenic targets in breast cancer. Immunotherapy *10*, 769–778. https://doi.org/10.2217/imt-2017-0179.

29. Marcar, L., Maclaine, N.J., Hupp, T.R., and Meek, D.W. (2010). Mage-A cancer/testis antigens inhibit p53 function by blocking its interaction with chromatin. Cancer Res. *70*, 10362–10370. https://doi.org/10.1158/0008-5472.CAN-10-1341.

30. Lajmi, N., Luetkens, T., Yousef, S., Templin, J., Cao, Y., Hildebrandt, Y., Bartels, K., Kröger, N., and Atanackovic, D. (2015). Cancer-testis antigen MAGEC2 promotes proliferation and resistance to apoptosis in Multiple Myeloma. Br. J. Haematol. *171*, 752–762. https://doi.org/10.1111/bjh.13762.

31. Greve, K.B.V., Lindgreen, J.N., Terp, M.G., Pedersen, C.B., Schmidt, S., Mollenhauer, J., Kristensen, S.B., Andersen, R.S., Relster, M.M., Ditzel, H.J., and Gjerstorff, M.F. (2015). Ectopic expression of cancer/testis antigen SSX2 induces DNA damage and promotes genomic instability. Mol. Oncol. *9*, 437–449. https://doi.org/10.1016/j.molonc.2014.09.001.

32. Por, E., Byun, H.J., Lee, E.J., Lim, J.H., Jung, S.Y., Park, I., Kim, Y.M., Jeoung, D.I., and Lee, H. (2010). The cancer/testis antigen CAGE with oncogenic potential stimulates cell proliferation by up-regulating cyclins D1 and E in an AP-1- and E2F-dependent manner. J. Biol. Chem. *285*, 14475–14485. https://doi.org/10.1074/jbc.M109.084400.

33. D'Arcy, P., Maruwge, W., Wolahan, B., Ma, L., and Brodin, B. (2014). Oncogenic functions of the cancer-testis antigen SSX on the proliferation, survival, and signaling pathways of cancer cells. PLoS One *9*, e95136. https://doi.org/10.1371/journal.pone.0095136.

34. Peche, L.Y., Scolz, M., Ladelfa, M.F., Monte, M., and Schneider, C. (2012). MageA2 restrains cellular senescence by targeting the function of PMLIV/p53 axis at the PML-NBs. Cell Death Differ. *19*, 926–936. https://doi.org/10.1038/cdd.2011.173.

35. Yang, F., Zhou, X., Miao, X., Zhang, T., Hang, X., Tie, R., Liu, N., Tian, F., Wang, F., and Yuan, J. (2014). MAGEC2, an epithelial-mesenchymal transition inducer, is associated with breast cancer metastasis. Breast Cancer Res. Treat. *145*, 23–32. https://doi.org/10.1007/s10549-014-2915-9.

36. Maine, E.A., Westcott, J.M., Prechtl, A.M., Dang, T.T., Whitehurst, A.W., and Pearson, G.W. (2016). The cancer-testis antigens SPANX-A/C/D and CTAG2 promote breast cancer invasion. Oncotarget *7*, 14708–14726. https://doi.org/10.18632/oncotarget.7408.

37. Jay, A., Reitz, D., Namekawa, S.H., and Heyer, W.D. (2021). Cancer testis antigens and genomic instability: More than immunology. DNA Repair *108*, 103214. https://doi.org/10.1016/j.dnarep.2021.103214.

38. Nielsen, A.Y., and Gjerstorff, M.F. (2016). Ectopic Expression of Testis Germ Cell Proteins in Cancer and Its Potential Role in Genomic Instability. Int. J. Mol. Sci. *17*, 890. https://doi.org/10.3390/ijms17060890.

39. Yousefzadeh, M., Henpita, C., Vyas, R., Soto-Palma, C., Robbins, P., and Niedernhofer, L. (2021). DNA damage-how and why we age? Elife *10*, e62852. https://doi.org/10.7554/eLife.62852.

40. Hakem, R. (2008). DNA-damage repair; the good, the bad, and the ugly. EMBO J. *27*, 589–605. https://doi.org/10.1038/emboj.2008.15.

41. Zhang, Y., Fang, M., Song, Y., Ren, J., Fang, J., and Wang, X. (2017). Brother of Regulator of Imprinted Sites (BORIS) suppresses apoptosis in colorectal cancer. Sci. Rep. *7*, 40786. https://doi.org/10.1038/srep40786.

42. Zuo, B.W., Yao, W.X., Fang, M.D., Ren, J., Tu, L.L., Fan, R.J., and Zhang, Y.M. (2023). Boris knockout eliminates AOM/DSS-induced in situ colorectal cancer by suppressing DNA damage repair and inflammation. Cancer Sci. *114*, 1972–1985. https://doi.org/10.1111/cas.15732.

43. Zhang, Y., Song, Y., Li, C., Ren, J., Fang, M., Fang, J., and Wang, X. (2020). Brother of regulator of imprinted sites inhibits cisplatin-induced DNA damage in non-small cell lung cancer. Oncol. Lett. *20*, 251. https://doi.org/10.3892/ol.2020.12114.

44. Coscia, F., Lengyel, E., Duraiswamy, J., Ashcroft, B., Bassani-Sternberg, M., Wierer, M., Johnson, A., Wroblewski, K., Montag, A., Yamada, S.D., et al. (2018). Multi-level Proteomics Identifies CT45 as a Chemosensitivity Mediator and Immunotherapy Target in Ovarian Cancer. Cell *175*, 159–170.e16. https://doi.org/10.1016/j.cell.2018.08.065.

45. Suzuki, I., Yoshida, S., Tabu, K., Kusunoki, S., Matsumura, Y., Izumi, H., Asanoma, K., Yagi, H., Onoyama, I., Sonoda, K., et al. (2021). YBX2 and cancer testis antigen 45 contribute to stemness, chemoresistance and a high degree of malignancy in human endometrial cancer. Sci. Rep. *11*, 4220. https://doi.org/10.1038/s41598-021-83200-5.

46. Kogo, H., Tsutsumi, M., Ohye, T., Inagaki, H., Abe, T., and Kurahashi, H. (2012). HORMAD1-dependent checkpoint/surveillance mechanism eliminates asynaptic oocytes. Gene Cell. *17*, 439–454. https://doi.org/10.1111/j.1365-2443.2012.01600.x.

47. Nichols, B.A., Oswald, N.W., McMillan, E.A., McGlynn, K., Yan, J., Kim, M.S., Saha, J., Mallipeddi, P.L., LaDuke, S.A., Villalobos, P.A., et al. (2018). HORMAD1 Is a Negative Prognostic Indicator in Lung Adenocarcinoma and Specifies Resistance to Oxidative and Genotoxic Stress. Cancer Res. *78*, 6196–6208. https://doi.org/10.1158/0008-5472.CAN-18-1377.

48. Herrera, L.R., Johnson, R.A., McGlynn, K., Gibbs, Z.A., Davis, A.J., and Whitehurst, A.W. (2023). The cancer testes antigen, HORMAD1, limits genomic instability in cancer cells by protecting stalled replication forks. J. Biol. Chem. *299*, 105348. https://doi.org/10.1016/j.jbc.2023.105348.

49. Watkins, J., Weekes, D., Shah, V., Gazinska, P., Joshi, S., Sidhu, B., Gillett, C., Pinder, S., Vanoli, F., Jasin, M., et al. (2015). Genomic Complexity Profiling Reveals That HORMAD1 Overexpression Contributes to Homologous Recombination Deficiency in Triple-Negative Breast Cancers. Cancer Discov. *5*, 488–505. https://doi.org/10.1158/2159-8290.CD-14-1092.

50. El-Botty, R., Vacher, S., Mainguené, J., Briaux, A., Ibadioune, S., Dahmani, A., Montaudon, E., Nemati, F., Huguet, L., Sourd, L., et al. (2023). HORMAD1 overexpression predicts response to anthracycline-cyclophosphamide and survival in triple-negative breast cancers. Mol. Oncol. *17*, 2017–2028. https://doi.org/10.1002/1878-0261.13412.

51. Liu, K., Wang, Y., Zhu, Q., Li, P., Chen, J., Tang, Z., Shen, Y., Cheng, X., Lu, L.Y., and Liu, Y. (2020). Aberrantly expressed HORMAD1 disrupts nuclear localization of MCM8-MCM9 complex and compromises DNA mismatch repair in cancer cells. Cell Death Dis. *11*, 519. https://doi.org/10.1038/s41419-020-2736-1.

52. Zong, B., Sun, L., Peng, Y., Wang, Y., Yu, Y., Lei, J., Zhang, Y., Guo, S., Li, K., and Liu, S. (2021). HORMAD1 promotes docetaxel resistance in triple negative breast cancer by enhancing DNA damage tolerance Corrigendum in/10.3892/or.2021.8146. Oncol. Rep. *46*, 1–15. https://doi.org/10.3892/or.2021.8089.

53. Tarantino, D., Walker, C., Weekes, D., Pemberton, H., Davidson, K., Torga, G., Frankum, J., Mendes-Pereira, A.M., Prince, C., Ferro, R., et al. (2022). Functional screening reveals HORMAD1-driven gene dependencies associated with translesion synthesis and replication stress tolerance. Oncogene *41*, 3969–3977. https://doi.org/10.1038/s41388-022-02369-9.

54. Shahzad, M.M.K., Shin, Y.H., Matsuo, K., Lu, C., Nishimura, M., Shen, D.Y., Kang, Y., Hu, W., Mora, E.M., Rodriguez-Aguayo, C., et al. (2013). Biological significance of HORMA domain containing protein 1 (HORMAD1) in epithelial ovarian carcinoma. Cancer Lett. *330*, 123–129. https://doi.org/10.1016/j.canlet.2012.07.001.

55. Gao, Y., Mutter-Rottmayer, E., Greenwalt, A.M., Goldfarb, D., Yan, F., Yang, Y., Martinez-Chacin, R.C., Pearce, K.H., Tateishi, S., Major, M.B., and Vaziri, C. (2016). A neomorphic cancer cell-specific role of MAGE-A4 in trans-lesion synthesis. Nat. Commun. *7*, 12105. https://doi.org/10.1038/ncomms12105.

56. Müller-Richter, U.D.A., Dowejko, A., Driemel, O., Reuther, T., Reichert, T.E., and Kübler, A.C. (2010). Impact of MAGE-A antigens on taxane response in oral squamous cell carcinoma. Oncol. Lett. *1*, 181–185. https://doi.org/10.3892/ol_00000033.

57. Luo, M., Yang, F., Leu, N.A., Landaiche, J., Handel, M.A., Benavente, R., La Salle, S., and Wang, P.J. (2013). MEIOB exhibits single-stranded DNA-binding and exonuclease activities and is essential for meiotic recombination. Nat. Commun. *4*, 2788. https://doi.org/10.1038/ncomms3788.

58. Gu, Y., Wang, C., Zhu, R., Yang, J., Yuan, W., Zhu, Y., Zhou, Y., Qin, N., Shen, H., Ma, H., et al. (2021). The cancer-testis gene, MEIOB, sensitizes triple-negative breast cancer to PARP1 inhibitors by inducing homologous recombination deficiency. Cancer Biol. Med. *18*, 74–87. https://doi.org/10.20892/j.issn.2095-3941.2020.0071.

59. Kulkarni, P., Dunker, A.K., Weninger, K., and Orban, J. (2016). Prostate-associated gene 4 (PAGE4), an intrinsically disordered cancer/testis antigen, is a novel therapeutic target for prostate cancer. Asian J. Androl. *18*, 695–703. https://doi.org/10.4103/1008-682X.181818.

60. Sampson, N., Ruiz, C., Zenzmaier, C., Bubendorf, L., and Berger, P. (2012). PAGE4 positivity is associated with attenuated AR signaling and predicts patient survival in hormone-naive prostate cancer. Am. J. Pathol. *181*, 1443–1454. https://doi.org/10.1016/j.ajpath.2012.06.040.

61. Molania, R., Mahjoubi, F., Mirzaei, R., Khatami, S.-R., and Mahjoubi, B. (2014). A Panel of Cancer Testis Antigens and Clinical Risk Factors to Predict Metastasis in Colorectal Cancer. J. Biomark. *2014*, 272683. https://doi.org/10.1155/2014/272683.

62. Lv, C., Fu, S., Dong, Q., Yu, Z., Zhang, G., Kong, C., Fu, C., and Zeng, Y. (2019). PAGE4 promotes prostate cancer cells survive under oxidative stress through modulating MAPK/JNK/ERK pathway. J. Exp. Clin. Cancer Res. *38*, 24. https://doi.org/10.1186/s13046-019-1032-3.

63. Zeng, Y., Gao, D., Kim, J.J., Shiraishi, T., Terada, N., Kakehi, Y., Kong, C., Getzenberg, R.H., and Kulkarni, P. (2013). Prostate-associated gene 4 (PAGE4) protects cells against stress by elevating p21 and suppressing reactive oxygen species production. Am. J. Clin. Exp. Urol. *1*, 39–52.

64. Gantchev, J., Martínez Villarreal, A., Gunn, S., Zetka, M., Ødum, N., and Litvinov, I.V. (2020). The ectopic expression of meiCT genes promotes meiomitosis and may facilitate carcinogenesis. Cell Cycle *19*, 837–854. https://doi.org/10.1080/15384101.2020.1743902.

65. Houle, A.A., Gibling, H., Lamaze, F.C., Edgington, H.A., Soave, D., Fave, M.J., Agbessi, M., Bruat, V., Stein, L.D., and Awadalla, P. (2018). Aberrant PRDM9 expression impacts the pan-cancer genomic landscape. Genome Res. *28*, 1611–1620. https://doi.org/10.1101/gr.231696.117.

66. Kaiser, V.B., and Semple, C.A. (2018). Chromatin loop anchors are associated with genome instability in cancer and recombination hotspots in the germline. Genome Biol. *19*, 101. https://doi.org/10.1186/s13059-018-1483-4.

67. Hussin, J., Sinnett, D., Casals, F., Idaghdour, Y., Bruat, V., Saillour, V., Healy, J., Grenier, J.C., de Malliard, T., Busche, S., et al. (2013). Rare allelic forms of PRDM9 associated with childhood leukemogenesis. Genome Res. *23*, 419–430. https://doi.org/10.1101/gr.144188.112.

68. Keeney, S. (2008). Spo11 and the Formation of DNA Double-Strand Breaks in Meiosis. Genome Dyn. Stab. *2*, 81–123. https://doi.org/10.1007/7050_2007_026.

69. Baudat, F., Manova, K., Yuen, J.P., Jasin, M., and Keeney, S. (2000). Chromosome synapsis defects and sexually dimorphic meiotic progression in mice lacking Spo11. Mol. Cell *6*, 989–998. https://doi.org/10.1016/s1097-2765(00)00098-8.

70. Yuan, L., Liu, J.G., Zhao, J., Brundell, E., Daneholt, B., and Höög, C. (2000). The murine SCP3 gene is required for synaptonemal complex assembly, chromosome synapsis, and male fertility. Mol. Cell *5*, 73–83. https://doi.org/10.1016/s1097-2765(00)80404-9.

71. Oh, S.J., Cho, H., Kim, S., Noh, K.H., Song, K.-H., Lee, H.-J., Woo, S.R., Kim, S., Choi, C.H., Chung, J.-Y., et al. (2018). Targeting Cyclin D-CDK4/6 Sensitizes Immune-Refractory Cancer by Blocking the SCP3–NANOG Axis. Cancer Res. *78*, 2638–2653. https://doi.org/10.1158/0008-5472.CAN-17-2325.

72. Cho, H., Noh, K.H., Chung, J.-Y., Takikita, M., Chung, E.J., Kim, B.W., Hewitt, S.M., Kim, T.W., and Kim, J.-H. (2014). Synaptonemal complex protein 3 is a prognostic marker in cervical cancer. PLoS One *9*, e98712. https://doi.org/10.1371/journal.pone.0098712.

73. Hosoya, N., Okajima, M., Kinomura, A., Fujii, Y., Hiyama, T., Sun, J., Tashiro, S., and Miyagawa, K. (2011). Synaptonemal complex protein SYCP3 impairs mitotic recombination by interfering with BRCA2. EMBO Rep. *13*, 44–51. https://doi.org/10.1038/embor.2011.221.

74. Qi, J., and Ouyang, Z. (2022). Targeting CDK4/6 for Anticancer Therapy. Biomedicines *10*, 685. https://doi.org/10.3390/biomedicines10030685.

75. Qiao, L., Zhang, L., and Wang, H. (2023). SPAG9 Expression Predicts Good Prognosis in Patients with Clear-Cell Renal Cell Carcinoma: A Bioinformatics Analysis with Experimental Validation. Genes *14*, 944. https://doi.org/10.3390/genes14040944.

76. Yan, Q., Yang, C., Fu, Q., Chen, Z., Liu, S., Fu, D., Rahman, R.N., Nakazato, R., Yoshioka, K., Kung, S.K.P., et al. (2017). Scaffold protein JLP mediates TCR-initiated CD4+T cell activation and CD154 expression. Mol. Immunol. *87*, 258–266. https://doi.org/10.1016/j.molimm.2017.05.006.

77. Kanojia, D., Garg, M., Saini, S., Agarwal, S., Parashar, D., Jagadish, N., Seth, A., Bhatnagar, A., Gupta, A., Kumar, R., et al. (2013). Sperm associated antigen 9 plays an important role in bladder transitional cell carcinoma. PLoS One *8*, e81348. https://doi.org/10.1371/journal.pone.0081348.

78. Miao, Z.F., Wang, Z.N., Zhao, T.T., Xu, Y.Y., Wu, J.H., Liu, X.Y., Xu, H., You, Y., and Xu, H.M. (2015). Overexpression of SPAG9 in human gastric cancer is correlated with poor prognosis. Virchows Arch. *467*, 525–533. https://doi.org/10.1007/s00428-015-1826-4.

79. Xie, C., Fu, L., Liu, N., and Li, Q. (2014). Overexpression of SPAG9 correlates with poor prognosis and tumor progression in hepatocellular carcinoma. Tumour Biol. *35*, 7685–7691. https://doi.org/10.1007/s13277-014-2030-x.

80. Li, H., Peng, Y., Niu, H., Wu, B., Zhang, Y., Zhang, Y., Bai, X., and He, P. (2014). SPAG9 is overexpressed in human prostate cancer and promotes cancer cell proliferation. Tumour Biol. *35*, 6949–6954. https://doi.org/10.1007/s13277-014-1947-4.

81. Jagadish, N., Fatima, R., Sharma, A., Devi, S., Suri, V., Kumar, V., and Suri, A. (2018). Sperm associated antigen 9 (SPAG9) a promising therapeutic target of ovarian carcinoma. Tumour Biol. *40*, 1010428318773652. https://doi.org/10.1177/1010428318773652.

82. Luo, S., Ren, B., Zou, G., Liu, J., Chen, W., Huang, Y., Chen, X., and Fu, Y. (2019). SPAG9/MKK3/p38 axis is a novel therapeutic target for liver cancer. Oncol. Rep. *41*, 2329–2336. https://doi.org/10.3892/or.2019.6987.

83. Pan, J., Yu, H., Guo, Z., Liu, Q., Ding, M., Xu, K., and Mao, L. (2018). Emerging role of sperm-associated antigen 9 in tumorigenesis. Biomed. Pharmacother. *103*, 1212–1216. https://doi.org/10.1016/j.biopha.2018.04.168.

84. Ingram, L., Munro, S., Coutts, A.S., and La Thangue, N.B. (2011). E2F-1 regulation by an unusual DNA damage-responsive DP partner subunit. Cell Death Differ. *18*, 122–132. https://doi.org/10.1038/cdd.2010.70.

85. Qiao, H., Di Stefano, L., Tian, C., Li, Y.Y., Yin, Y.H., Qian, X.P., Pang, X.W., Li, Y., McNutt, M.A., Helin, K., et al. (2007). Human TFDP3, a novel DP protein, inhibits DNA binding and transactivation by E2F. J. Biol. Chem. *282*, 454–466. https://doi.org/10.1074/jbc.M606169200.

86. Ma, Y., Xin, Y., Li, R., Wang, Z., Yue, Q., Xiao, F., and Hao, X. (2014). TFDP3 was expressed in coordination with E2F1 to inhibit E2F1-mediated apoptosis in prostate cancer. Gene *537*, 253–259. https://doi.org/10.1016/j.gene.2013.12.051.

87. Tian, C., Lv, D., Qiao, H., Zhang, J., Yin, Y.H., Qian, X.P., Wang, Y.P., Zhang, Y., and Chen, W.F. (2007). TFDP3 inhibits E2F1-induced, p53-mediated apoptosis. Biochem. Biophys. Res. Commun. *361*, 20–25. https://doi.org/10.1016/j.bbrc.2007.06.128.

88. Jiao, Y., Ding, L., Chu, M., Wang, T., Kang, J., Zhao, X., Li, H., Chen, X., Gao, Z., Gao, L., and Wang, Y. (2017). Effects of cancer-testis antigen, TFDP3, on cell cycle

regulation and its mechanism in L-02 and HepG2 cell lines in vitro. PLoS One *12*, e0182781. https://doi.org/10.1371/journal.pone.0182781.

89. Haschka, M., Karbon, G., Fava, L.L., and Villunger, A. (2018). Perturbing mitosis for anti-cancer therapy: is cell death the only answer? EMBO Rep. *19*, e45440. https://doi.org/10.15252/embr.201745440.

90. Atrafi, F., Boix, O., Subbiah, V., Diamond, J.R., Chawla, S.P., Tolcher, A.W., LoRusso, P.M., Eder, J.P., Gutierrez, M., Sankhala, K., et al. (2021). A Phase I Study of an MPS1 Inhibitor (BAY 1217389) in Combination with Paclitaxel Using a Novel Randomized Continual Reassessment Method for Dose Escalation. Clin. Cancer Res. *27*, 6366–6375. https://doi.org/10.1158/1078-0432.CCR-20-4185.

91. Whitehurst, A.W., Xie, Y., Purinton, S.C., Cappell, K.M., Swanik, J.T., Larson, B., Girard, L., Schorge, J.O., and White, M.A. (2010). Tumor antigen acrosin binding protein normalizes mitotic spindle function to promote cancer cell proliferation. Cancer Res. *70*, 7652–7661. https://doi.org/10.1158/0008-5472.CAN-10-0840.

92. Cheeseman, I.M., Hori, T., Fukagawa, T., and Desai, A. (2008). KNL1 and the CENP-H/I/K complex coordinately direct kinetochore assembly in vertebrates. Mol. Biol. Cell *19*, 587–594. https://doi.org/10.1091/mbc.e07-10-1051.

93. Rosenberg, J.S., Cross, F.R., and Funabiki, H. (2011). KNL1/Spc105 recruits PP1 to silence the spindle assembly checkpoint. Curr. Biol. *21*, 942–947. https://doi.org/10.1016/j.cub.2011.04.011.

94. Cui, Y., Zhang, C., Ma, S., Guo, W., Cao, W., and Guan, F. (2020). CASC5 is a potential tumour driving gene in lung adenocarcinoma. Cell Biochem. Funct. *38*, 733–742. https://doi.org/10.1002/cbf.3540.

95. Bogdanov, K.V., Merzlikina, O.V., Mirolyubova, Y.V., Girshova, L.L., Lomaia, E.G., and Zaritskey, A.Y. (2021). CASC5 Gene Expression Changes Correlate with Targeted Mutations in Leukemia. Mol. Biol. *55*, 121–132. https://doi.org/10.1134/S0026893321010027.

96. Park, H.J., Seo, H.J., Kim, H.W., Kim, J.S., Hwang, S.Y., and Seong, Y.S. (2007). The centrosomal localization of KM-HN-1 (MGC33607) depends on the leucine zipper motif and the C-terminal coiled-coil domain. Exp. Mol. Med. *39*, 828–838. https://doi.org/10.1038/emm.2007.90.

97. Lee, S.N., Hong, K.M., Seong, Y.S., and Kwak, S.J. (2020). Ectopic Overexpression of Coiled-Coil Domain Containing 110 Delays G2/M Entry in U2-OS Cells. Dev. Reprod. *24*, 101–111. https://doi.org/10.12717/DR.2020.24.2.101.

98. Cappell, K.M., Sinnott, R., Taus, P., Maxfield, K., Scarbrough, M., and Whitehurst, A.W. (2012). Multiple cancer testis antigens function to support tumor cell mitotic fidelity. Mol. Cell Biol. *32*, 4131–4140. https://doi.org/10.1128/MCB.00686-12.

99. Park, J.H., Song, M.H., Lee, C.H., Lee, M.K., Park, Y.M., Old, L., and Lee, S.Y. (2011). Expression of the human cancer/testis antigen NY-SAR-35 is activated by CpG island hypomethylation. Biotechnol. Lett. *33*, 1085–1091. https://doi.org/10.1007/s10529-011-0559-y.

100. Song, M.H., Kim, Y.R., Bae, J.H., Shin, D.H., and Lee, S.Y. (2017). A cancer/testis antigen, NY-SAR-35, induces EpCAM, CD44, and CD133, and activates ERK in HEK293 cells. Biochem. Biophys. Res. Commun. *484*, 298–303. https://doi.org/10.1016/j.bbrc.2017.01.105.

101. Song, M.H., Kim, Y.R., Lee, J.W., Lee, C.H., and Lee, S.Y. (2016). Cancer/testis antigen NY-SAR-35 enhances cell proliferation, migration, and invasion. Int. J. Oncol. *48*, 569–576. https://doi.org/10.3892/ijo.2015.3264.

102. Bi, S.Q., Peng, Y., Wei, Z.D., Yao, S.Z., Luo, B., Ge, Y.Y., Xie, X.X., Nong, W.X., Liu, C., Xiao, S.W., and Zhang, Q.M. (2022). FMR1NB Involved in Glioma Tumorigenesis Is a Promising Target for Prognosis and Therapy. Curr. Med. Sci. *42*, 803–816. https://doi.org/10.1007/s11596-022-2586-4.

103. Yu, L., Lang, Y., Hsu, C.C., Chen, W.M., Chiang, J.C., Hsieh, J.T., Story, M.D., Shang, Z.F., Chen, B.P.C., and Saha, D. (2022). Mitotic phosphorylation of tumor suppressor DAB2IP maintains spindle assembly checkpoint and chromosomal stability through activating PLK1-Mps1 signal pathway and stabilizing mitotic checkpoint complex. Oncogene *41*, 489–501. https://doi.org/10.1038/s41388-021-02106-8.

104. Abrieu, A., Magnaghi-Jaulin, L., Kahana, J.A., Peter, M., Castro, A., Vigneron, S., Lorca, T., Cleveland, D.W., and Labbé, J.C. (2001). Mps1 is a kinetochore-associated kinase essential for the vertebrate mitotic checkpoint. Cell *106*, 83–93. https://doi.org/10.1016/s0092-8674(01)00410-x.

105. Sarangapani, K.K., Koch, L.B., Nelson, C.R., Asbury, C.L., and Biggins, S. (2021). Kinetochore-bound Mps1 regulates kinetochore-microtubule attachments via Ndc80 phosphorylation. J. Cell Biol. *220*, e202106130. https://doi.org/10.1083/jcb.202106130.

106. Zheng, L., Chen, Z., Kawakami, M., Chen, Y., Roszik, J., Mustachio, L.M., Kurie, J.M., Villalobos, P., Lu, W., Behrens, C., et al. (2019). Tyrosine Threonine Kinase Inhibition Eliminates Lung Cancers by Augmenting Apoptosis and Polyploidy. Mol. Cancer Therapeut. *18*, 1775–1786. https://doi.org/10.1158/1535-7163.MCT-18-0864.

107. Chan, C.Y.K., Chiu, D.K.C., Yuen, V.W.H., Law, C.T., Wong, B.P.Y., Thu, K.L., Cescon, D.W., Soria-Bretones, I., Cheu, J.W.S., Lee, D., et al. (2022). CFI-402257, a TTK inhibitor, effectively suppresses hepatocellular carcinoma. Proc. Natl. Acad. Sci. USA *119*, e2119514119. https://doi.org/10.1073/pnas.2119514119.

108. Colombo, R., Caldarelli, M., Mennecozzi, M., Giorgini, M.L., Sola, F., Cappella, P., Perrera, C., Depaolini, S.R., Rusconi, L., Cucchi, U., et al. (2010). Targeting the mitotic checkpoint for cancer therapy with NMS-P715, an inhibitor of MPS1 kinase. Cancer Res. *70*, 10255–10264. https://doi.org/10.1158/0008-5472.CAN-10-2101.

109. Waenphimai, O., Mahalapbutr, P., Vaeteewoottacharn, K., Wongkham, S., and Sawanyawisuth, K. (2022). Multiple actions of NMS-P715, the monopolar spindle 1 (MPS1) mitotic checkpoint inhibitor in liver fluke-associated cholangiocarcinoma cells. Eur. J. Pharmacol. *922*, 174899. https://doi.org/10.1016/j.ejphar.2022.174899.

110. Pachis, S.T., and Kops, G.J.P.L. (2018). Leader of the SAC: molecular mechanisms of Mps1/TTK regulation in mitosis. Open Biol. *8*, 180109. https://doi.org/10.1098/rsob.180109.

111. Wengner, A.M., Siemeister, G., Koppitz, M., Schulze, V., Kosemund, D., Klar, U., Stoeckigt, D., Neuhaus, R., Lienau, P., Bader, B., et al. (2016). Novel Mps1 Kinase Inhibitors with Potent Antitumor Activity. Mol. Cancer Therapeut. *15*, 583–592. https://doi.org/10.1158/1535-7163.MCT-15-0500.

112. Kagami, Y., Nihira, K., Wada, S., Ono, M., Honda, M., and Yoshida, K. (2014). Mps1 phosphorylation of condensin II controls chromosome condensation at the onset of mitosis. J. Cell Biol. *205*, 781–790. https://doi.org/10.1083/jcb.201308172.

113. Jelluma, N., Brenkman, A.B., van den Broek, N.J.F., Cruijsen, C.W.A., van Osch, M.H.J., Lens, S.M.A., Medema, R.H., and Kops, G.J.P.L. (2008). Mps1 phosphorylates Borealin to control Aurora B activity and chromosome alignment. Cell *132*, 233–246. https://doi.org/10.1016/j.cell.2007.11.046.

114. Stucke, V.M., Silljé, H.H.W., Arnaud, L., and Nigg, E.A. (2002). Human Mps1 kinase is required for the spindle assembly checkpoint but not for centrosome duplication. EMBO J. *21*, 1723–1732. https://doi.org/10.1093/emboj/21.7.1723.

115. Tardif, K.D., Rogers, A., Cassiano, J., Roth, B.L., Cimbora, D.M., McKinnon, R., Peterson, A., Douce, T.B., Robinson, R., Dorweiler, I., et al. (2011). Characterization of the cellular and antitumor effects of MPI-0479605, a small-molecule inhibitor of the mitotic kinase Mps1. Mol. Cancer Therapeut. *10*, 2267–2275. https://doi.org/10.1158/1535-7163.MCT-11-0453.

116. Schöffski, P., Awada, A., de la Bigne, A.-M., Felloussi, Z., Burbridge, M., Cantero, F., Colombo, R., Maruzzelli, S., Ammattatelli, K., de Jonge, M., et al. (2022). First-in-man, first-in-class phase I study with the monopolar spindle 1 kinase inhibitor S81694 administered intravenously in adult patients with advanced, metastatic solid tumours. Eur. J. Cancer *169*, 135–145. https://doi.org/10.1016/j.ejca.2022.04.001.

117. Anderhub, S.J., Mak, G.W.-Y., Gurden, M.D., Faisal, A., Drosopoulos, K., Walsh, K., Woodward, H.L., Innocenti, P., Westwood, I.M., Naud, S., et al. (2019). High Proliferation Rate and a Compromised Spindle Assembly Checkpoint Confers Sensitivity to the MPS1 Inhibitor BOS172722 in Triple-Negative Breast Cancers. Mol. Cancer Therapeut. *18*, 1696–1707. https://doi.org/10.1158/1535-7163.MCT-18-1203.

118. Lu, L., Wang, Y., Chen, J., Li, Y., Liang, Q., Li, F., Zhen, C., and Xie, K. (2021). Targeting Mps1 in combination with paclitaxel inhibits osteosarcoma progression by modulating spindle assembly checkpoint and Akt/mTOR signaling. Oncol. Lett. *22*, 797. https://doi.org/10.3892/ol.2021.13058.

119. Sarwar, S., Morozov, V.M., Purayil, H., Daaka, Y., and Ishov, A.M. (2022). Inhibition of Mps1 kinase enhances taxanes efficacy in castration resistant prostate cancer. Cell Death Dis. *13*, 868. https://doi.org/10.1038/s41419-022-05312-8.

120. Maia, A.R.R., Linder, S., Song, J.-Y., Vaarting, C., Boon, U., Pritchard, C.E.J., Velds, A., Huijbers, I.J., van Tellingen, O., Jonkers, J., and Medema, R.H. (2018). Mps1 inhibitors synergise with low doses of taxanes in promoting tumour cell death by

enhancement of errors in cell division. Br. J. Cancer *118*, 1586–1595. https://doi.org/10.1038/s41416-018-0081-2.

121. Maia, A.R.R., de Man, J., Boon, U., Janssen, A., Song, J.-Y., Omerzu, M., Sterrenburg, J.G., Prinsen, M.B.W., Willemsen-Seegers, N., de Roos, J.a.D.M., et al. (2015). Inhibition of the spindle assembly checkpoint kinase TTK enhances the efficacy of docetaxel in a triple-negative breast cancer model. Ann. Oncol. *26*, 2180–2192. https://doi.org/10.1093/annonc/mdv293.

122. Jemaà, M., Galluzzi, L., Kepp, O., Senovilla, L., Brands, M., Boemer, U., Koppitz, M., Lienau, P., Prechtl, S., Schulze, V., et al. (2013). Characterization of novel MPS1 inhibitors with preclinical anticancer activity. Cell Death Differ. *20*, 1532–1545. https://doi.org/10.1038/cdd.2013.105.

123. Győrffy, B., Bottai, G., Lehmann-Che, J., Kéri, G., Orfi, L., Iwamoto, T., Desmedt, C., Bianchini, G., Turner, N.C., de Thè, H., et al. (2014). TP53 mutation-correlated genes predict the risk of tumor relapse and identify MPS1 as a potential therapeutic kinase in TP53-mutated breast cancers. Mol. Oncol. *8*, 508–519. https://doi.org/10.1016/j.molonc.2013.12.018.

124. Yu, Z.-C., Huang, Y.-F., and Shieh, S.-Y. (2016). Requirement for human Mps1/TTK in oxidative DNA damage repair and cell survival through MDM2 phosphorylation. Nucleic Acids Res. *44*, 1133–1150. https://doi.org/10.1093/nar/gkv1173.

125. Huang, Y.-F., Chang, M.D.-T., and Shieh, S.-Y. (2009). TTK/hMps1 mediates the p53-dependent postmitotic checkpoint by phosphorylating p53 at Thr18. Mol. Cell Biol. *29*, 2935–2944. https://doi.org/10.1128/MCB.01837-08.

126. Maachani, U.B., Kramp, T., Hanson, R., Zhao, S., Celiku, O., Shankavaram, U., Colombo, R., Caplen, N.J., Camphausen, K., and Tandle, A. (2015). Targeting MPS1 Enhances Radiosensitization of Human Glioblastoma by Modulating DNA Repair Proteins. Mol. Cancer Res. *13*, 852–862. https://doi.org/10.1158/1541-7786.MCR-14-0462-T.

127. Pan, J., Eckardt, S., Leu, N.A., Buffone, M.G., Zhou, J., Gerton, G.L., McLaughlin, K.J., and Wang, P.J. (2009). Inactivation of Nxf2 causes defects in male meiosis and age-dependent depletion of spermatogonia. Dev. Biol. *330*, 167–174. https://doi.org/10.1016/j.ydbio.2009.03.022.

128. Yang, F., Eckardt, S., Leu, N.A., McLaughlin, K.J., and Wang, P.J. (2008). Mouse TEX15 is essential for DNA double-strand break repair and chromosomal synapsis during male meiosis. J. Cell Biol. *180*, 673–679. https://doi.org/10.1083/jcb.200709057.

129. Okutman, O., Muller, J., Baert, Y., Serdarogullari, M., Gultomruk, M., Piton, A., Rombaut, C., Benkhalifa, M., Teletin, M., Skory, V., et al. (2015). Exome sequencing reveals a nonsense mutation in TEX15 causing spermatogenic failure in a Turkish family. Hum. Mol. Genet. *24*, 5581–5588. https://doi.org/10.1093/hmg/ddv290.

130. Lin, X., Chen, Z., Gao, P., Gao, Z., Chen, H., Qi, J., Liu, F., Ye, D., Jiang, H., Na, R., et al. (2017). TEX15: A DNA repair gene associated with prostate cancer risk in Han Chinese. Prostate *77*, 1271–1278. https://doi.org/10.1002/pros.23387.

131. Mantere, T., Tervasmäki, A., Nurmi, A., Rapakko, K., Kauppila, S., Tang, J., Schleutker, J., Kallioniemi, A., Hartikainen, J.M., Mannermaa, A., et al. (2017). Case-control analysis of truncating mutations in DNA damage response genes connects TEX15 and FANCD2 with hereditary breast cancer susceptibility. Sci. Rep. *7*, 681. https://doi.org/10.1038/s41598-017-00766-9.

132. Lin, W.F., Lin, X.L., Fu, S.W., Yang, L., Tang, C.T., Gao, Y.J., Chen, H.Y., and Ge, Z.Z. (2018). Pseudopod-associated protein KIF20B promotes Gli1-induced epithelial-mesenchymal transition modulated by pseudopodial actin dynamic in human colorectal cancer. Mol. Carcinog. *57*, 911–925. https://doi.org/10.1002/mc.22812.

133. Li, Z.Y., Wang, Z.X., and Li, C.C. (2019). Kinesin family member 20B regulates tongue cancer progression by promoting cell proliferation. Mol. Med. Rep. *19*, 2202–2210. https://doi.org/10.3892/mmr.2019.9851.

134. Li, G., Xie, Z.K., Zhu, D.S., Guo, T., Cai, Q.L., and Wang, Y. (2019). KIF20B promotes the progression of clear cell renal cell carcinoma by stimulating cell proliferation. J. Cell. Physiol. *234*, 16517–16525. https://doi.org/10.1002/jcp.28322.

135. Chen, J., Zhao, C.C., Chen, F.R., Feng, G.W., Luo, F., and Jiang, T. (2021). KIF20B Promotes Cell Proliferation and May Be a Potential Therapeutic Target in Pancreatic Cancer. JAMA Oncol. *2021*, 5572402. https://doi.org/10.1155/2021/5572402.

136. Kanehira, M., Katagiri, T., Shimo, A., Takata, R., Shuin, T., Miki, T., Fujioka, T., and Nakamura, Y. (2007). Oncogenic role of MPHOSPH1, a cancer-testis antigen specific to human bladder cancer. Cancer Res. *67*, 3276–3285. https://doi.org/10.1158/0008-5472.CAN-06-3748.

137. Liu, X., Zhou, Y., Liu, X., Peng, A., Gong, H., Huang, L., Ji, K., Petersen, R.B., Zheng, L., and Huang, K. (2014). MPHOSPH1: a potential therapeutic target for hepatocellular carcinoma. Cancer Res. *74*, 6623–6634. https://doi.org/10.1158/0008-5472.CAN-14-1279.

138. Liu, X., Li, Y., Zhang, X., Liu, X.Y., Peng, A., Chen, Y., Meng, L., Chen, H., Zhang, Y., Miao, X., et al. (2018). Inhibition of kinesin family member 20B sensitizes hepatocellular carcinoma cell to microtubule-targeting agents by blocking cytokinesis. Cancer Sci. *109*, 3450–3460. https://doi.org/10.1111/cas.13794.

139. Ciró, M., Prosperini, E., Quarto, M., Grazini, U., Walfridsson, J., McBlane, F., Nucifero, P., Pacchiana, G., Capra, M., Christensen, J., and Helin, K. (2009). ATAD2 is a novel cofactor for MYC, overexpressed and amplified in aggressive tumors. Cancer Res. *69*, 8491–8498. https://doi.org/10.1158/0008-5472.CAN-09-2131.

140. Tong, Y., Li, J., Peng, M., Qian, Q., Shi, W., Chen, Z., and Liu, B. (2022). ATAD2 drives colorectal cancer progression by regulating TRIM25 expression via a positive feedback loop with E2F transcriptional factors. Biochem. Biophys. Res. Commun. *594*, 146–152. https://doi.org/10.1016/j.bbrc.2022.01.036.

141. Koo, S.J., Fernández-Montalván, A.E., Badock, V., Ott, C.J., Holton, S.J., von Ahsen, O., Toedling, J., Vittori, S., Bradner, J.E., and Gorjánácz, M. (2016). ATAD2 is an epigenetic reader of newly synthesized histone marks during DNA replication. Oncotarget *7*, 70323–70335. https://doi.org/10.18632/oncotarget.11855.

142. Revenko, A.S., Kalashnikova, E.V., Gemo, A.T., Zou, J.X., and Chen, H.-W. (2010). Chromatin loading of E2F-MLL complex by cancer-associated coregulator ANCCA via reading a specific histone mark. Mol. Cell Biol. *30*, 5260–5272. https://doi.org/10.1128/MCB.00484-10.

143. Duan, Z., Andrews, N.P., Chen, C.Z., Fan, M., Wang, J., Shen, J., Li, J.J., and Chen, H.W. (2020). Targeting bromodomain protein ANCCA/ATAD2 enhances the efficacy of DNA-damaging chemotherapy agents and radiation. Oncol. Rep. *43*, 318–327. https://doi.org/10.3892/or.2019.7418.

144. Dutta, M., Mohapatra, D., Mohapatra, A.P., Senapati, S., and Roychowdhury, A. (2022). ATAD2 suppression enhances the combinatorial effect of gemcitabine and radiation in pancreatic cancer cells. Biochem. Biophys. Res. Commun. *635*, 179–186. https://doi.org/10.1016/j.bbrc.2022.10.021.

145. Wang, B., Pelletier, J., Massaad, M.J., Herscovics, A., and Shore, G.C. (2004). The yeast split-ubiquitin membrane protein two-hybrid screen identifies BAP31 as a regulator of the turnover of endoplasmic reticulum-associated protein tyrosine phosphatase-like B. Mol. Cell Biol. *24*, 2767–2778. https://doi.org/10.1128/MCB.24.7.2767-2778.2004.

146. Chandra, D., Choy, G., Deng, X., Bhatia, B., Daniel, P., and Tang, D.G. (2004). Association of active caspase 8 with the mitochondrial membrane during apoptosis: potential roles in cleaving BAP31 and caspase 3 and mediating mitochondrion-endoplasmic reticulum cross talk in etoposide-induced cell death. Mol. Cell Biol. *24*, 6592–6607. https://doi.org/10.1128/MCB.24.15.6592-6607.2004.

147. Grimm, S. (2012). The ER-mitochondria interface: the social network of cell death. Biochim. Biophys. Acta *1823*, 327–334. https://doi.org/10.1016/j.bbamcr.2011.11.018.

148. Jiang, X., Li, G., Zhu, B., Zang, J., Lan, T., Jiang, R., and Wang, B. (2023). p20BAP31 induces cell apoptosis via both AIF caspase-independent and the ROS/JNK mitochondrial pathway in colorectal cancer. Cell. Mol. Biol. Lett. *28*, 25. https://doi.org/10.1186/s11658-023-00434-z.

149. Chen, J., Guo, H., Jiang, H., Namusamba, M., Wang, C., Lan, T., Wang, T., and Wang, B. (2019). A BAP31 intrabody induces gastric cancer cell death by inhibiting p27(kip1) proteasome degradation. Int. J. Cancer *144*, 2051–2062. https://doi.org/10.1002/ijc.31930.

150. Wang, A., Zhang, Y., and Cao, P. (2019). Inhibition of BAP31 expression inhibits cervical cancer progression by suppressing metastasis and inducing intrinsic and extrinsic apoptosis. Biochem. Biophys. Res. Commun. *508*, 499–506. https://doi.org/10.1016/j.bbrc.2018.11.017.

151. Liang, H., Dong, J., Cheng, Z., Li, Q., Feng, D., and Ling, B. (2021). B-cell receptor-associated protein 31 promotes migration and invasion in ovarian cancer cells. Exp. Ther. Med. *22*, 858. https://doi.org/10.3892/etm.2021.10290.

152. Liu, T., Yu, J., Ge, C., Zhao, F., Miao, C., Jin, W., Su, Y., Geng, Q., Chen, T., Xie, H., et al. (2021). B-Cell Receptor-Associated Protein 31 Promotes Metastasis via AKT/beta-Catenin/Snail Pathway in Hepatocellular Carcinoma. Front. Mol. Biosci. *8*, 656151. https://doi.org/10.3389/fmolb.2021.656151.

153. Li, T., Hao, Z., Tang, Z., Li, C., Cheng, L., Wang, T., Zhu, X., He, Y., Huang, Y., and Wang, B. (2022). BAP31 Regulates Wnt Signaling to Modulate Cell Migration in Lung Cancer. Front. Oncol. *12*, 859195. https://doi.org/10.3389/fonc.2022.859195.

154. Sun, M., Liu, X., Wei, W., Ge, N., Luo, S., Shen, S., and Ge, R. (2022). BAP31 Promotes Proliferation, Invasion, and Metastasis of Liver Cancer Cells via Activating PI3K/AKT Pathway. J. Healthc. Eng. *2022*, 7686728. https://doi.org/10.1155/2022/7686728.

155. Zhang, X., Jiang, D., Yang, S., Sun, Y., Liu, Y., Shi, J., Hu, C., Pan, J., Liu, T., Jin, B., and Yang, K. (2020). BAP31 Promotes Tumor Cell Proliferation by Stabilizing SERPINE2 in Hepatocellular Carcinoma. Front. Cell Dev. Biol. *8*, 607906. https://doi.org/10.3389/fcell.2020.607906.

156. Yang, S., Sun, Y., Jiang, D., Wang, J., Dang, E., Li, Z., Zhou, J., Lu, Y., Shi, J., Tao, L., et al. (2021). MiR-362 suppresses cervical cancer progression via directly targeting BAP31 and activating TGFbeta/Smad pathway. Cancer Med. *10*, 305–316. https://doi.org/10.1002/cam4.3601.

157. van der Horst, A., Simmons, J., and Khanna, K.K. (2009). Cep55 stabilization is required for normal execution of cytokinesis. Cell Cycle *8*, 3742–3749. https://doi.org/10.4161/cc.8.22.10047.

158. Sinha, D., Nag, P., Nanayakkara, D., Duijf, P.H.G., Burgess, A., Raninga, P., Smits, V.A.J., Bain, A.L., Subramanian, G., Wall, M., et al. (2020). Cep55 overexpression promotes genomic instability and tumorigenesis in mice. Commun. Biol. *3*, 593. https://doi.org/10.1038/s42003-020-01304-6.

159. Jeffery, J., Sinha, D., Srihari, S., Kalimutho, M., and Khanna, K.K. (2016). Beyond cytokinesis: the emerging roles of CEP55 in tumorigenesis. Oncogene *35*, 683–690. https://doi.org/10.1038/onc.2015.128.

160. Kalimutho, M., Sinha, D., Jeffery, J., Nones, K., Srihari, S., Fernando, W.C., Duijf, P.H., Vennin, C., Raninga, P., Nanayakkara, D., et al. (2018). CEP55 is a determinant of cell fate during perturbed mitosis in breast cancer. EMBO Mol. Med. *10*, e8566. https://doi.org/10.15252/emmm.201708566.

161. Zhang, X., Xu, Q., Li, E., Shi, T., and Chen, H. (2023). CEP55 predicts the poor prognosis and promotes tumorigenesis in endometrial cancer by regulating the Foxo1 signaling. Mol. Cell. Biochem. *478*, 1561–1571. https://doi.org/10.1007/s11010-022-04607-w.

162. Wu, J., Chen, Y., Lin, Y., Lan, F., and Cui, Z. (2022). Cancer-testis antigen lactate dehydrogenase C4 as a novel biomarker of male infertility and cancer. Front. Oncol. *12*, 936767. https://doi.org/10.3389/fonc.2022.936767.

163. Odet, F., Gabel, S., London, R.E., Goldberg, E., and Eddy, E.M. (2013). Glycolysis and mitochondrial respiration in mouse LDHC-null sperm. Biol. Reprod. *88*, 95. https://doi.org/10.1095/biolreprod.113.108530.

164. Odet, F., Gabel, S.A., Williams, J., London, R.E., Goldberg, E., and Eddy, E.M. (2011). Lactate dehydrogenase C and energy metabolism in mouse sperm. Biol. Reprod. *85*, 556–564. https://doi.org/10.1095/biolreprod.111.091546.

165. Naik, A., and Decock, J. (2023). Commentary: Cancer-testis antigen lactate dehydrogenase C4 as a novel biomarker of male infertility and cancer. Front. Oncol. *13*, 1115620. https://doi.org/10.3389/fonc.2023.1115620.

166. Koslowski, M., Türeci, O., Bell, C., Krause, P., Lehr, H.A., Brunner, J., Seitz, G., Nestle, F.O., Huber, C., and Sahin, U. (2002). Multiple splice variants of lactate dehydrogenase C selectively expressed in human cancer. Cancer Res. *62*, 6750–6755.

167. Tan, H., Wang, H., Ma, J., Deng, H., He, Q., Chen, Q., and Zhang, Q. (2022). Identification of human LDHC4 as a potential target for anticancer drug discovery. Acta Pharm. Sin. B *12*, 2348–2357. https://doi.org/10.1016/j.apsb.2021.12.002.

168. Thomas, R., Shaath, H., Naik, A., Toor, S.M., Elkord, E., and Decock, J. (2020). Identification of two HLA-A*0201 immunogenic epitopes of lactate dehydrogenase C (LDHC): potential novel targets for cancer immunotherapy. Cancer Immunol. Immunother. *69*, 449–463. https://doi.org/10.1007/s00262-020-02480-4.

169. Bhatia, N., Xiao, T.Z., Rosenthal, K.A., Siddiqui, I.A., Thiyagarajan, S., Smart, B., Meng, Q., Zuleger, C.L., Mukhtar, H., Kenney, S.C., et al. (2013). MAGE-C2 promotes growth and tumorigenicity of melanoma cells, phosphorylation of KAP1, and DNA damage repair. J. Invest. Dermatol. *133*, 759–767. https://doi.org/10.1038/jid.2012.355.

170. Liu, Y., Cao, B., Hu, L., Ye, J., Tian, W., and He, X. (2022). The Dual Roles of MAGE-C2 in p53 Ubiquitination and Cell Proliferation Through E3 Ligases MDM2 and TRIM28. Front. Cell Dev. Biol. *10*, 922675. https://doi.org/10.3389/fcell.2022.922675.

171. Espantman, K.C., and O'Shea, C.C. (2010). aMAGEing new players enter the RING to promote ubiquitylation. Mol. Cell *39*, 835–837. https://doi.org/10.1016/j.molcel.2010.09.006.

172. Hao, J., Song, X., Wang, J., Guo, C., Li, Y., Li, B., Zhang, Y., and Yin, Y. (2015). Cancer-testis antigen MAGE-C2 binds Rbx1 and inhibits ubiquitin ligase-mediated turnover of cyclin E. Oncotarget *6*, 42028–42039. https://doi.org/10.18632/oncotarget.5973.

173. von Boehmer, L., Keller, L., Mortezavi, A., Provenzano, M., Sais, G., Hermanns, T., Sulser, T., Jungbluth, A.A., Old, L.J., Kristiansen, G., et al. (2011). MAGE-C2/CT10 protein expression is an independent predictor of recurrence in prostate cancer. PLoS One *6*, e21366. https://doi.org/10.1371/journal.pone.0021366.

174. Fujita, Y., Hayashi, T., Kiyomitsu, T., Toyoda, Y., Kokubu, A., Obuse, C., and Yanagida, M. (2007). Priming of centromere for CENP-A recruitment by human hMis18alpha, hMis18beta, and M18BP1. Dev. Cell *12*, 17–30. https://doi.org/10.1016/j.devcel.2006.11.002.

175. Gong, M., Li, Y., Song, E., Li, M., Qiu, S., Dong, W., and Yuan, R. (2022). OIP5 Is a Novel Prognostic Biomarker in Clear Cell Renal Cell Cancer Correlating With Immune Infiltrates. Front. Immunol. *13*, 805552. https://doi.org/10.3389/fimmu.2022.805552.

176. Pan, M., Wang, Y., Wang, Z., Duan, H., Shao, C., Ding, P., Lei, J., Zhao, J., Ma, Z., Zhang, F., et al. (2023). The mitosis-related gene OIP5 is a potential biomarker in pan-cancer. Ann. Transl. Med. *11*, 117. https://doi.org/10.21037/atm-22-6640.

177. Zhu, M., Takano, A., Tsevegjav, B., Yoshitake, Y., Shinohara, M., and Daigo, Y. (2022). Characterization of Opa interacting protein 5 as a new biomarker and therapeutic target for oral cancer. Int. J. Oncol. *60*, 27. https://doi.org/10.3892/ijo.2022.5317.

178. Li, Y., Xiao, F., Li, W., Hu, P., Xu, R., Li, J., Li, G., and Zhu, C. (2019). Overexpression of Opa interacting protein 5 increases the progression of liver cancer via BMPR2/JUN/CHEK1/RAC1 dysregulation. Oncol. Rep. *41*, 2075–2088. https://doi.org/10.3892/or.2019.7006.

179. He, X., Hou, J., Ping, J., Wen, D., and He, J. (2017). Opa interacting protein 5 acts as an oncogene in bladder cancer. J. Cancer Res. Clin. Oncol. *143*, 2221–2233. https://doi.org/10.1007/s00432-017-2485-4.

180. Huang, H., Lee, M.H., Liu, K., Dong, Z., Ryoo, Z., and Kim, M.O. (2021). PBK/TOPK: An Effective Drug Target with Diverse Therapeutic Potential. Cancers *13*, 2232. https://doi.org/10.3390/cancers13092232.

181. Wen, H., Chen, Z., Li, M., Huang, Q., Deng, Y., Zheng, J., Xiong, M., Wang, P., and Zhang, W. (2021). An Integrative Pan-Cancer Analysis of PBK in Human Tumors. Front. Mol. Biosci. *8*, 755911. https://doi.org/10.3389/fmolb.2021.755911.

182. Han, Z., Li, L., Huang, Y., Zhao, H., and Luo, Y. (2021). PBK/TOPK: A Therapeutic Target Worthy of Attention. Cells *10*, 371. https://doi.org/10.3390/cells10020371.

183. Liu, Y., Liu, H., Cao, H., Song, B., Zhang, W., and Zhang, W. (2015). PBK/TOPK mediates promyelocyte proliferation via Nrf2-regulated cell cycle progression and apoptosis. Oncol. Rep. *34*, 3288–3296. https://doi.org/10.3892/or.2015.4308.

184. Hu, F., Gartenhaus, R.B., Eichberg, D., Liu, Z., Fang, H.B., and Rapoport, A.P. (2010). PBK/TOPK interacts with the DBD domain of tumor suppressor p53 and modulates expression of transcriptional targets including p21. Oncogene *29*, 5464–5474. https://doi.org/10.1038/onc.2010.275.

185. Herbert, K.J., Puliyadi, R., Prevo, R., Rodriguez-Berriguete, G., Ryan, A., Ramadan, K., and Higgins, G.S. (2021). Targeting TOPK sensitises tumour cells to radiation-induced damage by enhancing replication stress. Cell Death Differ. *28*, 1333–1346. https://doi.org/10.1038/s41418-020-00655-1.

186. Mao, P., Bao, G., Wang, Y.-C., Du, C.-W., Yu, X., Guo, X.-Y., Li, R.-C., and Wang, M.-D. (2020). PDZ-Binding Kinase-Dependent Transcriptional Regulation of CCNB2 Promotes Tumorigenesis and Radio-Resistance in Glioblastoma. Transl. Oncol. *13*, 287–294. https://doi.org/10.1016/j.tranon.2019.09.011.

187. Ma, H., Qi, G., Han, F., Peng, J., Yuan, C., and Kong, B. (2022). PBK drives PARP inhibitor resistance through the TRIM37/NFκB axis in ovarian cancer. Exp. Mol. Med. *54*, 999–1010. https://doi.org/10.1038/s12276-022-00809-w.

188. Ma, H., Li, Y., Wang, X., Wu, H., Qi, G., Li, R., Yang, N., Gao, M., Yan, S., Yuan, C., and Kong, B. (2019). PBK, targeted by EVI1, promotes metastasis and confers cisplatin resistance through inducing autophagy in high-grade serous ovarian carcinoma. Cell Death Dis. *10*, 166. https://doi.org/10.1038/s41419-019-1415-6.

189. Ma, H., Han, F., Yan, X., Qi, G., Li, Y., Li, R., Yan, S., Yuan, C., Song, K., and Kong, B. (2021). PBK promotes aggressive phenotypes of cervical cancer through ERK/c-Myc signaling pathway. J. Cell. Physiol. *236*, 2767–2781. https://doi.org/10.1002/jcp.30134.

190. Park, J.-H., Park, S.-A., Lee, Y.-J., Park, H.-W., and Oh, S.-M. (2020). PBK attenuates paclitaxel-induced autophagic cell death by suppressing p53 in H460 non-small-cell lung cancer cells. FEBS Open Bio *10*, 937–950. https://doi.org/10.1002/2211-5463.12855.

191. Kar, A., Zhang, Y., Yacob, B.W., Saeed, J., Tompkins, K.D., Bagby, S.M., Pitts, T.M., Somerset, H., Leong, S., Wierman, M.E., and Kiseljak-Vassiliades, K. (2019). Targeting PDZ-binding kinase is anti-tumorigenic in novel preclinical models of ACC. Endocr. Relat. Cancer *26*, 765–778. https://doi.org/10.1530/ERC-19-0262.

192. Ishikawa, C., Senba, M., and Mori, N. (2018). Mitotic kinase PBK/TOPK as a therapeutic target for adult T-cell leukemia/lymphoma. Int. J. Oncol. *53*, 801–814. https://doi.org/10.3892/ijo.2018.4427.

193. Eisenhardt, A.E., Brugger, Z., Lausch, U., Kiefer, J., Zeller, J., Runkel, A., Schmid, A., Bronsert, P., Wehrle, J., Leithner, A., et al. (2022). Genotyping of Circulating Free DNA Enables Monitoring of Tumor Dynamics in Synovial Sarcomas. Cancers *14*, 2078. https://doi.org/10.3390/cancers14092078.

194. Cordier, F., Van der Meulen, J., Van Gaever, B., Lapeire, L., Sys, G., Van Dorpe, J., and Creytens, D. (2022). Undifferentiated sarcoma of bone with a round to epithelioid cell phenotype harboring a novel EWSR1-SSX2 fusion identified by RNA-based next-generation sequencing. Genes Chromosomes Cancer *61*, 44–49. https://doi.org/10.1002/gcc.22999.

195. Zaborowski, M., Vargas, A.C., Pulvers, J., Clarkson, A., de Guzman, D., Sioson, L., Maclean, F., Chou, A., and Gill, A.J. (2020). When used together SS18-SSX fusion-specific and SSX C-terminus immunohistochemistry are highly specific and sensitive for the diagnosis of synovial sarcoma and can replace FISH or molecular testing in most cases. Histopathology *77*, 588–600. https://doi.org/10.1111/his.14190.

196. Gjerstorff, M.F., Relster, M.M., Greve, K.B.V., Moeller, J.B., Elias, D., Lindgreen, J.N., Schmidt, S., Mollenhauer, J., Voldborg, B., Pedersen, C.B., et al. (2014). SSX2 is a novel DNA-binding protein that antagonizes polycomb group body formation and gene repression. Nucleic Acids Res. *42*, 11433–11446. https://doi.org/10.1093/nar/gku852.

197. Traynor, S., Møllegaard, N.E., Jørgensen, M.G., Brückmann, N.H., Pedersen, C.B., Terp, M.G., Johansen, S., Dejardin, J., Ditzel, H.J., and Gjerstorff, M.F. (2019). Remodeling and destabilization of chromosome 1 pericentromeric heterochromatin by SSX proteins. Nucleic Acids Res. *47*, 6668–6684. https://doi.org/10.1093/nar/gkz396.

198. Brückmann, N.H., Bennedsen, S.N., Duijf, P.H.G., Terp, M.G., Thomassen, M., Larsen, M., Pedersen, C.B., Kruse, T., Alcaraz, N., Ditzel, H.J., and Gjerstorff, M.F. (2019). A functional genetic screen identifies the Mediator complex as essential for SSX2-induced senescence. Cell Death Dis. *10*, 841. https://doi.org/10.1038/s41419-019-2068-1.

199. Sandhu, S., Sou, I.F., Hunter, J.E., Salmon, L., Wilson, C.L., Perkins, N.D., Hunter, N., Davies, O.R., and McClurg, U.L. (2021). Centrosome dysfunction associated with somatic expression of the synaptonemal complex protein TEX12. Commun. Biol. *4*, 1371. https://doi.org/10.1038/s42003-021-02887-4.

200. Zhou, H., Wu, L., Yu, L., Yang, Y., Kong, L., Liu, S., Chen, W., and Li, R. (2022). Identify a DNA Damage Repair Gene Signature for Predicting Prognosis and Immunotherapy Response in Cervical Squamous Cell Carcinoma. JAMA Oncol. *2022*, 8736575. https://doi.org/10.1155/2022/8736575.

201. van der Bruggen, P., Traversari, C., Chomez, P., Lurquin, C., De Plaen, E., Van den Eynde, B., Knuth, A., and Boon, T. (1991). A gene encoding an antigen recognized by cytolytic T lymphocytes on a human melanoma. Science *254*, 1643–1647. https://doi.org/10.1126/science.1840703.

202. Sahin, U., Türeci, O., Schmitt, H., Cochlovius, B., Johannes, T., Schmits, R., Stenner, F., Luo, G., Schobert, I., and Pfreundschuh, M. (1995). Human neoplasms elicit multiple specific immune responses in the autologous host. Proc. Natl. Acad. Sci. USA *92*, 11810–11813. https://doi.org/10.1073/pnas.92.25.11810.

203. Thomas, R., Al-Khadairi, G., Roelands, J., Hendrickx, W., Dermime, S., Bedognetti, D., and Decock, J. (2018). NY-ESO-1 Based Immunotherapy of Cancer: Current Perspectives. Front. Immunol. *9*, 947. https://doi.org/10.3389/fimmu.2018.00947.

204. Al-Khadairi, G., and Decock, J. (2019). Cancer Testis Antigens and Immunotherapy: Where Do We Stand in the Targeting of PRAME? Cancers *11*, 984. https://doi.org/10.3390/cancers11070984.

205. Kono, K., Mizukami, Y., Daigo, Y., Takano, A., Masuda, K., Yoshida, K., Tsunoda, T., Kawaguchi, Y., Nakamura, Y., and Fujii, H. (2009). Vaccination with multiple peptides derived from novel cancer-testis antigens can induce specific T-cell responses and clinical responses in advanced esophageal cancer. Cancer Sci. *100*, 1502–1509. https://doi.org/10.1111/j.1349-7006.2009.01200.x.

206. Iwahashi, M., Katsuda, M., Nakamori, M., Nakamura, M., Naka, T., Ojima, T., Iida, T., and Yamaue, H. (2010). Vaccination with peptides derived from cancer-testis antigens in combination with CpG-7909 elicits strong specific CD8+ T cell response in patients with metastatic esophageal squamous cell carcinoma. Cancer Sci. *101*, 2510–2517. https://doi.org/10.1111/j.1349-7006.2010.01732.x.

207. Suzuki, H., Fukuhara, M., Yamaura, T., Mutoh, S., Okabe, N., Yaginuma, H., Hasegawa, T., Yonechi, A., Osugi, J., Hoshino, M., et al. (2013). Multiple therapeutic peptide vaccines consisting of combined novel cancer testis antigens and anti-angiogenic peptides for patients with non-small cell lung cancer. J. Transl. Med. *11*, 97. https://doi.org/10.1186/1479-5876-11-97.

208. Iinuma, H., Fukushima, R., Inaba, T., Tamura, J., Inoue, T., Ogawa, E., Horikawa, M., Ikeda, Y., Matsutani, N., Takeda, K., et al. (2014). Phase I clinical study of multiple epitope peptide vaccine combined with chemoradiation therapy in esophageal cancer patients. J. Transl. Med. *12*, 84. https://doi.org/10.1186/1479-5876-12-84.

209. Vasileiou, S., Lulla, P.D., Tzannou, I., Watanabe, A., Kuvalekar, M., Callejas, W.L., Bilgi, M., Wang, T., Wu, M.J., Kamble, R., et al. (2021). T-Cell Therapy for Lymphoma Using Nonengineered Multiantigen-Targeted T Cells Is Safe and Produces Durable Clinical Effects. J. Clin. Oncol. *39*, 1415–1425. https://doi.org/10.1200/JCO.20.02224.

210. De Keersmaecker, B., Claerhout, S., Carrasco, J., Bar, I., Corthals, J., Wilgenhof, S., Neyns, B., and Thielemans, K. (2020). TriMix and tumor antigen mRNA electroporated dendritic cell vaccination plus ipilimumab: link between T-cell activation and clinical responses in advanced melanoma. J. Immunother. Cancer *8*, e000329. https://doi.org/10.1136/jitc-2019-000329.

211. He, K., Hong, D.S., Ke, D., Kebriaei, P., Wang, T., Danesi, H., Bertolet, G., Leuschner, C., Puebla-Osorio, N., Voss, T.A., et al. (2023). Durable control of metastases in an HLA-A2+ patient with refractory melanoma after low-dose radiotherapy in combination with MAGE-A4 T cell therapy: a case report. Melanoma Res. *33*, 332–337. https://doi.org/10.1097/CMR.0000000000000869.

212. Hong, D.S., Van Tine, B.A., Biswas, S., McAlpine, C., Johnson, M.L., Olszanski, A.J., Clarke, J.M., Araujo, D., Blumenschein, G.R., Kebriaei, P., et al. (2023). Autologous T cell therapy for MAGE-A4(+) solid cancers in HLA-A*02(+) patients: a phase 1 trial. Nat. Med. *29*, 104–114. https://doi.org/10.1038/s41591-022-02128-z.

213. Kageyama, S., Ikeda, H., Miyahara, Y., Imai, N., Ishihara, M., Saito, K., Sugino, S., Ueda, S., Ishikawa, T., Kokura, S., et al. (2015). Adoptive Transfer of MAGE-A4 T-cell Receptor Gene-Transduced Lymphocytes in Patients with Recurrent Esophageal Cancer. Clin. Cancer Res. *21*, 2268–2277. https://doi.org/10.1158/1078-0432.CCR-14-1559.

214. D'Angelo, S.P., Van Tine, B.A., Attia, S., Blay, J.-Y., Strauss, S.J., Valverde Morales, C.M., Abdul Razak, A.R., Van Winkle, E., Trivedi, T., Biswas, S., et al. (2021). SPEARHEAD-1: A phase 2 trial of afamitresgene autoleucel (Formerly ADP-A2M4) in patients with advanced synovial sarcoma or myxoid/round cell liposarcoma. J. Clin. Orthod. *39*, 11504. https://doi.org/10.1200/JCO.2021.39.15_suppl.11504.

215. Anderson, V.E., Brilha, S.S., Weber, A.M., Pachnio, A., Wiedermann, G.E., Dauleh, S., Ahmed, T., Pope, G.R., Quinn, L.L., Docta, R.Y., et al. (2023). Enhancing Efficacy of TCR-engineered CD4 + T Cells Via Coexpression of CD8α. J. Immunother. *46*, 132–144. https://doi.org/10.1097/CJI.0000000000000456.

216. Nin, D.S., and Deng, L.-W. (2023). Biology of Cancer-Testis Antigens and Their Therapeutic Implications in Cancer. Cells *12*, 926. https://doi.org/10.3390/cells12060926.

217. Zhang, H., Yao, W., Zhang, M., Lu, Y., Tang, J., Jiang, M., Mou, X., You, G., and Liang, X. (2021). TTK inhibitor promotes radiosensitivity of liver cancer cells through p21. Biochem. Biophys. Res. Commun. *550*, 84–91. https://doi.org/10.1016/j.bbrc.2021.01.089.

218. Naik, A., Thomas, R., Al-Khadairi, G., Bacha, R., Hendrickx, W., and Decock, J. (2021). Cancer testis antigen PRAME: An anti-cancer target with immunomodulatory potential. J. Cell Mol. Med. *25*, 10376–10388. https://doi.org/10.1111/jcmm.16967.

219. Wang, G., Chen, B., Su, Y., Qu, N., Zhou, D., and Zhou, W. (2023). CEP55 as a Promising Immune Intervention Marker to Regulate Tumor Progression: A Pan-Cancer Analysis with Experimental Verification. Cells *12*, 2457. https://doi.org/10.3390/cells12202457.

220. Xie, X., Liang, H., Jiangting, W., Wang, Y., Ma, X., Tan, Z., Cheng, L., Luo, Z., and Wang, T. (2023). Cancer-testis antigen CEP55 serves as a prognostic biomarker and is correlated with immune infiltration and immunotherapy efficacy in pan-cancer. Front. Mol. Biosci. *10*, 1198557. https://doi.org/10.3389/fmolb.2023.1198557.

221. Zaki, M.S.A., Eldeen, M.A., Abdulsahib, W.K., Shati, A.A., Alqahtani, Y.A., Al-Qahtani, S.M., Otifi, H.M., Asiri, A., Hassan, H.M., Emam Mohammed Ahmed, H., et al. (2023). A Comprehensive Pan-Cancer Analysis Identifies CEP55 as a Potential Oncogene and Novel Therapeutic Target. Diagnostics *13*, 1613. https://doi.org/10.3390/diagnostics13091613.

222. Ma, H., Zhang, J., Shi, Y., Wang, Z., Nie, W., Cai, J., Huang, Y., Liu, B., Wang, X., and Lian, C. (2023). PBK correlates with prognosis, immune escape and drug response in LUAD. Sci. Rep. *13*, 20452. https://doi.org/10.1038/s41598-023-47781-7.

223. Mu, W., Xie, Y., Li, J., Yan, R., Zhang, J., Liu, Y., and Fan, Y. (2022). High expression of PDZ-binding kinase is correlated with poor prognosis and immune infiltrates in hepatocellular carcinoma. World J. Surg. Oncol. *20*, 22. https://doi.org/10.1186/s12957-021-02479-w.

224. Feng, T., Zhang, Y., Ling, S., Xu, C., Lyu, Y., Lu, T., Liu, X., Ying, L., Wan, Y., Zhong, H., and Su, D. (2021). PDZ Binding Kinase/T-LAK Cell-Derived Protein Kinase Plays an Oncogenic Role and Promotes Immune Escape in Human Tumors. JAMA Oncol. *2021*, 8892479. https://doi.org/10.1155/2021/8892479.

225. Zheng, L., Li, L., Xie, J., Jin, H., and Zhu, N. (2021). Six Novel Biomarkers for Diagnosis and Prognosis of Esophageal squamous cell carcinoma: validated by scRNA-seq and qPCR. J. Cancer *12*, 899–911. https://doi.org/10.7150/jca.50443.

226. Li, J., Sun, H., Fu, M., Zheng, Z., Xu, C., Yang, K., Liu, Y., Xuan, Z., Bai, Y., Zheng, J., et al. (2023). TOPK mediates immune evasion of renal cell carcinoma via upregulating the expression of PD-L1. iScience *26*, 107185. https://doi.org/10.1016/j.isci.2023.107185.

227. Shukla, S.A., Bachireddy, P., Schilling, B., Galonska, C., Zhan, Q., Bango, C., Langer, R., Lee, P.C., Gusenleitner, D., Keskin, D.B., et al. (2018). Cancer-Germline Antigen Expression Discriminates Clinical Outcome to CTLA-4 Blockade. Cell *173*, 624–633.e8. https://doi.org/10.1016/j.cell.2018.03.026.

228. Freiberger, S.N., Holzmann, D., Morand, G.B., Hüllner, M., Levesque, M.P., Dummer, R., Koelzer, V.H., and Rupp, N.J. (2023). Combinational expression of tumor testis antigens NY-ESO-1, MAGE-A3, and MAGE-A4 predicts response to immunotherapy in mucosal melanoma patients. J. Cancer Res. Clin. Oncol. *149*, 5645–5653. https://doi.org/10.1007/s00432-022-04514-z.

229. Lestari, B., and Utomo, R.Y. (2022). CEP55 Inhibitor: Extensive Computational Approach Defining a New Target of Cell Cycle Machinery Agent. Adv. Pharmaceut. Bull. *12*, 191–199. https://doi.org/10.34172/apb.2022.021.

230. Talapatra, S.K., Rath, O., Clayton, E., Tomasi, S., and Kozielski, F. (2016). Depsidones from Lichens as Natural Product Inhibitors of M-Phase Phosphoprotein 1, a Human Kinesin Required for Cytokinesis. J. Nat. Prod. *79*, 1576–1585. https://doi.org/10.1021/acs.jnatprod.5b00962.

231. Allali-Hassani, A., Szewczyk, M.M., Ivanochko, D., Organ, S.L., Bok, J., Ho, J.S.Y., Gay, F.P.H., Li, F., Blazer, L., Eram, M.S., et al. (2019). Discovery of a chemical probe for PRDM9. Nat. Commun. *10*, 5759. https://doi.org/10.1038/s41467-019-13652-x.

232. Gao, T., Hu, Q., Hu, X., Lei, Q., Feng, Z., Yu, X., Peng, C., Song, X., He, H., Xu, Y., et al. (2019). Novel selective TOPK inhibitor SKLB-C05 inhibits colorectal carcinoma growth and metastasis. Cancer Lett. *445*, 11–23. https://doi.org/10.1016/j.canlet.2018.12.016.

233. Matsuo, Y., Park, J.-H., Miyamoto, T., Yamamoto, S., Hisada, S., Alachkar, H., and Nakamura, Y. (2014). TOPK inhibitor induces complete tumor regression in xenograft models of human cancer through inhibition of cytokinesis. Sci. Transl. Med. *6*, 259ra145. https://doi.org/10.1126/scitranslmed.3010277.

234. Stefka, A.T., Johnson, D., Rosebeck, S., Park, J.H., Nakamura, Y., and Jakubowiak, A.J. (2020). Potent anti-myeloma activity of the TOPK inhibitor OTS514 in preclinical models. Cancer Med. *9*, 324–334. https://doi.org/10.1002/cam4.2695.

235. de Boussac, H., Bruyer, A., Jourdan, M., Maes, A., Robert, N., Gourzones, C., Vincent, L., Seckinger, A., Cartron, G., Hose, D., et al. (2020). Kinome expression profiling to target new therapeutic avenues in multiple myeloma. Haematologica *105*, 784–795. https://doi.org/10.3324/haematol.2018.208306.

236. Zhang, Y., Fang, M., Li, S., Xu, H., Ren, J., Tu, L., Zuo, B., Yao, W., and Liang, G. (2022). BTApep-TAT peptide inhibits ADP-ribosylation of BORIS to induce DNA damage in cancer. Mol. Cancer *21*, 158. https://doi.org/10.1186/s12943-022-01621-w.

237. Song, Y., Li, C., Fang, M., Ren, J., Fang, J., Wang, X., and Zhang, Y. (2019). Abstract 1418: Brother of regulator of imprinted sites (BORIS) inhibits cisplatin induced DNA damage in non-small-cell lung cancer. Cancer Res. *79*, 1418. https://doi.org/10.1158/1538-7445.AM2019-1418.

238. Wang, D., Chen, Z., Lin, F., Wang, Z., Gao, Q., Xie, H., Xiao, H., Zhou, Y., Zhang, F., Ma, Y., et al. (2018). OIP5 Promotes Growth, Metastasis and Chemoresistance to Cisplatin in Bladder Cancer Cells. J. Cancer *9*, 4684–4695. https://doi.org/10.7150/jca.27381.

239. Kim, T.W., Lee, S.J., Park, Y.J., Park, S.Y., Oh, B.M., Park, Y.S., Kim, B.Y., Lee, Y.H., Cho, H.J., Yoon, S.R., et al. (2017). Opa-interacting protein 5 modulates docetaxel-induced cell death via regulation of mitophagy in gastric cancer. Tumour Biol. *39*, 1010428317733985. https://doi.org/10.1177/1010428317733985.

240. Lu, H., Zhang, Q., He, S., Liu, S., Xie, Z., Li, X., and Huang, Y. (2021). Reduction-Sensitive Fluorinated-Pt(IV) Universal Transfection Nanoplatform Facilitating CT45-Targeted CRISPR/dCas9 Activation for Synergistic and Individualized Treatment of Ovarian Cancer. Small *17*, e2102494. https://doi.org/10.1002/smll.202102494.

241. Wang, Z., Mao, J.W., Liu, G.Y., Wang, F.G., Ju, Z.S., Zhou, D., and Wang, R.Y. (2019). MicroRNA-372 enhances radiosensitivity while inhibiting cell invasion and metastasis in nasopharyngeal carcinoma through activating the PBK-dependent p53 signaling pathway. Cancer Med. *8*, 712–728. https://doi.org/10.1002/cam4.1924.

OMTON, Volume *32*

Supplemental information

Cancer testis antigens: Emerging therapeutic targets leveraging genomic instability in cancer

Adviti Naik, Boucif Lattab, Hanan Qasem, and Julie Decock

Supplemental material

Supplemental References, related to Table S1.

[S1] Fan, R., Huang, W., Luo, B., Zhang, Q.M., Xiao, S.W., and Xie, X.X. (2015). Cancer testis antigen OY-TES-1: analysis of protein expression in ovarian cancer with tissue microarrays. Eur J Gynaecol Oncol 36, 298–303.

[S2] Fu, J., Luo, B., Guo, W.W., Zhang, Q.M., Shi, L., Hu, Q.P., Chen, F., Xiao, S.W., and Xie, X.X. (2015). Down-regulation of cancer/testis antigen OY-TES-1 attenuates malignant behaviors of hepatocellular carcinoma cells in vitro. Int J Clin Exp Pathol 8, 7786–7797.

[S3] Li, X., Yan, J., Fan, R., Luo, B., Zhang, Q., Lin, Y., Zhou, S., Luo, G., Xie, X., and Xiao, S. (2017). Serum immunoreactivity of cancer/testis antigen OY-TES-1 and its tissues expression in glioma. Oncol Lett 13, 3080–3086. 10.3892/ol.2017.5799.

[S4] Luo, B., Yun, X., Fan, R., Lin, Y.-D., He, S.-J., Zhang, Q.-M., Mo, F.-R., Chen, F., Xiao, S.-W., and Xie, X.-X. (2013). Cancer testis antigen OY-TES-1 expression and serum immunogenicity in colorectal cancer: its relationship to clinicopathological parameters. Int J Clin Exp Pathol 6, 2835–2845.

[S5] Luo, B., Yun, X., Li, J., Fan, R., Guo, W.-W., Liu, C., Lin, Y., Ge, Y.-Y., Zeng, X., Bi, S.-Q., et al. (2020). Cancer-testis Antigen OY-TES-1 Expression and Immunogenicity in Hepatocellular Carcinoma. Curr Med Sci 40, 719–728. 10.1007/s11596-020-2241-x.

[S6] Ono, T., Kurashige, T., Harada, N., Noguchi, Y., Saika, T., Niikawa, N., Aoe, M., Nakamura, S., Higashi, T., Hiraki, A., et al. (2001). Identification of proacrosin binding protein sp32 precursor as a human cancer/testis antigen. Proc Natl Acad Sci U S A 98, 3282–3287. 10.1073/pnas.041625098.

[S7] Whitehurst, A.W., Xie, Y., Purinton, S.C., Cappell, K.M., Swanik, J.T., Larson, B., Girard, L., Schorge, J.O., and White, M.A. (2010). Tumor antigen acrosin binding protein normalizes mitotic spindle function to promote cancer cell proliferation. Cancer Res 70, 7652–7661. 10.1158/0008-5472.CAN-10-0840.

[S8] Ciro, M., Prosperini, E., Quarto, M., Grazini, U., Walfridsson, J., McBlane, F., Nucifero, P., Pacchiana, G., Capra, M., Christensen, J., et al. (2009). ATAD2 is a novel cofactor for MYC, overexpressed and amplified in aggressive tumors. Cancer Res 69, 8491–8498. 10.1158/0008-5472.CAN-09-2131.

[S9] Duan, Z., Andrews, N.P., Chen, C.Z., Fan, M., Wang, J., Shen, J., Li, J.J., and Chen, H.W. (2020). Targeting bromodomain protein ANCCA/ATAD2 enhances the efficacy of DNA-damaging chemotherapy agents and radiation. Oncol Rep 43, 318–327. 10.3892/or.2019.7418.

[S10] Dutta, M., Mohapatra, D., Mohapatra, A.P., Senapati, S., and Roychowdhury, A. (2022). ATAD2 suppression enhances the combinatorial effect of gemcitabine and radiation in pancreatic cancer cells. Biochem Biophys Res Commun 635, 179–186. 10.1016/j.bbrc.2022.10.021.

[S11] Hou, M., Huang, R., Song, Y., Feng, D., Jiang, Y., and Liu, M. (2016). ATAD2 overexpression is associated with progression and prognosis in colorectal cancer. Jpn J Clin Oncol 46, 222–227. 10.1093/jjco/hyv195.

[S12] Hwang, H.W., Ha, S.Y., Bang, H., and Park, C.-K. (2015). ATAD2 as a Poor Prognostic Marker for Hepatocellular Carcinoma after Curative Resection. Cancer Res Treat 47, 853–861. 10.4143/crt.2014.177.

[S13] Kalashnikova, E.V., Revenko, A.S., Gemo, A.T., Andrews, N.P., Tepper, C.G., Zou, J.X., Cardiff, R.D., Borowsky, A.D., and Chen, H.-W. (2010). ANCCA/ATAD2 overexpression identifies breast cancer patients with poor prognosis, acting to drive proliferation and survival of triple-negative cells through control of B-Myb and EZH2. Cancer Res 70, 9402–9412. 10.1158/0008-5472.CAN-10-1199.

[S14] Koo, S.J., Fernandez-Montalvan, A.E., Badock, V., Ott, C.J., Holton, S.J., von Ahsen, O., Toedling, J., Vittori, S., Bradner, J.E., and Gorjanacz, M. (2016). ATAD2 is an epigenetic reader of newly synthesized histone marks during DNA replication. Oncotarget 7, 70323–70335. 10.18632/oncotarget.11855.

[S15] Liu, Q., Liu, H., Li, L., Dong, X., Ru, X., Fan, X., Wen, T., and Liu, J. (2020). ATAD2 predicts poor outcomes in patients with ovarian cancer and is a marker of proliferation. Int J Oncol 56, 219–231. 10.3892/ijo.2019.4913.

[S16] Liu, X., Zhou, Y., Liu, X., Peng, A., Gong, H., Huang, L., Ji, K., Petersen, R.B., Zheng, L., and Huang, K. (2014). MPHOSPH1: a potential therapeutic target for hepatocellular carcinoma. Cancer Res 74, 6623–6634. 10.1158/0008-5472.CAN-14-1279.

[S17] Revenko, A.S., Kalashnikova, E.V., Gemo, A.T., Zou, J.X., and Chen, H.-W. (2010). Chromatin loading of E2F-MLL complex by cancer-associated coregulator ANCCA via reading a specific histone mark. Mol Cell Biol 30, 5260–5272. 10.1128/MCB.00484-10.

[S18] Shang, P., Meng, F., Liu, Y., and Chen, X. (2015). Overexpression of ANCCA/ATAD2 in endometrial carcinoma and its correlation with tumor progression and poor prognosis. Tumour Biol 36, 4479–4485. 10.1007/s13277-015-3089-8.

[S19] Tong, Y., Li, J., Peng, M., Qian, Q., Shi, W., Chen, Z., and Liu, B. (2022). ATAD2 drives colorectal cancer progression by regulating TRIM25 expression via a positive feedback loop with E2F transcriptional factors. Biochem Biophys Res Commun 594, 146–152. 10.1016/j.bbrc.2022.01.036.

[S20] Wang, D., Pan, Y., Hao, T., Chen, Y., Qiu, S., Chen, L., and Zhao, J. (2016). Clinical and Prognostic Significance of ANCCA in Squamous Cell Lung Carcinoma Patients. Arch Med Res 47, 89–95. 10.1016/j.arcmed.2016.04.001.

[S21] Zhang, M.-J., Zhang, C.-Z., Du, W.-J., Yang, X.-Z., and Chen, Z.-P. (2016). ATAD2 is overexpressed in gastric cancer and serves as an independent poor prognostic biomarker. Clin Transl Oncol 18, 776–781. 10.1007/s12094-015-1430-8.

[S22] Zhang, Y., Sun, Y., Li, Y., Fang, Z., Wang, R., Pan, Y., Hu, H., Luo, X., Ye, T., Li, H., et al. (2013). ANCCA protein expression is a novel independent poor prognostic marker in surgically resected lung adenocarcinoma. Ann Surg Oncol 20 Suppl 3, S577-582. 10.1245/s10434-013-3027-1.

[S23] Zheng, L., Li, T., Zhang, Y., Guo, Y., Yao, J., Dou, L., and Guo, K. (2015). Oncogene ATAD2 promotes cell proliferation, invasion and migration in cervical cancer. Oncol Rep 33, 2337–2344. 10.3892/or.2015.3867.

[S24] Chandra, D., Choy, G., Deng, X., Bhatia, B., Daniel, P., and Tang, D.G. (2004). Association of active caspase 8 with the mitochondrial membrane during apoptosis: potential roles in cleaving BAP31 and caspase 3 and mediating mitochondrion-endoplasmic reticulum cross talk in etoposide-induced cell death. Mol Cell Biol 24, 6592–6607. 10.1128/MCB.24.15.6592-6607.2004.

[S25] Chen, J., Guo, H., Jiang, H., Namusamba, M., Wang, C., Lan, T., Wang, T., and Wang, B. (2019). A BAP31 intrabody induces gastric cancer cell death by inhibiting p27(kip1) proteasome degradation. Int J Cancer 144, 2051–2062. 10.1002/ijc.31930.

[S26] Grimm, S. (2012). The ER-mitochondria interface: the social network of cell death. Biochim Biophys Acta 1823, 327–334. 10.1016/j.bbamcr.2011.11.018.

[S27] Jiang, X., Li, G., Zhu, B., Zang, J., Lan, T., Jiang, R., and Wang, B. (2023). p20BAP31 induces cell apoptosis via both AIF caspase-independent and the ROS/JNK mitochondrial pathway in colorectal cancer. Cell Mol Biol Lett 28, 25. 10.1186/s11658-023-00434-z.

[S28] Li, T., Hao, Z., Tang, Z., Li, C., Cheng, L., Wang, T., Zhu, X., He, Y., Huang, Y., and Wang, B. (2022). BAP31 Regulates Wnt Signaling to Modulate Cell Migration in Lung Cancer. Front Oncol 12, 859195. 10.3389/fonc.2022.859195.

[S29] Liang, H., Dong, J., Cheng, Z., Li, Q., Feng, D., and Ling, B. (2021). B-cell receptor-associated protein 31 promotes migration and invasion in ovarian cancer cells. Exp Ther Med 22, 858. 10.3892/etm.2021.10290.

[S30] Liu, T., Yu, J., Ge, C., Zhao, F., Miao, C., Jin, W., Su, Y., Geng, Q., Chen, T., Xie, H., et al. (2021). B-Cell Receptor-Associated Protein 31 Promotes Metastasis via AKT/beta-Catenin/Snail Pathway in Hepatocellular Carcinoma. Front Mol Biosci 8, 656151. 10.3389/fmolb.2021.656151.

[S31] Ma, C., Jin, R.M., Chen, K.J., Hao, T., Li, B.S., Zhao, D.H., and Jiang, H. (2018). Low expression of B-Cell-Associated protein 31 is associated with unfavorable prognosis in human colorectal cancer. Pathol Res Pract 214, 661–666. 10.1016/j.prp.2018.03.023.

[S32] Quistgaard, E.M. (2021). BAP31: Physiological functions and roles in disease. Biochimie 186, 105–129. 10.1016/j.biochi.2021.04.008.

[S33] Sun, M., Liu, X., Wei, W., Ge, N., Luo, S., Shen, S., and Ge, R. (2022). BAP31 Promotes Proliferation, Invasion, and Metastasis of Liver Cancer Cells via Activating PI3K/AKT Pathway. J Healthc Eng 2022, 7686728. 10.1155/2022/7686728.

[S34] Tan, N., Liu, Q., Liu, X., Gong, Z., Zeng, Y., Pan, G., Xu, Q., and He, S. (2016). Low expression of B-cell-associated protein 31 in human primary hepatocellular carcinoma correlates with poor prognosis. Histopathology 68, 221–229. 10.1111/his.12738.

[S35] Wang, A., Zhang, Y., and Cao, P. (2019). Inhibition of BAP31 expression inhibits cervical cancer progression by suppressing metastasis and inducing intrinsic and extrinsic apoptosis. Biochem Biophys Res Commun 508, 499–506. 10.1016/j.bbrc.2018.11.017.

[S36] Wang, B., Pelletier, J., Massaad, M.J., Herscovics, A., and Shore, G.C. (2004). The yeast split-ubiquitin membrane protein two-hybrid screen identifies BAP31 as a regulator of the turnover of endoplasmic reticulum-associated protein tyrosine phosphatase-like B. Mol Cell Biol 24, 2767–2778. 10.1128/MCB.24.7.2767-2778.2004.

[S37] Xu, K., Han, B., Bai, Y., Ma, X.Y., Ji, Z.N., Xiong, Y., Miao, S.K., Zhang, Y.Y., and Zhou, L.M. (2019). MiR-451a suppressing BAP31 can inhibit proliferation and increase apoptosis through inducing ER stress in colorectal cancer. Cell Death Dis 10, 152. 10.1038/s41419-019-1403-x.

[S38] Yang, S., Sun, Y., Jiang, D., Wang, J., Dang, E., Li, Z., Zhou, J., Lu, Y., Shi, J., Tao, L., et al. (2021). MiR-362 suppresses cervical cancer progression via directly targeting BAP31 and activating TGFbeta/Smad pathway. Cancer Med 10, 305–316. 10.1002/cam4.3601.

[S39] Zhang, X., Jiang, D., Yang, S., Sun, Y., Liu, Y., Shi, J., Hu, C., Pan, J., Liu, T., Jin, B., et al. (2020). BAP31 Promotes Tumor Cell Proliferation by Stabilizing SERPINE2 in Hepatocellular Carcinoma. Front Cell Dev Biol 8, 607906. 10.3389/fcell.2020.607906.

[S40] Dougherty, C.J., Ichim, T.E., Liu, L., Reznik, G., Min, W.-P., Ghochikyan, A., Agadjanyan, M.G., and Reznik, B.N. (2008). Selective apoptosis of breast cancer cells by siRNA targeting of BORIS. Biochem Biophys Res Commun 370, 109–112. 10.1016/j.bbrc.2008.03.040.

[S41] He, J., Huang, Y., Liu, Z., Zhao, R., Liu, Q., Wei, L., Yu, X., Li, B., and Qin, Y. (2017). Hypomethylation of BORIS is a promising prognostic biomarker in hepatocellular carcinoma. Gene 629, 29–34. 10.1016/j.gene.2017.07.077.

[S42] Martin-Kleiner, I. (2012). BORIS in human cancers -- a review. Eur J Cancer 48, 929–935. 10.1016/j.ejca.2011.09.009.

[S43] Okabayashi, K., Fujita, T., Miyazaki, J., Okada, T., Iwata, T., Hirao, N., Noji, S., Tsukamoto, N., Goshima, N., Hasegawa, H., et al. (2012). Cancer-testis antigen BORIS is a novel prognostic marker for patients with esophageal cancer. Cancer Sci 103, 1617–1624. 10.1111/j.1349-7006.2012.02355.x.

[S44] Zhang, Y., Fang, M., Song, Y., Ren, J., Fang, J., and Wang, X. (2017). Brother of Regulator of Imprinted Sites (BORIS) suppresses apoptosis in colorectal cancer. Sci Rep 7, 40786. 10.1038/srep40786.

[S45] Zhang, Y., Song, Y., Li, C., Ren, J., Fang, M., Fang, J., and Wang, X. (2020). Brother of regulator of imprinted sites inhibits cisplatin-induced DNA damage in non-small cell lung cancer. Oncol Lett 20, 251. 10.3892/ol.2020.12114.

[S46] Zuo, B., Yao, W., Fang, M., Ren, J., Tu, L., Fan, R., and Zhang, Y. (2023). Boris knockout eliminates AOM/DSS-induced in situ colorectal cancer by suppressing DNA damage repair and inflammation. Cancer Sci 114, 1972–1985. 10.1111/cas.15732.

[S47] Bogdanov, K.V., Merzlikina, O.V., Mirolyubova, Y.V., Girshova, L.L., Lomaia, E.G., and Zaritskey, A.Y. (2021). CASC5 Gene Expression Changes Correlate with Targeted Mutations in Leukemia. Molecular Biology 55, 121–132. 10.1134/S0026893321010027.

[S48] Cheeseman, I.M., Hori, T., Fukagawa, T., and Desai, A. (2008). KNL1 and the CENP-H/I/K complex coordinately direct kinetochore assembly in vertebrates. Mol Biol Cell 19, 587–594. 10.1091/mbc.e07-10-1051.

[S49] Cui, Y., Zhang, C., Ma, S., Guo, W., Cao, W., and Guan, F. (2020). CASC5 is a potential tumour driving gene in lung adenocarcinoma. Cell Biochem Funct 38, 733–742. 10.1002/cbf.3540.

[S50] Rosenberg, J.S., Cross, F.R., and Funabiki, H. (2011). KNL1/Spc105 recruits PP1 to silence the spindle assembly checkpoint. Curr Biol 21, 942–947. 10.1016/j.cub.2011.04.011.

[S51] Singh, P.K., Bhatt, M.L.B., Singh, P., Rath, S.K., Dalela, D., and Goel, M.M. (2021). CASC5 is a potential cancer-testis gene in human urinary bladder transitional cell carcinoma. Drug Discov Ther 15, 331–336. 10.5582/ddt.2021.01108.

[S52] Lee, S.N., Hong, K.M., Seong, Y.S., and Kwak, S.J. (2020). Ectopic Overexpression of Coiled-Coil Domain Containing 110 Delays G2/M Entry in U2-OS Cells. Dev Reprod 24, 101–111. 10.12717/DR.2020.24.2.101.

[S53] Monji, M., Nakatsura, T., Senju, S., Yoshitake, Y., Sawatsubashi, M., Shinohara, M., Kageshita, T., Ono, T., Inokuchi, A., and Nishimura, Y. (2004). Identification of a novel human cancer/testis

antigen, KM-HN-1, recognized by cellular and humoral immune responses. Clin Cancer Res 10, 6047–6057. 10.1158/1078-0432.CCR-04-0475.
[S54] Park, H.J., Seo, H.J., Kim, H.W., Kim, J.S., Hwang, S.Y., and Seong, Y.S. (2007). The centrosomal localization of KM-HN-1 (MGC33607) depends on the leucine zipper motif and the C-terminal coiled-coil domain. Exp Mol Med 39, 828–838. 10.1038/emm.2007.90.
[S55] Du, T., Yi, S., Wang, Y., Zhao, Q., Ma, P., and Jiang, W. (2022). Circular RNA_0120376 regulates microRNA-148b-3 and centrosomal protein 55 to promote non-small cell lung cancer development. Bioengineered 13, 11844–11855. 10.1080/21655979.2022.2052647.
[S56] Jeffery, J., Sinha, D., Srihari, S., Kalimutho, M., and Khanna, K.K. (2016). Beyond cytokinesis: the emerging roles of CEP55 in tumorigenesis. Oncogene 35, 683–690. 10.1038/onc.2015.128.
[S57] Jiang, W., Wang, Z., and Jia, Y. (2017). CEP55 overexpression predicts poor prognosis in patients with locally advanced esophageal squamous cell carcinoma. Oncol Lett 13, 236–242. 10.3892/ol.2016.5414.
[S58] Kalimutho, M., Sinha, D., Jeffery, J., Nones, K., Srihari, S., Fernando, W.C., Duijf, P.H., Vennin, C., Raninga, P., Nanayakkara, D., et al. (2018). CEP55 is a determinant of cell fate during perturbed mitosis in breast cancer. EMBO Mol Med 10. 10.15252/emmm.201708566.
[S59] Li, G.-S., Zhang, W., Huang, W.-Y., He, R.-Q., Huang, Z.-G., Gan, X.-Y., Yang, Z., Dang, Y.-W., Kong, J.-L., Zhou, H.-F., et al. (2023). CEP55: an immune-related predictive and prognostic molecular biomarker for multiple cancers. BMC Pulm Med 23, 166. 10.1186/s12890-023-02452-1.
[S60] Lin, K., Zhu, X., Luo, C., Bu, F., Zhu, J., and Zhu, Z. (2021). Data mining combined with experiments to validate CEP55 as a prognostic biomarker in colorectal cancer. Immun Inflamm Dis 9, 167–182. 10.1002/iid3.375.
[S61] Pei, L., Dong, C., Wang, Y., Lv, X., Jia, G., and Zhang, A. (2022). Circular RNA circSDHC (hsa_circ_0015004) regulates tumor growth and angiogenesis via regulating centrosomal protein 55 expression in renal cell carcinoma. Histol Histopathol 37, 971–983. 10.14670/HH-18-467.
[S62] Qi, J., Liu, G., and Wang, F. (2018). High levels of centrosomal protein 55 expression is associated with poor clinical prognosis in patients with cervical cancer. Oncol Lett 15, 9347–9352. 10.3892/ol.2018.8448.
[S63] Sinha, D., Nag, P., Nanayakkara, D., Duijf, P.H.G., Burgess, A., Raninga, P., Smits, V.A.J., Bain, A.L., Subramanian, G., Wall, M., et al. (2020). Cep55 overexpression promotes genomic instability and tumorigenesis in mice. Commun Biol 3, 593. 10.1038/s42003-020-01304-6.
[S64] van der Horst, A., Simmons, J., and Khanna, K.K. (2009). Cep55 stabilization is required for normal execution of cytokinesis. Cell Cycle 8, 3742–3749. 10.4161/cc.8.22.10047.
[S65] Wang, G., Chen, B., Su, Y., Qu, N., Zhou, D., and Zhou, W. (2023). CEP55 as a Promising Immune Intervention Marker to Regulate Tumor Progression: A Pan-Cancer Analysis with Experimental Verification. Cells 12, 2457. 10.3390/cells12202457.
[S66] Yan, S.-M., Liu, L., Gu, W.-Y., Huang, L.-Y., Yang, Y., Huang, Y.-H., and Luo, R.-Z. (2021). CEP55 Positively Affects Tumorigenesis of Esophageal Squamous Cell Carcinoma and Is Correlated with Poor Prognosis. J Oncol 2021, 8890715. 10.1155/2021/8890715.
[S67] Yang, C., Yang, Y., Wang, W., Zhou, W., Zhang, X., Xiao, Y., and Zhang, H. (2022). CEP55 3'-UTR promotes epithelial-mesenchymal transition and enhances tumorigenicity of bladder cancer cells by acting as a ceRNA regulating miR-497-5p. Cell Oncol (Dordr) 45, 1217–1236. 10.1007/s13402-022-00712-6.
[S68] Yang, L., He, Y., Zhang, Z., and Wang, W. (2020). Upregulation of CEP55 Predicts Dismal Prognosis in Patients with Liver Cancer. Biomed Res Int 2020, 4139320. 10.1155/2020/4139320.
[S69] Zhang, X., Xu, Q., Li, E., Shi, T., and Chen, H. (2022). CEP55 predicts the poor prognosis and promotes tumorigenesis in endometrial cancer by regulating the Foxo1 signaling. Mol Cell Biochem. 10.1007/s11010-022-04607-w.
[S70] Chen, Y.-T., Hsu, M., Lee, P., Shin, S.J., Mhawech-Fauceglia, P., Odunsi, K., Altorki, N.K., Song, C.-J., Jin, B.-Q., Simpson, A.J., et al. (2009). Cancer/testis antigen CT45: analysis of mRNA and protein expression in human cancer. Int J Cancer 124, 2893–2898. 10.1002/ijc.24296.
[S71] Chen, Y.T., Ross, D.S., Chiu, R., Zhou, X.K., Chen, Y.Y., Lee, P., Hoda, S.A., Simpson, A.J., Old, L.J., Caballero, O., et al. (2011). Multiple cancer/testis antigens are preferentially expressed in hormone-receptor negative and high-grade breast cancers. PLoS One 6, e17876. 10.1371/journal.pone.0017876.

[S72] Suzuki, I., Yoshida, S., Tabu, K., Kusunoki, S., Matsumura, Y., Izumi, H., Asanoma, K., Yagi, H., Onoyama, I., Sonoda, K., et al. (2021). YBX2 and cancer testis antigen 45 contribute to stemness, chemoresistance and a high degree of malignancy in human endometrial cancer. Sci Rep 11, 4220. 10.1038/s41598-021-83200-5.

[S73] Vlasenkova, R., Konysheva, D., Nurgalieva, A., and Kiyamova, R. (2023). Characterization of Cancer/Testis Antigens as Prognostic Markers of Ovarian Cancer. Diagnostics (Basel) 13, 3092. 10.3390/diagnostics13193092.

[S74] Bi, S.Q., Peng, Y., Wei, Z.D., Yao, S.Z., Luo, B., Ge, Y.Y., Xie, X.X., Nong, W.X., Liu, C., Xiao, S.W., et al. (2022). FMR1NB Involved in Glioma Tumorigenesis Is a Promising Target for Prognosis and Therapy. Curr Med Sci 42, 803–816. 10.1007/s11596-022-2586-4.

[S75] Cappell, K.M., Sinnott, R., Taus, P., Maxfield, K., Scarbrough, M., and Whitehurst, A.W. (2012). Multiple cancer testis antigens function to support tumor cell mitotic fidelity. Mol Cell Biol 32, 4131–4140. 10.1128/MCB.00686-12.

[S76] Kim, Y.D., Park, H.R., Song, M.H., Shin, D.H., Lee, C.H., Lee, M.K., and Lee, S.Y. (2012). Pattern of cancer/testis antigen expression in lung cancer patients. Int J Mol Med 29, 656–662. 10.3892/ijmm.2012.896.

[S77] Lee, S.Y., Obata, Y., Yoshida, M., Stockert, E., Williamson, B., Jungbluth, A.A., Chen, Y.T., Old, L.J., and Scanlan, M.J. (2003). Immunomic analysis of human sarcoma. Proc Natl Acad Sci U S A 100, 2651–2656. 10.1073/pnas.0437972100.

[S78] Park, J.H., Song, M.H., Lee, C.H., Lee, M.K., Park, Y.M., Old, L., and Lee, S.Y. (2011). Expression of the human cancer/testis antigen NY-SAR-35 is activated by CpG island hypomethylation. Biotechnol Lett 33, 1085–1091. 10.1007/s10529-011-0559-y.

[S79] Song, M.H., Kim, Y.R., Bae, J.H., Shin, D.H., and Lee, S.Y. (2017). A cancer/testis antigen, NY-SAR-35, induces EpCAM, CD44, and CD133, and activates ERK in HEK293 cells. Biochem Biophys Res Commun 484, 298–303. 10.1016/j.bbrc.2017.01.105.

[S80] Song, M.H., Kim, Y.R., Lee, J.W., Lee, C.H., and Lee, S.Y. (2016). Cancer/testis antigen NY-SAR-35 enhances cell proliferation, migration, and invasion. Int J Oncol 48, 569–576. 10.3892/ijo.2015.3264.

[S81] Bissanum, R., Kamolphiwong, R., Navakanitworakul, R., and Kanokwiroon, K. (2022). Integrated bioinformatic analysis of potential biomarkers of poor prognosis in triple-negative breast cancer. Transl Cancer Res 11, 3039–3049. 10.21037/tcr-22-662.

[S82] Chen, B., Tang, H., Chen, X., Zhang, G., Wang, Y., Xie, X., and Liao, N. (2019). Transcriptomic analyses identify key differentially expressed genes and clinical outcomes between triple-negative and non-triple-negative breast cancer. Cancer Manag Res 11, 179–190. 10.2147/CMAR.S187151.

[S83] El-Botty, R., Vacher, S., Mainguené, J., Briaux, A., Ibadioune, S., Dahmani, A., Montaudon, E., Nemati, F., Huguet, L., Sourd, L., et al. (2023). HORMAD1 overexpression predicts response to anthracycline-cyclophosphamide and survival in triple-negative breast cancers. Mol Oncol 17, 2017–2028. 10.1002/1878-0261.13412.

[S84] Gantchev, J., Messina-Pacheco, J., Martínez Villarreal, A., Ramchatesingh, B., Lefrançois, P., Xie, P., Amar, L., Xu, H.H., Raveendra, K., Sikorski, D., et al. (2023). Ectopically Expressed Meiosis-Specific Cancer Testis Antigen HORMAD1 Promotes Genomic Instability in Squamous Cell Carcinomas. Cells 12, 1627. 10.3390/cells12121627.

[S85] Gao, Y., Kardos, J., Yang, Y., Tamir, T.Y., Mutter-Rottmayer, E., Weissman, B., Major, M.B., Kim, W.Y., and Vaziri, C. (2018). The Cancer/Testes (CT) Antigen HORMAD1 promotes Homologous Recombinational DNA Repair and Radioresistance in Lung adenocarcinoma cells. Sci Rep 8, 15304. 10.1038/s41598-018-33601-w.

[S86] Herrera, L.R., Johnson, R.A., McGlynn, K., Gibbs, Z.A., Davis, A.J., and Whitehurst, A.W. (2023). The cancer testes antigen, HORMAD1, limits genomic instability in cancer cells by protecting stalled replication forks. J Biol Chem 299, 105348. 10.1016/j.jbc.2023.105348.

[S87] Kogo, H., Tsutsumi, M., Ohye, T., Inagaki, H., Abe, T., and Kurahashi, H. (2012). HORMAD1-dependent checkpoint/surveillance mechanism eliminates asynaptic oocytes. Genes to Cells 17, 439–454. 10.1111/j.1365-2443.2012.01600.x.

[S88] Liu, K., Wang, Y., Zhu, Q., Li, P., Chen, J., Tang, Z., Shen, Y., Cheng, X., Lu, L.Y., and Liu, Y. (2020). Aberrantly expressed HORMAD1 disrupts nuclear localization of MCM8-MCM9 complex and compromises DNA mismatch repair in cancer cells. Cell Death Dis 11, 519. 10.1038/s41419-020-2736-1.

[S89] Nichols, B.A., Oswald, N.W., McMillan, E.A., McGlynn, K., Yan, J., Kim, M.S., Saha, J., Mallipeddi, P.L., LaDuke, S.A., Villalobos, P.A., et al. (2018). HORMAD1 Is a Negative Prognostic Indicator in Lung Adenocarcinoma and Specifies Resistance to Oxidative and Genotoxic Stress. Cancer Res 78, 6196–6208. 10.1158/0008-5472.CAN-18-1377.

[S90] Shahzad, M.M., Shin, Y.H., Matsuo, K., Lu, C., Nishimura, M., Shen, D.Y., Kang, Y., Hu, W., Mora, E.M., Rodriguez-Aguayo, C., et al. (2013). Biological significance of HORMA domain containing protein 1 (HORMAD1) in epithelial ovarian carcinoma. Cancer Lett 330, 123–129. 10.1016/j.canlet.2012.07.001.

[S91] Tarantino, D., Walker, C., Weekes, D., Pemberton, H., Davidson, K., Torga, G., Frankum, J., Mendes-Pereira, A.M., Prince, C., Ferro, R., et al. (2022). Functional screening reveals HORMAD1-driven gene dependencies associated with translesion synthesis and replication stress tolerance. Oncogene 41, 3969–3977. 10.1038/s41388-022-02369-9.

[S92] Watkins, J., Weekes, D., Shah, V., Gazinska, P., Joshi, S., Sidhu, B., Gillett, C., Pinder, S., Vanoli, F., Jasin, M., et al. (2015). Genomic Complexity Profiling Reveals That HORMAD1 Overexpression Contributes to Homologous Recombination Deficiency in Triple-Negative Breast Cancers. Cancer Discov 5, 488–505. 10.1158/2159-8290.CD-14-1092.

[S93] Zong, B., Sun, L., Peng, Y., Wang, Y., Yu, Y., Lei, J., Zhang, Y., Guo, S., Li, K., and Liu, S. (2021). HORMAD1 promotes docetaxel resistance in triple negative breast cancer by enhancing DNA damage tolerance Corrigendum in /10.3892/or.2021.8146. Oncology Reports 46, 1–15. 10.3892/or.2021.8089.

[S94] Chen, L., Wu, Q., Xu, X., Yang, C., You, J., Chen, F., and Zeng, Y. (2021). Cancer/testis antigen LDHC promotes proliferation and metastasis by activating the PI3K/Akt/GSK-3beta-signaling pathway and the in lung adenocarcinoma. Exp Cell Res 398, 112414. 10.1016/j.yexcr.2020.112414.

[S95] Cui, Z., Chen, Y., Hu, M., Lin, Y., Zhang, S., Kong, L., and Chen, Y. (2020). Diagnostic and prognostic value of the cancer-testis antigen lactate dehydrogenase C4 in breast cancer. Clin Chim Acta 503, 203–209. 10.1016/j.cca.2019.11.032.

[S96] Cui, Z., Li, Y., Gao, Y., Kong, L., Lin, Y., and Chen, Y. (2020). Cancer-testis antigen lactate dehydrogenase C4 in hepatocellular carcinoma: a promising biomarker for early diagnosis, efficacy evaluation and prognosis prediction. Aging (Albany NY) 12, 19455–19467. 10.18632/aging.103879.

[S97] Gupta, G.S. (2012). LDH-C4: a target with therapeutic potential for cancer and contraception. Mol. Cell. Biochem. 371, 115–127. 10.1007/s11010-012-1428-2.

[S98] Koslowski, M., Tureci, O., Bell, C., Krause, P., Lehr, H.A., Brunner, J., Seitz, G., Nestle, F.O., Huber, C., and Sahin, U. (2002). Multiple splice variants of lactate dehydrogenase C selectively expressed in human cancer. Cancer Res 62, 6750–6755.

[S99] Naik, A., and Decock, J. (2022). Targeting of lactate dehydrogenase C dysregulates the cell cycle and sensitizes breast cancer cells to DNA damage response targeted therapy. Mol Oncol 16, 885–903. 10.1002/1878-0261.13024.

[S100] Naik, A., and Decock, J. (2023). Commentary: Cancer-testis antigen lactate dehydrogenase C4 as a novel biomarker of male infertility and cancer. Front Oncol 13, 1115620. 10.3389/fonc.2023.1115620.

[S101] Odet, F., Gabel, S., London, R.E., Goldberg, E., and Eddy, E.M. (2013). Glycolysis and mitochondrial respiration in mouse LDHC-null sperm. Biol Reprod 88, 95. 10.1095/biolreprod.113.108530.

[S102] Odet, F., Gabel, S.A., Williams, J., London, R.E., Goldberg, E., and Eddy, E.M. (2011). Lactate dehydrogenase C and energy metabolism in mouse sperm. Biol Reprod 85, 556–564. 10.1095/biolreprod.111.091546.

[S103] Peng, W., Chen, J., Xiao, Y., Su, G., Chen, Y., and Cui, Z. (2022). Cancer-Testis Antigen LDH-C4 in Tissue, Serum, and Serum-Derived Exosomes Serves as a Promising Biomarker in Lung Adenocarcinoma. Front Oncol 12, 912624. 10.3389/fonc.2022.912624.

[S104] Tan, H., Wang, H., Ma, J., Deng, H., He, Q., Chen, Q., and Zhang, Q. (2022). Identification of human LDHC4 as a potential target for anticancer drug discovery. Acta Pharm Sin B 12, 2348–2357. 10.1016/j.apsb.2021.12.002.

[S105] Thomas, R., Shaath, H., Naik, A., Toor, S.M., Elkord, E., and Decock, J. (2020). Identification of two HLA-A*0201 immunogenic epitopes of lactate dehydrogenase C (LDHC): potential novel targets

for cancer immunotherapy. Cancer Immunol Immunother 69, 449–463. 10.1007/s00262-020-02480-4.
[S106] Wu, J., Chen, Y., Lin, Y., Lan, F., and Cui, Z. (2022). Cancer-testis antigen lactate dehydrogenase C4 as a novel biomarker of male infertility and cancer. Front Oncol 12, 936767. 10.3389/fonc.2022.936767.
[S107] Alves, P.M.S., Lévy, N., Bouzourene, H., Viatte, S., Bricard, G., Ayyoub, M., Vuilleumier, H., Givel, J.-C.R., Halkic, N., Speiser, D.E., et al. (2007). Molecular and immunological evaluation of the expression of cancer/testis gene products in human colorectal cancer. Cancer Immunol Immunother 56, 839–847. 10.1007/s00262-006-0228-5.
[S108] Baba, T., Shiota, H., Kuroda, K., Shigematsu, Y., Ichiki, Y., Uramoto, H., Hanagiri, T., and Tanaka, F. (2013). Cancer/testis antigen expression as a predictor for epidermal growth factor receptor mutation and prognosis in lung adenocarcinoma. Eur J Cardiothorac Surg 43, 759–764. 10.1093/ejcts/ezs426.
[S109] Bandić, D., Juretić, A., Sarcević, B., Separović, V., Kujundzić-Tiljak, M., Hudolin, T., Spagnoli, G.C., Cović, D., and Samija, M. (2006). Expression and possible prognostic role of MAGE-A4, NY-ESO-1, and HER-2 antigens in women with relapsing invasive ductal breast cancer: retrospective immunohistochemical study. Croat Med J 47, 32–41.
[S110] Barrow, C., Browning, J., MacGregor, D., Davis, I.D., Sturrock, S., Jungbluth, A.A., and Cebon, J. (2006). Tumor antigen expression in melanoma varies according to antigen and stage. Clin. Cancer Res. 12, 764–771. 10.1158/1078-0432.CCR-05-1544.
[S111] Bellati, F., Napoletano, C., Tarquini, E., Palaia, I., Landi, R., Manci, N., Spagnoli, G., Rughetti, A., Panici, P.B., and Nuti, M. (2007). Cancer testis antigen expression in primary and recurrent vulvar cancer: association with prognostic factors. Eur J Cancer 43, 2621–2627. 10.1016/j.ejca.2007.08.031.
[S112] Bergeron, A., Picard, V., LaRue, H., Harel, F., Hovington, H., Lacombe, L., and Fradet, Y. (2009). High frequency of MAGE-A4 and MAGE-A9 expression in high-risk bladder cancer. Int J Cancer 125, 1365–1371. 10.1002/ijc.24503.
[S113] Cabezón, T., Gromova, I., Gromov, P., Serizawa, R., Timmermans Wielenga, V., Kroman, N., Celis, J.E., and Moreira, J.M.A. (2013). Proteomic profiling of triple-negative breast carcinomas in combination with a three-tier orthogonal technology approach identifies Mage-A4 as potential therapeutic target in estrogen receptor negative breast cancer. Mol Cell Proteomics 12, 381–394. 10.1074/mcp.M112.019786.
[S114] Chen, X., Cai, S., Wang, L., Zhang, X., Li, W., and Cao, X. (2019). Analysis of the function of MAGE-A in esophageal carcinoma by bioinformatics. Medicine (Baltimore) 98, e15774. 10.1097/MD.0000000000015774.
[S115] Chen, X., Wang, L., Yue, D., Liu, J., Huang, L., Yang, L., Cao, L., Qin, G., Li, A., Wang, D., et al. (2017). Correlation between the high expression levels of cancer-germline genes with clinical characteristics in esophageal squamous cell carcinoma. Histol Histopathol 32, 793–803. 10.14670/HH-11-847.
[S116] Chitale, D.A., Jungbluth, A.A., Marshall, D.S., Leitao, M.M., Hedvat, C.V., Kolb, D., Spagnoli, G.C., Iversen, K., and Soslow, R.A. (2005). Expression of cancer-testis antigens in endometrial carcinomas using a tissue microarray. Mod Pathol 18, 119–126. 10.1038/modpathol.3800232.
[S117] Daudi, S., Eng, K.H., Mhawech-Fauceglia, P., Morrison, C., Miliotto, A., Beck, A., Matsuzaki, J., Tsuji, T., Groman, A., Gnjatic, S., et al. (2014). Expression and immune responses to MAGE antigens predict survival in epithelial ovarian cancer. PLoS One 9, e104099. 10.1371/journal.pone.0104099.
[S118] Errington, J.A., Conway, R.M., Walsh-Conway, N., Browning, J., Freyer, C., Cebon, J., and Madigan, M.C. (2012). Expression of cancer-testis antigens (MAGE-A1, MAGE-A3/6, MAGE-A4, MAGE-C1 and NY-ESO-1) in primary human uveal and conjunctival melanoma. Br J Ophthalmol 96, 451–458. 10.1136/bjophthalmol-2011-300432.
[S119] Forghanifard, M.M., Gholamin, M., Farshchian, M., Moaven, O., Memar, B., Forghani, M.N., Dadkhah, E., Naseh, H., Moghbeli, M., Raeisossadati, R., et al. (2011). Cancer-testis gene expression profiling in esophageal squamous cell carcinoma: identification of specific tumor marker and potential targets for immunotherapy. Cancer Biol Ther 12, 191–197. 10.4161/cbt.12.3.15949.

[S120] Gao, Y., Mutter-Rottmayer, E., Greenwalt, A.M., Goldfarb, D., Yan, F., Yang, Y., Martinez-Chacin, R.C., Pearce, K.H., Tateishi, S., Major, M.B., et al. (2016). A neomorphic cancer cell-specific role of MAGE-A4 in trans-lesion synthesis. Nat Commun 7, 12105. 10.1038/ncomms12105.

[S121] Garcia-Soto, A.E., Schreiber, T., Strbo, N., Ganjei-Azar, P., Miao, F., Koru-Sengul, T., Simpkins, F., Nieves-Neira, W., Lucci, J., and Podack, E.R. (2017). Cancer-testis antigen expression is shared between epithelial ovarian cancer tumors. Gynecol Oncol 145, 413–419. 10.1016/j.ygyno.2017.03.512.

[S122] Gure, A.O., Chua, R., Williamson, B., Gonen, M., Ferrera, C.A., Gnjatic, S., Ritter, G., Simpson, A.J.G., Chen, Y.-T., Old, L.J., et al. (2005). Cancer-testis genes are coordinately expressed and are markers of poor outcome in non-small cell lung cancer. Clin. Cancer Res. 11, 8055–8062. 10.1158/1078-0432.CCR-05-1203.

[S123] Hanagiri, T., Shigematsu, Y., Shinohara, S., Takenaka, M., Oka, S., Chikaishi, Y., Nagata, Y., Baba, T., Uramoto, H., So, T., et al. (2013). Clinical significance of expression of cancer/testis antigen and down-regulation of HLA class-I in patients with stage I non-small cell lung cancer. Anticancer Res 33, 2123–2128.

[S124] Hashimoto, K., Nishimura, S., Ito, T., and Akagi, M. (2022). Clinicopathological Assessment of Cancer/Testis Antigens NY-ESO-1 and MAGE-A4 in Highly Aggressive Soft Tissue Sarcomas. Diagnostics (Basel) 12, 733. 10.3390/diagnostics12030733.

[S125] Hashimoto, K., Nishimura, S., Ito, T., Kakinoki, R., and Akagi, M. (2022). Immunohistochemical expression and clinicopathological assessment of PD-1, PD-L1, NY-ESO-1, and MAGE-A4 expression in highly aggressive soft tissue sarcomas. Eur J Histochem 66, 3393. 10.4081/ejh.2022.3393.

[S126] Hashimoto, K., Nishimura, S., Ito, T., Oka, N., Kakinoki, R., and Akagi, M. (2022). Clinicopathological assessment of cancer/testis antigens NY-ESO-1 and MAGE-A4 in osteosarcoma. Eur J Histochem 66. 10.4081/ejh.2022.3377.

[S127] Hashimoto, K., Nishimura, S., Shinyashiki, Y., Ito, T., Kakinoki, R., and Akagi, M. (2023). Involvement of NY-ESO-1 and MAGE-A4 in the pathogenesis of desmoid tumors. Medicine (Baltimore) 102, e33908. 10.1097/MD.0000000000033908.

[S128] Hong, D.S., Van Tine, B.A., Biswas, S., McAlpine, C., Johnson, M.L., Olszanski, A.J., Clarke, J.M., Araujo, D., Blumenschein, G.R., Kebriaei, P., et al. (2023). Autologous T cell therapy for MAGE-A4(+) solid cancers in HLA-A*02(+) patients: a phase 1 trial. Nat Med 29, 104–114. 10.1038/s41591-022-02128-z.

[S129] Hou, Z., Liang, X., Wang, X., Zhou, Z., and Shi, G. (2020). Myeloid-derived suppressor cells infiltration in non-small-cell lung cancer tumor and MAGE-A4 and NY-ESO-1 expression. Oncol Lett 19, 3982–3992. 10.3892/ol.2020.11497.

[S130] Hussein, Y.M., Gharib, A.F., Etewa, R.L., El-Shal, A.S., Abdel-Ghany, M.E., and Elsawy, W.H. (2011). The melanoma-associated antigen-A3, -A4 genes: relation to the risk and clinicopathological parameters in breast cancer patients. Mol Cell Biochem 351, 261–268. 10.1007/s11010-011-0734-4.

[S131] Ishihara, M., Kageyama, S., Miyahara, Y., Ishikawa, T., Ueda, S., Soga, N., Naota, H., Mukai, K., Harada, N., Ikeda, H., et al. (2020). MAGE-A4, NY-ESO-1 and SAGE mRNA expression rates and co-expression relationships in solid tumours. BMC Cancer 20, 606. 10.1186/s12885-020-07098-4.

[S132] Kakimoto, T., Matsumine, A., Kageyama, S., Asanuma, K., Matsubara, T., Nakamura, T., Iino, T., Ikeda, H., Shiku, H., and Sudo, A. (2019). Immunohistochemical expression and clinicopathological assessment of the cancer testis antigens NY-ESO-1 and MAGE-A4 in high-grade soft-tissue sarcoma. Oncol Lett 17, 3937–3943. 10.3892/ol.2019.10044.

[S133] Karimi, S., Mohammadi, F., Porabdollah, M., Mohajerani, S.A., Khodadad, K., and Nadji, S.A. (2012). Characterization of melanoma-associated antigen-a genes family differential expression in non-small-cell lung cancers. Clin Lung Cancer 13, 214–219. 10.1016/j.cllc.2011.09.007.

[S134] Kocher, T., Zheng, M., Bolli, M., Simon, R., Forster, T., Schultz-Thater, E., Remmel, E., Noppen, C., Schmid, U., Ackermann, D., et al. (2002). Prognostic relevance of MAGE-A4 tumor antigen expression in transitional cell carcinoma of the urinary bladder: a tissue microarray study. Int J Cancer 100, 702–705. 10.1002/ijc.10540.

[S135] Lüftl, M., Schuler, G., and Jungbluth, A.A. (2004). Melanoma or not? Cancer testis antigens may help. Br J Dermatol 151, 1213–1218. 10.1111/j.1365-2133.2004.06260.x.

[S136] Montoro, J.R. de M.C., Mamede, R.C.M., Neder Serafini, L., Saggioro, F.P., Figueiredo, D.L.A., Silva, W.A. da, Jungbluth, A.A., Spagnoli, G.C., and Zago, M.A. (2012). Expression of cancer-testis antigens MAGE-A4 and MAGE-C1 in oral squamous cell carcinoma. Head Neck 34, 1123–1128. 10.1002/hed.21880.
[S137] Müller-Richter, U.D.A., Dowejko, A., Driemel, O., Reuther, T., Reichert, T.E., and Kübler, A.C. (2010). Impact of MAGE-A antigens on taxane response in oral squamous cell carcinoma. Oncol Lett 1, 181–185. 10.3892/ol_00000033.
[S138] Nishikawa, H., Maeda, Y., Ishida, T., Gnjatic, S., Sato, E., Mori, F., Sugiyama, D., Ito, A., Fukumori, Y., Utsunomiya, A., et al. (2012). Cancer/testis antigens are novel targets of immunotherapy for adult T-cell leukemia/lymphoma. Blood 119, 3097–3104. 10.1182/blood-2011-09-379982.
[S139] Otte, M., Zafrakas, M., Riethdorf, L., Pichlmeier, U., Löning, T., Jänicke, F., and Pantel, K. (2001). MAGE-A gene expression pattern in primary breast cancer. Cancer Res 61, 6682–6687.
[S140] Peikert, T., Specks, U., Farver, C., Erzurum, S.C., and Comhair, S.A. (2006). Melanoma antigen A4 is expressed in non-small cell lung cancers and promotes apoptosis. Cancer Res 66, 4693–4700. 10.1158/0008-5472.CAN-05-3327.
[S141] Peng, J., Chen, H., Mou, D., Cao, J., Cong, X., Qin, L., Wei, L., Leng, X., Wang, Y., and Chen, W. (2005). Expression of cancer/testis (CT) antigens in Chinese hepatocellular carcinoma and its correlation with clinical parameters. Cancer Lett. 219, 223–232. 10.1016/j.canlet.2004.07.028.
[S142] Perez, D., Herrmann, T., Jungbluth, A.A., Samartzis, P., Spagnoli, G., Demartines, N., Clavien, P.-A., Marino, S., Seifert, B., and Jaeger, D. (2008). Cancer testis antigen expression in gastrointestinal stromal tumors: new markers for early recurrence. Int J Cancer 123, 1551–1555. 10.1002/ijc.23698.
[S143] Prasad, M.L., Jungbluth, A.A., Patel, S.G., Iversen, K., Hoshaw-Woodard, S., and Busam, K.J. (2004). Expression and significance of cancer testis antigens in primary mucosal melanoma of the head and neck. Head Neck 26, 1053–1057. 10.1002/hed.20112.
[S144] Resnick, M.B., Sabo, E., Kondratev, S., Kerner, H., Spagnoli, G.C., and Yakirevich, E. (2002). Cancer-testis antigen expression in uterine malignancies with an emphasis on carcinosarcomas and papillary serous carcinomas. Int J Cancer 101, 190–195. 10.1002/ijc.10585.
[S145] Sarcevic, B., Spagnoli, G.C., Terracciano, L., Schultz-Thater, E., Heberer, M., Gamulin, M., Krajina, Z., Oresic, T., Separovic, R., and Juretic, A. (2003). Expression of cancer/testis tumor associated antigens in cervical squamous cell carcinoma. Oncology 64, 443–449. 10.1159/000070305.
[S146] Sharma, P., Shen, Y., Wen, S., Bajorin, D.F., Reuter, V.E., Old, L.J., and Jungbluth, A.A. (2006). Cancer-testis antigens: expression and correlation with survival in human urothelial carcinoma. Clin Cancer Res 12, 5442–5447. 10.1158/1078-0432.CCR-06-0527.
[S147] Shigematsu, Y., Hanagiri, T., Shiota, H., Kuroda, K., Baba, T., Mizukami, M., So, T., Ichiki, Y., Yasuda, M., So, T., et al. (2010). Clinical significance of cancer/testis antigens expression in patients with non-small cell lung cancer. Lung Cancer 68, 105–110. 10.1016/j.lungcan.2009.05.010.
[S148] Soga, N., Hori, Y., Yamakado, K., Ikeda, H., Imai, N., Kageyama, S., Nakase, K., Yuta, A., Hayashi, N., Shiku, H., et al. (2013). Limited expression of cancer-testis antigens in renal cell carcinoma patients. Mol Clin Oncol 1, 326–330. 10.3892/mco.2012.40.
[S149] Srdelić, S., Kuzmić-Prusac, I., Spagnoli, G.C., Juretić, A., and Čapkun, V. (2019). MAGE-A4 and MAGE-A1 Immunohistochemical Expression in High-grade Endometrial Cancer. Int J Gynecol Pathol 38, 59–65. 10.1097/PGP.0000000000000470.
[S150] Trippel, A., Halling, F., Heymann, P., Ayna, M., Al-Nawas, B., and Ziebart, T. (2019). The expression of melanoma-associated antigen A (MAGE-A) in oral squamous cell carcinoma: an evaluation of the significance for tumor prognosis. Oral Maxillofac Surg 23, 343–352. 10.1007/s10006-019-00778-x.
[S151] Vital, D., Ikenberg, K., Moch, H., Roessle, M., and Huber, G.F. (2018). The expression of the cancer testis antigen MAGE A4: A favorable prognostic biomarker in salivary gland carcinomas related to low tumor grading. Laryngoscope Investig Otolaryngol 3, 182–190. 10.1002/lio2.160.
[S152] Wang, M., Li, J., Wang, L., Chen, X., Zhang, Z., Yue, D., Ping, Y., Shi, X., Huang, L., Zhang, T., et al. (2015). Combined cancer testis antigens enhanced prediction accuracy for prognosis of patients with hepatocellular carcinoma. Int J Clin Exp Pathol 8, 3513–3528.

[S153] Xiao, J., Huang, F., Li, L., Zhang, L., Xie, L., and Liu, B. (2023). Expression of four cancer-testis antigens in TNBC indicating potential universal immunotherapeutic targets. J Cancer Res Clin Oncol 149, 15003–15011. 10.1007/s00432-023-05274-0.

[S154] Yakirevich, E., Sabo, E., Lavie, O., Mazareb, S., Spagnoli, G.C., and Resnick, M.B. (2003). Expression of the MAGE-A4 and NY-ESO-1 cancer-testis antigens in serous ovarian neoplasms. Clin Cancer Res 9, 6453–6460.

[S155] Yoshida, N., Abe, H., Ohkuri, T., Wakita, D., Sato, M., Noguchi, D., Miyamoto, M., Morikawa, T., Kondo, S., Ikeda, H., et al. (2006). Expression of the MAGE-A4 and NY-ESO-1 cancer-testis antigens and T cell infiltration in non-small cell lung carcinoma and their prognostic significance. Int J Oncol 28, 1089–1098.

[S156] Zimmermann, A.-K., Imig, J., Klar, A., Renner, C., Korol, D., Fink, D., Stadlmann, S., Singer, G., Knuth, A., Moch, H., et al. (2013). Expression of MAGE-C1/CT7 and selected cancer/testis antigens in ovarian borderline tumours and primary and recurrent ovarian carcinomas. Virchows Arch 462, 565–574. 10.1007/s00428-013-1395-3.

[S157] Bhatia, N., Xiao, T.Z., Rosenthal, K.A., Siddiqui, I.A., Thiyagarajan, S., Smart, B., Meng, Q., Zuleger, C.L., Mukhtar, H., Kenney, S.C., et al. (2013). MAGE-C2 promotes growth and tumorigenicity of melanoma cells, phosphorylation of KAP1, and DNA damage repair. J Invest Dermatol 133, 759–767. 10.1038/jid.2012.355.

[S158] Bode, P.K., Barghorn, A., Fritzsche, F.R., Riener, M.-O., Kristiansen, G., Knuth, A., and Moch, H. (2011). MAGEC2 is a sensitive and novel marker for seminoma: a tissue microarray analysis of 325 testicular germ cell tumors. Mod Pathol 24, 829–835. 10.1038/modpathol.2011.6.

[S159] Chen, X., Wang, L., Liu, J., Huang, L., Yang, L., Gao, Q., Shi, X., Li, J., Li, F., Zhang, Z., et al. (2017). Expression and prognostic relevance of MAGE-A3 and MAGE-C2 in non-small cell lung cancer. Oncol Lett 13, 1609–1618. 10.3892/ol.2017.5665.

[S160] Chen, Y.-T., Cao, D., Chiu, R., and Lee, P. (2013). Chromosome X-encoded Cancer/Testis antigens are less frequently expressed in non-seminomatous germ cell tumors than in seminomas. Cancer Immun 13, 10.

[S161] Chen, Y.T., Chiu, R., Lee, P., Beneck, D., Jin, B., and Old, L.J. (2011). Chromosome X-encoded cancer/testis antigens show distinctive expression patterns in developing gonads and in testicular seminoma. Hum Reprod 26, 3232–3243. 10.1093/humrep/der330.

[S162] Condomines, M., Hose, D., Raynaud, P., Hundemer, M., De Vos, J., Baudard, M., Moehler, T., Pantesco, V., Moos, M., Schved, J.-F., et al. (2007). Cancer/testis genes in multiple myeloma: expression patterns and prognosis value determined by microarray analysis. J Immunol 178, 3307–3315. 10.4049/jimmunol.178.5.3307.

[S163] Cuffel, C., Rivals, J.-P., Zaugg, Y., Salvi, S., Seelentag, W., Speiser, D.E., Liénard, D., Monnier, P., Romero, P., Bron, L., et al. (2011). Pattern and clinical significance of cancer-testis gene expression in head and neck squamous cell carcinoma. Int. J. Cancer 128, 2625–2634. 10.1002/ijc.25607.

[S164] Curioni-Fontecedro, A., Nuber, N., Mihic-Probst, D., Seifert, B., Soldini, D., Dummer, R., Knuth, A., van den Broek, M., and Moch, H. (2011). Expression of MAGE-C1/CT7 and MAGE-C2/CT10 predicts lymph node metastasis in melanoma patients. PLoS One 6, e21418. 10.1371/journal.pone.0021418.

[S165] Curioni-Fontecedro, A., Pitocco, R., Schoenewolf, N.L., Holzmann, D., Soldini, D., Dummer, R., Calvieri, S., Moch, H., Mihic-Probst, D., and Fitsche, A. (2015). Intratumoral Heterogeneity of MAGE-C1/CT7 and MAGE-C2/CT10 Expression in Mucosal Melanoma. Biomed Res Int 2015, 432479. 10.1155/2015/432479.

[S166] de Carvalho, F., Alves, V.L.F., Braga, W.M.T., Xavier, C.V., and Colleoni, G.W.B. (2013). MAGE-C1/CT7 and MAGE-C2/CT10 are frequently expressed in multiple myeloma and can be explored in combined immunotherapy for this malignancy. Cancer Immunol Immunother 62, 191–195. 10.1007/s00262-012-1376-4.

[S167] Espantman, K.C., and O'Shea, C.C. (2010). aMAGEing new players enter the RING to promote ubiquitylation. Mol Cell 39, 835–837. 10.1016/j.molcel.2010.09.006.

[S168] Figueiredo, D.L.A., Mamede, R.C.M., Spagnoli, G.C., Silva, W.A., Zago, M., Neder, L., Jungbluth, A.A., and Saggioro, F.P. (2011). High expression of cancer testis antigens MAGE-A, MAGE-C1/CT7, MAGE-C2/CT10, NY-ESO-1, and gage in advanced squamous cell carcinoma of the larynx. Head Neck 33, 702–707. 10.1002/hed.21522.

[S169] Ghadban, T., Perez, D.R., Vashist, Y.K., Bockhorn, M., Koenig, A.M., El Gammal, A.T., Izbicki, J.R., Metzger, U., Hauswirth, F., Frosina, D., et al. (2014). Expression of cancer testis antigens CT10 (MAGE-C2) and GAGE in gastrointestinal stromal tumors. Eur J Surg Oncol 40, 1307–1312. 10.1016/j.ejso.2014.03.011.

[S170] Hao, J., Song, X., Wang, J., Guo, C., Li, Y., Li, B., Zhang, Y., and Yin, Y. (2015). Cancer-testis antigen MAGE-C2 binds Rbx1 and inhibits ubiquitin ligase-mediated turnover of cyclin E. Oncotarget 6, 42028–42039. 10.18632/oncotarget.5973.

[S171] Hodgson, A., Jungbluth, A.A., Katabi, N., Xu, B., and Downes, M.R. (2020). Evaluation of cancer testis antigen (CT10, PRAME) and MHC I expression in high-grade urothelial carcinoma of the bladder. Virchows Arch 476, 535–542. 10.1007/s00428-019-02661-2.

[S172] Hou, S., Sang, M., Zhao, L., Hou, R., and Shan, B. (2016). The expression of MAGE-C1 and MAGE-C2 in breast cancer and their clinical significance. Am J Surg 211, 142–151. 10.1016/j.amjsurg.2015.05.028.

[S173] Inaoka, R.J., Jungbluth, A.A., Baiocchi, O.C., Assis, M.C., Hanson, N.C., Frosina, D., Tassello, J., Bortoluzzo, A.B., Alves, A.C., and Colleoni, G.W. (2011). An overview of cancer/testis antigens expression in classical Hodgkin's lymphoma (cHL) identifies MAGE-A family and MAGE-C1 as the most frequently expressed antigens in a set of Brazilian cHL patients. BMC Cancer 11, 416. 10.1186/1471-2407-11-416.

[S174] Lajmi, N., Luetkens, T., Yousef, S., Templin, J., Cao, Y., Hildebrandt, Y., Bartels, K., Kroger, N., and Atanackovic, D. (2015). Cancer-testis antigen MAGEC2 promotes proliferation and resistance to apoptosis in Multiple Myeloma. Br J Haematol 171, 752–762. 10.1111/bjh.13762.

[S175] Li, B., Qian, X.-P., Pang, X.-W., Zou, W.-Z., Wang, Y.-P., Wu, H.-Y., and Chen, W.-F. (2003). HCA587 antigen expression in normal tissues and cancers: correlation with tumor differentiation in hepatocellular carcinoma. Lab Invest 83, 1185–1192. 10.1097/01.lab.0000080605.73839.96.

[S176] Li, M., Yuan, Y.-H., Han, Y., Liu, Y.-X., Yan, L., Wang, Y., and Gu, J. (2005). Expression profile of cancer-testis genes in 121 human colorectal cancer tissue and adjacent normal tissue. Clin Cancer Res 11, 1809–1814. 10.1158/1078-0432.CCR-04-1365.

[S177] Liu, Y., Cao, B., Hu, L., Ye, J., Tian, W., and He, X. (2022). The Dual Roles of MAGE-C2 in p53 Ubiquitination and Cell Proliferation Through E3 Ligases MDM2 and TRIM28. Front Cell Dev Biol 10, 922675. 10.3389/fcell.2022.922675.

[S178] Mischo, A., Kubuschok, B., Ertan, K., Preuss, K.-D., Romeike, B., Regitz, E., Schormann, C., de Bruijn, D., Wadle, A., Neumann, F., et al. (2006). Prospective study on the expression of cancer testis genes and antibody responses in 100 consecutive patients with primary breast cancer. Int. J. Cancer 118, 696–703. 10.1002/ijc.21352.

[S179] Nardiello, T., Jungbluth, A.A., Mei, A., Diliberto, M., Huang, X., Dabrowski, A., Andrade, V.C.C., Wasserstrum, R., Ely, S., Niesvizky, R., et al. (2011). MAGE-A inhibits apoptosis in proliferating myeloma cells through repression of Bax and maintenance of survivin. Clin Cancer Res 17, 4309–4319. 10.1158/1078-0432.CCR-10-1820.

[S180] Oba-Shinjo, S.M., Caballero, O.L., Jungbluth, A.A., Rosemberg, S., Old, L.J., Simpson, A.J.G., and Marie, S.K.N. (2008). Cancer-testis (CT) antigen expression in medulloblastoma. Cancer Immun 8, 7.

[S181] Riener, M.-O., Wild, P.J., Soll, C., Knuth, A., Jin, B., Jungbluth, A., Hellerbrand, C., Clavien, P.-A., Moch, H., and Jochum, W. (2009). Frequent expression of the novel cancer testis antigen MAGE-C2/CT-10 in hepatocellular carcinoma. Int J Cancer 124, 352–357. 10.1002/ijc.23966.

[S182] Scanlan, M.J., Altorki, N.K., Gure, A.O., Williamson, B., Jungbluth, A., Chen, Y.T., and Old, L.J. (2000). Expression of cancer-testis antigens in lung cancer: definition of bromodomain testis-specific gene (BRDT) as a new CT gene, CT9. Cancer Lett 150, 155–164. 10.1016/s0304-3835(99)00385-7.

[S183] Sideras, K., Bots, S.J., Biermann, K., Sprengers, D., Polak, W.G., IJzermans, J.N.M., de Man, R.A., Pan, Q., Sleijfer, S., Bruno, M.J., et al. (2015). Tumour antigen expression in hepatocellular carcinoma in a low-endemic western area. Br J Cancer 112, 1911–1920. 10.1038/bjc.2015.92.

[S184] Syed, O.N., Mandigo, C.E., Killory, B.D., Canoll, P., and Bruce, J.N. (2012). Cancer-testis and melanocyte-differentiation antigen expression in malignant glioma and meningioma. J Clin Neurosci 19, 1016–1021. 10.1016/j.jocn.2011.10.008.

[S185] von Boehmer, L., Keller, L., Mortezavi, A., Provenzano, M., Sais, G., Hermanns, T., Sulser, T., Jungbluth, A.A., Old, L.J., Kristiansen, G., et al. (2011). MAGE-C2/CT10 protein expression is an

independent predictor of recurrence in prostate cancer. PLoS One 6, e21366. 10.1371/journal.pone.0021366.
[S186] Zhao, L., Mou, D.-C., Leng, X.-S., Peng, J.-R., Wang, W.-X., Huang, L., Li, S., and Zhu, J.-Y. (2004). Expression of cancer-testis antigens in hepatocellular carcinoma. World J Gastroenterol 10, 2034–2038. 10.3748/wjg.v10.i14.2034.
[S187] Zhao, Q., Xu, W.-T., and Shalieer, T. (2016). Pilot Study on MAGE-C2 as a Potential Biomarker for Triple-Negative Breast Cancer. Dis Markers 2016, 2325987. 10.1155/2016/2325987.
[S188] Zhou, X., Yang, F., Zhang, T., Zhuang, R., Sun, Y., Fang, L., Zhang, C., Ma, Y., Huang, G., Ma, F., et al. (2013). Heterogeneous expression of CT10, CT45 and GAGE7 antigens and their prognostic significance in human breast carcinoma. Jpn J Clin Oncol 43, 243–250. 10.1093/jjco/hys236.
[S189] Gu, Y., Wang, C., Zhu, R., Yang, J., Yuan, W., Zhu, Y., Zhou, Y., Qin, N., Shen, H., Ma, H., et al. (2021). The cancer-testis gene, MEIOB, sensitizes triple-negative breast cancer to PARP1 inhibitors by inducing homologous recombination deficiency. Cancer Biol Med 18, 74–87. 10.20892/j.issn.2095-3941.2020.0071.
[S190] Luo, M., Yang, F., Leu, N.A., Landaiche, J., Handel, M.A., Benavente, R., La Salle, S., and Wang, P.J. (2013). MEIOB exhibits single-stranded DNA-binding and exonuclease activities and is essential for meiotic recombination. Nat Commun 4, 2788. 10.1038/ncomms3788.
[S191] Wang, C., Gu, Y., Zhang, K., Xie, K., Zhu, M., Dai, N., Jiang, Y., Guo, X., Liu, M., Dai, J., et al. (2016). Systematic identification of genes with a cancer-testis expression pattern in 19 cancer types. Nat Commun 7, 10499. 10.1038/ncomms10499.
[S192] Abe, T., Kohashi, K., Takemoto, J., Kinoshita, F., Eto, M., and Oda, Y. (2018). Clinicopathological Significance and Antitumor Effect of MPHOSPH1 in Testicular Germ Cell Tumor. J Cancer 9, 4440–4448. 10.7150/jca.25279.
[S193] Ansari, D., Andersson, R., Bauden, M.P., Andersson, B., Connolly, J.B., Welinder, C., Sasor, A., and Marko-Varga, G. (2015). Protein deep sequencing applied to biobank samples from patients with pancreatic cancer. J Cancer Res Clin Oncol 141, 369–380. 10.1007/s00432-014-1817-x.
[S194] Chen, J., Zhao, C.C., Chen, F.R., Feng, G.W., Luo, F., and Jiang, T. (2021). KIF20B Promotes Cell Proliferation and May Be a Potential Therapeutic Target in Pancreatic Cancer. J Oncol 2021, 5572402. 10.1155/2021/5572402.
[S195] Kanehira, M., Katagiri, T., Shimo, A., Takata, R., Shuin, T., Miki, T., Fujioka, T., and Nakamura, Y. (2007). Oncogenic role of MPHOSPH1, a cancer-testis antigen specific to human bladder cancer. Cancer Res 67, 3276–3285. 10.1158/0008-5472.CAN-06-3748.
[S196] Li, G., Xie, Z.K., Zhu, D.S., Guo, T., Cai, Q.L., and Wang, Y. (2019). KIF20B promotes the progression of clear cell renal cell carcinoma by stimulating cell proliferation. J Cell Physiol 234, 16517–16525. 10.1002/jcp.28322.
[S197] Li, T.F., Zeng, H.J., Shan, Z., Ye, R.Y., Cheang, T.Y., Zhang, Y.J., Lu, S.H., Zhang, Q., Shao, N., and Lin, Y. (2020). Overexpression of kinesin superfamily members as prognostic biomarkers of breast cancer. Cancer Cell Int 20, 123. 10.1186/s12935-020-01191-1.
[S198] Li, Z.Y., Wang, Z.X., and Li, C.C. (2019). Kinesin family member 20B regulates tongue cancer progression by promoting cell proliferation. Mol Med Rep 19, 2202–2210. 10.3892/mmr.2019.9851.
[S199] Lin, W.F., Lin, X.L., Fu, S.W., Yang, L., Tang, C.T., Gao, Y.J., Chen, H.Y., and Ge, Z.Z. (2018). Pseudopod-associated protein KIF20B promotes Gli1-induced epithelial-mesenchymal transition modulated by pseudopodial actin dynamic in human colorectal cancer. Mol Carcinog 57, 911–925. 10.1002/mc.22812.
[S200] Liu, X., Li, Y., Zhang, X., Liu, X.Y., Peng, A., Chen, Y., Meng, L., Chen, H., Zhang, Y., Miao, X., et al. (2018). Inhibition of kinesin family member 20B sensitizes hepatocellular carcinoma cell to microtubule-targeting agents by blocking cytokinesis. Cancer Sci 109, 3450–3460. 10.1111/cas.13794.
[S201] Abrieu, A., Magnaghi-Jaulin, L., Kahana, J.A., Peter, M., Castro, A., Vigneron, S., Lorca, T., Cleveland, D.W., and Labbe, J.C. (2001). Mps1 is a kinetochore-associated kinase essential for the vertebrate mitotic checkpoint. Cell 106, 83–93. 10.1016/s0092-8674(01)00410-x.
[S202] Anderhub, S.J., Mak, G.W.-Y., Gurden, M.D., Faisal, A., Drosopoulos, K., Walsh, K., Woodward, H.L., Innocenti, P., Westwood, I.M., Naud, S., et al. (2019). High Proliferation Rate and a Compromised Spindle Assembly Checkpoint Confers Sensitivity to the MPS1 Inhibitor BOS172722 in Triple-Negative Breast Cancers. Mol Cancer Ther 18, 1696–1707. 10.1158/1535-7163.MCT-18-1203.

[S203] Chan, C.Y., Chiu, D.K., Yuen, V.W., Law, C.T., Wong, B.P., Thu, K.L., Cescon, D.W., Soria-Bretones, I., Cheu, J.W., Lee, D., et al. (2022). CFI-402257, a TTK inhibitor, effectively suppresses hepatocellular carcinoma. Proc Natl Acad Sci U S A 119, e2119514119. 10.1073/pnas.2119514119.
[S204] Chen, S., Wang, J., Wang, L., Peng, H., Xiao, L., Li, C., Lin, D., and Yang, K. (2019). Silencing TTK expression inhibits the proliferation and progression of prostate cancer. Exp Cell Res 385, 111669. 10.1016/j.yexcr.2019.111669.
[S205] Choi, M., Min, Y.H., Pyo, J., Lee, C.-W., Jang, C.-Y., and Kim, J.-E. (2017). TC Mps1 12, a novel Mps1 inhibitor, suppresses the growth of hepatocellular carcinoma cells via the accumulation of chromosomal instability. Br J Pharmacol 174, 1810–1825. 10.1111/bph.13782.
[S206] Colombo, R., Caldarelli, M., Mennecozzi, M., Giorgini, M.L., Sola, F., Cappella, P., Perrera, C., Depaolini, S.R., Rusconi, L., Cucchi, U., et al. (2010). Targeting the mitotic checkpoint for cancer therapy with NMS-P715, an inhibitor of MPS1 kinase. Cancer Res 70, 10255–10264. 10.1158/0008-5472.CAN-10-2101.
[S207] Gyorffy, B., Bottai, G., Lehmann-Che, J., Keri, G., Orfi, L., Iwamoto, T., Desmedt, C., Bianchini, G., Turner, N.C., de The, H., et al. (2014). TP53 mutation-correlated genes predict the risk of tumor relapse and identify MPS1 as a potential therapeutic kinase in TP53-mutated breast cancers. Mol Oncol 8, 508–519. 10.1016/j.molonc.2013.12.018.
[S208] Huang, Y.-F., Chang, M.D.-T., and Shieh, S.-Y. (2009). TTK/hMps1 mediates the p53-dependent postmitotic checkpoint by phosphorylating p53 at Thr18. Mol Cell Biol 29, 2935–2944. 10.1128/MCB.01837-08.
[S209] Jelluma, N., Brenkman, A.B., van den Broek, N.J.F., Cruijsen, C.W.A., van Osch, M.H.J., Lens, S.M.A., Medema, R.H., and Kops, G.J.P.L. (2008). Mps1 phosphorylates Borealin to control Aurora B activity and chromosome alignment. Cell 132, 233–246. 10.1016/j.cell.2007.11.046.
[S210] Jemaà, M., Galluzzi, L., Kepp, O., Senovilla, L., Brands, M., Boemer, U., Koppitz, M., Lienau, P., Prechtl, S., Schulze, V., et al. (2013). Characterization of novel MPS1 inhibitors with preclinical anticancer activity. Cell Death Differ 20, 1532–1545. 10.1038/cdd.2013.105.
[S211] Jiang, H., Yuan, F., Zhao, Z., Xue, T., Ge, N., Ren, Z., and Zhang, L. (2021). Expression and Clinical Significance of MPS-1 in Hepatocellular Carcinoma. Int J Gen Med 14, 9145–9152. 10.2147/IJGM.S334378.
[S212] Kagami, Y., Nihira, K., Wada, S., Ono, M., Honda, M., and Yoshida, K. (2014). Mps1 phosphorylation of condensin II controls chromosome condensation at the onset of mitosis. J Cell Biol 205, 781–790. 10.1083/jcb.201308172.
[S213] Kaistha, B.P., Honstein, T., Muller, V., Bielak, S., Sauer, M., Kreider, R., Fassan, M., Scarpa, A., Schmees, C., Volkmer, H., et al. (2014). Key role of dual specificity kinase TTK in proliferation and survival of pancreatic cancer cells. Br J Cancer 111, 1780–1787. 10.1038/bjc.2014.460.
[S214] Kessler, A.F., Feldheim, J., Schmitt, D., Feldheim, J.J., Monoranu, C.M., Ernestus, R.-I., Löhr, M., and Hagemann, C. (2020). Monopolar Spindle 1 Kinase (MPS1/TTK) mRNA Expression is Associated with Earlier Development of Clinical Symptoms, Tumor Aggressiveness and Survival of Glioma Patients. Biomedicines 8, 192. 10.3390/biomedicines8070192.
[S215] Liang, X.D., Dai, Y.C., Li, Z.Y., Gan, M.F., Zhang, S.R., Yin, P., Lu, H.S., Cao, X.Q., Zheng, B.J., Bao, L.F., et al. (2014). Expression and function analysis of mitotic checkpoint genes identifies TTK as a potential therapeutic target for human hepatocellular carcinoma. PLoS One 9, e97739. 10.1371/journal.pone.0097739.
[S216] Ling, Y., Zhang, X., Bai, Y., Li, P., Wei, C., Song, T., Zheng, Z., Guan, K., Zhang, Y., Zhang, B., et al. (2014). Overexpression of Mps1 in colon cancer cells attenuates the spindle assembly checkpoint and increases aneuploidy. Biochem Biophys Res Commun 450, 1690–1695. 10.1016/j.bbrc.2014.07.071.
[S217] Longo, L.V.G., Hughes, T., McNeil-Laidley, B., Cottini, F., Hilinski, G., Merritt, E., and Benson, D.M. (2023). TTK/MPS1 inhibitor OSU-13 targets the mitotic checkpoint and is a potential therapeutic strategy for myeloma. Haematologica. 10.3324/haematol.2023.282838.
[S218] Lu, L., Wang, Y., Chen, J., Li, Y., Liang, Q., Li, F., Zhen, C., and Xie, K. (2021). Targeting Mps1 in combination with paclitaxel inhibits osteosarcoma progression by modulating spindle assembly checkpoint and Akt/mTOR signaling. Oncol Lett 22, 797. 10.3892/ol.2021.13058.
[S219] Maachani, U.B., Kramp, T., Hanson, R., Zhao, S., Celiku, O., Shankavaram, U., Colombo, R., Caplen, N.J., Camphausen, K., and Tandle, A. (2015). Targeting MPS1 Enhances

Radiosensitization of Human Glioblastoma by Modulating DNA Repair Proteins. Mol Cancer Res 13, 852–862. 10.1158/1541-7786.MCR-14-0462-T.
[S220] Maia, A.R.R., de Man, J., Boon, U., Janssen, A., Song, J.-Y., Omerzu, M., Sterrenburg, J.G., Prinsen, M.B.W., Willemsen-Seegers, N., de Roos, J. a. D.M., et al. (2015). Inhibition of the spindle assembly checkpoint kinase TTK enhances the efficacy of docetaxel in a triple-negative breast cancer model. Ann Oncol 26, 2180–2192. 10.1093/annonc/mdv293.
[S221] Maia, A.R.R., Linder, S., Song, J.-Y., Vaarting, C., Boon, U., Pritchard, C.E.J., Velds, A., Huijbers, I.J., van Tellingen, O., Jonkers, J., et al. (2018). Mps1 inhibitors synergise with low doses of taxanes in promoting tumour cell death by enhancement of errors in cell division. Br J Cancer 118, 1586–1595. 10.1038/s41416-018-0081-2.
[S222] Pachis, S.T., and Kops, G.J.P.L. (2018). Leader of the SAC: molecular mechanisms of Mps1/TTK regulation in mitosis. Open Biol 8, 180109. 10.1098/rsob.180109.
[S223] Sarangapani, K.K., Koch, L.B., Nelson, C.R., Asbury, C.L., and Biggins, S. (2021). Kinetochore-bound Mps1 regulates kinetochore-microtubule attachments via Ndc80 phosphorylation. J Cell Biol 220. 10.1083/jcb.202106130.
[S224] Sarwar, S., Morozov, V.M., Purayil, H., Daaka, Y., and Ishov, A.M. (2022). Inhibition of Mps1 kinase enhances taxanes efficacy in castration resistant prostate cancer. Cell Death Dis 13, 868. 10.1038/s41419-022-05312-8.
[S225] Schöffski, P., Awada, A., de la Bigne, A.-M., Felloussi, Z., Burbridge, M., Cantero, F., Colombo, R., Maruzzelli, S., Ammattatelli, K., de Jonge, M., et al. (2022). First-in-man, first-in-class phase I study with the monopolar spindle 1 kinase inhibitor S81694 administered intravenously in adult patients with advanced, metastatic solid tumours. Eur J Cancer 169, 135–145. 10.1016/j.ejca.2022.04.001.
[S226] Shiraishi, T., Terada, N., Zeng, Y., Suyama, T., Luo, J., Trock, B., Kulkarni, P., and Getzenberg, R.H. (2011). Cancer/Testis Antigens as potential predictors of biochemical recurrence of prostate cancer following radical prostatectomy. J Transl Med 9, 153. 10.1186/1479-5876-9-153.
[S227] Simon Serrano, S., Sime, W., Abassi, Y., Daams, R., Massoumi, R., and Jemaa, M. (2020). Inhibition of mitotic kinase Mps1 promotes cell death in neuroblastoma. Sci Rep 10, 11997. 10.1038/s41598-020-68829-y.
[S228] Stucke, V.M., Silljé, H.H.W., Arnaud, L., and Nigg, E.A. (2002). Human Mps1 kinase is required for the spindle assembly checkpoint but not for centrosome duplication. EMBO J 21, 1723–1732. 10.1093/emboj/21.7.1723.
[S229] Szymiczek, A., Carbone, M., Pastorino, S., Napolitano, A., Tanji, M., Minaai, M., Pagano, I., Mason, J.M., Pass, H.I., Bray, M.R., et al. (2017). Inhibition of the spindle assembly checkpoint kinase Mps-1 as a novel therapeutic strategy in malignant mesothelioma. Oncogene 36, 6501–6507. 10.1038/onc.2017.266.
[S230] Tannous, B.A., Kerami, M., Van der Stoop, P.M., Kwiatkowski, N., Wang, J., Zhou, W., Kessler, A.F., Lewandrowski, G., Hiddingh, L., Sol, N., et al. (2013). Effects of the selective MPS1 inhibitor MPS1-IN-3 on glioblastoma sensitivity to antimitotic drugs. J Natl Cancer Inst 105, 1322–1331. 10.1093/jnci/djt168.
[S231] Tardif, K.D., Rogers, A., Cassiano, J., Roth, B.L., Cimbora, D.M., McKinnon, R., Peterson, A., Douce, T.B., Robinson, R., Dorweiler, I., et al. (2011). Characterization of the cellular and antitumor effects of MPI-0479605, a small-molecule inhibitor of the mitotic kinase Mps1. Mol Cancer Ther 10, 2267–2275. 10.1158/1535-7163.MCT-11-0453.
[S232] Waenphimai, O., Mahalapbutr, P., Vaeteewoottacharn, K., Wongkham, S., and Sawanyawisuth, K. (2022). Multiple actions of NMS-P715, the monopolar spindle 1 (MPS1) mitotic checkpoint inhibitor in liver fluke-associated cholangiocarcinoma cells. Eur J Pharmacol 922, 174899. 10.1016/j.ejphar.2022.174899.
[S233] Wengner, A.M., Siemeister, G., Koppitz, M., Schulze, V., Kosemund, D., Klar, U., Stoeckigt, D., Neuhaus, R., Lienau, P., Bader, B., et al. (2016). Novel Mps1 Kinase Inhibitors with Potent Antitumor Activity. Mol Cancer Ther 15, 583–592. 10.1158/1535-7163.MCT-15-0500.
[S234] Xu, Q., Xu, Y., Pan, B., Wu, L., Ren, X., Zhou, Y., Mao, F., Lin, Y., Guan, J., Shen, S., et al. (2016). TTK is a favorable prognostic biomarker for triple-negative breast cancer survival. Oncotarget 7, 81815–81829. 10.18632/oncotarget.13245.
[S235] Yu, L., Lang, Y., Hsu, C.C., Chen, W.M., Chiang, J.C., Hsieh, J.T., Story, M.D., Shang, Z.F., Chen, B.P.C., and Saha, D. (2022). Mitotic phosphorylation of tumor suppressor DAB2IP maintains spindle assembly checkpoint and chromosomal stability through activating PLK1-Mps1 signal

pathway and stabilizing mitotic checkpoint complex. Oncogene 41, 489–501. 10.1038/s41388-021-02106-8.
[S236] Yu, Z.-C., Huang, Y.-F., and Shieh, S.-Y. (2016). Requirement for human Mps1/TTK in oxidative DNA damage repair and cell survival through MDM2 phosphorylation. Nucleic Acids Res 44, 1133–1150. 10.1093/nar/gkv1173.
[S237] Zhang, L., Jiang, B., Zhu, N., Tao, M., Jun, Y., Chen, X., Wang, Q., and Luo, C. (2019). Mitotic checkpoint kinase Mps1/TTK predicts prognosis of colon cancer patients and regulates tumor proliferation and differentiation via PKCalpha/ERK1/2 and PI3K/Akt pathway. Med Oncol 37, 5. 10.1007/s12032-019-1320-y.
[S238] Zheng, L., Chen, Z., Kawakami, M., Chen, Y., Roszik, J., Mustachio, L.M., Kurie, J.M., Villalobos, P., Lu, W., Behrens, C., et al. (2019). Tyrosine Threonine Kinase Inhibition Eliminates Lung Cancers by Augmenting Apoptosis and Polyploidy. Mol Cancer Ther 18, 1775–1786. 10.1158/1535-7163.MCT-18-0864.
[S239] Chen, Y.T., Panarelli, N.C., Piotti, K.C., and Yantiss, R.K. (2014). Cancer-testis antigen expression in digestive tract carcinomas: frequent expression in esophageal squamous cell carcinoma and its precursor lesions. Cancer Immunol Res 2, 480–486. 10.1158/2326-6066.CIR-13-0124.
[S240] Pan, J., Eckardt, S., Leu, N.A., Buffone, M.G., Zhou, J., Gerton, G.L., McLaughlin, K.J., and Wang, P.J. (2009). Inactivation of Nxf2 causes defects in male meiosis and age-dependent depletion of spermatogonia. Dev Biol 330, 167–174. 10.1016/j.ydbio.2009.03.022.
[S241] Piotti, K.C., Scognamiglio, T., Chiu, R., and Chen, Y.T. (2013). Expression of cancer/testis (CT) antigens in squamous cell carcinoma of the head and neck: evaluation as markers of squamous dysplasia. Pathol Res Pract 209, 721–726. 10.1016/j.prp.2013.08.004.
[S242] Fujita, Y., Hayashi, T., Kiyomitsu, T., Toyoda, Y., Kokubu, A., Obuse, C., and Yanagida, M. (2007). Priming of centromere for CENP-A recruitment by human hMis18alpha, hMis18beta, and M18BP1. Dev Cell 12, 17–30. 10.1016/j.devcel.2006.11.002.
[S243] Gong, M., Li, Y., Song, E., Li, M., Qiu, S., Dong, W., and Yuan, R. (2022). OIP5 Is a Novel Prognostic Biomarker in Clear Cell Renal Cell Cancer Correlating With Immune Infiltrates. Front Immunol 13, 805552. 10.3389/fimmu.2022.805552.
[S244] He, J., Zhao, Y., Zhao, E., Wang, X., Dong, Z., Chen, Y., Yang, L., and Cui, H. (2018). Cancer-testis specific gene OIP5: a downstream gene of E2F1 that promotes tumorigenesis and metastasis in glioblastoma by stabilizing E2F1 signaling. Neuro Oncol 20, 1173–1184. 10.1093/neuonc/noy037.
[S245] He, X., Hou, J., Ping, J., Wen, D., and He, J. (2017). Opa interacting protein 5 acts as an oncogene in bladder cancer. J Cancer Res Clin Oncol 143, 2221–2233. 10.1007/s00432-017-2485-4.
[S246] Li, Y., Xiao, F., Li, W., Hu, P., Xu, R., Li, J., Li, G., and Zhu, C. (2019). Overexpression of Opa interacting protein 5 increases the progression of liver cancer via BMPR2/JUN/CHEK1/RAC1 dysregulation. Oncol Rep 41, 2075–2088. 10.3892/or.2019.7006.
[S247] Pan, M., Wang, Y., Wang, Z., Duan, H., Shao, C., Ding, P., Lei, J., Zhao, J., Ma, Z., Zhang, F., et al. (2023). The mitosis-related gene OIP5 is a potential biomarker in pan-cancer. Ann Transl Med 11, 117. 10.21037/atm-22-6640.
[S248] Rodrigues-Junior, D.M., Biassi, T.P., Carlin, V., Buri, M.V., Torrecilhas, A.C., Bortoluci, K.R., and Vettore, A.L. (2018). OIP5 Expression Sensitize Glioblastoma Cells to Lomustine Treatment. J Mol Neurosci 66, 383–389. 10.1007/s12031-018-1184-1.
[S249] Zhang, X., Gu, W., Lin, A., Duan, R., Lian, L., Huang, Y., Li, T., and Sun, Q. (2023). The role of OIP5 in the carcinogenesis and progression of ovarian cancer. J Ovarian Res 16, 185. 10.1186/s13048-023-01265-4.
[S250] Zhu, M., Takano, A., Tsevegjav, B., Yoshitake, Y., Shinohara, M., and Daigo, Y. (2022). Characterization of Opa interacting protein 5 as a new biomarker and therapeutic target for oral cancer. Int J Oncol 60. 10.3892/ijo.2022.5317.
[S251] Kulkarni, P., Dunker, A.K., Weninger, K., and Orban, J. (2016). Prostate-associated gene 4 (PAGE4), an intrinsically disordered cancer/testis antigen, is a novel therapeutic target for prostate cancer. Asian J Androl 18, 695–703. 10.4103/1008-682X.181818.
[S252] Lv, C., Fu, S., Dong, Q., Yu, Z., Zhang, G., Kong, C., Fu, C., and Zeng, Y. (2019). PAGE4 promotes prostate cancer cells survive under oxidative stress through modulating MAPK/JNK/ERK pathway. J Exp Clin Cancer Res 38, 24. 10.1186/s13046-019-1032-3.

[S253] Molania, R., Mahjoubi, F., Mirzaei, R., Khatami, S.-R., and Mahjoubi, B. (2014). A Panel of Cancer Testis Antigens and Clinical Risk Factors to Predict Metastasis in Colorectal Cancer. J Biomark 2014, 272683. 10.1155/2014/272683.
[S254] Sampson, N., Ruiz, C., Zenzmaier, C., Bubendorf, L., and Berger, P. (2012). PAGE4 positivity is associated with attenuated AR signaling and predicts patient survival in hormone-naive prostate cancer. Am J Pathol 181, 1443–1454. 10.1016/j.ajpath.2012.06.040.
[S255] Suyama, T., Shiraishi, T., Zeng, Y., Yu, W., Parekh, N., Vessella, R.L., Luo, J., Getzenberg, R.H., and Kulkarni, P. (2010). Expression of cancer/testis antigens in prostate cancer is associated with disease progression. Prostate 70, 1778–1787. 10.1002/pros.21214.
[S256] Zeng, Y., Gao, D., Kim, J.J., Shiraishi, T., Terada, N., Kakehi, Y., Kong, C., Getzenberg, R.H., and Kulkarni, P. (2013). Prostate-associated gene 4 (PAGE4) protects cells against stress by elevating p21 and suppressing reactive oxygen species production. Am J Clin Exp Urol 1, 39–52.
[S257] Chang, C.-F., Chen, S.-L., Sung, W.-W., Hsieh, M.-J., Hsu, H.-T., Chen, L.-H., Chen, M.-K., Ko, J.-L., Chen, C.-J., and Chou, M.-C. (2016). PBK/TOPK Expression Predicts Prognosis in Oral Cancer. Int J Mol Sci 17, 1007. 10.3390/ijms17071007.
[S258] Chen, J.H., Liang, Y.X., He, H.C., Chen, J.Y., Lu, J.M., Chen, G., Lin, Z.Y., Fu, X., Ling, X.H., Han, Z.D., et al. (2015). Overexpression of PDZ-binding kinase confers malignant phenotype in prostate cancer via the regulation of E2F1. Int J Biol Macromol 81, 615–623. 10.1016/j.ijbiomac.2015.08.048.
[S259] Deng, Y., Wen, H., Yang, H., Zhu, Z., Huang, Q., Bi, Y., Wang, P., Zhou, M., Guan, J., Zhang, W., et al. (2022). Identification of PBK as a hub gene and potential therapeutic target for medulloblastoma. Oncol Rep 48, 125. 10.3892/or.2022.8336.
[S260] Dong, C., Fan, W., and Fang, S. (2020). PBK as a Potential Biomarker Associated with Prognosis of Glioblastoma. J Mol Neurosci 70, 56–64. 10.1007/s12031-019-01400-1.
[S261] Feng, T., Zhang, Y., Ling, S., Xu, C., Lyu, Y., Lu, T., Liu, X., Ying, L., Wan, Y., Zhong, H., et al. (2021). PDZ Binding Kinase/T-LAK Cell-Derived Protein Kinase Plays an Oncogenic Role and Promotes Immune Escape in Human Tumors. J Oncol 2021, 8892479. 10.1155/2021/8892479.
[S262] Gao, T., Hu, Q., Hu, X., Lei, Q., Feng, Z., Yu, X., Peng, C., Song, X., He, H., Xu, Y., et al. (2019). Novel selective TOPK inhibitor SKLB-C05 inhibits colorectal carcinoma growth and metastasis. Cancer Lett 445, 11–23. 10.1016/j.canlet.2018.12.016.
[S263] Han, Z., Li, L., Huang, Y., Zhao, H., and Luo, Y. (2021). PBK/TOPK: A Therapeutic Target Worthy of Attention. Cells 10, 371. 10.3390/cells10020371.
[S264] Hayashi, T., Hayakawa, Y., Koh, M., Tomita, T., Nagai, S., Kashiwazaki, D., Sugimori, M., Origasa, H., and Kuroda, S. (2018). Impact of a novel biomarker, T-LAK cell-originating protein kinase (TOPK) expression on outcome in malignant glioma. Neuropathology 38, 144–153. 10.1111/neup.12446.
[S265] He, F., Yan, Q., Fan, L., Liu, Y., Cui, J., Wang, J., Wang, L., Wang, Y., Wang, Z., Guo, Y., et al. (2010). PBK/TOPK in the differential diagnosis of cholangiocarcinoma from hepatocellular carcinoma and its involvement in prognosis of human cholangiocarcinoma. Hum Pathol 41, 415–424. 10.1016/j.humpath.2009.05.016.
[S266] Herbert, K.J., Puliyadi, R., Prevo, R., Rodriguez-Berriguete, G., Ryan, A., Ramadan, K., and Higgins, G.S. (2021). Targeting TOPK sensitises tumour cells to radiation-induced damage by enhancing replication stress. Cell Death Differ 28, 1333–1346. 10.1038/s41418-020-00655-1.
[S267] Hu, F., Gartenhaus, R.B., Eichberg, D., Liu, Z., Fang, H.B., and Rapoport, A.P. (2010). PBK/TOPK interacts with the DBD domain of tumor suppressor p53 and modulates expression of transcriptional targets including p21. Oncogene 29, 5464–5474. 10.1038/onc.2010.275.
[S268] Hu, F., Gartenhaus, R.B., Zhao, X.F., Fang, H.-B., Minkove, S., Poss, D.E., and Rapoport, A.P. (2013). c-Myc and E2F1 drive PBK/TOPK expression in high-grade malignant lymphomas. Leuk Res 37, 447–454. 10.1016/j.leukres.2012.11.010.
[S269] Huang, H., Lee, M.H., Liu, K., Dong, Z., Ryoo, Z., and Kim, M.O. (2021). PBK/TOPK: An Effective Drug Target with Diverse Therapeutic Potential. Cancers (Basel) 13. 10.3390/cancers13092232.
[S270] Ikeda, Y., Park, J.-H., Miyamoto, T., Takamatsu, N., Kato, T., Iwasa, A., Okabe, S., Imai, Y., Fujiwara, K., Nakamura, Y., et al. (2016). T-LAK Cell-Originated Protein Kinase (TOPK) as a Prognostic Factor and a Potential Therapeutic Target in Ovarian Cancer. Clin Cancer Res 22, 6110–6117. 10.1158/1078-0432.CCR-16-0207.

[S271] Ishikawa, C., Senba, M., and Mori, N. (2018). Mitotic kinase PBK/TOPK as a therapeutic target for adult T-cell leukemia/lymphoma. Int J Oncol 53, 801–814. 10.3892/ijo.2018.4427.

[S272] Kar, A., Zhang, Y., Yacob, B.W., Saeed, J., Tompkins, K.D., Bagby, S.M., Pitts, T.M., Somerset, H., Leong, S., Wierman, M.E., et al. (2019). Targeting PDZ-binding kinase is anti-tumorigenic in novel preclinical models of ACC. Endocr Relat Cancer 26, 765–778. 10.1530/ERC-19-0262.

[S273] Kwon, C.H., Park, H.J., Choi, Y.R., Kim, A., Kim, H.W., Choi, J.H., Hwang, C.S., Lee, S.J., Choi, C.I., Jeon, T.Y., et al. (2016). PSMB8 and PBK as potential gastric cancer subtype-specific biomarkers associated with prognosis. Oncotarget 7, 21454–21468. 10.18632/oncotarget.7411.

[S274] Lee, D.H., Jeong, Y.J., Won, J.Y., Sim, H.I., Park, Y., and Jin, H.S. (2022). PBK/TOPK Is a Favorable Prognostic Biomarker Correlated with Antitumor Immunity in Colon Cancers. Biomedicines 10. 10.3390/biomedicines10020299.

[S275] Lei, B., Liu, S., Qi, W., Zhao, Y., Li, Y., Lin, N., Xu, X., Zhi, C., Mei, J., Yan, Z., et al. (2013). PBK/TOPK expression in non-small-cell lung cancer: its correlation and prognostic significance with Ki67 and p53 expression. Histopathology 63, 696–703. 10.1111/his.12215.

[S276] Lei, B., Qi, W., Zhao, Y., Li, Y., Liu, S., Xu, X., Zhi, C., Wan, L., and Shen, H. (2015). PBK/TOPK expression correlates with mutant p53 and affects patients' prognosis and cell proliferation and viability in lung adenocarcinoma. Hum Pathol 46, 217–224. 10.1016/j.humpath.2014.07.026.

[S277] Li, F., Liu, C., Nong, W., Lin, L., Ge, Y., Luo, B., Xiao, S., Zhang, Q., and Xie, X. (2023). Identification of potential biomarkers in cancer testis antigens for glioblastoma. Am J Transl Res 15, 799–816.

[S278] Li, J., and Hou, W. (2021). Expression patterns and clinical significances of PBK in lung cancer: an analysis based on Oncomine database. Transl Cancer Res 10, 2036–2043. 10.21037/tcr-20-3435.

[S279] Liu, K., Chen, Y., Feng, P., Wang, Y., Sun, M., Song, T., Tan, J., Li, C., Liu, S., Kong, Q., et al. (2022). Identification of Pathologic and Prognostic Genes in Prostate Cancer Based on Database Mining. Front Genet 13, 854531. 10.3389/fgene.2022.854531.

[S280] Liu, Y., Liu, H., Cao, H., Song, B., Zhang, W., and Zhang, W. (2015). PBK/TOPK mediates promyelocyte proliferation via Nrf2-regulated cell cycle progression and apoptosis. Oncol Rep 34, 3288–3296. 10.3892/or.2015.4308.

[S281] Liu, Y., Xiang, J., Peng, G., and Shen, C. (2021). Omics- and Pharmacogenomic Evidence for the Prognostic, Regulatory, and Immune-Related Roles of PBK in a Pan-Cancer Cohort. Front Mol Biosci 8, 785370. 10.3389/fmolb.2021.785370.

[S282] Luo, Q., Lei, B., Liu, S., Chen, Y., Sheng, W., Lin, P., Li, W., Zhu, H., and Shen, H. (2014). Expression of PBK/TOPK in cervical cancer and cervical intraepithelial neoplasia. Int J Clin Exp Pathol 7, 8059–8064.

[S283] Ma, H., Han, F., Yan, X., Qi, G., Li, Y., Li, R., Yan, S., Yuan, C., Song, K., and Kong, B. (2021). PBK promotes aggressive phenotypes of cervical cancer through ERK/c-Myc signaling pathway. J Cell Physiol 236, 2767–2781. 10.1002/jcp.30134.

[S284] Ma, H., Li, Y., Wang, X., Wu, H., Qi, G., Li, R., Yang, N., Gao, M., Yan, S., Yuan, C., et al. (2019). PBK, targeted by EVI1, promotes metastasis and confers cisplatin resistance through inducing autophagy in high-grade serous ovarian carcinoma. Cell Death Dis 10, 166. 10.1038/s41419-019-1415-6.

[S285] Ma, H., Qi, G., Han, F., Peng, J., Yuan, C., and Kong, B. (2022). PBK drives PARP inhibitor resistance through the TRIM37/NFκB axis in ovarian cancer. Exp Mol Med 54, 999–1010. 10.1038/s12276-022-00809-w.

[S286] Ma, H., Zhang, J., Shi, Y., Wang, Z., Nie, W., Cai, J., Huang, Y., Liu, B., Wang, X., and Lian, C. (2023). PBK correlates with prognosis, immune escape and drug response in LUAD. Sci Rep 13, 20452. 10.1038/s41598-023-47781-7.

[S287] Mao, P., Bao, G., Wang, Y.-C., Du, C.-W., Yu, X., Guo, X.-Y., Li, R.-C., and Wang, M.-D. (2020). PDZ-Binding Kinase-Dependent Transcriptional Regulation of CCNB2 Promotes Tumorigenesis and Radio-Resistance in Glioblastoma. Transl Oncol 13, 287–294. 10.1016/j.tranon.2019.09.011.

[S288] Mu, W., Xie, Y., Li, J., Yan, R., Zhang, J., Liu, Y., and Fan, Y. (2022). High expression of PDZ-binding kinase is correlated with poor prognosis and immune infiltrates in hepatocellular carcinoma. World J Surg Oncol 20, 22. 10.1186/s12957-021-02479-w.

[S289] Nagano-Matsuo, A., Inoue, S., Koshino, A., Ota, A., Nakao, K., Komura, M., Kato, H., Naiki-Ito, A., Watanabe, K., Nagayasu, Y., et al. (2021). PBK expression predicts favorable survival in colorectal cancer patients. Virchows Arch 479, 277–284. 10.1007/s00428-021-03062-0.

[S290] Nandi, A., Tidwell, M., Karp, J., and Rapoport, A.P. (2004). Protein expression of PDZ-binding kinase is up-regulated in hematologic malignancies and strongly down-regulated during terminal differentiation of HL-60 leukemic cells. Blood Cells Mol Dis 32, 240–245. 10.1016/j.bcmd.2003.10.004.
[S291] Ohashi, T., Komatsu, S., Ichikawa, D., Miyamae, M., Okajima, W., Imamura, T., Kiuchi, J., Kosuga, T., Konishi, H., Shiozaki, A., et al. (2017). Overexpression of PBK/TOPK relates to tumour malignant potential and poor outcome of gastric carcinoma. Br J Cancer 116, 218–226. 10.1038/bjc.2016.394.
[S292] Ohashi, T., Komatsu, S., Ichikawa, D., Miyamae, M., Okajima, W., Imamura, T., Kiuchi, J., Nishibeppu, K., Kosuga, T., Konishi, H., et al. (2016). Overexpression of PBK/TOPK Contributes to Tumor Development and Poor Outcome of Esophageal Squamous Cell Carcinoma. Anticancer Res 36, 6457–6466. 10.21873/anticanres.11244.
[S293] Park, J.H., Lin, M.L., Nishidate, T., Nakamura, Y., and Katagiri, T. (2006). PDZ-binding kinase/T-LAK cell-originated protein kinase, a putative cancer/testis antigen with an oncogenic activity in breast cancer. Cancer Res 66, 9186–9195. 10.1158/0008-5472.CAN-06-1601.
[S294] Park, J.-H., Park, S.-A., Lee, Y.-J., Park, H.-W., and Oh, S.-M. (2020). PBK attenuates paclitaxel-induced autophagic cell death by suppressing p53 in H460 non-small-cell lung cancer cells. FEBS Open Bio 10, 937–950. 10.1002/2211-5463.12855.
[S295] Qiao, L., Ba, J., Xie, J., Zhu, R., Wan, Y., Zhang, M., Jin, Z., Guo, Z., Yu, J., Chen, S., et al. (2022). Overexpression of PBK/TOPK relates to poor prognosis of patients with breast cancer: a retrospective analysis. World J Surg Oncol 20, 316. 10.1186/s12957-022-02769-x.
[S296] Shih, M.-C., Chen, J.-Y., Wu, Y.-C., Jan, Y.-H., Yang, B.-M., Lu, P.-J., Cheng, H.-C., Huang, M.-S., Yang, C.-J., Hsiao, M., et al. (2012). TOPK/PBK promotes cell migration via modulation of the PI3K/PTEN/AKT pathway and is associated with poor prognosis in lung cancer. Oncogene 31, 2389–2400. 10.1038/onc.2011.419.
[S297] Singh, P.K., Srivastava, A.K., Dalela, D., Rath, S.K., Goel, M.M., and Bhatt, M.L.B. (2014). Expression of PDZ-binding kinase/T-LAK cell-originated protein kinase (PBK/TOPK) in human urinary bladder transitional cell carcinoma. Immunobiology 219, 469–474. 10.1016/j.imbio.2014.02.003.
[S298] Su, T.C., Chen, C.Y., Tsai, W.C., Hsu, H.T., Yen, H.H., Sung, W.W., and Chen, C.J. (2018). Cytoplasmic, nuclear, and total PBK/TOPK expression is associated with prognosis in colorectal cancer patients: A retrospective analysis based on immunohistochemistry stain of tissue microarrays. PLoS One 13, e0204866. 10.1371/journal.pone.0204866.
[S299] Thanindratarn, P., Wei, R., Dean, D.C., Singh, A., Federman, N., Nelson, S.D., Hornicek, F.J., and Duan, Z. (2021). T-LAK cell-originated protein kinase (TOPK): an emerging prognostic biomarker and therapeutic target in osteosarcoma. Mol Oncol 15, 3721–3737. 10.1002/1878-0261.13039.
[S300] Wang, K., Chai, J., Xu, J., Wei, J., Li, P., Liu, Y., Ma, J., Xu, T., Zhao, D., Yu, K., et al. (2021). TOPK: A new predictor of the therapeutic response to neoadjuvant chemotherapy and prognosis in triple-negative breast cancer. Pathol Res Pract 226, 153603. 10.1016/j.prp.2021.153603.
[S301] Wen, H., Chen, Z., Li, M., Huang, Q., Deng, Y., Zheng, J., Xiong, M., Wang, P., and Zhang, W. (2021). An Integrative Pan-Cancer Analysis of PBK in Human Tumors. Front Mol Biosci 8, 755911. 10.3389/fmolb.2021.755911.
[S302] Wu, W., Xu, J., Gao, D., Xie, Z., Chen, W., Li, W., Yuan, Q., Duan, L., Zhang, Y., Yang, X., et al. (2023). TOPK promotes the growth of esophageal cancer in vitro and in vivo by enhancing YB1/eEF1A1 signal pathway. Cell Death Dis 14, 364. 10.1038/s41419-023-05883-0.
[S303] Yu, W.-N., Lin, H.-F., Lee, Y.I., Shia, W.-C., Sung, W.-W., Yeh, C.-M., and Lin, Y.-M. (2021). PBK Expression Is Associated With Prognosis of Patients With Oral Squamous Cell Carcinoma Treated With Radiotherapy: A Retrospective Study. Anticancer Res 41, 2177–2182. 10.21873/anticanres.14991.
[S304] Zhang, Y., Yang, X., Wang, R., and Zhang, X. (2019). Prognostic Value of PDZ-Binding Kinase/T-LAK Cell-Originated Protein Kinase (PBK/TOPK) in Patients with Cancer. J Cancer 10, 131–137. 10.7150/jca.28216.
[S305] Zheng, L., Li, L., Xie, J., Jin, H., and Zhu, N. (2021). Six Novel Biomarkers for Diagnosis and Prognosis of Esophageal squamous cell carcinoma: validated by scRNA-seq and qPCR. J Cancer 12, 899–911. 10.7150/jca.50443.

[S306] Zhou, L., Zhang, Y., Wei, M., Du, K., Lin, J., and Wei, L. (2023). Comprehensive analysis of CXCL14 uncovers its role during liver metastasis in colon cancer. BMC Gastroenterol 23, 273. 10.1186/s12876-023-02896-z.
[S307] Gantchev, J., Martinez Villarreal, A., Gunn, S., Zetka, M., Odum, N., and Litvinov, I.V. (2020). The ectopic expression of meiCT genes promotes meiomitosis and may facilitate carcinogenesis. Cell Cycle 19, 837–854. 10.1080/15384101.2020.1743902.
[S308] Houle, A.A., Gibling, H., Lamaze, F.C., Edgington, H.A., Soave, D., Fave, M.J., Agbessi, M., Bruat, V., Stein, L.D., and Awadalla, P. (2018). Aberrant PRDM9 expression impacts the pan-cancer genomic landscape. Genome Res 28, 1611–1620. 10.1101/gr.231696.117.
[S309] Hussin, J., Sinnett, D., Casals, F., Idaghdour, Y., Bruat, V., Saillour, V., Healy, J., Grenier, J.C., de Malliard, T., Busche, S., et al. (2013). Rare allelic forms of PRDM9 associated with childhood leukemogenesis. Genome Res 23, 419–430. 10.1101/gr.144188.112.
[S310] Kaiser, V.B., and Semple, C.A. (2018). Chromatin loop anchors are associated with genome instability in cancer and recombination hotspots in the germline. Genome Biol 19, 101. 10.1186/s13059-018-1483-4.
[S311] Agarwal, S., Parashar, D., Gupta, N., Jagadish, N., Thakar, A., Suri, V., Kumar, R., Gupta, A., Ansari, A.S., Lohiya, N.K., et al. (2015). Sperm associated antigen 9 (SPAG9) expression and humoral response in benign and malignant salivary gland tumors. Oncoimmunology 3, e974382. 10.4161/2162402X.2014.974382.
[S312] Baser, E., Togrul, C., Ozgu, E., Ayhan, S., Caglar, M., Erkaya, S., and Gungor, T. (2013). Sperm-associated antigen 9 is a promising marker for early diagnosis of endometrial cancer. Asian Pac J Cancer Prev 14, 7635–7638. 10.7314/apjcp.2013.14.12.7635.
[S313] Bi, B.-A., C, R.C., N, A., Am, K., N, T., D, M., M, S., M, K.S., A, P., D, M., et al. (2023). An HLA-G/SPAG9/STAT3 axis promotes brain metastases. Proceedings of the National Academy of Sciences of the United States of America 120. 10.1073/pnas.2205247120.
[S314] Garg, M., Kanojia, D., Suri, S., Gupta, S., Gupta, A., and Suri, A. (2009). Sperm-associated antigen 9: a novel diagnostic marker for thyroid cancer. J Clin Endocrinol Metab 94, 4613–4618. 10.1210/jc.2009-0703.
[S315] Jagadish, N., Fatima, R., Sharma, A., Devi, S., Suri, V., Kumar, V., and Suri, A. (2018). Sperm associated antigen 9 (SPAG9) a promising therapeutic target of ovarian carcinoma. Tumour Biol 40, 1010428318773652. 10.1177/1010428318773652.
[S316] Kanojia, D., Garg, M., Gupta, S., Gupta, A., and Suri, A. (2011). Sperm-associated antigen 9 is a novel biomarker for colorectal cancer and is involved in tumor growth and tumorigenicity. Am J Pathol 178, 1009–1020. 10.1016/j.ajpath.2010.11.047.
[S317] Kanojia, D., Garg, M., Saini, S., Agarwal, S., Kumar, R., and Suri, A. (2010). Sperm associated antigen 9 expression and humoral response in chronic myeloid leukemia. Leuk Res 34, 858–863. 10.1016/j.leukres.2010.01.017.
[S318] Kanojia, D., Garg, M., Saini, S., Agarwal, S., Parashar, D., Jagadish, N., Seth, A., Bhatnagar, A., Gupta, A., Kumar, R., et al. (2013). Sperm associated antigen 9 plays an important role in bladder transitional cell carcinoma. PLoS One 8, e81348. 10.1371/journal.pone.0081348.
[S319] Li, H., Peng, Y., Niu, H., Wu, B., Zhang, Y., Zhang, Y., Bai, X., and He, P. (2014). SPAG9 is overexpressed in human prostate cancer and promotes cancer cell proliferation. Tumour Biol 35, 6949–6954. 10.1007/s13277-014-1947-4.
[S320] Luo, S., Ren, B., Zou, G., Liu, J., Chen, W., Huang, Y., Chen, X., and Fu, Y. (2019). SPAG9/MKK3/p38 axis is a novel therapeutic target for liver cancer. Oncol Rep 41, 2329–2336. 10.3892/or.2019.6987.
[S321] Miao, Z.F., Wang, Z.N., Zhao, T.T., Xu, Y.Y., Wu, J.H., Liu, X.Y., Xu, H., You, Y., and Xu, H.M. (2015). Overexpression of SPAG9 in human gastric cancer is correlated with poor prognosis. Virchows Arch 467, 525–533. 10.1007/s00428-015-1826-4.
[S322] Pan, J., Yu, H., Guo, Z., Liu, Q., Ding, M., Xu, K., and Mao, L. (2018). Emerging role of sperm-associated antigen 9 in tumorigenesis. Biomed Pharmacother 103, 1212–1216. 10.1016/j.biopha.2018.04.168.
[S323] Qiao, L., Zhang, L., and Wang, H. (2023). SPAG9 Expression Predicts Good Prognosis in Patients with Clear-Cell Renal Cell Carcinoma: A Bioinformatics Analysis with Experimental Validation. Genes (Basel) 14, 944. 10.3390/genes14040944.

[S324] Ren, B., Wei, X., Zou, G., He, J., Xu, G., Xu, F., Huang, Y., Zhu, H., Li, Y., Ma, G., et al. (2016). Cancer testis antigen SPAG9 is a promising marker for the diagnosis and treatment of lung cancer. Oncol Rep 35, 2599–2605. 10.3892/or.2016.4645.

[S325] Seleit, I., Bakry, O.A., Samaka, R.M., and Malak, M.A. (2015). Immunohistochemical expression of sperm-associated antigen 9 in nonmelanoma skin cancer. Am J Dermatopathol 37, 38–45. 10.1097/DAD.0000000000000126.

[S326] Sun, H.-F., Wang, W.-D., and Feng, L. (2017). Effect of SPAG9 on migration, invasion and prognosis of prostate cancer. Int J Clin Exp Pathol 10, 9468–9474.

[S327] Wang, Y., Dong, Q., Miao, Y., Fu, L., Lin, X., and Wang, E. (2013). Clinical significance and biological roles of SPAG9 overexpression in non-small cell lung cancer. Lung Cancer 81, 266–272. 10.1016/j.lungcan.2013.04.021.

[S328] Xiao, C., Fu, L., Yan, C., Shou, F., Liu, Q., Li, L., Cui, S., Duan, J., Jin, G., Chen, J., et al. (2016). SPAG9 is overexpressed in osteosarcoma, and regulates cell proliferation and invasion through regulation of JunD. Oncol Lett 12, 2674–2679. 10.3892/ol.2016.4920.

[S329] Xie, C., Fu, L., Liu, N., and Li, Q. (2014). Overexpression of SPAG9 correlates with poor prognosis and tumor progression in hepatocellular carcinoma. Tumour Biol 35, 7685–7691. 10.1007/s13277-014-2030-x.

[S330] Yan, Q., Yang, C., Fu, Q., Chen, Z., Liu, S., Fu, D., Rahman, R.N., Nakazato, R., Yoshioka, K., Kung, S.K.P., et al. (2017). Scaffold protein JLP mediates TCR-initiated CD4+T cell activation and CD154 expression. Mol Immunol 87, 258–266. 10.1016/j.molimm.2017.05.006.

[S331] Yang, C., Shen, B., Zhang, J., and Zhang, Q. (2016). Sperm-associated antigen 9 overexpression correlates with poor prognosis and insensitive to Taxol treatment in breast cancer. Biomarkers 21, 62–67. 10.3109/1354750X.2015.1118534.

[S332] Yi, F., Ni, W., Liu, W., Pan, X., Han, X., Yang, L., Kong, X., Ma, R., and Chang, R. (2013). SPAG9 is overexpressed in human astrocytoma and promotes cell proliferation and invasion. Tumour Biol 34, 2849–2855. 10.1007/s13277-013-0845-5.

[S333] Baudat, F., Manova, K., Yuen, J.P., Jasin, M., and Keeney, S. (2000). Chromosome synapsis defects and sexually dimorphic meiotic progression in mice lacking Spo11. Mol Cell 6, 989–998. 10.1016/s1097-2765(00)00098-8.

[S334] Eldai, H., Periyasamy, S., Al Qarni, S., Al Rodayyan, M., Muhammed Mustafa, S., Deeb, A., Al Sheikh, E., Afzal, M., Johani, M., Yousef, Z., et al. (2013). Novel genes associated with colorectal cancer are revealed by high resolution cytogenetic analysis in a patient specific manner. PLoS One 8, e76251. 10.1371/journal.pone.0076251.

[S335] Keeney, S. (2008). Spo11 and the Formation of DNA Double-Strand Breaks in Meiosis. Genome Dyn Stab 2, 81–123. 10.1007/7050_2007_026.

[S336] Lindsey, S.F., Byrnes, D.M., Eller, M.S., Rosa, A.M., Dabas, N., Escandon, J., and Grichnik, J.M. (2013). Potential role of meiosis proteins in melanoma chromosomal instability. J Skin Cancer 2013, 190109. 10.1155/2013/190109.

[S337] Litvinov, I.V., Cordeiro, B., Huang, Y., Zargham, H., Pehr, K., Dore, M.A., Gilbert, M., Zhou, Y., Kupper, T.S., and Sasseville, D. (2014). Ectopic expression of cancer-testis antigens in cutaneous T-cell lymphoma patients. Clin Cancer Res 20, 3799–3808. 10.1158/1078-0432.CCR-14-0307.

[S338] Nielsen, A.Y., and Gjerstorff, M.F. (2016). Ectopic Expression of Testis Germ Cell Proteins in Cancer and Its Potential Role in Genomic Instability. Int J Mol Sci 17. 10.3390/ijms17060890.

[S339] Wei, M., Su, J., Zhang, J., Liu, S., Ma, J., and Meng, X.P. (2023). Construction of a DDR-related signature for predicting of prognosis in metastatic colorectal carcinoma. Front Oncol 13, 1043160. 10.3389/fonc.2023.1043160.

[S340] Brückmann, N.H., Bennedsen, S.N., Duijf, P.H.G., Terp, M.G., Thomassen, M., Larsen, M., Pedersen, C.B., Kruse, T., Alcaraz, N., Ditzel, H.J., et al. (2019). A functional genetic screen identifies the Mediator complex as essential for SSX2-induced senescence. Cell Death Dis 10, 841. 10.1038/s41419-019-2068-1.

[S341] Cordier, F., Van der Meulen, J., Van Gaever, B., Lapeire, L., Sys, G., Van Dorpe, J., and Creytens, D. (2022). Undifferentiated sarcoma of bone with a round to epithelioid cell phenotype harboring a novel EWSR1-SSX2 fusion identified by RNA-based next-generation sequencing. Genes, Chromosomes and Cancer 61, 44–49. 10.1002/gcc.22999.

[S342] Eisenhardt, A.E., Brugger, Z., Lausch, U., Kiefer, J., Zeller, J., Runkel, A., Schmid, A., Bronsert, P., Wehrle, J., Leithner, A., et al. (2022). Genotyping of Circulating Free DNA Enables Monitoring of Tumor Dynamics in Synovial Sarcomas. Cancers (Basel) 14, 2078. 10.3390/cancers14092078.
[S343] Gjerstorff, M.F., Relster, M.M., Greve, K.B.V., Moeller, J.B., Elias, D., Lindgreen, J.N., Schmidt, S., Mollenhauer, J., Voldborg, B., Pedersen, C.B., et al. (2014). SSX2 is a novel DNA-binding protein that antagonizes polycomb group body formation and gene repression. Nucleic Acids Res 42, 11433–11446. 10.1093/nar/gku852.
[S344] Greve, K.B., Lindgreen, J.N., Terp, M.G., Pedersen, C.B., Schmidt, S., Mollenhauer, J., Kristensen, S.B., Andersen, R.S., Relster, M.M., Ditzel, H.J., et al. (2015). Ectopic expression of cancer/testis antigen SSX2 induces DNA damage and promotes genomic instability. Mol Oncol 9, 437–449. 10.1016/j.molonc.2014.09.001.
[S345] Traynor, S., Mollegaard, N.E., Jorgensen, M.G., Bruckmann, N.H., Pedersen, C.B., Terp, M.G., Johansen, S., Dejardin, J., Ditzel, H.J., and Gjerstorff, M.F. (2019). Remodeling and destabilization of chromosome 1 pericentromeric heterochromatin by SSX proteins. Nucleic Acids Res 47, 6668–6684. 10.1093/nar/gkz396.
[S346] Türeci, O., Sahin, U., Schobert, I., Koslowski, M., Scmitt, H., Schild, H.J., Stenner, F., Seitz, G., Rammensee, H.G., and Pfreundschuh, M. (1996). The SSX-2 gene, which is involved in the t(X;18) translocation of synovial sarcomas, codes for the human tumor antigen HOM-MEL-40. Cancer Res 56, 4766–4772.
[S347] Zaborowski, M., Vargas, A.C., Pulvers, J., Clarkson, A., de Guzman, D., Sioson, L., Maclean, F., Chou, A., and Gill, A.J. (2020). When used together SS18-SSX fusion-specific and SSX C-terminus immunohistochemistry are highly specific and sensitive for the diagnosis of synovial sarcoma and can replace FISH or molecular testing in most cases. Histopathology 77, 588–600. 10.1111/his.14190.
[S348] Zhang, Y., Bao, L., Lu, J., Liu, K.-Y., Li, J.-L., Qin, Y.-Z., Chen, H., Li, L.-D., Kong, Y., Shi, H.-X., et al. (2014). The clinical value of the quantitative detection of four cancer-testis antigen genes in multiple myeloma. Mol Cancer 13, 25. 10.1186/1476-4598-13-25.
[S349] Cho, H., Noh, K.H., Chung, J.-Y., Takikita, M., Chung, E.J., Kim, B.W., Hewitt, S.M., Kim, T.W., and Kim, J.-H. (2014). Synaptonemal complex protein 3 is a prognostic marker in cervical cancer. PLoS One 9, e98712. 10.1371/journal.pone.0098712.
[S350] Chung, J.Y., Kitano, H., Takikita, M., Cho, H., Noh, K.H., Kim, T.W., Ylaya, K., Hanaoka, J., Fukuoka, J., and Hewitt, S.M. (2013). Synaptonemal complex protein 3 as a novel prognostic marker in early stage non-small cell lung cancer. Hum Pathol 44, 472–479. 10.1016/j.humpath.2012.06.018.
[S351] Hosoya, N., and Miyagawa, K. (2021). Synaptonemal complex proteins modulate the level of genome integrity in cancers. Cancer Sci 112, 989–996. 10.1111/cas.14791.
[S352] Hosoya, N., Okajima, M., Kinomura, A., Fujii, Y., Hiyama, T., Sun, J., Tashiro, S., and Miyagawa, K. (2011). Synaptonemal complex protein SYCP3 impairs mitotic recombination by interfering with BRCA2. EMBO Rep 13, 44–51. 10.1038/embor.2011.221.
[S353] Kang, T.H., Noh, K.H., Kim, J.H., Bae, H.C., Lin, K.Y., Monie, A., Pai, S.I., Hung, C.F., Wu, T.C., and Kim, T.W. (2010). Ectopic expression of X-linked lymphocyte-regulated protein pM1 renders tumor cells resistant to antitumor immunity. Cancer Res 70, 3062–3070. 10.1158/0008-5472.CAN-09-3856.
[S354] Kitano, H., Chung, J.Y., Noh, K.H., Lee, Y.H., Kim, T.W., Lee, S.H., Eo, S.H., Cho, H.J., Choi, C.H., Inoue, S., et al. (2017). Synaptonemal complex protein 3 is associated with lymphangiogenesis in non-small cell lung cancer patients with lymph node metastasis. J Transl Med 15, 138. 10.1186/s12967-017-1241-5.
[S355] Mobasheri, M.B., Jahanzad, I., Mohagheghi, M.A., Aarabi, M., Farzan, S., and Modarressi, M.H. (2007). Expression of two testis-specific genes, TSGA10 and SYCP3, in different cancers regarding to their pathological features. Cancer Detect Prev 31, 296–302. 10.1016/j.cdp.2007.05.002.
[S356] Niemeyer, P., Tureci, O., Eberle, T., Graf, N., Pfreundschuh, M., and Sahin, U. (2003). Expression of serologically identified tumor antigens in acute leukemias. Leuk Res 27, 655–660. 10.1016/s0145-2126(02)00230-8.
[S357] Oh, S.J., Cho, H., Kim, S., Noh, K.H., Song, K.-H., Lee, H.-J., Woo, S.R., Kim, S., Choi, C.H., Chung, J.-Y., et al. (2018). Targeting Cyclin D-CDK4/6 Sensitizes Immune-Refractory Cancer by Blocking the SCP3–NANOG Axis. Cancer Res 78, 2638–2653. 10.1158/0008-5472.CAN-17-2325.

[S358] Oh, S.J., Noh, K.H., Song, K.-H., and Kim, T.W. (2021). Interaction between SCP3 and JAB1 Confers Cancer Therapeutic Resistance and Stem-like Properties through EGF Expression. Int J Mol Sci 22, 8839. 10.3390/ijms22168839.
[S359] Yuan, L., Liu, J.G., Zhao, J., Brundell, E., Daneholt, B., and Höög, C. (2000). The murine SCP3 gene is required for synaptonemal complex assembly, chromosome synapsis, and male fertility. Mol Cell 5, 73–83. 10.1016/s1097-2765(00)80404-9.
[S360] Sandhu, S., Sou, I.F., Hunter, J.E., Salmon, L., Wilson, C.L., Perkins, N.D., Hunter, N., Davies, O.R., and McClurg, U.L. (2021). Centrosome dysfunction associated with somatic expression of the synaptonemal complex protein TEX12. Commun Biol 4, 1371. 10.1038/s42003-021-02887-4.
[S361] Zhou, H., Wu, L., Yu, L., Yang, Y., Kong, L., Liu, S., Chen, W., and Li, R. (2022). Identify a DNA Damage Repair Gene Signature for Predicting Prognosis and Immunotherapy Response in Cervical Squamous Cell Carcinoma. J Oncol 2022, 8736575. 10.1155/2022/8736575.
[S362] Lin, X., Chen, Z., Gao, P., Gao, Z., Chen, H., Qi, J., Liu, F., Ye, D., Jiang, H., Na, R., et al. (2017). TEX15: A DNA repair gene associated with prostate cancer risk in Han Chinese. Prostate 77, 1271–1278. 10.1002/pros.23387.
[S363] Mantere, T., Tervasmaki, A., Nurmi, A., Rapakko, K., Kauppila, S., Tang, J., Schleutker, J., Kallioniemi, A., Hartikainen, J.M., Mannermaa, A., et al. (2017). Case-control analysis of truncating mutations in DNA damage response genes connects TEX15 and FANCD2 with hereditary breast cancer susceptibility. Sci Rep 7, 681. 10.1038/s41598-017-00766-9.
[S364] Okutman, O., Muller, J., Baert, Y., Serdarogullari, M., Gultomruk, M., Piton, A., Rombaut, C., Benkhalifa, M., Teletin, M., Skory, V., et al. (2015). Exome sequencing reveals a nonsense mutation in TEX15 causing spermatogenic failure in a Turkish family. Hum Mol Genet 24, 5581–5588. 10.1093/hmg/ddv290.
[S365] Yang, F., Eckardt, S., Leu, N.A., McLaughlin, K.J., and Wang, P.J. (2008). Mouse TEX15 is essential for DNA double-strand break repair and chromosomal synapsis during male meiosis. J Cell Biol 180, 673–679. 10.1083/jcb.200709057.
[S366] Huang, J., Wang, Y., Liu, J., Chu, M., and Wang, Y. (2021). TFDP3 as E2F Unique Partner, Has Crucial Roles in Cancer Cells and Testis. Front Oncol 11, 742462. 10.3389/fonc.2021.742462.
[S367] Ingram, L., Munro, S., Coutts, A.S., and La Thangue, N.B. (2011). E2F-1 regulation by an unusual DNA damage-responsive DP partner subunit. Cell Death Differ 18, 122–132. 10.1038/cdd.2010.70.
[S368] Jiao, Y., Ding, L., Chu, M., Wang, T., Kang, J., Zhao, X., Li, H., Chen, X., Gao, Z., Gao, L., et al. (2017). Effects of cancer-testis antigen, TFDP3, on cell cycle regulation and its mechanism in L-02 and HepG2 cell lines in vitro. PLoS One 12, e0182781. 10.1371/journal.pone.0182781.
[S369] Ma, Y., Xin, Y., Li, R., Wang, Z., Yue, Q., Xiao, F., and Hao, X. (2014). TFDP3 was expressed in coordination with E2F1 to inhibit E2F1-mediated apoptosis in prostate cancer. Gene 537, 253–259. 10.1016/j.gene.2013.12.051.
[S370] Qiao, H., Di Stefano, L., Tian, C., Li, Y.Y., Yin, Y.H., Qian, X.P., Pang, X.W., Li, Y., McNutt, M.A., Helin, K., et al. (2007). Human TFDP3, a novel DP protein, inhibits DNA binding and transactivation by E2F. J Biol Chem 282, 454–466. 10.1074/jbc.M606169200.
[S371] Tian, C., Lv, D., Qiao, H., Zhang, J., Yin, Y.H., Qian, X.P., Wang, Y.P., Zhang, Y., and Chen, W.F. (2007). TFDP3 inhibits E2F1-induced, p53-mediated apoptosis. Biochem Biophys Res Commun 361, 20–25. 10.1016/j.bbrc.2007.06.128.
[S372] Wang, X., Xing, Z., Xu, H., Yang, H., and Xing, T. (2021). Development and validation of epithelial mesenchymal transition-related prognostic model for hepatocellular carcinoma. Aging (Albany NY) 13, 13822–13845. 10.18632/aging.202976.
[S373] Zhang, D., Zhou, S., and Liu, B. (2020). Identification and Validation of an Individualized EMT-Related Prognostic Risk Score Formula in Gastric Adenocarcinoma Patients. Biomed Res Int 2020, 7082408. 10.1155/2020/7082408.
